# Supplementary material for: Barriers and facilitators to patient uptake and utilisation of digital interventions for the self-management of low back pain: a systematic review of qualitative studies
Source: BMJ Open. 2020 Dec 12;10(12):e038800. doi: 10.1136/bmjopen-2020-038800 (PMC7735096; doi:10.1136/bmjopen-2020-038800)
Supplement: Supplementary data [file bmjopen-2020-038800supp001.pdf]

Supplementary File 1: Search details

MEDLINE - search details

Ovid MEDLINE(R) 1946 to March Week 1 2016

1	exp back pain/(back pain$ or lumbago or back ache$ or backache$ or (lumbar adj2 pain$) or (spin$ adj2 pain$)).ti,ab,kw,kf.
2	computer peripherals/ or computer storage devices/ or computer terminals/ or modems/ or microcomputers/ or computers, handheld/ or minicomputers/ or attitude to computers/ or computers/ or computer systems/ or medical informatics/ or medical informatics applications/ or educational technology/ or audiovisual aids/ or telecommunications/ or multimedia/ or computer-assisted instruction/ or user-computer interface/ or hypermedia/ or video games/ or electronic health records/ or social networking/ or (computer$ or microcomputer$ or pc or pcs or mac or macs or internet or www or web or website$ or webpage$ or local area network$).ti,ab,kf. or software.ti,ab,kf. or (cellular phone$ or cellular telephone$ or mobile$ or cell phone$ or cell telephone$ or smartphone$ or smart-phone$ or smart-telephone$).ti,ab,kf. or (handset$ or hand-set$ or wireless or wire-less or wifi or wi-fi or gps or global positioning system$ or bluetooth or text messag$ or texting or sms or short messag$ or multimedia messag$ or multi-media messag$ or mms or instant messag$ or social media$ or facebook or twitter or webcast$ or webinar$ or podcast$ or wiki or wikis or app or apps or android$ or blackberr$ or apple$ or ios or iphone$ or ipad$ or s40 or symbian$ or windows).ti,ab,kf. or ((electronic$ or digital$ or device$) adj2 tablet$).ti,ab,kf. or (video$ or dvd or dvds).ti,ab,kf. or (youtube or you tube or vimeo).ti,ab,kf. or (online or on line or interactive).ti,ab,kf. or (chat room$ or chatroom$).ti,ab,kf. or (blog$1 or web-log$1 or weblog$1).ti,ab,kf. or (bulletin board$ or bulletinboard$ or messageboard$ or message board$).ti,ab,kf. or (ehealth or e-health or mhealth or m-health).ti,ab,kf. or exp telemedicine/ or mobile applications/ or (pda or pdas or personal digital).ti,ab,kf. or device-based.ti,ab,kf. or (email$ or e-mail$ or electronic mail$).ti,ab,kw,kf.
3	1 and 2
4	limit 3 to yr="2000 -Current"

Updated searches: 1) October 21 2016 (not shown), 2) December 18 2018 (below):

1	exp back pain/(back pain$ or lumbago or back ache$ or backache$ or (lumbar adj2 pain$) or (spin$ adj2 pain$)).ti,ab,kw,kf.
2	computer peripherals/ or computer storage devices/ or computer terminals/ or modems/ or microcomputers/ or computers, handheld/ or minicomputers/ or attitude to computers/ or computers/ or computer systems/ or medical informatics/ or medical informatics applications/ or educational technology/ or audiovisual aids/ or telecommunications/ or multimedia/ or computer-assisted instruction/ or user-computer interface/ or hypermedia/ or video games/ or electronic health records/ or social networking/
3	(computer$ or microcomputer$ or pc or pcs or mac or macs or internet or www or web or website$ or webpage$ or local area network$).ti,ab,kf. or software.ti,ab,kf. or (cellular phone$ or cellular telephone$ or mobile$ or cell phone$ or cell telephone$ or smartphone$ or smart-phone$ or smart-telephone$).ti,ab,kf. or (handset$ or hand-set$ or wireless or wire-less or wifi or wi-fi or gps or global positioning system$ or bluetooth or text messag$ or texting or sms or short messag$ or multimedia messag$ or multi-media messag$ or mms or instant messag$ or social media$ or facebook or twitter or webcast$ or webinar$ or podcast$ or wiki or wikis or app or apps or android$ or blackberr$ or apple$ or ios or iphone$ or ipad$ or s40 or symbian$ or windows).ti,ab,kf. or ((electronic$ or digital$ or device$) adj2 tablet$).ti,ab,kf. or (video$ or dvd or dvds).ti,ab,kf. or (youtube or you tube or vimeo).ti,ab,kf. or (online or on line or interactive).ti,ab,kf. or (chat room$ or chatroom$).ti,ab,kf. or (blog$1 or web-log$1 or weblog$1).ti,ab,kf. or (bulletin board$ or bulletinboard$ or messageboard$ or message board$).ti,ab,kf. or (ehealth or e-health or mhealth or m-health).ti,ab,kf. or exp telemedicine/ or mobile applications/ or (pda or pdas or personal digital).ti,ab,kf. or device-based.ti,ab,kf. or (email$ or e-mail$ or electronic mail$).ti,ab,kw,kf.
4	1 and (2 or 3)
5	limit 4 to yr="2000 -Current"
6	5 and (201610* or 201611* or 2017* or 2018*).ed.

Embase - search details

Ovid Embase (R) 1974 to 2016 March 18

1	exp backache/ or (spinal pain$ or back pain$ or lumbago or back ache$ or backache$ or (lumbar adj2 pain$) or (spin$ adj2 pain$)).ti,ab,kw.
2	(exp backache/th or exp backache/pc or exp backache/rh or exp *backache/) not exp backache/su
3	exp communication protocol/ or computer assisted therapy/ or e-mail/ or human computer interaction/ or information technology/ or interactive voice response system/ or internet/ or mass communication/ or medical informatics/ or medical technology/ or mobile application/ or mobile phone/ or social media/ or exp telecommunication/ or exp telehealth/ or telephone/ or text messaging/ or webcast/ or wireless communication/
4	computer storage device/ or computer terminal/ or microcomputer/ or minicomputer/ or attitude to computers/ or computer/ or computer system/ or medical information system/ or educational technology/ or audiovisual aid/ or exp multimedia/ or computer interface/ or hypermedia/ or electronic medical record/ or social networking/
5	(computer$ or microcomputer$ or pc or pcs or mac or macs or internet or www or web or website$ or webpage$ or local area network$ or software or (cellular phone$ or cellular telephone$ or mobile$ or cell phone$ or cell telephone$ or smartphone$ or smart-phone$ or smart-telephone$) or (handset$ or hand-set$ or wireless or wire-less or wifi or wi-fi or gps or global positioning system$ or bluetooth or text messag$ or texting or sms or short messag$ or multimedia messag$ or multi-media messag$ or mms or instant messag$ or social media$ or facebook or twitter or webcast$ or webinar$ or podcast$ or wiki or wikis or app or apps or android$ or blackberr$ or apple$ or ios or iphone$ or ipad$ or s40 or symbian$ or windows) or ((electronic$ or digital$ or device$) adj2 tablet$) or (video$ or dvd or dvds) or (youtube or you tube or vimeo) or (online or on line or interactive) or (chat room$ or chatroom$) or (blog$1 or web-log$1 or weblog$1) or (bulletin board$ or bulletinboard$ or messageboard$ or message board$) or (ehealth or e-health or mhealth or m-health) or (app or apps) or (pda or pdas or personal digital) or device-based or (email$ or e-mail$ or electronic mail$)).ti,ab,kw.
6	2 and 3
7	limit 6 to yr="2000 -Current"
8	1 and (3 or 4 or 5)
9	limit 8 to yr="2000 -Current"
10	9 not 7

Updated searches: 1) October 21 2016 (not shown), 2) December 18 2018 (below):

1	exp backache/ or (spinal pain$ or back pain$ or lumbago or back ache$ or backache$ or (lumbar adj2 pain$) or (spin$ adj2 pain$)).ti,ab,kw.
2	exp communication protocol/ or computer assisted therapy/ or e-mail/ or human computer interaction/ or information technology/ or interactive voice response system/ or internet/ or mass communication/ or medical informatics/ or medical technology/ or mobile application/ or mobile phone/ or social media/ or exp telecommunication/ or exp telehealth/ or telephone/ or text messaging/ or webcast/ or wireless communication/
3	computer storage device/ or computer terminal/ or microcomputer/ or minicomputer/ or attitude to computers/ or computer/ or computer system/ or medical information system/ or educational technology/ or audiovisual aid/ or exp multimedia/ or computer interface/ or hypermedia/ or electronic medical record/ or social networking/
4	(computer$ or microcomputer$ or pc or pcs or mac or macs or internet or www or web or website$ or webpage$ or local area network$ or software or (cellular phone$ or cellular telephone$ or mobile$ or cell phone$ or cell telephone$ or smartphone$ or smart-phone$ or smart-telephone$) or (handset$ or hand-set$ or wireless or wire-less or wifi or wi-fi or gps or global positioning system$ or bluetooth or text messag$ or texting or sms or short messag$ or multimedia messag$ or multi-media messag$ or mms or instant messag$ or social media$ or facebook or twitter or webcast$ or webinar$ or podcast$ or wiki or wikis or app or apps or android$ or blackberr$ or apple$ or ios or iphone$ or ipad$ or s40 or symbian$ or windows) or ((electronic$ or digital$ or device$) adj2 tablet$) or (video$ or dvd or dvds) or (youtube or you tube or vimeo) or (online or on line or interactive) or (chat room$ or chatroom$) or (blog$1 or web-log$1 or weblog$1) or (bulletin board$ or bulletinboard$ or messageboard$ or message board$) or (ehealth or e-health or mhealth or m-health) or (app or apps) or (pda or pdas or personal digital) or device-based or (email$ or e-mail$ or electronic mail$)).ti,ab,kw.
5	1 and (2 or 3 or 4)
6	limit 5 to yr="2000 -Current"

7	limit 5 to yr="2016 -Current"

CINAHL - search details

CINAHL (R) March 2016 through EBSCOhost

S6	S1 AND S4	
S5	S1 AND S4	
S4	S2 OR S3	
S3	TI (computer* OR microcomputer* OR pc OR pcs OR mac OR macs OR internet OR www OR web OR website* OR webpage* OR "local area network*" OR software OR "cellular phone*" OR "cellular telephone*" OR mobile* OR "cell phone"* OR "cell telephone*" OR smartphone* OR smart-phone* OR smart-telephone* OR handset* OR hand-set* OR wireless OR wire-less OR wifi OR wi-fi OR gps OR "global positioning system*" OR bluetooth OR "text messag*" OR texting OR sms OR "short messag*" OR "multimedia messag*" OR "multi-media messag*" OR mms OR "instant messag*" OR "social media*" OR facebook OR twitter OR webcast* OR webinar* OR podcast* OR wiki OR wikis OR app OR apps OR android* OR blackberr* OR apple* OR ios OR iphone* OR ipad* OR s40 OR symbian* OR windows OR ((electronic* OR digital* OR device*) W2 tablet*) OR video* OR dvd OR dvds OR youtube OR "you tube" OR vimeo OR online OR "on line" or interactive OR "chat room*" OR chatroom* OR blog OR blogs OR web-log OR web-logs OR weblog OR weblogs OR "bulletin board*" OR bulletinboard$ OR messageboard$ OR "message board*" OR ehealth OR e-health OR mhealth OR m-health OR app OR apps OR pda OR pdas OR "personal digital" OR "device-based" OR email* OR e-mail* OR "electronic mail*") OR AB (computer* OR microcomputer* OR pc OR pcs OR mac OR macs OR internet OR www OR web OR website* OR webpage* OR "local area network*" OR software OR "cellular phone*" OR "cellular telephone*" OR mobile* OR "cell phone"* OR "cell telephone*" OR smartphone* OR smart-phone* OR smart-telephone* OR handset* OR hand-set* OR wireless OR wire-less OR wifi OR wi-fi OR gps OR "global positioning system*" OR bluetooth OR "text messag*" OR texting OR sms OR "short messag*" OR "multimedia messag*" OR "multi-media messag*" OR mms OR "instant messag*" OR "social media*" OR facebook OR twitter OR webcast* OR webinar* OR podcast* OR wiki OR wikis OR app OR apps OR android* OR blackberr* OR apple* OR ios OR iphone* OR ipad* OR s40 OR symbian* OR windows OR ((electronic* OR digital* OR device*) W2 tablet*) OR video* OR dvd OR dvds OR youtube OR "you tube" OR vimeo OR online OR "on line" or interactive OR "chat room*" OR chatroom* OR blog OR blogs OR web-log OR web-logs OR weblog OR weblogs OR "bulletin board*" OR bulletinboard$ OR messageboard$ OR "message board*" OR ehealth OR e-health OR mhealth OR m-health OR app OR apps OR pda OR pdas OR "personal digital" OR "device-based" OR email* OR e-mail* OR "electronic mail*"))	
S2	(MH "Computer peripherals") OR (MH "Computer storage devices") OR (MH "Computer terminals") OR (MH	

	"Microcomputers") OR (MH "Computers, hand-held") OR (MH "Attitude to computers") OR (MH "Computer systems") OR (MH "Medical informatics") OR (MH "Educational technology") OR (MH "Audiovisuals") OR (MH "Audiorecording") OR (MH "Videorecording") OR (MH "Multimedia") OR (MH "Computer Environment") OR (MH "Computer Assisted Instruction") OR (MH "Hypermedia") OR (MH "Video games") OR (MH "Mobile applications") OR (MH "Patient record systems") OR (MH "Computerized patient record") OR (MH "") OR (MH "Computer communication networks+") OR (MH "Telecommunications") OR (MH "Electronic Bulletin Boards") OR (MH "Electronic Mail") OR (MH "Instant Messaging") OR (MH "Interactive Voice Response Systems") OR (MH "Text Messaging") OR (MH "Cellular Phone") OR (MH "Telephone") OR (MH "Internet+") OR (MH "Remote Consultation") OR (MH "Telemedicine") OR (MH "Telehealth") OR (MH "Telenursing") OR (MH "Smartphone") OR (MH "User-Computer Interface+")	
S1	(MH "Back Pain+") OR TI ("spinal pain* " OR "back pain*" OR lumbago OR "back acke*" OR backache OR (lumbar W2 pain*) OR (spin* W2 pain*)) OR AB ("spinal pain* " OR "back pain*" OR lumbago OR "back acke*" OR backache OR (lumbar W2 pain*) OR (spin* W2 pain*))	

Updated searches: 1) October 21 2016, 2) December 18 2018

Cochrane Library - search details (Through Wiley Online Library)

- Cochrane Database of Systematic Review (CDSR)
- Database of Reviews of Systematic Reviews (DARE, discontinued)
- Central Cochrane Register of Controlled Trials (CENTRAL)
- 'Method studies'
- 'Technology assessments'
- 'Economic evaluations'

#1	(spinal next pain* or back next pain* or lumbago or back next acke* or backache or (lumbar near/2 pain*) or (spin* near/2 pain*)):ti,ab,kw
#2	(computer* or microcomputer* or "pc" or "pcs" or "mac" or "macs" or "internet" or "www" or "web" or website* or webpage* or local next area next network* or "software" or cellular next phone* or cellular next telephone* or mobile* or cell next phone* or cell next telephone* or smartphone* or smart-phone* or smart-telephone* or handset* or hand-set* or "wireless" or "wire-less" or "wifi" or "wi-fi" or "gps" or global next positioning next system* or "bluetooth" or text next messag* or "texting" or "sms" or short next messag* or multimedia next messag* or multi-media next messag* or "mms" or instant next messag* or social next media* or "facebook" or "twitter" or webcast* or webinar* or podcast* or "wiki" or "wikis" or "app" or "apps" or android* or blackberr* or apple* or "ios" or iphone* or ipad* or "s40" or symbian* or "windows" or ((electronic* or digital* or device*) near/2 tablet*) or video* or "dvd" or "dvds" or "youtube" or "you tube" or "vimeo" or "online" or "on line" or "interactive" or chat next room* or chatroom* or "blog" or "blogs" or "web-log" or "web-logs" or "weblog" or "weblogs" or bulletin next board* or bulletinboard* or messageboard* or message next board* or "ehealth" or "e-health" or "mhealth" or "m-health" or "app" or "apps"

	or "pda" or "pdas" or "personal digital" or "device-based" or email* or e-mail* or electronic next mail*):ti,ab,kw
#3	#1 and #2

Updated searches: 1) October 21 2016 (not shown), 2) December 18 2018 (below):

#1	(spinal next pain* or back next pain* or lumbago or back next acke* or backache or (lumbar near/2 pain*) or (spin* near/2 pain*)):ti,ab,kw
#2	(computer* or microcomputer* or "pc" or "pcs" or "mac" or "macs" or "internet" or "www" or "web" or website* or webpage* or local next area next network* or "software" or cellular next phone* or cellular next telephone* or mobile* or cell next phone* or cell next telephone* or smartphone* or smart-phone* or smart-telephone* or handset* or hand-set* or "wireless" or "wire-less" or "wifi" or "wi-fi" or "gps" or global next positioning next system* or "bluetooth" or text next messag* or "texting" or "sms" or short next messag* or multimedia next messag* or multi-media next messag* or "mms" or instant next messag* or social next media* or "facebook" or "twitter" or webcast* or webinar* or podcast* or "wiki" or "wikis" or "app" or "apps" or android* or blackberr* or apple* or "ios" or iphone* or ipad* or "s40" or symbian* or "windows" or ((electronic* or digital* or device*) near/2 tablet*) or video* or "dvd" or "dvds" or "youtube" or "you tube" or "vimeo" or "online" or "on line" or "interactive" or chat next room* or chatroom* or "blog" or "blogs" or "web-log" or "web-logs" or "weblog" or "weblogs" or bulletin next board* or bulletinboard* or messageboard* or message next board* or "ehealth" or "e-health" or "mhealth" or "m-health" or "app" or "apps" or "pda" or "pdas" or "personal digital" or "device-based" or email* or e-mail* or electronic next mail*):ti,ab,kw
#3	#1 and #2
	With Publication Year from 2016 to 2018, with Cochrane Library publication date from Jan 2016 to Dec 2018, in Trials

#1	(spinal next pain* or back next pain* or lumbago or back next acke* or backache or (lumbar near/2 pain*) or (spin* near/2 pain*)):ti,ab,kw
#2	(computer* or microcomputer* or "pc" or "pcs" or "mac" or "macs" or "internet" or "www" or "web" or website* or webpage* or local next area next network* or "software" or cellular next phone* or cellular next telephone* or mobile* or cell next phone* or cell next telephone* or smartphone* or smart-phone* or smart-telephone* or handset* or hand-set* or "wireless" or "wire-less" or "wifi" or "wi-fi" or "gps" or global next positioning next system* or "bluetooth" or text next messag* or "texting" or "sms" or short next messag* or multimedia next messag* or multi-media next messag* or "mms" or instant next messag* or social next media* or "facebook" or "twitter" or webcast* or webinar* or podcast* or "wiki" or "wikis" or "app" or "apps" or android* or blackberr* or apple* or "ios" or iphone* or ipad* or "s40" or symbian* or "windows" or ((electronic* or digital* or device*) near/2 tablet*) or video* or "dvd" or "dvds" or "youtube" or "you tube" or "vimeo" or "online" or "on line" or "interactive" or chat next room* or chatroom* or "blog" or "blogs" or "web-log" or "web-logs" or "weblog" or "weblogs" or bulletin next board* or bulletinboard* or messageboard* or message next board* or "ehealth" or "e-health" or "mhealth" or "m-health" or "app" or "apps" or "pda" or "pdas" or "personal digital" or "device-based" or email* or e-mail* or electronic next mail*):ti,ab,kw
#3	#1 and #2

	With Cochrane Library publication date from Jan 2016 to Dec 2018, in Cochrane Reviews and Cochrane Protocols

PsycINFO - search details

Ovid PsycINFO (R) 1987 to March Week 4 2016

1	exp back pain/ or (spinal pain$ or back pain$ or lumbago or back ache$ or backache$ or (lumbar adj2 pain$) or (spin$ adj2 pain$)).ti,ab,id.
2	exp Human Computer Interaction/ or Computer Peripheral Devices/ or Computer Software/ or Human Machine Systems/ or exp Electronic Communication/ or exp Computers/ or exp Mobile Devices/ or exp Internet/ or exp Computer Applications/ or Computer Attitudes/ or Information Technology/ or exp AUDIOVISUAL INSTRUCTION/ or exp AUDIOVISUAL COMMUNICATIONS MEDIA/ or exp EDUCATIONAL AUDIOVISUAL AIDS/ or Telecommunications Media/ or Multimedia/ or exp Social media/ or exp Telephone systems/ or Telemedicine/ or exp Websites/ or (computer$ or microcomputer$ or pc or pcs or mac or macs or internet or www or web or website$ or webpage$ or local area network$ or software or cellular phone$ or cellular telephone$ or mobile$ or cell phone$ or cell telephone$ or smartphone$ or smart-phone$ or smart-telephone$ or handset$ or hand-set$ or wireless or wire-less or wifi or wi-fi or gps or global positioning system$ or bluetooth or text messag$ or texting or sms or short messag$ or multimedia messag$ or multi-media messag$ or mms or instant messag$ or social media$ or facebook or twitter or webcast$ or webinar$ or podcast$ or wiki or wikis or app or apps or android$ or blackberr$ or apple$ or ios or iphone$ or ipad$ or s40 or symbian$ or windows or ((electronic$ or digital$ or device$) adj2 tablet$) or video$ or dvd or dvds or youtube or you tube or vimeo or online or on line or interactive or chat room$ or chatroom$ or blog$1 or web-log$1 or weblog$1 or bulletin board$ or bulletinboard$ or messageboard$ or message board$ or ehealth or e-health or mhealth or m-health or app or apps or pda or pdas or personal digital or device-based or email$ or e-mail$ or electronic mail$).ti,ab,id.
3	1 and 2
4	limit 3 to yr="2000 -Current"

Updated searches: 1) October 21 2016 (not shown), 2) December 18 2018 (below):

1	exp back pain/ or (spinal pain$ or back pain$ or lumbago or back ache$ or backache$ or (lumbar adj2 pain$) or (spin$ adj2 pain$)).ti,ab,id.
2	exp Human Computer Interaction/ or Computer Peripheral Devices/ or Computer Software/ or Human Machine Systems/ or exp Electronic Communication/ or exp Computers/ or exp Mobile Devices/ or exp Internet/ or exp Computer Applications/ or Computer Attitudes/ or Information Technology/ or exp AUDIOVISUAL INSTRUCTION/ or exp AUDIOVISUAL COMMUNICATIONS MEDIA/ or exp EDUCATIONAL AUDIOVISUAL AIDS/ or Telecommunications Media/ or Multimedia/ or exp Social media/ or exp Telephone systems/ or Telemedicine/ or exp Websites/
3	(computer$ or microcomputer$ or pc or pcs or mac or macs or internet or www or web or website$ or webpage$ or local area network$ or software or cellular phone$ or cellular telephone$ or mobile$ or cell phone$ or cell telephone$ or smartphone$ or smart-phone$ or smart-telephone$ or handset$ or hand-set$ or wireless or wire-less or wifi or wi-fi or gps or global positioning system$ or bluetooth or text messag$ or texting or sms or short messag$ or multimedia messag$ or multi-media messag$ or mms or instant messag$ or social media$ or facebook or twitter or webcast$ or webinar$ or podcast$ or wiki or wikis or app or apps or android$ or blackberr$ or apple$ or ios or iphone$ or ipad$ or s40 or symbian$ or windows or ((electronic$ or digital$ or device$) adj2 tablet$) or video$ or dvd or dvds or youtube or you tube or vimeo or online or on line or interactive or chat room$ or chatroom$ or blog$1 or web-log$1 or weblog$1 or bulletin board$ or bulletinboard$ or messageboard$ or message board$ or ehealth or e-health or mhealth or m-health or app or apps or pda or pdas or personal digital or device-based or email$ or e-mail$ or electronic mail$).ti,ab,id.
4	1 and (2 or 3)
5	limit 4 to yr="2000 -Current"
6	5 and (20161* or 2017* or 2018* or 2019*).up.

DoPHER - search details

Database of Promoting Health Effectiveness Reviews
Focussed coverage of systematic and non-systematic reviews of effectiveness in health promotion and public health worldwide (3700).

Search date 11.04.2016

1	Freetext (Year): >1999
2	Freetext (All but Authors): “spinal pain” OR “back pain” OR “spinal pains” OR “back pains” OR lumbago OR “back ache” OR “back aches” OR “backache*”
3	1 AND 2

Updated searches: 1) October 21 2016, 2) December 18 2018

TROPHI - search details

Trials Register of Promoting Health Interventions
Focussed coverage of trials of interventions in health promotion and public health worldwide. It covers both randomised and non-randomised controlled trials and currently contains details of over 7,750 trials.

Search date 11.04.2016

5	Freetext (All but Authors): “spinal pain” OR “back pain” OR “spinal pains” OR “back pains” OR lumbago OR “back ache” OR “back aches” OR “backache*”
6	Freetext (Year): >1999
7	5 AND 6

Updated searches: 1) October 21 2016, 2) December 18 2018

Web of Science - search details
(Thomson Reuters)

Databases selected:

- Science Citation Index (SCI Expanded)
- Social Science Citation Index (SSCI)
- Conference Proceedings Citation Index – Science (CPCI-S)
- Conference Proceedings Citation Index – Social Science (SPCI-SSH)

Search date 6.4.2016

#3	#2 AND #1<br>*Indexes=SCI-EXPANDED, SSCI, CPCI-S, CPCI-SSH Timespan=2000-2016*

#2	**TOPIC:** (computer$ OR microcomputer* OR pc OR pcs OR mac OR macs OR internet OR www OR web OR website* OR webpage* OR "local area network*" OR software OR "cellular phone*" OR "cellular telephone*" OR mobile* OR "cell phone*" OR "cell telephone*" OR smartphone* OR smart-phone* OR smart-telephone* OR handset* OR hand-set* OR wireless OR wire-less OR wifi OR wi-fi OR gps OR "global positioning system*" OR bluetooth OR "text messag*" OR texting OR sms OR "short messag*" OR "multimedia messag*" OR "multi-media messag*" OR mms OR "instant messag*" OR "social media*" OR facebook OR twitter OR webcast* OR webinar* OR podcast* OR wiki OR wikis OR app OR apps OR android* OR blackberr* OR apple* OR ios OR iphone* OR ipad* OR s40 OR symbian* OR windows OR ((electronic* OR digital* OR device*) NEAR/2 tablet*) OR video* OR dvd OR dvds OR youtube OR "you tube" OR vimeo OR online OR "on line" OR interactive OR "chat room*" OR chatroom* OR blog OR blogs OR web-log OR web-logs OR weblog OR weblogs OR "bulletin board*" OR bulletinboard* OR messageboard* OR "message board*" OR ehealth OR e-health OR mhealth OR m-health OR pda OR pdas OR "personal digital" OR "device-based" OR email* OR e-mail* OR "electronic mail*")<br>*Indexes=SCI-EXPANDED, SSCI, CPCI-S, CPCI-SSH Timespan=2000-2016*
#1	**TOPIC:** ("spinal pain*" OR "back pain*" OR lumbago OR "back ache*" OR backache* OR lumbar NEAR/2 pain* OR spin* NEAR/2 pain*)<br>*Indexes=SCI-EXPANDED, SSCI, CPCI-S, CPCI-SSH Timespan=2000-2016*

Updated searches: 1) October 21 2016, 2) December 18 2018

OT Seeker - search details

Occupational therapy systematic evaluation of evidence.
http://www.otseeker.com/Search/BasicSearch.aspx

1	back pain AND (internet OR web)

Updated searches: 1) October 21 2016, 2) December 18 2018
