# Supplementary material for: Barriers and facilitators to patient uptake and utilisation of digital interventions for the self-management of low back pain: a systematic review of qualitative studies
Source: BMJ Open. 2020 Dec 12;10(12):e038800. doi: 10.1136/bmjopen-2020-038800 (PMC7735096; doi:10.1136/bmjopen-2020-038800)
Supplement: Supplementary data [file bmjopen-2020-038800supp002.pdf]

Supplementary File 2: Consensus summary of quality appraisal as per the 32-item Consolidated Criteria for Reporting Qualitative Research (COREQ) checklist (Booth et al., 2014; Tong et al., 2007) and comprehensiveness of reporting.

No	Item	Guide questions	de Jong et al., 2009	Caiata Zufferey & Schulz, 2009	Schulz et al., 2010	Nordin et al., 2017	Rabbi et al., 2018	Number of articles reporting each item (%)
**Domain 1: Research team and reflexivity**								
**Personal characteristics**								
**1**	Interviewer/facilitator	Which author/s conducted the interview or focus group?	N/R	N/R	N/R	Principal author	N/R	1 (20%)
**2**	Credentials	What were the researcher's credentials? E.g. PhD, MD	N/R	N/R	N/R	PhD	PhD, PhD and MD	2 (40%)
**3**	Occupation	What was their occupation at the time of the study?	N/R	N/R	N/R	N/R	N/R	0 (0%)
**4**	Gender	Was the researcher male or female?	N/R	N/R	N/R	Female	N/R	1 (20%)
**5**	Experience and training	What experience or training did the researcher have?	N/R	N/R	N/R	N/R	N/R	0 (0%)
**Relationship with participants**								
**6**	Relationship established	Was a relationship established prior to study commencement?	N/R	N/R	N/R	Participants had participated in the RCT, of which the qualitative study was a later part	N/R	1 (20%)
**7**	Participant knowledge of the interviewer	What did the participants know about the researcher? e.g.	N/R	N/R	N/R	N/R	N/R	0 (0%)

		personal goals, reasons for doing the research						
**8**	Interviewer characteristics	What characteristics were reported about the interviewer/facilitator? e.g. Bias, assumptions, reasons and interests in the research topic	N/R	N/R	N/R	N/R	N/R	0 (0%)
**Domain 2: Study design**								
**Theoretical framework**								
**9**	Methodological orientation and theory	What methodological orientation was stated to underpin the study? e.g. grounded theory, discourse analysis, ethnography, phenomenology, content analysis	Thematic analysis	Grounded theory	Inductive approach	Content Analysis	Thematic analysis	2 (40%)
**Participant selection**								
**10**	Sampling	How were participants selected? e.g. purposive, convenience, consecutive, snowball	Convenience	Purposive and convenience	Purposive and convenience	Consecutively	No selection, all participants of the DHI took part.	5 (100%)
**11**	Method of approach	How were participants approached? e.g. face-to-face, telephone, mail, email	N/R	Email	Email	First approach not clear, but once given oral consent contacted by telephone	Method of sending invitations not clear. If eligible face-to-face meeting	4 (80%)
**12**	Sample size	How many participants were in the study?	11 OPs who recruited; 8 OPs who did not recruit & 9 employees	18	18	19	10	5 (100%)

**13**	Non-participation	How many people refused to participate or dropped out? Reasons?	7 OPs who did not recruit; 15 employees. Reasons - no time, insufficient use of program, problems with recalling experiences	238 approached to participate; 32 responded; 14 of these did not participate – reasons not stated	N/R	3 – reasons not stated	None	4 (80%)
**Setting**								
**14**	Setting of data collection	Where was the data collected? e.g. home, clinic, workplace	Telephone interviews	Home or University	Home or University	Health Care Centres, County City Buildings, Participant's home	Web-based exit survey	5 (100%)
**15**	Presence of non-participants	Was anyone else present besides the participants and researchers?	N/R	N/R	N/R	N/R	N/R	0 (0%)
**16**	Description of sample	What are the important characteristics of the sample? e.g. demographic data, date	Not stated for OPs; Employees 67% male; 40-50 years; 75% LBP; white & blue-collar workers; varying educational levels; varying sickness absence levels due to LBP	9 females, 9 males; 28-72 years; chronic LBP for 1-30 years; mix of diagnoses including 8 with no clear diagnosis; all had at least secondary school education (5 had degree);	9 females, 9 males; 28-72 years; chronic LBP 1-30 years ; mixed diagnoses, varied level of education and frequency of website use	15 females, 4 males; mean age 45; MSK pain for average 7.5 years; most at least secondary education; majority working.	7 females, 3 males; 31-60 years; chronic LBP 5-33 years duration; mixed diagnoses.	5 (100%)

			7weeks-6 months	range of website use amongst participants				
**Data collection**								
**17**	Interview guide	Were questions, prompts, guides provided by the authors? Was it pilot tested?	Topic guides used. Pilot tested	No questions, prompts or guides provided; Piloting not reported	No questions, prompts or guides provided; Piloting not reported	No questions, prompts or guides provided; Piloting not reported	Open-ended question in web survey provided. Piloting not reported	2 (40%)
**18**	Repeat interviews	Were repeat interviews carried out? If yes, how many?	N/R	N/R	N/R	N/R	N/R	0 (0%)
**19**	Audio/visual recording	Did the research use audio or visual recording to collect the data?	Audio recorded	Not specifically stated "Recorded" and transcribed verbatim	Audio recorded	Audio recorded	No – used free text web survey	5 (100%)
**20**	Field notes	Were field notes made during and/or after the interview or focus group?	N/R	N/R	N/R	N/R	N/R	0 (0%)
**21**	Duration	What was the duration of the interviews or focus group?	Approx. 30 minutes	Approx. 45 minutes	Approx. 45 minutes	31 – 56 minutes. Mean 48 minutes	N/R	4 (80%)
**22**	Data saturation	Was data saturation discussed?	Yes	Yes	N/R	N/R	N/R	2 (40%)
**23**	Transcripts returned	Were transcripts returned to participants for comment and/or correction?	N/R	N/R	N/R	N/R	N/R	0 (0%)
**Domain 3: Analysis and findings**								

**Data analysis**								
**24**	Number of data coders	How many data coders coded the data?	N/R	N/R	N/R	4	N/R	1 (20%)
**25**	Description of coding tree	Did authors provide a description of the coding tree?	N/R	N/R	N/R	Yes	N/R	1 (20%)
**26**	Derivation of themes	Were themes identified in advance or derived from the data?	Derived from data	Derived from data	Essentially inductive	Derived from data	Derived from data	5 (100%)
**27**	Software	What software, if applicable, was used to manage the data?	Excel	ATLAS.ti	ATLAS.ti	Open Code	N/R	4 (80%)
**28**	Participant checking	Did participants provide feedback on the findings?	N/R	N/R	N/R	N/R	N/R	0 (0%)
**Reporting**								
**29**	Quotations presented	Were participant quotations presented to illustrate the themes / findings? Was each quotation identified? e.g. participant number	Few direct quotes; only identified as either OP or employee	Yes - identified by gender, age & occupation	Yes - identified by gender, age & occupation	Yes – identified by participant number and gender	Yes – identified by participant number	5 (100%)
**30**	Data and findings consistent	Was there consistency between the data presented and the findings?	A little unclear – little qualitative data presented	Yes	Yes	Yes	Yes	5 (100%)
**31**	Clarity of major themes	Were major themes clearly presented in the findings?	Yes	Yes	Yes	Yes	Yes	5 (100%)
**32**	Clarity of minor themes	Is there a description of diverse cases or discussion of minor themes?	Not clear	Range of themes presented but not clear what is major/minor	Range of themes presented but not clear what is major/minor	Yes	Yes	2 (40%)

**TOTAL, number (%)**	14 (44%)	15 (47%)	12 (38%)	21 (67%)	14 (44%)	
**DHI: digital health intervention; LBP: low back pain; N/R: not reported; OPs: occupational physicians;**						

References

Booth A, Hannes K, Harden A, Noyes J, Harris J, Tong A. COREQ (Consolidated Criteria for Reporting Qualitative Studies). In: Moher D, Altman DG, Schulz KF, Simera I, Wager E, editors. Guidelines for reporting health research: A user's manual. Oxford: Wiley; 2014. p. 214-26.

Caiata Zufferey M, Schulz PJ. Self-management of chronic low back pain: an exploration of the impact of a patient-centered website. Patient education and counseling. 2009;77(1):27-32.

de Jong T, Heinrich J, Blatter BM, Anema JR, van der Beek AJ. The feasibility of a web-based counselling program for occupational physicians and employees on sick leave due to back or neck pain. BMC medical informatics and decision making. 2009;9:46.

Nordin C, Michaelson P, Eriksson MK, Gard G. It's About Me: Patients' Experiences of Patient Participation in the Web Behavior Change Program for Activity in Combination With Multimodal Pain Rehabilitation. Journal of medical Internet research. 2017;19(1):e22-e.

Rabbi M, Aung MS, Gay G, Reid MC, Choudhury T. Feasibility and Acceptability of Mobile Phone-Based Auto-Personalized Physical Activity Recommendations for Chronic Pain Self-Management: Pilot Study on Adults. Journal of medical Internet research. 2018;20(10):e10147-e.

Schulz PJ, Rubinelli S, Zufferey MC, Hartung U. Coping with Chronic Lower Back Pain: Designing and Testing the Online Tool ONESELF. Journal of Computer-Mediated Communication. 2010;15(4):625-45.

Tong A, Sainsbury P, Craig J. Consolidated criteria for reporting qualitative research (COREQ): a 32-item checklist for interviews and focus groups. International journal for quality in health care : journal of the International Society for Quality in Health Care. 2007;19(6):349-57.
