# Supplementary material for: Barriers and facilitators to patient uptake and utilisation of digital interventions for the self-management of low back pain: a systematic review of qualitative studies
Source: BMJ Open. 2020 Dec 12;10(12):e038800. doi: 10.1136/bmjopen-2020-038800 (PMC7735096; doi:10.1136/bmjopen-2020-038800)
Supplement: Supplementary data [file bmjopen-2020-038800supp003.pdf]

Supplementary File 3: Taxonomy of barriers and facilitators with exemplar quotations

References:

[27] Schulz PJ, Rubinelli S, Zufferey MC, Hartung U. Coping with Chronic Lower Back Pain: Designing and Testing the Online Tool ONESELF. Journal of Computer-Mediated Communication. 2010;15(4):625-45.
[28] Caiata Zufferey M, Schulz PJ. Self-management of chronic low back pain: an exploration of the impact of a patient-centered website. Patient education and counseling. 2009;77(1):27-32.
[36] de Jong T, Heinrich J, Blatter BM, Anema JR, van der Beek AJ. The feasibility of a web-based counselling program for occupational physicians and employees on sick leave due to back or neck pain. BMC medical informatics and decision making. 2009;9:46.
[37] Rabbi M, Aung MS, Gay G, Reid MC, Choudhury T. Feasibility and Acceptability of Mobile Phone-Based Auto-Personalized Physical Activity Recommendations for Chronic Pain Self-Management: Pilot Study on Adults. Journal of medical Internet research. 2018;20(10):e10147-e.
[38] Nordin C, Michaelson P, Eriksson MK, Gard G. It's About Me: Patients' Experiences of Patient Participation in the Web Behavior Change Program for Activity in Combination With Multimodal Pain Rehabilitation. Journal of medical Internet research. 2017;19(1):e22-e.

**Barriers and facilitators for patient uptake and utilisation of digital self-management interventions for LBP**					
**Theme**	**Taxonomy**	**Barriers**	**Exemplar quotations**	**Facilitators**	**Exemplar quotations**
IT usability and accessibility	Functionality and usability	• Too much choice between functions<br>• Fixed advancement pace<br>• Issues logging into DHI<br>• *Low user-friendliness<br>• *Issues logging into DHI<br>• *Low level of functionality (e.g. registration, navigation, helpdesk)	• Though, the freedom of choice in the Web-BCPA entailed perceptions of restrained patient participation for some informants [38, p4]<br>• Finally, some OPs faced practical obstacles such as log-in problems […] [36, p5]<br>• Although OPs were generally positive about the user-friendliness and	• Flexible structure and navigation<br>• Conveniently arranged<br>• Variation of media types (text, audio and video)<br>• Reminders and notifications<br>• High user-friendliness<br>• *High user-friendliness	• *I liked this thing about the exercise video a lot because seeing it with the video gives you a lot more. They seem simple, but a lot of times when there are drawings I can't understand them easily, then I don't have the will anymore [28, p29]*<br>• It was enough to open the mailbox for reasons that could be independent of cLBP

			design of the program, some felt that further improving user-friendliness (functionality) might enhance its use. […] It should also be easier to register employees in the program [36, p6]<br>• Although, some informants perceived restrained patient participation by the fact that […] not being able to select a faster advancement in the program by themselves [36, p5]<br>• A small number of employees either had problems with 'logging in into the program' […] [36, p6]		to get a reminder of the website and the necessity of self-management […] *I usually went on the website when I read the newsletter. I read the letter and then I'm there, it's like a conditioned reflex [27, p641]*<br>• *It would be helpful to have reminders and suggestions pop up in the morning or at other chosen times. This could be optional and set by the user [37, p10]*<br>• These effects could be reached thanks to the specificities of the website, that is its […] multimediality (material was provided in written, audio and video form), usability (the website was easy to use […]) [28, p31]<br>• They [the users] were positive about the content, user-friendliness and web-based design. They

					said the information was easy to understand, to the point and conveniently arranged [36, p5]<br>• Finally, almost all OPs were positive about the user-friendliness and design of the program [36, p5]
	IT affinity	• Lack of affinity with computers<br>• *Lack of affinity with web-based programmes	• Some OPs had no affinity with the use of a web-based program in general and therefore preferred not to use this method [36, p5]<br>• A small number of employees […] had 'no affinity with computers' [36, p6]	• Enjoying working with a computer	• In addition, some informants stated that […] to enjoy working at the computer, facilitated patient participation in the rehabilitation [38, p6]
	Access and convenience	• Not able to choose starting time of DHI<br>• *No access to computer during consultation	• Although, some informants perceived restrained patient participation by the fact that they were not able to choose the starting time of the Web-BCPA course themselves (due to study protocol) […] [38, p5]<br>• Finally, some OPs faced practical	• Easily accessible with low effort<br>• Accessible at all hours and locations<br>• Accessible even during periods with severe pain symptoms<br>• Ability to take all the time needed	• Patient participation was emphasized by having access to the Web-BCPA on computer or tablet at all hours and locations [38, p5]<br>• The opportunities to work in the Web-BCPA at home were experienced to provide continuity in

			obstacles such as […] no access to a computer or the internet in their consulting rooms [36, p5]		the rehabilitation [38, p5]<br>• These effects could be reached thanks to the specificities of the website, that is its usability ([…] accessible from home without the necessity of intermediaries) [28, p31]<br>• […] informants described that the Web-BCPA provided opportunities to rehabilitation during periods with severe symptoms without having to be present at the health care center [38, p6]
Quality and quantity of content	Quality of content	• Contradicting content between DHI and HCP	• For some employees the exercises suggested by the program conflicted with the exercises given by the physiotherapist [36, p5]	• Trustworthy content and source<br>• Easily understandable content<br>• High quality of content<br>• Steady content<br>• *Appropriate content	• *Knowing there is a serious website where there are contributions, it strengthens you a bit [28, p29]*<br>• Some users felt reassured because they had a trustworthy place where they could address concerns [27, p641]

					• These effects could be reached thanks to the specificities of the website, that is its […] trustworthiness (material was controlled by health professionals according to the criteria of Evidence Based Medicine) [28, p31]<br>• They [the users] were positive about the content, user-friendliness and web-based design. They said the information was easy to understand, to the point and conveniently arranged [36, p5]<br>• More than half of the OPs were positive about the content (e.g. information, exercises, instructions) [36, p5]<br>• […] the stability of the material helped them to construct their personal frame of reference about the nature and the

					course of their cLBP [27, p640]
	Amount of content	• Too much content to choose from<br>• Too much information to fully comprehend	• According to some people, Oneself provided too much information, risking creating confusion about the comprehension of the health problem and the identification of the best way to treat it: *There is a lot of information, probably almost too much, don't you think? [28, p29]*<br>• […] having difficulties to choose from its content, were experienced to restrain patient participation [38, p8]	• A lot of content to choose from	• The richness and trustworthiness of the information […] helped them to construct their personal frame of reference about the nature and the course of their cLBP [28, p28]<br>• First, the quality and continual update of the website encouraged people to visit Oneself again and to continue thinking about self-management [28, p29]
Tailoring and personalisation	Tailoring, specificity and personalisation	• Content not tailored to individual needs and/or pain severity<br>• Content perceived not new or relevant	• […] because some of the advice and exercises were not specific enough, they did not apply to the employee's situation [36, p5]	• Content accounting for individual needs and/or pain severity<br>• Self-identification in content<br>• Opportunity to influence treatment	• *..it was obvious that it (the rehabilitation) was about me, it wasn't about just anyone.. it was about my problems, my strengths and how I felt.. they (the HCPs*

			• Some persons perceived information not new nor relevant. In this case, the use of Oneself lead to feelings of hopelessness: two participants had the impression that again there was no solution for their problem [28, p29]<br>• *The exercises that you have on the website are good, but I can't do any of them, no. I tried to do them a bit on the bed, but with my arm that doesn't work, my knees that don't work… There are lots, indeed I had written down those that I could do, but then many times your will is missing (…) Then you get sick of it. I know, that it's for my own good that I should exercise, but after a while I… Then you don't have grand results, and so even*		*started from a blank page, I was not fitted into an average template of how it ought to be.. it (the rehabilitation) started with my point of view [38, p4-5]*<br>• *I really liked the personalization. I thought it was a nice touch. Suggestions were more specific and tailored, which for me made them more relevant and likely for me to use them [37, p9]*<br>• *Previously I had read about CBT (Cognitive Behavioral Therapy), but I had never thought of it as a help for my condition.. I want to compare this rehabilitation with a smorgasbord from which is it easy to taste [38, p5]*<br>• *It gives you descriptions and you say: this stuff here.. I see it, I see it! I recognise myself in it,*

			for my back sometimes I go through periods, moments where I'm, let's say, very diligent, and then sometimes… (…) Yes, it's interesting. But there are always the same things that you then don't do [28, p29]		*I recognise myself here [27, p640]*<br>• Informants experienced that being able to identify themselves with the content in the rehabilitation and finding it trustworthy were important to patient participation and being confirmed [38, p5]<br>• They [informants] described that they were confirmed when they could identify their illness experience and life situation, as well as their own thoughts and cognitions about their pain condition, in the texts and the assignments of the Web-BCPA [38, p7]
Motivation and support	Personal attributes and resources	• Adhering to biomedical model of LBP<br>• Seeing LBP as a marginal problem<br>• Preferring other treatment regimens, e.g. with human contact	• *I went to a doctor who told me 'there is nothing to do, just resign yourself to it'. So this unleashed really the research to find something. But after eight years I didn't find the magic*	• High level of awareness and self-management of LBP<br>• Aware that LBP would not be fixed with a medical solution and ready to accept active role	• In addition, some informants stated that their work experience, such as having a solution-focused work […] facilitated patient participation in the rehabilitation [38, p6]

		• Lack of knowledge about LBP and treatments<br>• Physical health (e.g. pain, fatigue)<br>• Psychological symptoms	*cure, unfortunately. And one continuously hears 'they are doing new research!' But hopefully they will arrive in time in order to do something. (…) I'm always in search of the super novelty, the one that heals [28, p30]*<br>• One employee mentioned that the back or neck pain they were suffering from may have prevented them from sitting at a computer [36, p6]<br>• Pain, fatigue and other psychological symptoms were perceived to limit patient participation [38, p6]<br>• Three users could be defined as passive self-managers: They adhered to a traditional biomedical model of cLBP and were convinced that the solution of their problem had to be	• Emotional and cognitive resources, e.g. motivation, interest, commitment and self-confidence in self-management of LBP<br>• Enjoy solution focused work	• *I already know which road I have to follow in detail. I need details or confirmation on these details [28, p29]*<br>• They described emotions and cognitions that affected patient participation. Having motivation, interest, commitment, and self-confidence were perceived to favor patient participation [38, p6]<br>• Most of the users could be defined as experienced self-managers, in the sense that they had a rather high level of awareness and self-management of cLBP even before knowing Oneself. These people […] had a rather clear idea about their diagnosis, and knew that they had to play an active role in dealing with their health problem [27, p635]

			found by health professionals. These people went to Oneself to find a definitive medical solution for their cLBP [27, p635]<br>• Three users could be defined as latent self-managers. […] For all of them, cLBP was at the moment a marginal problem, in the sense that it was intermittent and light. These users did not really need to engage in a long-term process of self-management: When pain appeared, they usually dealt with it through some easy coping strategies, such as taking painkillers, going to the chiropractic, etc [27, p636]		• Two users could be defined as novices in terms of self-management. These participants were aware that a medical solution to cLBP did not exist and were ready to accept that they had to become actively involved in their cLBP care. However, they did not know how to do it [27, p635]
	Support to use DHI	• HCP unsupportive of use of DHI<br>• No support from authorities	• *I planned to complete the program (the Web-BCPA).. I am not sure how much I had left.. probably the last module.. but I was denied sick-leave*	• HCP supportive of use of DHI<br>• Support from family<br>• Support from authorities<br>• Support from other suffers (e.g. successful testimonials)	• *It's nice knowing that there is someone else [28, p29]*<br>• *When you are going through a moment when you have backache and you*

			compensation by the Social Insurance Agency and had to put in a lot of energy to explain my situation and meet with the psychosocial counsellor.. I did not have the strength to do anything else.. I have used so much energy to fight for my cause [12052, 6]<br>• One employee said, *I expected more commitment from my OP.* This did not encourage employees to use the program [2120, 5]		*read a testimony which says 'yes, there is someone who was able to do it', it gives you hope [28, p29]*<br>• Support, trust and respect from a family member, employer, the Swedish Social Insurance Agency (SSIA) or the Employment Service were experienced to facilitate patient participation in the rehabilitation [38, p6]
	Features of DHI	• DHI not guiding or supporting participants enough (e.g. to plan for execution of physical activity recommendation from DHI)	• *I received the suggestion to ride a bike, but that's currently simply not possible, logistically [37, p10]*<br>• *If it could ask me to rank the things I enjoy doing and then download weather data for the following days. This could suggest times when I have performed these tasks in the*	• Interaction/interactivity<br>• Information about self-management of LBP<br>• Goal-setting<br>• Action-planning<br>• Follow-up and evaluation<br>• Adjusting treatment related to setbacks and progress<br>• Monitoring own progress in graphs<br>• Variation of content<br>• Update of content	• To acquire knowledge and insights were thought of as patient participation, and included self-reflection, self-identification, and feedback [38, p5]<br>• […] with opportunities to influence and a variety of treatments to choose according to one's own needs and priorities [38, p5]

			past and also match it with weather predictions. "You played tennis for last Tuesday in the afternoon for 90 minutes. How about from 2 to 4 today when the weather will be clear and 85". [37, p10]		• To adjust a goal or treatment planning in relation to progress or setback was described as patient participation: *I feel it is important to set goals and to follow-up those goals.. and to why a goal is reached and why another is not.. this made me aware of that I needed other tools (in the rehabilitation)* [38, p6]<br>• Patient participation was reported when informants monitored results shown by the interactive graphs in the Web-BCPA: *.. days when I had a lot of pain I used to remain sedentary, and as soon as I had a better day I was eager to do all kinds of activities that day.. before I started with the assignment activity planning (in the Web-BCPA) I was*

					not aware of how my behaviour related to the days with pain, but by monitoring this over time I started to plan my daily activities in a more balanced way [38, p6]<br>• These effects could be reached thanks to the specificities of the website, that is its interactivity (people could ask specific questions to health professionals who were available daily for responding), […] dynamism (the website was updated weekly) […] [28, p31]<br>• The informants' experienced patient participation when they analyzed their situation taken into account their resources and restrictions, set goals for behavior change, and planned treatments and activities. Also, patient participation was stated when

					treatments, self-care, and planning were followed-up and evaluated [38, p6]
	HCP factors for support of patients	• *Time restrictions of consultations<br>• *Difficulty keeping DHI in mind during consultations<br>• *Difficulty providing patients with accurate information about DHI<br>• *Perceiving no benefit of DHI compared to usual treatment<br>• *Preferring other treatment regimens, e.g. with human contact	• *It takes time to get used to the recruitment process and to using the program [36, p5]*<br>• A second important barrier for OPs was the limited time available for introducing employees to the program and working with it as well. […] *We lack the time to do this kind of projects [36, p5]*<br>• One OP stated that he did not use the program because he did not believe in 'computer-based treatment' of physical pain. He explained, *The ability to touch people is an essential element in the treatment of people with back or neck pain. [36, p5]*	• *DHI a good medium for counselling employees	• About half of the OPs indicated that a website is a good medium for counselling of employees with back or neck pain [36, p5]

			• One OP stated that he was quite capable of managing the RTW process himself and did not need a program for additional support. Many preferred the more familiar therapies (e.g. physiotherapy) […]. They preferred having personal contact with employees [36, p5]		

*= HCP perspective; **IT**: information technology; **HCP**: healthcare professional; **DHI**: Digital health intervention
