# Supplementary material for: Barriers and facilitators to patient uptake and utilisation of digital interventions for the self-management of low back pain: a systematic review of qualitative studies
Source: BMJ Open. 2020 Dec 12;10(12):e038800. doi: 10.1136/bmjopen-2020-038800 (PMC7735096; doi:10.1136/bmjopen-2020-038800)
Supplement: Author's manuscript [file bmjopen-2020-038800.draft_revisions.pdf]

BMJ Open

BMJ Open is committed to open peer review. As part of this commitment we make the peer review history of every article we publish publicly available.

When an article is published we post the peer reviewers' comments and the authors' responses online. We also post the versions of the paper that were used during peer review. These are the versions that the peer review comments apply to.

The versions of the paper that follow are the versions that were submitted during the peer review process. They are not the versions of record or the final published versions. They should not be cited or distributed as the published version of this manuscript.

BMJ Open is an open access journal and the full, final, typeset and author-corrected version of record of the manuscript is available on our site with no access controls, subscription charges or pay-per-view fees (http://bmjopen.bmj.com).

If you have any questions on BMJ Open's open peer review process please email info.bmjopen@bmj.com

BMJ Open

# Barriers and facilitators to patient uptake and utilisation of digital interventions for the self-management of low back pain: a systematic review of qualitative studies



| | |
|---|---|
| Journal: | *BMJ Open* |
| Manuscript ID | bmjopen-2020-038800 |
| Article Type: | Original research |
| Date Submitted by the Author: | 26-Mar-2020 |



| | |
|---|---|
| Complete List of Authors: | Svendsen, Malene; National Research Centre for the Working Environment, Musculoskeletal disorders and physical work demands; University of Southern Denmark Department of Sports Science and Clinical Biomechanics, Physical activity and health in work life<br>Wood, Karen; University of Glasgow<br>Kyle, John ; University of Glasgow, Institute of Health and Wellbeing<br>Cooper, K; Robert Gordon University, School of Health Sciences<br>Rasmussen, Charlotte Diana Nørregaard; The National Research Centre for The Working Environment,<br>Sandal, Louise; University of Southern Denmark, Department of Sport Science and Clinical Biomechanics<br>Stochkendahl, Mette; Nordic Institute of Chiropractic and Clinical Biomechanics, ; University of Southern Denmark, Institute of Sports Science and Clinical Biomechanics<br>Mair, Frances; University of Glasgow, General Practice and Primary Care<br>Nicholl, Barbara; University of Glasgow, General Practice and Primary Care |



| | |
|---|---|
| Keywords: | Back pain < ORTHOPAEDIC & TRAUMA SURGERY, Health informatics < BIOTECHNOLOGY & BIOINFORMATICS, PAIN MANAGEMENT, QUALITATIVE RESEARCH |


SCHOLARONE™
Manuscripts

*I, the Submitting Author has the right to grant and does grant on behalf of all authors of the Work (as defined in the below author licence), an exclusive licence and/or a non-exclusive licence for contributions from authors who are: i) UK Crown employees; ii) where BMJ has agreed a CC-BY licence shall apply, and/or iii) in accordance with the terms applicable for US Federal Government officers or employees acting as part of their official duties; on a worldwide, perpetual, irrevocable, royalty-free basis to BMJ Publishing Group Ltd ("BMJ") its licensees and where the relevant Journal is co-owned by BMJ to the co-owners of the Journal, to publish the Work in this journal and any other BMJ products and to exploit all rights, as set out in our licence.*

*The Submitting Author accepts and understands that any supply made under these terms is made by BMJ to the Submitting Author unless you are acting as an employee on behalf of your employer or a postgraduate student of an affiliated institution which is paying any applicable article publishing charge ("APC") for Open Access articles. Where the Submitting Author wishes to make the Work available on an Open Access basis (and intends to pay the relevant APC), the terms of reuse of such Open Access shall be governed by a Creative Commons licence – details of these licences and which Creative Commons licence will apply to this Work are set out in our licence referred to above.*

*Other than as permitted in any relevant BMJ Author's Self Archiving Policies, I confirm this Work has not been accepted for publication elsewhere, is not being considered for publication elsewhere and does not duplicate material already published. I confirm all authors consent to publication of this Work and authorise the granting of this licence.*

For peer review only

# Barriers and facilitators to patient uptake and utilisation of digital interventions for the self-management of low back pain: a systematic review of qualitative studies

Malene Jagd Svendsen[2,5], Karen Wood[1], John Kyle[1], Kay Cooper[3], Charlotte Diana Nørregaard Rasmussen[2], Louise Fleng Sandal[5], Mette Jensen Stochkendahl[5,6], Frances S Mair[1], Barbara I Nicholl[1]

**Affiliations**

1. Institute of Health & Wellbeing, University of Glasgow, Glasgow, UK
2. National Research Centre for the Working Environment, Copenhagen Ø, Denmark
3. School of Health Sciences, Robert Gordon University, Aberdeen, UK
4. Department of Public Health and Nursing, Faculty of Medicine, NTNU, Norwegian University of Science and Technology, Trondheim, Norway
5. Department of Sports Science and Clinical Biomechanics, University of Southern Denmark, Odense, Denmark
6. Nordic Institute of Chiropractic and Clinical Biomechanics, Odense, Denmark

**Email Addresses**

Malene Jagd Svendsen mas@nrcwe.dk

Karen Wood Karen.Wood@glasgow.ac.uk

John Kyle John.Kyle@nhs.net

Kay Cooper k.cooper@rgu.ac.uk

Charlotte Diana Nørregaard Rasmussen cnr@nrcwe.dk

Louise Fleng Sandal lsandal@health.sdu.dk

Mette Jensen Stochkendahl m.jensen@nikkb.dk



For peer review only - http://bmjopen.bmj.com/site/about/guidelines.xhtml

For peer review only

Frances S Mair Frances.Mair@glasgow.ac.uk

Barbara Nicholl Barbara.Nicholl@glasgow.ac.uk

**Corresponding Author:**

Malene Jagd Svendsen

National Research Centre for the Working Environment, Lersø Parkallé 105, 2100 Copenhagen, Denmark

Tel: +45 39165329 Email: mas@nrcwe.dk

Word count: 5851 words

**Abstract**

**Objective:** Low back pain (LBP) is a leading contributor to disability globally. Self-management is a core component of LBP management; however, this often proves challenging for individuals. We aimed to synthesise and critically appraise published qualitative literature concerning digital health interventions to support self-management of LBP to: 1) determine what engagement strategies have been utilised, 2) identify barriers and facilitators affecting patient uptake and utilisation and, 3) develop a preliminary conceptual model of barriers and facilitators to uptake and utilisation.

**Setting:** Community and primary care contexts in Switzerland, the Netherlands, Sweden and the US.

**Participants:** Fifty-six adults with non-specific low back pain and nineteen health care professionals providing care for such patients from four qualitative studies.

**Primary and secondary outcome measures:** Barriers and facilitators for uptake and utilisation of digital health interventions for self-management of low back pain; strategies for recruitment and enrolment into digital health interventions for self-management of low back pain.

**Results:** Systematic search of eight bibliographic databases conducted for publications between 2000 and December 2018 using the concepts: 1) back pain, 2) digital intervention, and 3) self-management resulted



For peer review only - http://bmjopen.bmj.com/site/about/guidelines.xhtml

in five full text articles from four studies included in the review. Four major themes relating to barriers and facilitators were identified: IT usability and accessibility; quality and quantity of content; tailoring and personalisation; motivation and support.

**Conclusions**: We highlight key barriers and facilitators that should be considered when designing digital interventions to support self-management of LBP. Many of our findings are in keeping with reviews of digital interventions for self-management of other long-term conditions, implying these findings may not be condition specific. Further research is required to identify which approaches are likely to impact on user engagement and self-management outcomes.

**PROSPERO Registration number** CRD42016051182

**Systematic review registration**: A protocol for this systematic review was registered with https://www.crd.york.ac.uk/PROSPERO/ (CRD42016051182) on November 10th, 2016. https://www.crd.york.ac.uk/PROSPERO/display_record.php?ID=CRD42016051182

**Article summary**

Strengths and limitations of this study:

- This systematic review of qualitative studies explored barriers and facilitators for the uptake and utilisation of digital health interventions for low back pain (LBP) to inform the future design and implementation processes of such interventions.
- Searches in multiple databases and independent data extraction, quality appraisal and detailed data analysis are strengths of our review. However, our search strategy revealed that literature in the field of digital self-management for LBP is sparse as only a small number of eligible studies were identified.

For peer review only

- Given the limited literature, it is possible that not all important barriers and facilitators for uptake and utilisation have been identified and thus our conceptual model must be considered preliminary.

**Keywords:** Low back pain; eHealth; self-management; qualitative, engagement; utilisation; NPT

## Background

Low back pain (LBP) affects approximately 12% of the general population at any point in time (1); it is the leading contributor to disability worldwide (2) and is associated with significant personal (3) and societal costs (4, 5). Self-management approaches are consistently recommended in clinical guidelines as a core component of LBP management (6, 7); however, adherence to self-management strategies has proved challenging, especially without support and reinforcement (8, 9). Digital health interventions (DHIs) offer a potential method of supporting self-management (10-12), and particularly the possibility of tailoring self-management advice, may hold significant potential for people with LBP (13). DHIs or "digital therapeutics" are becoming increasingly popular and, as technological innovations increase, it is expected that this trend will continue (14, 15). Until now, two systematic reviews have examined the use of DHIs to support the self-management of LBP. The first, by Garg et al., aimed to determine which web-based interventions are of benefit to patients (16). They identified nine randomised controlled trials (RCTs), including a total of 1796 participants. Four trials studied online cognitive behavioural therapy (CBT) with the remaining five trials studying web-based interventions with interactive features such as a virtual gym, testimonials, or moderated discussion groups. Garg et al. reported that online CBT approaches appeared to reduce catastrophizing and improve patient attitudes, whilst studies of web-based interventions with interactive features used a variety of diverse outcome measures yielding inconclusive results; thus, making it difficult to draw firm conclusions regarding long-term impact for people with LBP.

For peer review only



For peer review only - http://bmjopen.bmj.com/site/about/guidelines.xhtml

The second review, by Nicholl et al., aimed to appraise the evidence concerning the use of interactive DHIs to support patient self-management of LBP with a focus on the outcome measures used and reported effects (17). They identified six completed RCTs studying digital tools for the self-management of LBP including a total of 2706 participants. Nicholl et al. reported that only one of the six completed RCTs observed a between-group difference in favour of the digital intervention, with none of the studies demonstrating any evidence of harm. The authors noted that there was considerable variation in the nature and delivery of the interventions and inconsistency in the choice of outcomes and concluded that the current evidence base for DHIs to support the self-management of LBP remained weak.

Yet, hundreds of smartphone applications (apps) related to LBP are currently available on the app market, most developed with very little scientific rigour (18). In order to facilitate the development of appropriate and effective self-management DHIs for those with LBP, it is important to have an understanding of the factors that help or hinder user engagement and adherence. Across different conditions, multiple barriers and facilitators to engaging with DHIs have previously been identified, including issues such as motivation and support, digital literacy, privacy, usability, quality and tailoring (17, 19). However, given the diverse range of DHIs available, it can be difficult to apply these findings to a specific patient population or piece of technology. Understanding the experience of users of DHIs designed specifically to assist self-management of LBP would help determine how to optimise DHIs for this group of users.

The purpose of this systematic review was therefore to synthesise and critically appraise the published qualitative literature concerning the use of DHIs to promote self-management of LBP in order to address the following two research questions:

1. What engagement strategies at the time of enrolment have been utilised in DHIs aimed at supporting patient self-management of LBP?

2. What are the barriers and facilitators to patient uptake and utilisation of digital interventions to support self-management of LBP?

For peer review only

121 The final objective of the systematic review was to develop a preliminary conceptual model of barriers and facilitators to uptake and utilisation of digital interventions to support self-management of LBP.

## Methods

Protocol and registration

This review was registered in the International Prospective Register of Systematic Reviews, PROSPERO, registration no. CRD42016051182 (20) and reporting is consistent with the Preferred Reporting Items for Systematic Reviews and Meta-Analyses (PRISMA) statement (21).

Eligibility criteria

Qualitative studies that examine engagement, barriers and/or facilitators to patient uptake and utilisation of digital interventions for the self-management of LBP were included; inclusion and exclusion criteria are outlined in Table 1.

**Table 1:** Inclusion and exclusion criteria.

| **Inclusion criteria** | |
|---|---|
| Study type | • Published in peer-reviewed journals between January 1st 2000 and December 18th 2018.<br>• Original qualitative studies, studies involving secondary qualitative analysis of qualitative data and qualitative studies that were part of a mixed methods study (provided the qualitative methodology was described).<br>• Qualitative data collected via questionnaires or other methods not involving direct contact or observation of participants were eligible for inclusion provided questions were answered using free text and analysed using a qualitative approach.<br>• Qualitative data describing barriers and/or facilitators to the uptake or utilisation of digital interventions or containing a description of an engagement strategy (i.e. any method used to get people to enrol into the study) from a patient or HCP's perspective. |
| Language | • Published in English, Danish or Norwegian. |
| Participants | • Adults >18 years with non-specific LBP or HCPs providing care for such patients. |
| Setting | • Community, primary or secondary care and other specialist contexts including those that recruit via media. |



For peer review only - http://bmjopen.bmj.com/site/about/guidelines.xhtml

| | | |
|---|---|---|
| Digital intervention | | • Any intervention accessed through a computer, mobile phone, or other handheld device, involving a web-based programme, desktop programme or application that provided self-management content (consistent with previous reviews (17, 22)).<br>• Interventions must involve an element of interaction between the user and the digital interface; this was defined as information being taken from users which then provided some form of automated feedback and/or advice in response.<br>• Interventions that included face-to-face contact were only included if this interaction was in addition to an automated, interactive digital component without direct HCP mediation. |
| **Exclusion criteria** | | |
| Study type | | • Descriptive case studies, lexical studies that analyse natural language data presented as qualitative results, literature or systematic reviews, meta-analyses, studies without a sampling procedure (i.e. no clear description of recruitment strategy) and commentary articles written to convey opinion or stimulate discussion with no research component. |

**HCP**: Healthcare professional

Information sources and search strategy

A systematic search of bibliographic databases (MEDLINE, Embase, CINAHL, PsycINFO, Cochrane Library, DoPHER, TROPHI, Web of Science and OT Seeker) was conducted after the search strategy had been developed in collaboration with an information specialist at the Norwegian University of Science and Technology (NTNU) and experienced researchers in the field of LBP and digital health interventions. The search strategy has previously been described and published by Nicholl et al. (17). Reference and citation tracking was utilised to identify relevant references. All databases were searched for publications using three groups of concepts: (1) low back pain, (2) digital intervention, and (3) self-management. The search was conducted in three waves using the same search strategy: the first for publications added between January 2000 and March 2016, then a subsequent updated search for articles added between March 2016 and October 2016, and lastly, articles added between October 2016 and December 2018. Limitation of year of publication from 2000 onwards was chosen as our review was aimed at understanding current experiences of digital health technologies, justified by emerging Internet access around the millennium and the developing field of digital health interventions that followed, and further supported by other systematic

For peer review only



For peer review only - http://bmjopen.bmj.com/site/about/guidelines.xhtml

reviews of digital interventions (16, 23, 24). The complete search strategy, including specifications on the use of title, keywords or abstract screening is documented in Supplementary File 1.

## Study selection

All identified citations were uploaded to Distiller SR software (Evidence Partners, Ottawa, Canada) and duplicates were removed. Title and abstract screening was performed by two of four independent reviewers (JK, MaS, KC, KW) using Distiller SR. Any disagreement between the two reviewers resulted in inclusion of the citation to the next screening level. Full-text screening was also performed by two of four independent reviewers (JK, MaS, KC, KW) with any discrepancies at this level being resolved through discussion mediated by a third party (BN, CR, MeS, KC).

## Data extraction

A comprehensive, standardised data extraction template designed specifically for this review in Distiller SR was utilised by two of four independent researchers (JK, MaS, BN, KW). Where available, information collected included the study title, authors, citation, year of study and publication, country, inclusion/exclusion criteria, aim, setting, characteristics of the digital intervention, recruitment methods, method of qualitative data collection and analysis, participant numbers and characteristics, any engagement strategies, barriers or facilitators identified either by the authors or in participant quotes, conclusions, limitations, funding sources and any potential conflicts of interest declared.

## Quality appraisal

The complete 32-item Consolidated Criteria for Reporting Qualitative Research (COREQ) checklist (25, 26) was used to assess the methodological quality of the articles progressing to data extraction. Two of three reviewers (BN, KC, KW) independently identified whether each of the 32-items were reported or not, and descriptive information was provided where possible. Disagreements between reviewers were resolved



For peer review only - http://bmjopen.bmj.com/site/about/guidelines.xhtml

through discussion. A-priori cut-off points were not determined as studies were not excluded on the basis of methodological quality. Two of the included articles report on the qualitative evaluation of the same intervention but were treated as separate articles for quality appraisal (27, 28).

Data synthesis and analysis

Information on the engagement strategies, defined as methods used to recruit and initially motivate participants to enrol in the DHI study, in each study was described narratively as this was only provided descriptively in the included studies. Our data synthesis of barriers and facilitators to patient uptake and utilisation of the DHI for LBP involved a thematic approach (29). Data on barriers and facilitators were extracted from results and discussion sections of the included studies. Each item of extracted data was initially coded by one reviewer (MaS). When new codes appeared during the analysis of a particular article, the articles that had previously been examined were re-read and re-coded if appropriate. This continuous adjustment was carried out in cooperation with a second reviewer (KW). Emergence and mapping of codes were discussed at coding clinics to ensure construction of themes that were internally homogenous and externally heterogeneous (i.e. no data excluded due to lack of a suitable theme, and no data falling between two themes or fitting into more than one theme) (30, 31) (MaS, KW, FM, BN). This resulted in a coding taxonomy for mapping identified codes as barriers or facilitators for each theme.

A preliminary conceptual model of barriers and facilitators to uptake and utilisation of DHIs to support self-management of LBP was developed by mapping the identified themes to the four constructs of Normalization Process Theory (NPT). NPT is a sociological theory developed to explore the process of implementing a new complex intervention, in this case it can help explain how people individually and collectively embed DHIs into everyday practice (32, 33). The identified themes were mapped to NPT constructs by four reviewers (KW, FM, BN, JK) using the coding framework presented in Table 2. This approach has been successfully applied in other systematic reviews of DHIs for chronic disease self-management issues (19, 34, 35) and provides a solid conceptual basis from which to understand barriers



For peer review only - http://bmjopen.bmj.com/site/about/guidelines.xhtml

and facilitators to patient and HCP uptake and utilisation of DHIs. Any themes that could not be coded to the NPT constructs were carefully noted to ensure that themes outside the scope of NPT would still be captured to assure appropriateness of the model.

**Table 2:** Core constructs of Normalization Process Theory (NPT) (32, 33) and related coding framework for development of preliminary conceptual model of barriers and facilitators to uptake and utilisations of digital interventions to support self-management of LBP.

| **Core constructs of NPT** | **Coding framework** |
|---|---|
| Coherence (Sense Making Work; enrolling with the DHI): development of an individual and collective understanding of the new intervention when faced with operationalizing it. | • How people understand and view the benefits versus disbenefits of DHIs and decide whether it is appropriate for them to use.<br>• Motivation and willingness to commit to self-management activities. |
| Cognitive Participation (Engagement Work; engaging with the DHI): relational work to build and sustain engagement with a new intervention. | • Willingness to "buy into" the DHI and whether it is a legitimate means to promote self-management of LBP.<br>• Issues relating to the support provided to use the DHI and level of engagement of HCPs involved with the DHI. |
| Collective Action (Operationalisation Work; utilising the DHI): investment of effort and resources to enact the new intervention. | • Ease of use, accessibility and appropriateness of the DHI.<br>• Resources, training, workload and technical support.<br>• Perceived quality and trustworthiness of DHI content and function. |
| Reflexive Monitoring (Appraisal Work; maintaining engagement with DHI): evaluation of the impact of the new intervention on individuals and groups along with any reconfigurations suggested. | • How people judge the new DHI and the self-monitoring work that accompanied uptake of the DHI.<br>• Ability to tailor to an individual's needs. |
| **Codes falling outside the NPT framework** | |
| | • Inherent personal attributes such as personal physical or cognitive abilities that could promote or inhibit DHI use. |

**DHI**: Digital health intervention; **HCP**: Healthcare professional

For peer review only

## Results

Study selection

Of 14191 citations identified, 5973 were excluded as duplicates; a further 7436 were excluded following title screening, 677 at abstract screening and 100 at full text screening. Overall, five full text articles were included in the review (Figure 1). These articles described four separate studies and included a total of 75 participants. The two articles (27, 28) reporting on the same study (Oneself) consisted of a qualitative



For peer review only - http://bmjopen.bmj.com/site/about/guidelines.xhtml

evaluation of a website (28) and a mixed-method reporting of the same qualitative data combined with quantitative (pre- and post-use surveys and log files) data (27). As these two studies included the same qualitative data and user quotes, they were combined for analysis purposes.

**Figure 1** PRISMA flow diagram illustrating the screening process (Adapted from Moher et al (21)).

Study characteristics

The Get Well Fast (36) and Oneself studies (27, 28) were undertaken between 2006 and 2008 in the Netherlands and Switzerland, respectively. The MyBehaviorCBP study was conducted in the US between 2012 and 2014 (37), whilst the study period for the Swedish Web-BCPA study was not reported (38). The characteristics of the study participants are summarised in Table 3. No information was reported on comorbidities or ethnicity and only limited information on participant socioeconomic status was included.

For peer review only



For peer review only - http://bmjopen.bmj.com/site/about/guidelines.xhtml

**Table 3:** Participant characteristics of included studies

| Study; Country | Year of study | Number of participants in qualitative study | Age range | Sex (%) | SES |
|---|---|---|---|---|---|
| Oneself (27, 28)<br>Switzerland | 2006-2008 | N = 18 | 28-72 years<br><br><29 yrs: n = 1<br>30-39 yrs: n = 3<br>40-49 yrs: n = 5<br>50-59 yrs: n = 6<br>>60 yrs: n = 3 | 50% female | *Education*:<br>Secondary school: n = 2; High school or equivalent: n = 11; University degree: n = 5 |
| Get Well Fast (36)<br>Netherlands | 2008 | N = 28<br><br>OP+ = 11<br>OP- = 8<br>Employee: 9 | 40-50 years | OP: N/R<br><br>Employee: 33% female | White and blue-collar workers. Various levels of education |
| MyBehaviorCBP (37)<br>USA | 2012-2014 | N = 10 | 31-60 years | 70% female | N/R |
| Web-BCPA (38)<br>Sweden | N/R | N = 19 | 27-60 years | 79 % female | *Education:*<br>Elementary school: n = 2;<br>Secondary school: n = 12;<br>University degree: n = 5)<br><br>*Employment:*<br>Permanent employment: n = 12;<br>Temporary employment: n = 3;<br>Unemployed: n = 3; Social benefits: n = 1 |

**N:** Number; **OP+**: occupational physicians who recruited patients into DHI; **OP-:** occupational physicians who did not recruit patients into DHI; **N/R:** not reported; **SES:** socioeconomic status

DHI delivery mode varied between studies. In the Oneself, Get Well Fast and Web-BCPA studies, the DHI consisted of information available on websites to which participants had either open access (27, 28) or had personal log-ins (36, 38). The content of the MyBehaviorCBP intervention was delivered to participants via a mobile phone app (37). Two of the studies tailored the content of their DHI to the individual participant by collecting information about the users and providing content that matched their needs (36, 37); in the Get Well Fast study, content was tailored based on patient reports on pain, limitations, treatment,



For peer review only - http://bmjopen.bmj.com/site/about/guidelines.xhtml

For peer review only

counselling, reintegration to work, work situation and work characteristics, relations at work, personality and daily activities (36), while the MyBehaviourCBP intervention collected sensory data from the users' smartphone (accelerometer signals and geolocation) and patient self-reported physical activity logs (37). Three interventions offered time limited programs of either five (36, 37) or eight weeks (38), while the fourth intervention was an open-to-access website with no time restrictions (27, 28) (Table 4).

**Table 4:** Participant inclusion criteria, sampling procedure for qualitative component and characteristics of digital intervention in included studies

| Study | Inclusion criteria for digital health intervention | Inclusion criteria and sampling procedures for qualitative study | Characteristics of digital health intervention |
|---|---|---|---|
| Oneself (27, 28) | • Anyone could register and use the Oneself website. | • Registered users of Oneself for at least 6 months.<br>• Visited the website at least 3 times.<br>• Suffering from chronic LBP (duration not defined).<br>• Living in the Italian part of Switzerland.<br>• Invitation to participate in interview sent via email to registered users. | Open access website containing:<br>• Library – textual educational information on back pain.<br>• Radio – 10x2-minute recorded audio messages on relevant topics.<br>• Gym - videos demonstrating stretching, stabilization and mobilization exercises accompanied by photographs and written descriptions.<br>• Forum – users could interact with other users and HCPs, monitored by a content manager.<br>• Chat room – users could interact with other users and HCPs. Once a week, a HCP would be available to discuss specific topics selected from conversations published on the Forum.<br>• Specialist answers – information on topics suggested by users.<br>• Testimonials - users could share stories and comment on other users' stories.<br>• Ability for users to request information they felt lacked on the website. |



For peer review only - http://bmjopen.bmj.com/site/about/guidelines.xhtml

| | | | |
|---|---|---|---|
| Get Well Fast (36) | • Employees of KLM Royal Dutch Airlines or National Railways and their OPs.<br>Employee criteria:<br>• Contracted for at least 12 hours per week.<br>• Absent from work for a minimum of 2 weeks due to non-specific back or neck pain.<br>• No serious health problems defined as "warning flags: e.g. fever, pain in arms or legs, serious disease".<br>• Ability to speak and write in Dutch.<br>• Internet access. | • Users of the Get Well Fast website.<br>• The employees' OPs.<br>• Sampling procedure N/R. | • Web-based, 5-weeks programme during which the employee completed 4 questionnaires and received tailored information via a personal digital diary.<br>• Based on weekly questionnaires, information about advice on improving physical fitness, setting a daily timetable, pain-coping strategies, and exercise instructions is provided.<br>• Employees spent around 15 minutes/day reading information, completing questionnaires, and following exercises.<br>• Employee's OP had access to the employee's diary and received reports when the employee completed a questionnaire, detailing the employee's condition, current treatments, and absence details. |
| MyBehaviorCBP (37) | • Aged 18-65 years<br>• History of chronic back pain (≥6 months).<br>• Willingness to use MyBehaviorCBP app on an Android mobile phone (own or provided by study).<br>• Reasonable level of outdoor movement (e.g. travelling to and from work).<br>• Not being significantly housebound.<br>• Fluent in English<br>• Basic level of mobile proficiency. | • None in addition to inclusion criteria for digital intervention.<br>• Sampling procedure N/R. All participants in digital intervention received exit survey. | • 5-week app based programme during which participants received recommendations for PA.<br>• App tracks participant's mobility state and geolocation using in-phone sensors or manual input. Recurring patterns of PA form base for new PA recommendations.<br>• Week 1 - baseline period: no recommendations were given.<br>• Week 2 & 3 - control phase: PA recommendations were random, generic and unrelated to participants' past behaviour.<br>• Week 4 & 5 – experimental phase: PA recommendations generated by MyBehaviorCBP based on PA |



For peer review only - http://bmjopen.bmj.com/site/about/guidelines.xhtml

| | | | |
|---|---|---|---|
| | | | behaviour during control phase.<br>• Participants were blinded to when the different PA recommendation forms were activated.<br>Participants completed a daily in-phone survey regarding ease of following recommendations, how many recommendations they followed, and their emotional state. |
| Web-BCPA (38) | • Aged 18-63 years.<br>• Persistent musculoskeletal pain with duration of at least 3 months in the back, neck, shoulder, and/or generalised pain.<br>• OMPSQ score ≥90, screening for psychosocial factors that indicates an estimated risk for long-lasting pain and future disability (39).<br>• Work ability of at least 25% (assessment method N/R).<br>• Familiar with written and spoken Swedish.<br>• Internet and computer access. | • Participants must have spent at least 15 minutes per module in 5 of 8 modules.<br>• Participants had to have reached their 4-month follow-up assessment<br>• Information about interview study in conjunction with 4-month follow-up. Formal invitation via telephone. | • Website-based Web Behavior Change Program for Activity (Web-BCPA) in combination with MMR.<br>• Web-BCPA consisted of eight modules: 1) pain, 2) activity, 3) behavior, 4) stress and thoughts, 5) sleep and negative thoughts, 6) communication and self-esteem, 7) solutions, and 8) maintenance and progress.<br>• Modules contained information, assignments and exercises delivered as educational texts, videos and writing tasks.<br>• Participants could access 1 new module/week during the first 8 weeks of rehabilitation, and had access to the website 24/7 for 4 months. |

**HCP**: healthcare professional; **OP**: occupational physician; **OMPSQ**: Örebro musculoskeletal pain screening questionnaire; **MMR**: multimodal rehabilitation; **PA**: physical activity; **N/R**: not reported



For peer review only - http://bmjopen.bmj.com/site/about/guidelines.xhtml

Qualitative components of included studies

Where reported, sampling procedures for the qualitative parts of the studies varied across studies (Table 4). Qualitative interviews were conducted via telephone (36), in the participant's home (27, 28, 38), or at a local university (27, 28), health care centre (38) or council building (38). All of the interviews were semi-structured, recorded and either transcribed verbatim (27, 28, 38) or as written descriptions of answers including quotes (36). For the MyBehaviorCBP study (37), free-text answers from the electronic exit survey were extracted. Data was then analysed to identify common themes (27, 28, 36-38).

Quality appraisal

The comprehensiveness of reporting varied across the included studies (Supplementary File 2) and ranged from 13 (41%) to 23 (72%) of the 32-item COREQ checklist (25, 26). Items within domain 1 (Research team and reflexivity) generally had very poor reporting with several items not reported by any studies. All studies reported sampling procedure, sample size, setting of data collection, description of sample, recording, derivation of themes, quotations presented, consistency of data and findings and clarity of major themes

Engagement strategies

We defined engagement strategies as any method used to recruit and initially motivate participants to enrol in the DHI study. The identified engagement strategies included: use of mailing lists of retired personnel (37); mailing list for a university wellness centre (37); or invitation from OP or HCP (27, 28, 36). In addition, the Oneself study advertised for participation through media: radio (project leader and managers interviewed about project at local radio station), television (rheumatologists involved in project spoke about project on local television station), and through a press conference for which the major daily journals from the area were invited (27, 28).

Barriers and facilitators for uptake and utilisation of digital health interventions



For peer review only - http://bmjopen.bmj.com/site/about/guidelines.xhtml

We identified four major themes: 1) IT usability and accessibility, 2) Quality and amount of content, 3) Tailoring and personalisation, and 4) Motivation and support (Table 5). Under each theme, both barriers and facilitators were identified. Distinction between uptake (initial engagement) and utilisation (use) in the included studies was not possible, and they are therefore treated as one. Participant quotes are provided in the text to substantiate the data for each theme. More exemplar quotations are provided in Supplementary File 3.

**Table 5:** Factors affecting uptake and utilisation of DHIs for self-management of LBP

| Theme | Subtheme | Barriers | Facilitators |
|---|---|---|---|
| **IT usability and accessibility** | Functionality and usability | • Too much choice between functions<br>• Fixed advancement pace<br>• Issues logging into DHI<br>• *Low user-friendliness<br>• *Issues logging into DHI<br>• *Low level of functionality (e.g. registration, navigation, helpdesk) | • Flexible structure and navigation<br>• Conveniently arranged<br>• Variation of media types (text, audio and video)<br>• Reminders and notifications<br>• High user-friendliness<br>• *High user-friendliness |
| | IT affinity | • Lack of affinity with computers<br>• *Lack of affinity with web-based programmes | • Enjoying working with a computer |
| | Access and convenience | • Not able to choose starting time of DHI<br>• *No access to computer during consultation | • Easily accessible with low effort<br>• Accessible at all hours and locations<br>• Accessible even during periods with severe pain symptoms<br>• Ability to take all the time needed |
| **Quality and amount of content** | Quality of content | • Contradictory content between DHI and HCP | • Trustworthy content and source<br>• Easily understandable content<br>• High quality of content<br>• Steady content<br>• *Appropriate content |
| | Amount of content | • Too much content to choose from<br>• Too much information to fully comprehend | • A lot of content to choose from |



For peer review only - http://bmjopen.bmj.com/site/about/guidelines.xhtml

| | | | |
|---|---|---|---|
| **Tailoring and personalisation** | Tailoring, specificity and personalisation | • Content not tailored to individual needs and/or pain severity<br>• Content perceived not new or relevant | • Content accounting for individual needs and/or pain severity<br>• Self-identification in content<br>• Opportunity to influence treatment |
| **Motivation and support** | Personal attributes and resources | • Adhering to biomedical model of LBP<br>• Seeing LBP as a marginal problem<br>• Preferring other treatment regimens, e.g. with human contact<br>• Lack of knowledge about LBP and treatments<br>• Physical health (e.g. pain, fatigue)<br>• Psychological symptoms | • High level of awareness and self-management of LBP<br>• Aware that LBP would not be fixed with a medical solution and ready to accept active role<br>• Emotional and cognitive resources, e.g. motivation, interest, commitment and self-confidence in self-management of LBP<br>• Enjoy solution focused work |
| | Support to use DHI | • HCP unsupportive of use of DHI<br>• No support from authorities | • HCP supportive of use of DHI<br>• Support from family<br>• Support from authorities<br>• Support from other suffers (e.g. successful testimonials) |
| | Features of DHI | • DHI not guiding or supporting participants enough (e.g. to plan for execution of physical activity recommendation from DHI) | • Interaction/interactivity<br>• Information about self-management of LBP<br>• Goal-setting<br>• Action-planning<br>• Follow-up and evaluation<br>• Adjusting treatment related to setbacks and progress<br>• Monitoring own progress in graphs<br>• Variation of content<br>• Update of content |
| | HCP factors for support of patients | • *Time restrictions of consultations<br>• *Difficulty keeping DHI in mind during consultations<br>• *Difficulty providing patients with accurate information about DHI<br>• *Perceiving no benefit of DHI compared to usual treatment<br>• *Preferring other treatment regimens, e.g. with human contact | • *DHI a good medium for counselling employees |

For peer review only

*: Occupational physician perspective; **IT**: information technology; **HCP**: healthcare professional; **DHI**: Digital health intervention

*1) IT usability and accessibility*

The first theme that emerged concerned functionality and usability, IT affinity or access and convenience of the DHI. A flexible and convenient structure with high user-friendliness aided use of DHIs (36, 38). Inclusion of a variety of media types such as video was also appreciated (27, 28) as well as getting reminders or notifications from the DHI (27, 28).

*"Usually I went on the website when I read the newsletter. I read the letter and then I'm there, it's like a conditioned reflex (Woman, 49, nurse)"* (27, 28).

On the other hand, low user-friendliness and problems with logging in were barriers for use of DHIs for both study participants and HCPs (36). A fixed starting point or set advancement pace were also demotivating for some users (38). Affinity with computers and web-based programmes highly affected uptake of DHIs. Participants with a high level of computer affinity and who enjoyed working on a computer expressed positive feelings towards using DHIs (38), whereas lack of computer affinity was an important barrier for uptake of the intervention (36). Accessibility to a computer was not surprisingly a requirement for uptake to the study. When computers were readily available, DHIs were considered easy to access with unlimited 24h access (27, 28, 38).

*"… thanks to the program (the Web-BCPA) I was able to perform the basic body awareness exercises of my own choice… and to repeat those that I felt most effective as many times that I preferred… the flexibility made it mine (the rehabilitation) (Woman, participant)"* (38).

Even during periods with severe pain symptoms, a DHI was considered an attainable and effortless option as participants did not have to go anywhere (e.g. a healthcare centre) (27, 28, 38).

*2) Quality and amount of content*

For peer review only



For peer review only - http://bmjopen.bmj.com/site/about/guidelines.xhtml

Quality and amount of content provided in DHIs affected use for both participants and HCPs. Trustworthiness of the source and information provided facilitated use, and participants seemed to be reassured when knowing the content had been reviewed and validated by HCPs (27, 28, 38). For participants, richness and consistency of content facilitated use (27, 28), especially when the content was easily understandable (36).

*"Knowing that there is a serious website where there are contributions, it strengthens you a bit (Woman, 37, teacher"* (28).

Likewise, content that suited the patients was appreciated by HCPs (36). On the other hand, when participants experienced contradictory advice from their HCP and the DHI, this was a barrier for using the DHI (36). Large volumes of information or too much content to choose from also limited uptake and utilisation, particularly in relation to the amount of time required to go through it (27, 28, 36).

*"There is a lot of information, probably almost too much, don't you think? (Man, 47, bank director)"* (27, 28).

### *3) Tailoring and personalisation*

The participants' perception of the degree of tailoring and personalisation of the content to their needs was the third major theme affecting use of DHIs for self-management of LBP. Self-identification increased utilisation of DHIs when participants were able to recognise themselves in the content, e.g. in the information and explanations about pain and symptoms, or thoughts related to dealing with LBP (27, 28, 38).

*"It gives you descriptions and you say: this stuff here... I see it, I see it! I recognise myself in it, I recognise myself here (Man, 58, teacher)"* (27, 28).

When the content of the DHI accounted for the individual participant's activities, needs or pain severity it further encouraged use of the DHI (36-38).



For peer review only - http://bmjopen.bmj.com/site/about/guidelines.xhtml

*"I really liked the personalization. I thought it was a nice touch. Suggestions were more specific and tailored, which for me made them more relevant and likely for me to use them (Participant)"* (37). Participants appreciated the opportunity to influence their own rehabilitation by being able to select exactly what they wanted from a variety of options that fitted their situation (37, 38).

*"Previously I had read about CBT (Cognitive Behavioral Therapy), but I had never thought of it as a help for my condition... I want to compare this rehabilitation with a smorgasbord from which is it easy to taste (Participant)"* (38).

When content was not tailored to the individual participant or the participant's pain severity, it was experienced as a barrier for use of the DHI as it was not perceived to apply to their situation. This in turn would negatively impact the participant's motivation and sustained engagement (28, 36). Content that was not perceived relevant or new to the participant could also lead to a feeling of hopelessness as participants' got the impression that there was no solution to their problem (28).

*4) Motivation and support*

The fourth major theme related to the participants motivation and support, and included subthemes related to the personal attributes and resources of participants, support to use DHIs, features of DHIs, and lastly HCPs' perceptions and how they affect HCPs' support of DHIs. Specific participant attributes impacted the utilisation of DHIs; already being involved or being ready to accept an active role in rehabilitation (27), and having motivation, interest, commitment and confidence in self-managing LBP facilitated use (27, 28, 38). Enjoying solution focused work, e.g. as experience from day job, was also a facilitator (38). Contrary, not wanting to take an active role (27), or preferring other treatment regimens (27) hindered use, as well as lacking information about treatments (38) or preferring other available treatment regimens, e.g. with human contact (36). Relying on a HCP to find a solution (27, 28) or seeing LBP as only a marginal problem, led to lower motivation for use of the DHI (27). Furthermore, use of DHIs was constrained by physical (36, 38) or psychological (38) restrictions. Getting support from a variety of sources facilitated use; both support



For peer review only - http://bmjopen.bmj.com/site/about/guidelines.xhtml

from outside and within the DHI. Support from family, authorities and HCPs was perceived as encouraging (38), and so were successful testimonials from other users whose LBP symptoms had improved (27, 28).

*"When you are going through a moment when you have backache and you read a testimony which says 'yes, there is someone who was able to do it', it gives you hope (Woman, 28, academic researcher)* (27, 28).

Not having HCPs or local agencies (e.g. authorities) support in their use of the DHI held participants back from utilising DHIs to manage their LBP (36, 38).

*"I expected more commitment from my OP [occupational physician] (Employee)"* (36).

Features of DHIs could both facilitate and restrain use. DHIs that were interactive, used goal-setting and action-planning, and had a great variation of content encouraged use (37, 38). Participants also appreciated information that guided them on how to self-manage their LBP (e.g. exercises and advice) (27, 28, 36-38), and some participants felt updates of content facilitated their use further (27, 28). Furthermore, DHIs that allowed participants to monitor and reflect on their own progress, improvement or goal attainment, e.g. through interactive graphs, were considered to enable self-management actions and to motivate further use (38). Follow-up and evaluation on goal achievement was also appreciated and reinforced the importance of tailoring DHIs towards individual participant's experience.

*" ... days when I had a lot of pain I used to remain sedentary, and as soon as I had a better day I was eager to do all kinds of activities that day.. before I started with the assignment activity planning (in the Web-BCPA) I was not aware of how my behaviour related to the days with pain, but by monitoring this over time I started to plan my daily activities in a more balanced way (Woman, participant)"* (38).

On the contrary, DHIs that did not support or guide participants enough, e.g. to execute recommendations given by the DHI, were perceived as constraining (37).

HCPs had reasons to support or not support participants' use of DHIs for self-management of LBP. HCPs either did not perceive additional benefits of DHIs compared to usual care or preferred other treatment regimens, e.g. ones that involved physical contact (36).

For peer review only



For peer review only - http://bmjopen.bmj.com/site/about/guidelines.xhtml

*"The ability to touch people is an essential element in the treatment of people with back or neck pain (Occupational physician)"* (36).

HCPs also reported having too little time during consultations to support use of DHI or difficulty in keeping the DHI in mind during their consultation – and even if they remembered it, they struggled with providing patients with accurate information about the DHI (36). However, HCPs who perceived DHIs as a good medium for counselling were positive about using and recommending DHIs (36).

Suggestions for improved utilisation

Participants of all included studies provided the authors with suggestions for how DHIs could be improved to facilitate continued or improved utilisation. As these items were only perceived as potential facilitators if implemented they are reported separately from the themes above. Some suggestions were improvement of usability of existing DHIs, e.g. increased user-friendliness (36), incorporation of illustrations and cartoons (36), or easier registration (36). Optimisation of tailoring to adjust for changes over time (36), or better adaption of physical activity recommendations that accommodated differences between weekdays and accounted for weather forecasts was also suggested (37). System improvements that enabled the DHI to learn from participants' activity level related to their pain days was also proposed (37). Lastly, application of a participatory approach for the process of designing DHIs was suggested (38). Other suggestions were new features to add to DHIs, e.g. direct contact to HCPs via DHI (36), a helpdesk (36), content about how to deal with LBP mentally (36), and a sophisticated reminder system with just-in-time notifications for both planning and execution of physical activities (37).

Developing a conceptual understanding

We applied the NPT framework (Table 2) to the taxonomy of barriers and facilitators as summarised in Table 5. Most of the identified codes fell within the four NPT constructs, with the exception of codes related to participants' own physical, mental and emotional health, which although affecting an individual's



For peer review only - http://bmjopen.bmj.com/site/about/guidelines.xhtml

For peer review only

capacity, they are not specific actionable tasks involved in the uptake and utilisation of a DHI for LBP. Applying the NPT framework allowed us to conceptualise how the codes identified may affect the uptake and utilisation of DHIs for the self-management of LBP, at both an individual and collective level, through the four stages of deciding whether to enrol, engage, utilise and maintain engagement with such a tool.

**Figure 2** Preliminary conceptual model of barriers and facilitators to uptake and utilisation of LBP DHIs

## Discussion

We have conducted a systematic search of the literature to explore the methods used to encourage participation with DHIs for the self-management of LBP and the barriers and facilitators to patient uptake and utilisation of these tools. Our review identified four studies published in five articles, demonstrating that the literature remains sparse.

Our review has enabled us to develop a preliminary conceptual model for engagement and utilisation of a DHI for LBP self-management by applying the NPT framework to the barriers and facilitators identified in the included studies. The model suggests that users value DHIs that are easily understandable, which they can navigate at their own pace and which help enhance subsequent communication with HCPs, family and colleagues. Providing regular updates and prompts appears to help users engage with DHIs whilst the ability to interact with other users is viewed positively in terms of providing support, motivation and validation. Users expect information to be easily accessible, structured, up-to-date and accurate, with tailoring to individual user experience being particularly valued.

Conversely, large volumes of information and lack of time appear to have a negative impact on user understanding, motivation and engagement. Lack of support or encouragement by HCPs also appears to be off putting for some whilst others face challenges accessing the DHIs. Participant's own attributes including the symptoms they experienced and their attitudes and preferences for treatment for LBP can further restrict capacity to self-manage and influence motivation and engagement with DHIs. Other significant

For peer review only



For peer review only - http://bmjopen.bmj.com/site/about/guidelines.xhtml

barriers to user engagement and utilisation include missing or conflicting information, content that was not tailored to the individual, and lack of feedback or evaluation.

In this review we explored how studies engaged participants to enrol into the study and begin using a DHI, this was mainly through identification of potential participants and subsequent invitation. Sustaining engagement beyond initial participation was not discussed in-depth in any of the included studies, some used email prompts and regular updates or newsletters. However, all studies did report participants' suggestions to improve DHIs, which mainly focussed on improving usability, (dynamic) tailoring of content, additional features to support users and the inclusion of participants in the design of DHIs. While not considered as facilitators to uptake and utilisation, some positive consequences of using the DHIs were identified by some users, e.g. acquiring a vocabulary and an individual understanding of their situation, and increased confidence in self-managing their LBP, which may have reinforced users in their self-management and in turn may have increased use of DHIs. Further, some general points to increase utilisation of DHIs for LBP were highlighted by participants, including the importance of participatory involvement of patients in the development of a DHI.

Comparison with previous literature

Although there was a significant variation in intervention recruitment and content in studies included in this review, there was a large degree of overlap in terms of the barriers and facilitators identified. Many of these are generally in keeping with the findings of other qualitative reviews for DHIs in general (19, 40) as well as those looking specifically at hypertension (41) and pain management in older adults (42). A review by O'Connor et al (19) identified four main themes relating to barriers and facilitators to engagement and recruitment to DHIs in general: personal agency and motivation; personal life and values; engagement and recruitment approach, and quality of the DHI. Another review by Hardiker & Grant (40) identified five overarching themes concerning barriers and facilitators influencing engagement with eHealth services: characteristics of users; technological issues; characteristics of eHealth services; social aspects of use; and



For peer review only - http://bmjopen.bmj.com/site/about/guidelines.xhtml

eHealth services in use. Despite the differing terminology of the major theme headings used in these studies and those found in this review, comparison of the codes or subthemes reveals the barriers and facilitators to be broadly similar, suggesting that these may be generally transferable across DHIs. The main exception is the specific mention of security and privacy of personal information in these earlier reviews (19, 40), which was not found as a barrier in this review, although this may be due to the small number of studies and participants.

*Functionality and general IT issues*

Factors including age, ethnicity, economic status, level of educational attainment and familiarity with the internet are recognised as being significant factors influencing access to and engagement with DHIs (40). O'Connor et al. (19) reported that a lack of digital literacy, issues accessing IT equipment or the internet and the cost of such equipment or access are barriers to the use of DHIs. The user friendliness, design and ease of registration/logging in to a DHI were found to be significant issues for users in this review and should be carefully considered when planning a DHI.

For peer review only

*Quality and amount of content*

Trust is a significant issue when accessing information online (40). Clinical endorsement seems to be important to users in terms of the perceived quality of content and is in keeping with the findings of other studies in this area (19, 43). Additionally, consideration should be given to the potential for users to receive contradictory advice from the DHI and their HCP. Our findings suggest that whilst some users considered large volumes of information as a barrier, others valued the ability to read widely on the subject. This is thought to reflect individual preference and personal factors such as time pressures. Taking such preferences into account during the development and delivery of DHIs may increase user engagement.

*Tailoring and personalisation*



For peer review only - http://bmjopen.bmj.com/site/about/guidelines.xhtml

It is clear from our findings that user's symptomology, prior knowledge and experience play a role in engagement. Tailoring DHIs to the user's individual symptoms and functional limitations is thought to enhance engagement (19) and may thus improve the effectiveness of the intervention. A recent review of DHIs for the self-management of LBP (17) found that no DHI for LBP used tailoring to enhance effectiveness, but commented that this could be an important means of enhancing engagement. In addition, O'Connor et al. (19) recommended that any DHI should be designed and tailored to individual needs in order to reduce the self-care burden. Our findings suggest that users improved understanding of LBP and enhanced communication with their HCP during subsequent consultations. Some users commented that they would have appreciated some direct support from a HCP or that this might have enhanced engagement. This finding is consistent with those of Steele et al (44), who during an evaluation of an internet-based physical activity behaviour change program, found that many participants in the internet group would have preferred traditional face-to-face sessions. Some of the occupational physician's interviewed felt that they did not have the time and capacity within their consultation to discuss DHI use in detail (36). If the intended purpose of a DHI is to facilitate HCP – patient communication then how the DHI or a supporting HCP dashboard could be designed to allow for efficient and useful interactions during a consultation should be considered at the design and development stage.

*Motivation and support*

Personal recommendations and social support were recognised as being important in encouraging DHI user registration and in fostering engagement (19). We found that some users valued the emotional support of being able to interact with other users. Whilst this was a positive finding in our study and is consistent with those reported elsewhere (40), there exists the possibility of potentially abusive or threatening behaviours developing online which could act as a barrier to some (45). Other reports of discussion threads deviating from the original topic or containing misleading information (40) raise questions on the need for monitoring such interactive features. Our findings further suggest that an individual's personal attributes



For peer review only - http://bmjopen.bmj.com/site/about/guidelines.xhtml

and resources (e.g. emotional and cognitive) and attitudes towards self-management can influence their use of DHIs. Additional support may therefore be required for some potential users to participate and benefit from DHIs.

O'Connor et al (19) reported that some individuals do not view technology as a way of addressing healthcare needs and prefer alternative approaches to managing their health issues such as seeking support from family, friends or healthcare professionals. They also highlight the potential for DHIs to be impersonal and commented on the lack of a therapeutic relationship, particularly in situations where sensitive health or social issues are involved. Such views were also reflected among individuals, including some HCPs, in our findings. In contrast, other users appreciate the freedom to access health information at a time and place that suits the user along with the anonymity DHIs can offer (43), issues that can be challenging for traditional healthcare services to match.

Strengths and limitations

This systematic review was conducted by an experienced team and follows the PRISMA guidelines for the reporting of systematic reviews. Our iterative search strategy utilised multiple databases and involved independent data extraction, quality appraisal and data analysis by two reviewers, with a third reviewer adjudicating in the case of any disagreements.

Our review does however have some limitations. Many DHIs are developed commercially and do not undergo formal academic evaluation (15) resulting in relatively sparse literature in this area. Our search strategy involved a number of constraints and focussed on published literature which may have inadvertently excluded potentially useful studies. Further relevant research may also have been missed as grey literature was not included in our search strategy. Our analysis and synthesis of data was based on



For peer review only - http://bmjopen.bmj.com/site/about/guidelines.xhtml

reviewing published literature, not the original data, which could have impacted on the background context to some of the quotes used.

The studies included in this review (27, 28, 36-38) were conducted in real-life settings and as a result sampling procedures were acknowledged as being convenient, had the potential to be biased towards individuals who found the interventions beneficial and may not have been representative of all users. Furthermore, the literature contained very limited information on user's sociodemographic characteristics. However, as a consequence of the small number of studies identified by our search strategy, we did not exclude studies on the basis of quality, potentially reducing the reliability of the findings of this review.

Finally, given the limited number of studies and research context, it is possible that not all the important barriers and facilitators may have been identified, and thus our conceptual model must be considered preliminary and will need confirmation by further research. This information will be of particular use to those involved in designing and implementing DHIs focussed on self-management of LBP and more widely.

**Conclusions**

Our systematic review highlights barriers and facilitators affecting the utilisation of DHIs for the self-management of LBP and identified key areas involved in embedding such interventions into everyday practice. The limited and varied quality of literature found by this review suggests that further primary research investigating the implementation of DHIs and user's experiences is required. Future research should aim to describe DHIs and their users in more detail and include descriptions of engagement strategies and barriers or facilitators encountered in order to enhance our knowledge of which approaches are likely to have the greatest impact on user engagement and outcomes, and for whom.



For peer review only - http://bmjopen.bmj.com/site/about/guidelines.xhtml

**List of abbreviations**

COREQ - Consolidated Criteria for Reporting Qualitative Research

DHI - Digital health intervention

HCP – Healthcare professional

IT - Information technology

LBP - Low back pain

NPT - Normalization process theory

OP - Occupational physician

PA – Physical activity

PRISMA - Preferred reporting items for systematic reviews and meta-analyses

**Supplementary File 1:** Search details, as previously described and published by Nicholl et al. (17)

**Supplementary File 2:** Consensus summary of quality appraisal as per 32-item COREQ checklist and comprehensiveness of reporting

**Supplementary File 3:** Taxonomy of barriers and facilitators with exemplar quotations

**Declarations**

Ethics approval and consent to participate: Not applicable

Consent for publication: Not applicable

Patient and public involvement: This research was done without patient involvement. Patients were not invited to comment on the study design and were not consulted to develop patient relevant outcomes or interpret the results. Patients were not invited to contribute to the writing or editing of this document for readability or accuracy.

Acknowledgements: We would like to thank our librarian adviser Ingrid Ingeborg Riphagen, Department of

For peer review only



For peer review only - http://bmjopen.bmj.com/site/about/guidelines.xhtml

Public Health and Nursing, Faculty of Medicine and Health Sciences, Norwegian University of Science and Technology (NTNU).

**Contributorship statement**: Study design was developed by all authors. Title, abstract and full text screening was performed JK, MaS, KC and KW with any discrepancies being resolved by BN, CR and MeS. JK, MaS, BN and KW carried out data extraction. JK, KW and MaS conducted data synthesis and analysis aided by BN and FM. Quality appraisal was assessed by BN, KC and KW. LS, CR and MeS critically scrutinized first drafts and provided comments. All authors read, commented and approved the final manuscript.

**Competing interests:** The authors declare that they have no competing interests

**Funding**: This project has received funding from the European Union Horizon 2020 Research and innovation programme under grant agreement No 689040.

**Data sharing statement**: The datasets used and analysed during the current study are available from the corresponding author on reasonable request.

For peer review only

**References**

1. Hoy D, Bain C, Williams G, March L, Brooks P, Blyth F, et al. A systematic review of the global prevalence of low back pain. Arthritis and rheumatism. 2012;64(6):2028-37.
2. Vos T, Flaxman AD, Naghavi M, Lozano R, Michaud C, Ezzati M, et al. Years lived with disability (YLDs) for 1160 sequelae of 289 diseases and injuries 1990-2010: a systematic analysis for the Global Burden of Disease Study 2010. Lancet (London, England). 2012;380(9859):2163-96.
3. Nolet PS, Kristman VL, Cote P, Carroll LJ, Cassidy JD. Is low back pain associated with worse health-related quality of life 6 months later? European spine journal : official publication of the European Spine Society, the European Spinal Deformity Society, and the European Section of the Cervical Spine Research Society. 2015;24(3):458-66.
4. Dagenais S, Caro J, Haldeman S. A systematic review of low back pain cost of illness studies in the United States and internationally. The spine journal : official journal of the North American Spine Society. 2008;8(1):8-20.
5. Maniadakis N, Gray A. The economic burden of back pain in the UK. Pain. 2000;84(1):95-103.
6. (NICE) NIfHaCE. Low back pain and sciatica in over 16s: assessment and management: National Institute for Health and Care Excellence (NICE). 2016 [Available from: https://www.nice.org.uk/guidance/ng59.
7. Authority DH. National clinical guideline: Interventions for recent onset low back pain [in Danish]: Danish Health Authority; 2016 [Available from: https://www.sst.dk/da/udgivelser/2016/nkr-laenderygsmerter.
8. Sluijs EM, Kok GJ, van der Zee J. Correlates of exercise compliance in physical therapy. Physical therapy. 1993;73(11):771-82; discussion 83-6.



For peer review only - http://bmjopen.bmj.com/site/about/guidelines.xhtml

9. Newson JT, Huguet N, Ramage-Morin PL, McCarthy MJ, Bernier J, Kaplan MS, et al. Health behaviour changes after diagnosis of chronic illness among Canadians aged 50 or older. Health reports. 2012;23(4):49-53.
10. Murray E, Burns J, See TS, Lai R, Nazareth I. Interactive Health Communication Applications for people with chronic disease. The Cochrane database of systematic reviews. 2005(4):Cd004274.
11. Beatty L, Lambert S. A systematic review of internet-based self-help therapeutic interventions to improve distress and disease-control among adults with chronic health conditions. Clinical psychology review. 2013;33(4):609-22.
12. Zhao J, Freeman B, Li M. Can Mobile Phone Apps Influence People's Health Behavior Change? An Evidence Review. Journal of medical Internet research. 2016;18(11):e287.
13. Hayden JA, van Tulder MW, Tomlinson G. Systematic review: strategies for using exercise therapy to improve outcomes in chronic low back pain. Annals of internal medicine. 2005;142(9):776-85.
14. Strecher V. Internet methods for delivering behavioral and health-related interventions (eHealth). Annual review of clinical psychology. 2007;3:53-76.
15. Lalloo C, Jibb LA, Rivera J, Agarwal A, Stinson JN. "There's a Pain App for That": Review of Patient-targeted Smartphone Applications for Pain Management. The Clinical journal of pain. 2015;31(6):557-63.
16. Garg S, Garg D, Turin TC, Chowdhury MF. Web-Based Interventions for Chronic Back Pain: A Systematic Review. Journal of medical Internet research. 2016;18(7):e139.
17. Nicholl BI, Sandal LF, Stochkendahl MJ, McCallum M, Suresh N, Vasseljen O, et al. Digital Support Interventions for the Self-Management of Low Back Pain: A Systematic Review. Journal of medical Internet research. 2017;19(5):e179.
18. Machado GC, Pinheiro MB, Lee H, Ahmed OH, Hendrick P, Williams C, et al. Smartphone apps for the self-management of low back pain: A systematic review. Best practice & research Clinical rheumatology. 2016;30(6):1098-109.
19. O'Connor S, Hanlon P, O'Donnell CA, Garcia S, Glanville J, Mair FS. Understanding factors affecting patient and public engagement and recruitment to digital health interventions: a systematic review of qualitative studies. BMC medical informatics and decision making. 2016;16(1):120.
20. International Register of Systematic Reviews P. [Available from: https://www.crd.york.ac.uk/PROSPERO/display_record.php?ID=CRD42016051182.
21. Moher D, Liberati A, Tetzlaff J, Altman DG. Preferred reporting items for systematic reviews and meta-analyses: the PRISMA statement. International journal of surgery (London, England). 2010;8(5):336-41.
22. McLean G, Band R, Saunderson K, Hanlon P, Murray E, Little P, et al. Digital interventions to promote self-management in adults with hypertension systematic review and meta-analysis. Journal of hypertension. 2016;34(4):600-12.
23. Fleming T, Bavin L, Lucassen M, Stasiak K, Hopkins S, Merry S. Beyond the Trial: Systematic Review of Real-World Uptake and Engagement With Digital Self-Help Interventions for Depression, Low Mood, or Anxiety. Journal of medical Internet research. 2018;20(6):e199.
24. Carolan S, Harris PR, Cavanagh K. Improving Employee Well-Being and Effectiveness: Systematic Review and Meta-Analysis of Web-Based Psychological Interventions Delivered in the Workplace. Journal of medical Internet research. 2017;19(7):e271.
25. Booth A, Hannes K, Harden A, Noyes J, Harris J, Tong A. COREQ (Consolidated Criteria for Reporting Qualitative Studies). In: Moher D, Altman DG, Schulz KF, Simera I, Wager E, editors. Guidelines for Reporting Health Research: A User's Manual. Oxford: Wiley; 2014. p. 214-26.
26. Tong A, Sainsbury P, Craig J. Consolidated criteria for reporting qualitative research (COREQ): a 32-item checklist for interviews and focus groups. International journal for quality in health care : journal of the International Society for Quality in Health Care. 2007;19(6):349-57.



For peer review only - http://bmjopen.bmj.com/site/about/guidelines.xhtml

27. Schulz PJ, Rubinelli S, Zufferey MC, Hartung U. Coping with Chronic Lower Back Pain: Designing and Testing the Online Tool ONESELF. Journal of Computer-Mediated Communication. 2010;15(4):625-45.
28. Caiata Zufferey M, Schulz PJ. Self-management of chronic low back pain: an exploration of the impact of a patient-centered website. Patient education and counseling. 2009;77(1):27-32.
29. Thomas J, Harden A. Methods for the thematic synthesis of qualitative research in systematic reviews. BMC medical research methodology. 2008;8:45.
30. Graneheim UH, Lundman B. Qualitative content analysis in nursing research: concepts, procedures and measures to achieve trustworthiness. Nurse Education Today. 2004;24(2):105-12.
31. Krippendorff K. Content Analysis: An Introduction to Its Methodology. 2nd ed. Thousand Oaks, CA: SAGE Publications; 2004. 422 p.
32. May C, Finch T. Implementing, Embedding, and Integrating Practices: An Outline of Normalization Process Theory. Sociology. 2009;43(3):535-54.
33. May CR, Mair F, Finch T, MacFarlane A, Dowrick C, Treweek S, et al. Development of a theory of implementation and integration: Normalization Process Theory. Implementation Science. 2009;4(1):29.
34. Mair FS, May C, O'Donnell C, Finch T, Sullivan F, Murray E. Factors that promote or inhibit the implementation of e-health systems: an explanatory systematic review. Bulletin of the World Health Organization. 2012;90(5):357-64.
35. Gallacher K, Morrison D, Jani B, Macdonald S, May CR, Montori VM, et al. Uncovering treatment burden as a key concept for stroke care: a systematic review of qualitative research. PLoS medicine. 2013;10(6):e1001473.
36. de Jong T, Heinrich J, Blatter BM, Anema JR, van der Beek AJ. The feasibility of a web-based counselling program for occupational physicians and employees on sick leave due to back or neck pain. BMC medical informatics and decision making. 2009;9:46.
37. Rabbi M, Aung MS, Gay G, Reid MC, Choudhury T. Feasibility and Acceptability of Mobile Phone-Based Auto-Personalized Physical Activity Recommendations for Chronic Pain Self-Management: Pilot Study on Adults. Journal of medical Internet research. 2018;20(10):e10147-e.
38. Nordin C, Michaelson P, Eriksson MK, Gard G. It's About Me: Patients' Experiences of Patient Participation in the Web Behavior Change Program for Activity in Combination With Multimodal Pain Rehabilitation. Journal of medical Internet research. 2017;19(1):e22-e.
39. Linton S. Early assessment of psychological factors: the Örebro Screening Questionnaire for Pain. In: Linton S, editor. New avenues for the prevention of chronic musculoskeletal pain. Amsterdam: Elsevier; 2002.
40. Hardiker NR, Grant MJ. Factors that influence public engagement with eHealth: A literature review. International journal of medical informatics. 2011;80(1):1-12.
41. Band R, Bradbury K, Morton K, May C, Michie S, Mair FS, et al. Intervention planning for a digital intervention for self-management of hypertension: a theory-, evidence- and person-based approach. Implementation science : IS. 2017;12(1):25.
42. Parker SJ, Jessel S, Richardson JE, Reid MC. Older adults are mobile too!Identifying the barriers and facilitators to older adults' use of mHealth for pain management. BMC geriatrics. 2013;13:43.
43. Skinner H, Biscope S, Poland B. Quality of internet access: barrier behind internet use statistics. Social science & medicine (1982). 2003;57(5):875-80.
44. Steele R, Mummery KW, Dwyer T. Development and process evaluation of an internet-based physical activity behaviour change program. Patient education and counseling. 2007;67(1-2):127-36.
45. Horvath KJ, Danilenko GP, Williams ML, Simoni J, Amico KR, Oakes JM, et al. Technology use and reasons to participate in social networking health websites among people living with HIV in the US. AIDS and behavior. 2012;16(4):900-10.



For peer review only - http://bmjopen.bmj.com/site/about/guidelines.xhtml

# PRISMA 2009 Flow Diagram

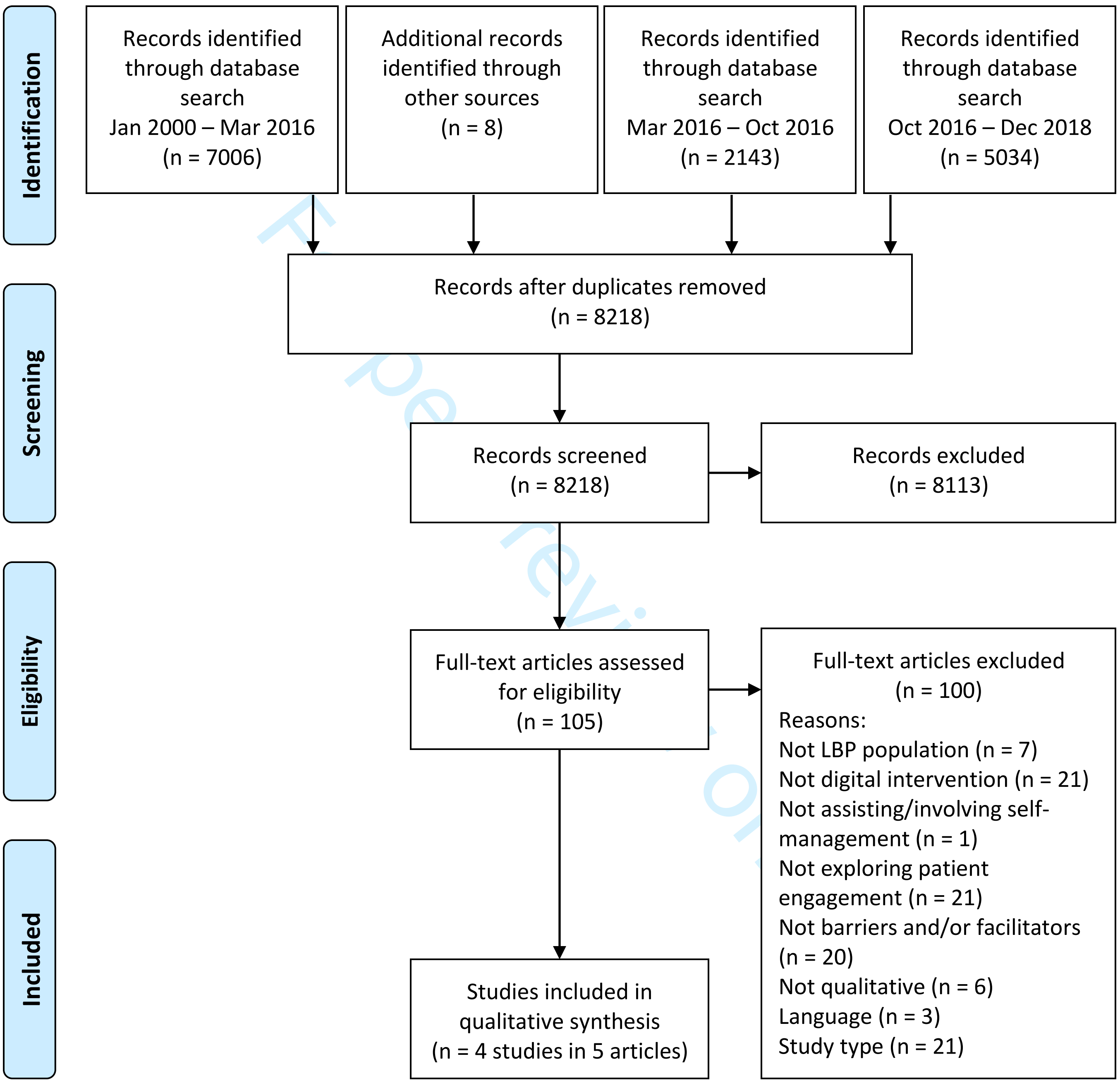


*From:* Moher D, Liberati A, Tetzlaff J, Altman DG, The PRISMA Group (2009). *P*referred *R*eporting *I*tems for *S*ystematic Reviews and *M*eta-*A*nalyses: The PRISMA Statement. PLoS Med 6(7): e1000097. doi:10.1371/journal.pmed1000097

**For more information, visit www.prisma-statement.org.**

For peer review only - http://bmjopen.bmj.com/site/about/guidelines.xhtml

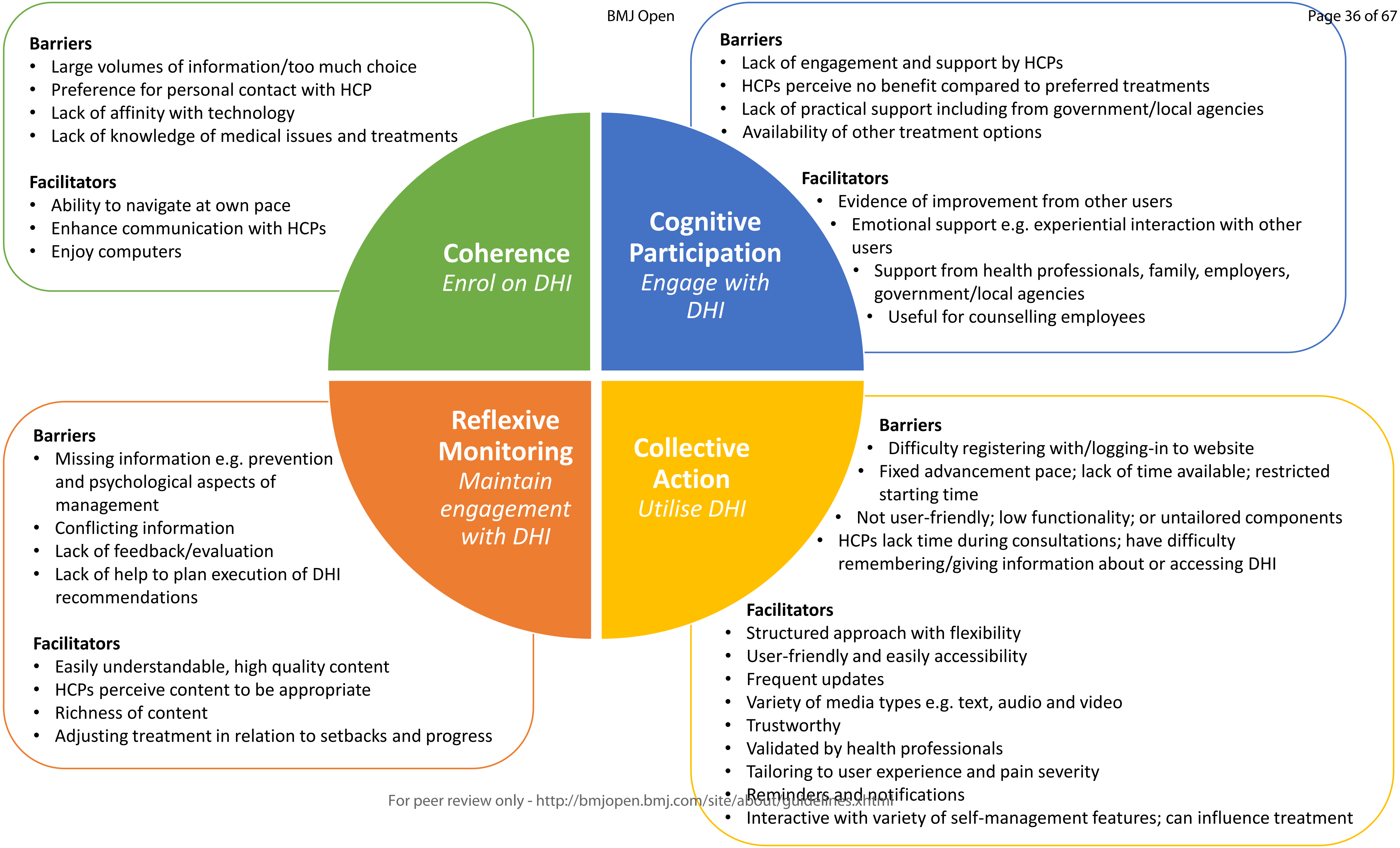
Barriers
• Large volumes of information/too much choice
• Preference for personal contact with HCP
• Lack of affinity with technology
• Lack of knowledge of medical issues and treatments
Facilitators
• Ability to navigate at own pace
• Enhance communication with HCPs
• Enjoy computers
Coherence
Enrol on DHI
Barriers
• Lack of engagement and support by HCPs
• HCPs perceive no benefit compared to preferred treatments
• Lack of practical support including from government/local agencies
• Availability of other treatment options
Facilitators
• Evidence of improvement from other users
• Emotional support e.g. experiential interaction with other users
• Support from health professionals, family, employers, government/local agencies
• Useful for counselling employees
Cognitive Participation
Engage with DHI
Reflexive Monitoring
Maintain engagement with DHI
Barriers
• Missing information e.g. prevention and psychological aspects of management
• Conflicting information
• Lack of feedback/evaluation
• Lack of help to plan execution of DHI recommendations
Facilitators
• Easily understandable, high quality content
• HCPs perceive content to be appropriate
• Richness of content
• Adjusting treatment in relation to setbacks and progress
Collective Action
Utilise DHI
Barriers
• Difficulty registering with/logging-in to website
• Fixed advancement pace; lack of time available; restricted starting time
• Not user-friendly; low functionality; or untailored components
• HCPs lack time during consultations; have difficulty remembering/giving information about or accessing DHI
Facilitators
• Structured approach with flexibility
• User-friendly and easily accessibility
• Frequent updates
• Variety of media types e.g. text, audio and video
• Trustworthy
• Validated by health professionals
• Tailoring to user experience and pain severity
• Reminders and notifications
• Interactive with variety of self-management features; can influence treatment

**Supplementary File 1:** Search details

## MEDLINE - search details

Ovid MEDLINE(R) 1946 to March Week 1 2016

| | |
|---|---|
| 1 | exp back pain/(back pain$ or lumbago or back ache$ or backache$ or (lumbar adj2 pain$) or (spin$ adj2 pain$)).ti,ab,kw,kf. |
| 2 | computer peripherals/ or computer storage devices/ or computer terminals/ or modems/ or microcomputers/ or computers, handheld/ or minicomputers/ or attitude to computers/ or computers/ or computer systems/ or medical informatics/ or medical informatics applications/ or educational technology/ or audiovisual aids/ or telecommunications/ or multimedia/ or computer-assisted instruction/ or user-computer interface/ or hypermedia/ or video games/ or electronic health records/ or social networking/ or (computer$ or microcomputer$ or pc or pcs or mac or macs or internet or www or web or website$ or webpage$ or local area network$).ti,ab,kf. or software.ti,ab,kf. or (cellular phone$ or cellular telephone$ or mobile$ or cell phone$ or cell telephone$ or smartphone$ or smart-phone$ or smart-telephone$).ti,ab,kf. or (handset$ or hand-set$ or wireless or wire-less or wifi or wi-fi or gps or global positioning system$ or bluetooth or text messag$ or texting or sms or short messag$ or multimedia messag$ or multi-media messag$ or mms or instant messag$ or social media$ or facebook or twitter or webcast$ or webinar$ or podcast$ or wiki or wikis or app or apps or android$ or blackberr$ or apple$ or ios or iphone$ or ipad$ or s40 or symbian$ or windows).ti,ab,kf. or ((electronic$ or digital$ or device$) adj2 tablet$).ti,ab,kf. or (video$ or dvd or dvds).ti,ab,kf. or (youtube or you tube or vimeo).ti,ab,kf. or (online or on line or interactive).ti,ab,kf. or (chat room$ or chatroom$).ti,ab,kf. or (blog$1 or web-log$1 or weblog$1).ti,ab,kf. or (bulletin board$ or bulletinboard$ or messageboard$ or message board$).ti,ab,kf. or (ehealth or e-health or mhealth or m-health).ti,ab,kf. or exp telemedicine/ or mobile applications/ or (pda or pdas or personal digital).ti,ab,kf. or device-based.ti,ab,kf. or (email$ or e-mail$ or electronic mail$).ti,ab,kw,kf. |
| 3 | 1 and 2 |
| 4 | limit 3 to yr="2000 -Current" |

Updated searches: 1) October 21 2016 (not shown), 2) December 18 2018 (below):

| | |
|---|---|
| 1 | exp back pain/(back pain$ or lumbago or back ache$ or backache$ or (lumbar adj2 pain$) or (spin$ adj2 pain$)).ti,ab,kw,kf. |
| 2 | computer peripherals/ or computer storage devices/ or computer terminals/ or modems/ or microcomputers/ or computers, handheld/ or minicomputers/ or attitude to computers/ or computers/ or computer systems/ or medical informatics/ or medical informatics applications/ or educational technology/ or audiovisual aids/ or telecommunications/ or multimedia/ or computer-assisted instruction/ or user-computer interface/ or hypermedia/ or video games/ or electronic health records/ or social networking/ |
| 3 | (computer$ or microcomputer$ or pc or pcs or mac or macs or internet or www or web or website$ or webpage$ or local area network$).ti,ab,kf. or software.ti,ab,kf. or (cellular phone$ or cellular telephone$ or mobile$ or cell phone$ or cell telephone$ or smartphone$ or smart-phone$ or smart-telephone$).ti,ab,kf. or (handset$ or hand-set$ or wireless or wire-less or wifi or wi-fi or gps or global positioning system$ or bluetooth or text messag$ or texting or sms or short messag$ or multimedia messag$ or multi-media messag$ or mms or instant messag$ or social media$ or facebook or twitter or webcast$ or webinar$ or podcast$ or wiki or wikis or app or apps or android$ or blackberr$ or apple$ or ios or iphone$ or ipad$ or s40 or symbian$ or windows).ti,ab,kf. or ((electronic$ or digital$ or device$) adj2 tablet$).ti,ab,kf. or (video$ or dvd or dvds).ti,ab,kf. or (youtube or you tube or vimeo).ti,ab,kf. or (online or on line or interactive).ti,ab,kf. or (chat room$ or chatroom$).ti,ab,kf. or (blog$1 or web-log$1 or weblog$1).ti,ab,kf. or (bulletin board$ or bulletinboard$ or messageboard$ or message board$).ti,ab,kf. or (ehealth or e-health or mhealth or m-health).ti,ab,kf. or exp telemedicine/ or mobile applications/ or (pda or pdas or personal digital).ti,ab,kf. or device-based.ti,ab,kf. or (email$ or e-mail$ or electronic mail$).ti,ab,kw,kf. |
| 4 | 1 and (2 or 3) |
| 5 | limit 4 to yr="2000 -Current" |
| 6 | 5 and (201610* or 201611* or 2017* or 2018*).ed. |

## Embase - search details

For peer review only

Ovid Embase (R) 1974 to 2016 March 18

| | |
|---|---|
| 1 | exp backache/ or (spinal pain$ or back pain$ or lumbago or back ache$ or backache$ or (lumbar adj2 pain$) or (spin$ adj2 pain$)).ti,ab,kw. |
| 2 | (exp backache/th or exp backache/pc or exp backache/rh or exp *backache/) not exp backache/su |
| 3 | exp communication protocol/ or computer assisted therapy/ or e-mail/ or human computer interaction/ or information technology/ or interactive voice response system/ or internet/ or mass communication/ or medical informatics/ or medical technology/ or mobile application/ or mobile phone/ or social media/ or exp telecommunication/ or exp telehealth/ or telephone/ or text messaging/ or webcast/ or wireless communication/ |
| 4 | computer storage device/ or computer terminal/ or microcomputer/ or minicomputer/ or attitude to computers/ or computer/ or computer system/ or medical information system/ or educational technology/ or audiovisual aid/ or exp multimedia/ or computer interface/ or hypermedia/ or electronic medical record/ or social networking/ |
| 5 | (computer$ or microcomputer$ or pc or pcs or mac or macs or internet or www or web or website$ or webpage$ or local area network$ or software or (cellular phone$ or cellular telephone$ or mobile$ or cell phone$ or cell telephone$ or smartphone$ or smart-phone$ or smart-telephone$) or (handset$ or hand-set$ or wireless or wire-less or wifi or wi-fi or gps or global positioning system$ or bluetooth or text messag$ or texting or sms or short messag$ or multimedia messag$ or multi-media messag$ or mms or instant messag$ or social media$ or facebook or twitter or webcast$ or webinar$ or podcast$ or wiki or wikis or app or apps or android$ or blackberr$ or apple$ or ios or iphone$ or ipad$ or s40 or symbian$ or windows) or ((electronic$ or digital$ or device$) adj2 tablet$) or (video$ or dvd or dvds) or (youtube or you tube or vimeo) or (online or on line or interactive) or (chat room$ or chatroom$) or (blog$1 or web-log$1 or weblog$1) or (bulletin board$ or bulletinboard$ or messageboard$ or message board$) or (ehealth or e-health or mhealth or m-health) or (app or apps) or (pda or pdas or personal digital) or device-based or (email$ or e-mail$ or electronic mail$)).ti,ab,kw. |
| 6 | 2 and 3 |
| 7 | limit 6 to yr="2000 -Current" |
| 8 | 1 and (3 or 4 or 5) |
| 9 | limit 8 to yr="2000 -Current" |
| 10 | 9 not 7 |

Updated searches: 1) October 21 2016 (not shown), 2) December 18 2018 (below):

| | |
|---|---|
| 1 | exp backache/ or (spinal pain$ or back pain$ or lumbago or back ache$ or backache$ or (lumbar adj2 pain$) or (spin$ adj2 pain$)).ti,ab,kw. |
| 2 | exp communication protocol/ or computer assisted therapy/ or e-mail/ or human computer interaction/ or information technology/ or interactive voice response system/ or internet/ or mass communication/ or medical informatics/ or medical technology/ or mobile application/ or mobile phone/ or social media/ or exp telecommunication/ or exp telehealth/ or telephone/ or text messaging/ or webcast/ or wireless communication/ |
| 3 | computer storage device/ or computer terminal/ or microcomputer/ or minicomputer/ or attitude to computers/ or computer/ or computer system/ or medical information system/ or educational technology/ or audiovisual aid/ or exp multimedia/ or computer interface/ or hypermedia/ or electronic medical record/ or social networking/ |
| 4 | (computer$ or microcomputer$ or pc or pcs or mac or macs or internet or www or web or website$ or webpage$ or local area network$ or software or (cellular phone$ or cellular telephone$ or mobile$ or cell phone$ or cell telephone$ or smartphone$ or smart-phone$ or smart-telephone$) or (handset$ or hand-set$ or wireless or wire-less or wifi or wi-fi or gps or global positioning system$ or bluetooth or text messag$ or texting or sms or short messag$ or multimedia messag$ or multi-media messag$ or mms or instant messag$ or social media$ or facebook or twitter or webcast$ or webinar$ or podcast$ or wiki or wikis or app or apps or android$ or blackberr$ or apple$ or ios or iphone$ or ipad$ or s40 or symbian$ or windows) or ((electronic$ or digital$ or device$) adj2 tablet$) or (video$ or dvd or dvds) or (youtube or you tube or vimeo) or (online or on line or interactive) or (chat room$ or chatroom$) or (blog$1 or web-log$1 or weblog$1) or (bulletin board$ or bulletinboard$ or messageboard$ or message board$) or (ehealth or e-health or mhealth or m-health) or (app or apps) or (pda or pdas or personal digital) or device-based or (email$ or e-mail$ or electronic mail$)).ti,ab,kw. |
| 5 | 1 and (2 or 3 or 4) |
| 6 | limit 5 to yr="2000 -Current" |

For peer review only

| | |
|---|---|
| 7 | limit 5 to yr="2016 -Current" |

**CINAHL - search details**

CINAHL (R) March 2016 through EBSCOhost

| | | |
|---|---|---|
| S6 | S1 AND S4 | |
| S5 | S1 AND S4 | |
| S4 | S2 OR S3 | |
| S3 | TI (computer* OR microcomputer* OR pc OR pcs OR mac OR macs OR internet OR www OR web OR website* OR webpage* OR "local area network*" OR software OR "cellular phone*" OR "cellular telephone*" OR mobile* OR "cell phone"* OR "cell telephone*" OR smartphone* OR smart-phone* OR smart-telephone* OR handset* OR hand-set* OR wireless OR wire-less OR wifi OR wi-fi OR gps OR "global positioning system*" OR bluetooth OR "text messag*" OR texting OR sms OR "short messag*" OR "multimedia messag*" OR "multi-media messag*" OR mms OR "instant messag*" OR "social media*" OR facebook OR twitter OR webcast* OR webinar* OR podcast* OR wiki OR wikis OR app OR apps OR android* OR blackberr* OR apple* OR ios OR iphone* OR ipad* OR s40 OR symbian* OR windows OR ((electronic* OR digital* OR device*) W2 tablet*) OR video* OR dvd OR dvds OR youtube OR "you tube" OR vimeo OR online OR "on line" or interactive OR "chat room*" OR chatroom* OR blog OR blogs OR web-log OR web-logs OR weblog OR weblogs OR "bulletin board*" OR bulletinboard$ OR messageboard$ OR "message board*" OR ehealth OR e-health OR mhealth OR m-health OR app OR apps OR pda OR pdas OR "personal digital" OR "device-based" OR email* OR e-mail* OR "electronic mail*") OR AB (computer* OR microcomputer* OR pc OR pcs OR mac OR macs OR internet OR www OR web OR website* OR webpage* OR "local area network*" OR software OR "cellular phone*" OR "cellular telephone*" OR mobile* OR "cell phone"* OR "cell telephone*" OR smartphone* OR smart-phone* OR smart-telephone* OR handset* OR hand-set* OR wireless OR wire-less OR wifi OR wi-fi OR gps OR "global positioning system*" OR bluetooth OR "text messag*" OR texting OR sms OR "short messag*" OR "multimedia messag*" OR "multi-media messag*" OR mms OR "instant messag*" OR "social media*" OR facebook OR twitter OR webcast* OR webinar* OR podcast* OR wiki OR wikis OR app OR apps OR android* OR blackberr* OR apple* OR ios OR iphone* OR ipad* OR s40 OR symbian* OR windows OR ((electronic* OR digital* OR device*) W2 tablet*) OR video* OR dvd OR dvds OR youtube OR "you tube" OR vimeo OR online OR "on line" or interactive OR "chat room*" OR chatroom* OR blog OR blogs OR web-log OR web-logs OR weblog OR weblogs OR "bulletin board*" OR bulletinboard$ OR messageboard$ OR "message board*" OR ehealth OR e-health OR mhealth OR m-health OR app OR apps OR pda OR pdas OR "personal digital" OR "device-based" OR email* OR e-mail* OR "electronic mail*")) | |
| S2 | (MH "Computer peripherals") OR (MH "Computer storage devices") OR (MH "Computer terminals") OR (MH | |

For peer review only

| | | |
|---|---|---|
| | "Microcomputers") OR (MH "Computers, hand-held") OR (MH "Attitude to computers") OR (MH "Computer systems") OR (MH "Medical informatics") OR (MH "Educational technology") OR (MH "Audiovisuals") OR (MH "Audiorecording") OR (MH "Videorecording") OR (MH "Multimedia") OR (MH "Computer Environment") OR (MH "Computer Assisted Instruction") OR (MH "Hypermedia") OR (MH "Video games") OR (MH "Mobile applications") OR (MH "Patient record systems") OR (MH "Computerized patient record") OR (MH "") OR (MH "Computer communication networks+") OR (MH "Telecommunications") OR (MH "Electronic Bulletin Boards") OR (MH "Electronic Mail") OR (MH "Instant Messaging") OR (MH "Interactive Voice Response Systems") OR (MH "Text Messaging") OR (MH "Cellular Phone") OR (MH "Telephone") OR (MH "Internet+") OR (MH "Remote Consultation") OR (MH "Telemedicine") OR (MH "Telehealth") OR (MH "Telenursing") OR (MH "Smartphone") OR (MH "User-Computer Interface+") | |
| S1 | (MH "Back Pain+") OR TI ("spinal pain* " OR "back pain*" OR lumbago OR "back acke*" OR backache OR (lumbar W2 pain*) OR (spin* W2 pain*)) OR AB ("spinal pain* " OR "back pain*" OR lumbago OR "back acke*" OR backache OR (lumbar W2 pain*) OR (spin* W2 pain*)) | |

Updated searches: 1) October 21 2016, 2) December 18 2018

**Cochrane Library - search details** (Through Wiley Online Library)

- Cochrane Database of Systematic Review (CDSR)
- Database of Reviews of Systematic Reviews (DARE, discontinued)
- Central Cochrane Register of Controlled Trials (CENTRAL)
- ‘Method studies’
- ‘Technology assessments’
- ‘Economic evaluations’

| | |
|---|---|
| #1 | (spinal next pain* or back next pain* or lumbago or back next acke* or backache or (lumbar near/2 pain*) or (spin* near/2 pain*)):ti,ab,kw |
| #2 | (computer* or microcomputer* or "pc" or "pcs" or "mac" or "macs" or "internet" or "www" or "web" or website* or webpage* or local next area next network* or "software" or cellular next phone* or cellular next telephone* or mobile* or cell next phone* or cell next telephone* or smartphone* or smart-phone* or smart-telephone* or handset* or hand-set* or "wireless" or "wire-less" or "wifi" or "wi-fi" or "gps" or global next positioning next system* or "bluetooth" or text next messag* or "texting" or "sms" or short next messag* or multimedia next messag* or multi-media next messag* or "mms" or instant next messag* or social next media* or "facebook" or "twitter" or webcast* or webinar* or podcast* or "wiki" or "wikis" or "app" or "apps" or android* or blackberr* or apple* or "ios" or iphone* or ipad* or "s40" or symbian* or "windows" or ((electronic* or digital* or device*) near/2 tablet*) or video* or "dvd" or "dvds" or "youtube" or "you tube" or "vimeo" or "online" or "on line" or "interactive" or chat next room* or chatroom* or "blog" or "blogs" or "web-log" or "web-logs" or "weblog" or "weblogs" or bulletin next board* or bulletinboard* or messageboard* or message next board* or "ehealth" or "e-health" or "mhealth" or "m-health" or "app" or "apps" |

For peer review only

| | |
|---|---|
| | or "pda" or "pdas" or "personal digital" or "device-based" or email* or e-mail* or electronic next mail*):ti,ab,kw |
| #3 | #1 and #2 |

Updated searches: 1) October 21 2016 (not shown), 2) December 18 2018 (below):

| | |
|---|---|
| #1 | (spinal next pain* or back next pain* or lumbago or back next acke* or backache or (lumbar near/2 pain*) or (spin* near/2 pain*)):ti,ab,kw |
| #2 | (computer* or microcomputer* or "pc" or "pcs" or "mac" or "macs" or "internet" or "www" or "web" or website* or webpage* or local next area next network* or "software" or cellular next phone* or cellular next telephone* or mobile* or cell next phone* or cell next telephone* or smartphone* or smart-phone* or smart-telephone* or handset* or hand-set* or "wireless" or "wire-less" or "wifi" or "wi-fi" or "gps" or global next positioning next system* or "bluetooth" or text next messag* or "texting" or "sms" or short next messag* or multimedia next messag* or multi-media next messag* or "mms" or instant next messag* or social next media* or "facebook" or "twitter" or webcast* or webinar* or podcast* or "wiki" or "wikis" or "app" or "apps" or android* or blackberr* or apple* or "ios" or iphone* or ipad* or "s40" or symbian* or "windows" or ((electronic* or digital* or device*) near/2 tablet*) or video* or "dvd" or "dvds" or "youtube" or "you tube" or "vimeo" or "online" or "on line" or "interactive" or chat next room* or chatroom* or "blog" or "blogs" or "web-log" or "web-logs" or "weblog" or "weblogs" or bulletin next board* or bulletinboard* or messageboard* or message next board* or "ehealth" or "e-health" or "mhealth" or "m-health" or "app" or "apps" or "pda" or "pdas" or "personal digital" or "device-based" or email* or e-mail* or electronic next mail*):ti,ab,kw |
| #3 | #1 and #2 |
| | With Publication Year from 2016 to 2018, with Cochrane Library publication date from Jan 2016 to Dec 2018, in Trials |

| | |
|---|---|
| #1 | (spinal next pain* or back next pain* or lumbago or back next acke* or backache or (lumbar near/2 pain*) or (spin* near/2 pain*)):ti,ab,kw |
| #2 | (computer* or microcomputer* or "pc" or "pcs" or "mac" or "macs" or "internet" or "www" or "web" or website* or webpage* or local next area next network* or "software" or cellular next phone* or cellular next telephone* or mobile* or cell next phone* or cell next telephone* or smartphone* or smart-phone* or smart-telephone* or handset* or hand-set* or "wireless" or "wire-less" or "wifi" or "wi-fi" or "gps" or global next positioning next system* or "bluetooth" or text next messag* or "texting" or "sms" or short next messag* or multimedia next messag* or multi-media next messag* or "mms" or instant next messag* or social next media* or "facebook" or "twitter" or webcast* or webinar* or podcast* or "wiki" or "wikis" or "app" or "apps" or android* or blackberr* or apple* or "ios" or iphone* or ipad* or "s40" or symbian* or "windows" or ((electronic* or digital* or device*) near/2 tablet*) or video* or "dvd" or "dvds" or "youtube" or "you tube" or "vimeo" or "online" or "on line" or "interactive" or chat next room* or chatroom* or "blog" or "blogs" or "web-log" or "web-logs" or "weblog" or "weblogs" or bulletin next board* or bulletinboard* or messageboard* or message next board* or "ehealth" or "e-health" or "mhealth" or "m-health" or "app" or "apps" or "pda" or "pdas" or "personal digital" or "device-based" or email* or e-mail* or electronic next mail*):ti,ab,kw |
| #3 | #1 and #2 |

For peer review only

| | |
|---|---|
| | With Cochrane Library publication date from Jan 2016 to Dec 2018, in Cochrane Reviews and Cochrane Protocols |

**PsycINFO - search details**

Ovid PsycINFO (R) 1987 to March Week 4 2016

| | |
|---|---|
| 1 | exp back pain/ or (spinal pain$ or back pain$ or lumbago or back ache$ or backache$ or (lumbar adj2 pain$) or (spin$ adj2 pain$)).ti,ab,id. |
| 2 | exp Human Computer Interaction/ or Computer Peripheral Devices/ or Computer Software/ or Human Machine Systems/ or exp Electronic Communication/ or exp Computers/ or exp Mobile Devices/ or exp Internet/ or exp Computer Applications/ or Computer Attitudes/ or Information Technology/ or exp AUDIOVISUAL INSTRUCTION/ or exp AUDIOVISUAL COMMUNICATIONS MEDIA/ or exp EDUCATIONAL AUDIOVISUAL AIDS/ or Telecommunications Media/ or Multimedia/ or exp Social media/ or exp Telephone systems/ or Telemedicine/ or exp Websites/ or (computer$ or microcomputer$ or pc or pcs or mac or macs or internet or www or web or website$ or webpage$ or local area network$ or software or cellular phone$ or cellular telephone$ or mobile$ or cell phone$ or cell telephone$ or smartphone$ or smart-phone$ or smart-telephone$ or handset$ or hand-set$ or wireless or wire-less or wifi or wi-fi or gps or global positioning system$ or bluetooth or text messag$ or texting or sms or short messag$ or multimedia messag$ or multi-media messag$ or mms or instant messag$ or social media$ or facebook or twitter or webcast$ or webinar$ or podcast$ or wiki or wikis or app or apps or android$ or blackberr$ or apple$ or ios or iphone$ or ipad$ or s40 or symbian$ or windows or ((electronic$ or digital$ or device$) adj2 tablet$) or video$ or dvd or dvds or youtube or you tube or vimeo or online or on line or interactive or chat room$ or chatroom$ or blog$1 or web-log$1 or weblog$1 or bulletin board$ or bulletinboard$ or messageboard$ or message board$ or ehealth or e-health or mhealth or m-health or app or apps or pda or pdas or personal digital or device-based or email$ or e-mail$ or electronic mail$).ti,ab,id. |
| 3 | 1 and 2 |
| 4 | limit 3 to yr="2000 -Current" |

Updated searches: 1) October 21 2016 (not shown), 2) December 18 2018 (below):

| | |
|---|---|
| 1 | exp back pain/ or (spinal pain$ or back pain$ or lumbago or back ache$ or backache$ or (lumbar adj2 pain$) or (spin$ adj2 pain$)).ti,ab,id. |
| 2 | exp Human Computer Interaction/ or Computer Peripheral Devices/ or Computer Software/ or Human Machine Systems/ or exp Electronic Communication/ or exp Computers/ or exp Mobile Devices/ or exp Internet/ or exp Computer Applications/ or Computer Attitudes/ or Information Technology/ or exp AUDIOVISUAL INSTRUCTION/ or exp AUDIOVISUAL COMMUNICATIONS MEDIA/ or exp EDUCATIONAL AUDIOVISUAL AIDS/ or Telecommunications Media/ or Multimedia/ or exp Social media/ or exp Telephone systems/ or Telemedicine/ or exp Websites/ |
| 3 | (computer$ or microcomputer$ or pc or pcs or mac or macs or internet or www or web or website$ or webpage$ or local area network$ or software or cellular phone$ or cellular telephone$ or mobile$ or cell phone$ or cell telephone$ or smartphone$ or smart-phone$ or smart-telephone$ or handset$ or hand-set$ or wireless or wire-less or wifi or wi-fi or gps or global positioning system$ or bluetooth or text messag$ or texting or sms or short messag$ or multimedia messag$ or multi-media messag$ or mms or instant messag$ or social media$ or facebook or twitter or webcast$ or webinar$ or podcast$ or wiki or wikis or app or apps or android$ or blackberr$ or apple$ or ios or iphone$ or ipad$ or s40 or symbian$ or windows or ((electronic$ or digital$ or device$) adj2 tablet$) or video$ or dvd or dvds or youtube or you tube or vimeo or online or on line or interactive or chat room$ or chatroom$ or blog$1 or web-log$1 or weblog$1 or bulletin board$ or bulletinboard$ or messageboard$ or message board$ or ehealth or e-health or mhealth or m-health or app or apps or pda or pdas or personal digital or device-based or email$ or e-mail$ or electronic mail$).ti,ab,id. |
| 4 | 1 and (2 or 3) |
| 5 | limit 4 to yr="2000 -Current" |
| 6 | 5 and (20161* or 2017* or 2018* or 2019*).up. |

**DoPHER - search details**

Database of Promoting Health Effectiveness Reviews
Focussed coverage of systematic and non-systematic reviews of effectiveness in health promotion and public health worldwide (3700).

Search date 11.04.2016

| | |
|---|---|
| 1 | Freetext (Year): >1999 |
| 2 | Freetext (All but Authors): “spinal pain” OR “back pain” OR “spinal pains” OR “back pains” OR lumbago OR “back ache” OR “back aches” OR “backache*” |
| 3 | 1 AND 2 |

Updated searches: 1) October 21 2016, 2) December 18 2018

**TROPHI - search details**

Trials Register of Promoting Health Interventions
Focussed coverage of trials of interventions in health promotion and public health worldwide. It covers both randomised and non-randomised controlled trials and currently contains details of over 7,750 trials.

Search date 11.04.2016

| | |
|---|---|
| 5 | Freetext (All but Authors): “spinal pain” OR “back pain” OR “spinal pains” OR “back pains” OR lumbago OR “back ache” OR “back aches” OR “backache*” |
| 6 | Freetext (Year): >1999 |
| 7 | 5 AND 6 |

Updated searches: 1) October 21 2016, 2) December 18 2018

**Web of Science - search details**
(Thomson Reuters)

Databases selected:

- Science Citation Index (SCI Expanded)
- Social Science Citation Index (SSCI)
- Conference Proceedings Citation Index – Science (CPCI-S)
- Conference Proceedings Citation Index – Social Science (SPCI-SSH)

Search date 6.4.2016

| | |
|---|---|
| #3 | #2 AND #1<br>*Indexes=SCI-EXPANDED, SSCI, CPCI-S, CPCI-SSH Timespan=2000-2016* |

For peer review only

| | |
|---|---|
| #2 | **TOPIC:** (computer$ OR microcomputer* OR pc OR pcs OR mac OR macs OR internet OR www OR web OR website* OR webpage* OR "local area network*" OR software OR "cellular phone*" OR "cellular telephone*" OR mobile* OR "cell phone*" OR "cell telephone*" OR smartphone* OR smart-phone* OR smart-telephone* OR handset* OR hand-set* OR wireless OR wire-less OR wifi OR wi-fi OR gps OR "global positioning system*" OR bluetooth OR "text messag*" OR texting OR sms OR "short messag*" OR "multimedia messag*" OR "multi-media messag*" OR mms OR "instant messag*" OR "social media*" OR facebook OR twitter OR webcast* OR webinar* OR podcast* OR wiki OR wikis OR app OR apps OR android* OR blackberr* OR apple* OR ios OR iphone* OR ipad* OR s40 OR symbian* OR windows OR ((electronic* OR digital* OR device*) NEAR/2 tablet*) OR video* OR dvd OR dvds OR youtube OR "you tube" OR vimeo OR online OR "on line" OR interactive OR "chat room*" OR chatroom* OR blog OR blogs OR web-log OR web-logs OR weblog OR weblogs OR "bulletin board*" OR bulletinboard* OR messageboard* OR "message board*" OR ehealth OR e-health OR mhealth OR m-health OR pda OR pdas OR "personal digital" OR "device-based" OR email* OR e-mail* OR "electronic mail*")<br>*Indexes=SCI-EXPANDED, SSCI, CPCI-S, CPCI-SSH Timespan=2000-2016* |
| #1 | **TOPIC:** ("spinal pain*" OR "back pain*" OR lumbago OR "back ache*" OR backache* OR lumbar NEAR/2 pain* OR spin* NEAR/2 pain*)<br>*Indexes=SCI-EXPANDED, SSCI, CPCI-S, CPCI-SSH Timespan=2000-2016* |

Updated searches: 1) October 21 2016, 2) December 18 2018

**OT Seeker - search details**

Occupational therapy systematic evaluation of evidence.
http://www.otseeker.com/Search/BasicSearch.aspx

| | |
|---|---|
| 1 | back pain AND (internet OR web) |

Updated searches: 1) October 21 2016, 2) December 18 2018

For peer review only

**Supplementary File 2:** Consensus summary of quality appraisal as per the 32-item Consolidated Criteria for Reporting Qualitative Research (COREQ) checklist (Booth et al., 2014; Tong et al., 2007) and comprehensiveness of reporting.

| No | Item | Guide questions | de Jong et al., 2009 | Caiata Zufferey & Schulz, 2009 | Schulz et al., 2010 | Nordin et al., 2017 | Rabbi et al., 2018 | Number of articles reporting each item (%) |
|---|---|---|---|---|---|---|---|---|
| **Domain 1: Research team and reflexivity** | | | | | | | | |
| **Personal characteristics** | | | | | | | | |
| **1** | Interviewer/facilitator | Which author/s conducted the interview or focus group? | N/R | N/R | N/R | Principal author | N/R | 1 (20%) |
| **2** | Credentials | What were the researcher's credentials? E.g. PhD, MD | N/R | N/R | N/R | PhD | PhD, PhD and MD | 2 (40%) |
| **3** | Occupation | What was their occupation at the time of the study? | N/R | N/R | N/R | N/R | N/R | 0 (0%) |
| **4** | Gender | Was the researcher male or female? | N/R | N/R | N/R | Female | N/R | 1 (20%) |
| **5** | Experience and training | What experience or training did the researcher have? | N/R | N/R | N/R | N/R | N/R | 0 (0%) |
| **Relationship with participants** | | | | | | | | |
| **6** | Relationship established | Was a relationship established prior to study commencement? | N/R | N/R | N/R | Participants had participated in the RCT, of which the qualitative study was a later part | N/R | 1 (20%) |
| **7** | Participant knowledge of the interviewer | What did the participants know about the researcher? e.g. | N/R | N/R | N/R | N/R | N/R | 0 (0%) |

For peer review only

| | | | | | | | | |
|---|---|---|---|---|---|---|---|---|
| | | personal goals, reasons for doing the research | | | | | | |
| **8** | Interviewer characteristics | What characteristics were reported about the interviewer/facilitator? e.g. Bias, assumptions, reasons and interests in the research topic | N/R | N/R | N/R | N/R | N/R | 0 (0%) |
| **Domain 2: Study design** | | | | | | | | |
| **Theoretical framework** | | | | | | | | |
| **9** | Methodological orientation and theory | What methodological orientation was stated to underpin the study? e.g. grounded theory, discourse analysis, ethnography, phenomenology, content analysis | N/R | Grounded theory | N/R | Content Analysis | N/R | 2 (40%) |
| **Participant selection** | | | | | | | | |
| **10** | Sampling | How were participants selected? e.g. purposive, convenience, consecutive, snowball | Convenience | Convenience | Convenience | Convenience | Convenience | 5 (100%) |
| **11** | Method of approach | How were participants approached? e.g. face-to-face, telephone, mail, email | N/R | Email | Email | First approach not clear, but once given oral consent contacted by telephone | Method of sending invitations not clear. If eligible face-to-face meeting | 4 (80%) |
| **12** | Sample size | How many participants were in the study? | 11 OPs who recruited; 8 OPs who did not recruit & 9 employees | 18 | 18 | 19 | 10 | 5 (100%) |

For peer review only

| | | | | | | | | |
|---|---|---|---|---|---|---|---|---|
| **13** | Non-participation | How many people refused to participate or dropped out? Reasons? | 7 OPs who did not recruit; 15 employees. Reasons - no time, insufficient use of program, problems with recalling experiences | 238 approached to participate; 32 responded; 14 of these did not participate – reasons not stated | N/R | 3 – reasons not stated | None | 4 (80%) |
| **Setting** | | | | | | | | |
| **14** | Setting of data collection | Where was the data collected? e.g. home, clinic, workplace | Telephone interviews | Home or University | Home or University | Health Care Centres, County City Buildings, Participant's home | Web-based exit survey | 5 (100%) |
| **15** | Presence of non-participants | Was anyone else present besides the participants and researchers? | N/R | N/R | N/R | N/R | N/R | 0 (0%) |
| **16** | Description of sample | What are the important characteristics of the sample? e.g. demographic data, date | Not stated for OPs; Employees 67% male; 40-50 years; 75% LBP; white & blue-collar workers; varying educational levels; varying sickness absence levels due to LBP | 9 females, 9 males; 28-72 years; chronic LBP for 1-30 years; mix of diagnoses including 8 with no clear diagnosis; all had at least secondary school education (5 had degree); | 9 females, 9 males; 28-72 years; chronic LBP 1-30 years ; mixed diagnoses, varied level of education and frequency of website use | 15 females, 4 males; mean age 45; MSK pain for average 7.5 years; most at least secondary education; majority working. | 7 females, 3 males; 31-60 years; chronic LBP 5-33 years duration; mixed diagnoses. | 5 (100%) |

For peer review only

| | | | | | | | | |
|---|---|---|---|---|---|---|---|---|
| | | | 7weeks-6 months | range of website use amongst participants | | | | |
| **Data collection** | | | | | | | | |
| **17** | Interview guide | Were questions, prompts, guides provided by the authors? Was it pilot tested? | Topic guides used. Pilot tested | No questions, prompts or guides provided; Piloting not reported | No questions, prompts or guides provided; Piloting not reported | No questions, prompts or guides provided; Piloting not reported | Open-ended question in web survey provided. Piloting not reported | 2 (40%) |
| **18** | Repeat interviews | Were repeat interviews carried out? If yes, how many? | N/R | N/R | N/R | N/R | N/R | 0 (0%) |
| **19** | Audio/visual recording | Did the research use audio or visual recording to collect the data? | Audio recorded | Not specifically stated "Recorded" and transcribed verbatim | Audio recorded | Audio recorded | No – used free text web survey | 5 (100%) |
| **20** | Field notes | Were field notes made during and/or after the interview or focus group? | N/R | N/R | N/R | N/R | N/R | 0 (0%) |
| **21** | Duration | What was the duration of the interviews or focus group? | Approx. 30 minutes | Approx. 45 minutes | Approx. 45 minutes | 31 – 56 minutes. Mean 48 minutes | N/R | 4 (80%) |
| **22** | Data saturation | Was data saturation discussed? | Yes | Yes | N/R | N/R | N/R | 2 (40%) |
| **23** | Transcripts returned | Were transcripts returned to participants for comment and/or correction? | N/R | N/R | N/R | N/R | N/R | 0 (0%) |
| **Domain 3: Analysis and findings** | | | | | | | | |

| | | | | | | | | |
|---|---|---|---|---|---|---|---|---|
| **Data analysis** | | | | | | | | |
| **24** | Number of data coders | How many data coders coded the data? | N/R | N/R | N/R | 4 | N/R | 1 (20%) |
| **25** | Description of coding tree | Did authors provide a description of the coding tree? | N/R | N/R | N/R | Yes | N/R | 1 (20%) |
| **26** | Derivation of themes | Were themes identified in advance or derived from the data? | Derived from data | Derived from data | Essentially inductive | Derived from data | Derived from data | 5 (100%) |
| **27** | Software | What software, if applicable, was used to manage the data? | Excel | ATLAS.ti | ATLAS.ti | Open Code | N/R | 4 (80%) |
| **28** | Participant checking | Did participants provide feedback on the findings? | N/R | N/R | N/R | N/R | N/R | 0 (0%) |
| **Reporting** | | | | | | | | |
| **29** | Quotations presented | Were participant quotations presented to illustrate the themes / findings? Was each quotation identified? e.g. participant number | Few direct quotes; only identified as either OP or employee | Yes - identified by gender, age & occupation | Yes - identified by gender, age & occupation | Yes – identified by participant number and gender | Yes – identified by participant number | 5 (100%) |
| **30** | Data and findings consistent | Was there consistency between the data presented and the findings? | A little unclear – little qualitative data presented | Yes | Yes | Yes | Yes | 5 (100%) |
| **31** | Clarity of major themes | Were major themes clearly presented in the findings? | Yes | Yes | Yes | Yes | Yes | 5 (100%) |
| **32** | Clarity of minor themes | Is there a description of diverse cases or discussion of minor themes? | Not clear | Range of themes presented but not clear what is major/minor | Range of themes presented but not clear what is major/minor | Yes | Yes | 2 (40%) |

For peer review only

| | | | | | | |
|---|---|---|---|---|---|---|
| **TOTAL, number (%)** | 14 (44%) | 15 (47%) | 12 (38%) | 21 (67%) | 14 (44%) | |
| **N/R: not reported; OPs: occupational physicians; LBP: low back pain** | | | | | | |

## References

Booth A, Hannes K, Harden A, Noyes J, Harris J, Tong A. COREQ (Consolidated Criteria for Reporting Qualitative Studies). In: Moher D, Altman DG, Schulz KF, Simera I, Wager E, editors. Guidelines for reporting health research: A user's manual. Oxford: Wiley; 2014. p. 214-26.

Caiata Zufferey M, Schulz PJ. Self-management of chronic low back pain: an exploration of the impact of a patient-centered website. Patient education and counseling. 2009;77(1):27-32.

de Jong T, Heinrich J, Blatter BM, Anema JR, van der Beek AJ. The feasibility of a web-based counselling program for occupational physicians and employees on sick leave due to back or neck pain. BMC medical informatics and decision making. 2009;9:46.

Nordin C, Michaelson P, Eriksson MK, Gard G. It's About Me: Patients' Experiences of Patient Participation in the Web Behavior Change Program for Activity in Combination With Multimodal Pain Rehabilitation. Journal of medical Internet research. 2017;19(1):e22-e.

Rabbi M, Aung MS, Gay G, Reid MC, Choudhury T. Feasibility and Acceptability of Mobile Phone-Based Auto-Personalized Physical Activity Recommendations for Chronic Pain Self-Management: Pilot Study on Adults. Journal of medical Internet research. 2018;20(10):e10147-e.

Schulz PJ, Rubinelli S, Zufferey MC, Hartung U. Coping with Chronic Lower Back Pain: Designing and Testing the Online Tool ONESELF. Journal of Computer-Mediated Communication. 2010;15(4):625-45.

Tong A, Sainsbury P, Craig J. Consolidated criteria for reporting qualitative research (COREQ): a 32-item checklist for interviews and focus groups. International journal for quality in health care : journal of the International Society for Quality in Health Care. 2007;19(6):349-57.

For peer review only

**Supplementary File 3:** Taxonomy of barriers and facilitators with exemplar quotations

**References**:

[27] Schulz PJ, Rubinelli S, Zufferey MC, Hartung U. Coping with Chronic Lower Back Pain: Designing and Testing the Online Tool ONESELF. Journal of Computer-Mediated Communication. 2010;15(4):625-45.
[28] Caiata Zufferey M, Schulz PJ. Self-management of chronic low back pain: an exploration of the impact of a patient-centered website. Patient education and counseling. 2009;77(1):27-32.
[36] de Jong T, Heinrich J, Blatter BM, Anema JR, van der Beek AJ. The feasibility of a web-based counselling program for occupational physicians and employees on sick leave due to back or neck pain. BMC medical informatics and decision making. 2009;9:46.
[37] Rabbi M, Aung MS, Gay G, Reid MC, Choudhury T. Feasibility and Acceptability of Mobile Phone-Based Auto-Personalized Physical Activity Recommendations for Chronic Pain Self-Management: Pilot Study on Adults. Journal of medical Internet research. 2018;20(10):e10147-e.
[38] Nordin C, Michaelson P, Eriksson MK, Gard G. It's About Me: Patients' Experiences of Patient Participation in the Web Behavior Change Program for Activity in Combination With Multimodal Pain Rehabilitation. Journal of medical Internet research. 2017;19(1):e22-e.

| **Barriers and facilitators for patient uptake and utilisation of digital self-management interventions for LBP** | | | | | |
|---|---|---|---|---|---|
| **Theme** | **Taxonomy** | **Barriers** | **Exemplar quotations** | **Facilitators** | **Exemplar quotations** |
| IT usability and accessibility | Functionality and usability | • Too much choice between functions<br>• Fixed advancement pace<br>• Issues logging into DHI<br>• *Low user-friendliness<br>• *Issues logging into DHI<br>• *Low level of functionality (e.g. registration, navigation, helpdesk) | • Though, the freedom of choice in the Web-BCPA entailed perceptions of restrained patient participation for some informants [38, p4]<br>• Finally, some OPs faced practical obstacles such as log-[illegible] v [illegible] [illegible] [illegible] ,o p5]<br>• Although OPs were generally positive about the user-friendliness and | • Flexible structure and navigation<br>• Conveniently arranged<br>• Variation of media types (text, audio and video)<br>• Reminders and notifications<br>• High user-friendliness<br>• *High user-friendliness | • *I liked this thing about the exercise video a lot because seeing it with the video gives you a lot more. They seem simple, but a lot of times when there are drawings I can't understand them easily, then I don't have the will anymore [28, p29]*<br>• It was enough to open the mailbox for reasons that could be independent of cLBP |

| | | | | | |
|---|---|---|---|---|---|
| | | | design of the program, some felt that further improving user-friendliness (functionality) might [illegible] It should also be easier to register employees in the program [36, p6]<br>• Although, some informants perceived restrained patient participation by the fact t[illegible] being able to select a faster advancement in the program by themselves [36, p5]<br>• A small number of employees either had problems with [illegible] the [illegible] | | to get a reminder of the website and the necessity of self-[illegible] *usually went on the website when I read the newsletter. I read the letter and then I'm there, it's like a conditioned reflex [27, p641]*<br>• *It would be helpful to have reminders and suggestions pop up in the morning or at other chosen times. This could be optional and set by the user [37, p10]*<br>• These effects could be reached thanks to the specificities of the website, th[illegible] multimediality (material was provided in written, audio and video form), usability (the website was easy to use [...]) [28, p31]<br>• They [the users] were positive about the content, user-friendliness and web-based design. They |

For peer review only

For peer review only

| | | | | | |
|---|---|---|---|---|---|
| | | | | | said the information was easy to understand, to the point and conveniently arranged [36, p5]<br>• Finally, almost all OPs were positive about the user-friendliness and design of the program [36, p5] |
| | IT affinity | • Lack of affinity with computers<br>• *Lack of affinity with web-based programmes | • Some OPs had no affinity with the use of a web-based program in general and therefore preferred not to use this method [36, p5]<br>• A small number of employees [illegible] affinity with [illegible] p6] [illegible] | • Enjoying working with a computer | • In addition, some informants stated [illegible] working at the computer, facilitated patient participation in the rehabilitation [38, p6] |
| | Access and convenience | • Not able to choose starting time of DHI<br>• *No access to computer during consultation | • Although, some informants perceived restrained patient participation by the fact that they were not able to choose the starting time of the Web-BCPA course themselves (due to study [illegible] 38, p5] [illegible]<br>• Finally, some OPs faced practical | • Easily accessible with low effort<br>• Accessible at all hours and locations<br>• Accessible even during periods with severe pain symptoms<br>• Ability to take all the time needed | • Patient participation was emphasized by having access to the Web-BCPA on computer or tablet at all hours and locations [38, p5]<br>• The opportunities to work in the Web-BCPA at home were experienced to provide continuity in |

| | | | | | |
|---|---|---|---|---|---|
| | | | obstacles such as [illegible] no access to a computer or the internet in their consulting rooms [36, p5] | | the rehabilitation [38, p5]<br>• These effects could be reached thanks to the specificities of the website, that is its [illegible] accessible from home without the necessity of intermediaries) [28, p31]<br>• [illegible] described that the Web-BCPA provided opportunities to rehabilitation during periods with severe symptoms without having to be present at the health care center [38, p6] |
| Quality and quantity of content | Quality of content | • Contradicting content between DHI and HCP | • For some employees the exercises suggested by the program conflicted with the exercises given by the physiotherapist [36, p5] | • Trustworthy content and source<br>• Easily understandable content<br>• High quality of content<br>• Steady content<br>• *Appropriate content | • *Knowing there is a serious website where there are contributions, it strengthens you a bit [28, p29]*<br>• Some users felt reassured because they had a trustworthy place where they could address concerns [27, p641] |

For peer review only

| | | | | | |
|---|---|---|---|---|---|
| | | | | | • These effects could be reached thanks to the specificities of the [illegible] trustworthiness (material was controlled by health professionals according to the criteria of Evidence Based Medicine) [28, p31]<br>• They [the users] were positive about the content, user-friendliness and web-based design. They said the information was easy to understand, to the point and conveniently arranged [36, p5]<br>• More than half of the OPs were positive about the content (e.g. information, exercises, instructions) [36, p5]<br>• [illegible] material helped them to construct their personal frame of reference about the nature and the |

For peer review only

| | | | | | |
|---|---|---|---|---|---|
| | | | | | course of their cLBP [27, p640] |
| | Amount of content | • Too much content to choose from<br>• Too much information to fully comprehend | • According to some people, Oneself provided too much information, risking creating confusion about the comprehension of the health problem and the identification of the best way to treat it: *There is a lot of information, probably almost too much, don't you think? [28, p29]*<br>• [illegible] to choose from its content, were experienced to restrain patient participation [38, p8] | • A lot of content to choose from | • The richness and trustworthiness of [illegible] helped them to construct their personal frame of reference about the nature and the course of their cLBP [28, p28]<br>• First, the quality and continual update of the website encouraged people to visit Oneself again and to continue thinking about self-management [28, p29] |
| Tailoring and personalisation | Tailoring, specificity and personalisation | • Content not tailored to individual needs and/or pain severity<br>• Content perceived not new or relevant | • [illegible] the advice and exercises were not specific enough, they did not apply to the [illegible]ation [illegible] [36, p5] | • Content accounting for individual needs and/or pain severity<br>• Self-identification in content<br>• Opportunity to influence treatment | • *..it was obvious that it (the rehabilitation) was about me, it wasn't about just anyone.. it was about my problems, my strengths and how I felt.. they (the HCPs* |

For peer review only

| | | | | | |
|---|---|---|---|---|---|
| | | | • Some persons perceived information not new nor relevant. In this case, the use of Oneself lead to feelings of hopelessness: two participants had the impression that again there was no solution for their problem [28, p29]<br>• *The exercises that you have on the website are good, but I can't do any of them, no. I tried to do them a bit on the bed, but with my arm that doesn't work, my knees that don't work... There are lots, indeed I had written down those that I could do, but then many times your will is missing (...) Then you get sick of it. I know, that it's for my own good that I should exercise, but after a while I... Then you don't have grand results, and so even* | | *started from a blank page, I was not fitted into an average template of how it ought to be.. it (the rehabilitation) started with my point of view [38, p4-5]*<br>• *I really liked the personalization. I thought it was a nice touch. Suggestions were more specific and tailored, which for me made them more relevant and likely for me to use them [37, p9]*<br>• *Previously I had read about CBT (Cognitive Behavioral Therapy), but I had never thought of it as a help for my condition.. I want to compare this rehabilitation with a smorgasbord from which is it easy to taste [38, p5]*<br>• *It gives you descriptions and you say: this stuff here.. I see it, I see it! I recognise myself in it,* |

For peer review only

| | | | | | |
|---|---|---|---|---|---|
| | | | *for my back sometimes I go through periods, moments where I'm, let's say, very diligent, and then sometimes… (…) Yes, it's interesting. But there are always the same things that you then don't do [28, p29]* | | *I recognise myself here [27, p640]*<br>• Informants experienced that being able to identify themselves with the content in the rehabilitation and finding it trustworthy were important to patient participation and being confirmed [38, p5]<br>• They [informants] described that they were confirmed when they could identify their illness experience and life situation, as well as their own thoughts and cognitions about their pain condition, in the texts and the assignments of the Web-BCPA [38, p7] |
| Motivation and support | Personal attributes and resources | • Adhering to biomedical model of LBP<br>• Seeing LBP as a marginal problem<br>• Preferring other treatment regimens, e.g. with human contact | • *I went to a doctor who told me 'there is nothing to do, just resign yourself to it'. So this unleashed really the research to find something. But after eight years I didn't find the magic* | • High level of awareness and self-management of LBP<br>• Aware that LBP would not be fixed with a medical solution and ready to accept active role | • In addition, some informants stated that their work experience, such as having a solution- [illegible] facilitated patient participation in the rehabilitation [38, p6] |

For peer review only

| | | | | | |
|---|---|---|---|---|---|
| | | • Lack of knowledge about LBP and treatments<br>• Physical health (e.g. pain, fatigue)<br>• Psychological symptoms | *cure, unfortunately. And one continuously hears 'they are doing new research!' But hopefully they will arrive in time in order to do something. (...) I'm always in search of the super novelty, the one that heals [28, p30]*<br>• One employee mentioned that the back or neck pain they were suffering from may have prevented them from sitting at a computer [36, p6]<br>• Pain, fatigue and other psychological symptoms were perceived to limit patient participation [38, p6]<br>• Three users could be defined as passive self-managers: They adhered to a traditional biomedical model of cLBP and were convinced that the solution of their problem had to be | • Emotional and cognitive resources, e.g. motivation, interest, commitment and self-confidence in self-management of LBP<br>• Enjoy solution focused work | • *I already know which road I have to follow in detail. I need details or confirmation on these details [28, p29]*<br>• They described emotions and cognitions that affected patient participation. Having motivation, interest, commitment, and self-confidence were perceived to favor patient participation [38, p6]<br>• Most of the users could be defined as experienced self-managers, in the sense that they had a rather high level of awareness and self-management of cLBP even before knowing Oneself. These [illegible] rather clear idea about their diagnosis, and knew that they had to play an active role in dealing with their health problem [27, p635] |

For peer review only

| | | | | | |
|---|---|---|---|---|---|
| | | | found by health professionals. These people went to Oneself to find a definitive medical solution for their cLBP [27, p635]<br>• Three users could be defined as latent self- u v P [illegible] of them, cLBP was at the moment a marginal problem, in the sense that it was intermittent and light. These users did not really need to engage in a long-term process of self-management: When pain appeared, they usually dealt with it through some easy coping strategies, such as taking painkillers, going to the chiropractic, etc [27, p636] | | • Two users could be defined as novices in terms of self-management. These participants were aware that a medical solution to cLBP did not exist and were ready to accept that they had to become actively involved in their cLBP care. However, they did not know how to do it [27, p635] |
| | Support to use DHI | • HCP unsupportive of use of DHI<br>• No support from authorities | • *I planned to complete the program (the Web-BCPA).. I am not sure how much I had left.. probably the last module.. but I was denied sick-leave* | • HCP supportive of use of DHI<br>• Support from family<br>• Support from authorities<br>• Support from other suffers (e.g. successful testimonials) | • *It's nice knowing that there is someone else [28, p29]*<br>• *When you are going through a moment when you have backache and you* |

For peer review only

| | | | | | |
|---|---|---|---|---|---|
| | | | *compensation by the Social Insurance Agency and had to put in a lot of energy to explain my situation and meet with the psychosocial counsellor.. I did not have the strength to do anything else.. I have used so much energy to fight for my cause [12052, 6]*<br>• One employee said, *I expected more commitment from my OP*. This did not encourage employees to use the program [2120, 5] | | *read a testimony which says 'yes, there is someone who was able to do it', it gives you hope [28, p29]*<br>• Support, trust and respect from a family member, employer, the Swedish Social Insurance Agency (SSIA) or the Employment Service were experienced to facilitate patient participation in the rehabilitation [38, p6] |
| | Features of DHI | • DHI not guiding or supporting participants enough (e.g. to plan for execution of physical activity recommendation from DHI) | • *I received the suggestion to ride a bike, but that's currently simply not possible, logistically [37, p10]*<br>• *If it could ask me to rank the things I enjoy doing and then download weather data for the following days. This could suggest times when I have performed these tasks in the* | • Interaction/interactivity<br>• Information about self-management of LBP<br>• Goal-setting<br>• Action-planning<br>• Follow-up and evaluation<br>• Adjusting treatment related to setbacks and progress<br>• Monitoring own progress in graphs<br>• Variation of content<br>• Update of content | • To acquire knowledge and insights were thought of as patient participation, and included self-reflection, self-identification, and feedback [38, p5]<br>• [illegible] opportunities to influence and a variety of treatments to choose according [illegible] and priorities [38, p5] |

For peer review only

| | | | | | |
|---|---|---|---|---|---|
| | | | *past and also match it with weather predictions. "You played tennis for last Tuesday in the afternoon for 90 minutes. How about from 2 to 4 today when the weather will be clear and 85".* *[37, p10]* | | • To adjust a goal or treatment planning in relation to progress or setback was described as patient participation: *I feel it is important to set goals and to follow-up those goals.. and to why a goal is reached and why another is not.. this made me aware of that I needed other tools (in the rehabilitation)* [38, p6]<br>• Patient participation was reported when informants monitored results shown by the interactive graphs in the Web-BCPA: .. *days when I had a lot of pain I used to remain sedentary, and as soon as I had a better day I was eager to do all kinds of activities that day.. before I started with the assignment activity planning (in the Web-BCPA) I was* |

For peer review only

| | | | | | |
|---|---|---|---|---|---|
| | | | | | *not aware of how my behaviour related to the days with pain, but by monitoring this over time I started to plan my daily activities in a more balanced way [38, p6]*<br>• These effects could be reached thanks to the specificities of the website, that is its interactivity (people could ask specific questions to health professionals who were available daily [illegible] dynamism (the website was updated [illegible] 28, p31] [illegible]<br>• [illegible] experienced patient participation when they analyzed their situation taken into account their resources and restrictions, set goals for behavior change, and planned treatments and activities. Also, patient participation was stated when |

For peer review only

| | | | | | |
|---|---|---|---|---|---|
| | | | | | treatments, self-care, and planning were followed-up and evaluated [38, p6] |
| | HCP factors for support of patients | • *Time restrictions of consultations<br>• *Difficulty keeping DHI in mind during consultations<br>• *Difficulty providing patients with accurate information about DHI<br>• *Perceiving no benefit of DHI compared to usual treatment<br>• *Preferring other treatment regimens, e.g. with human contact | • *It takes time to get used to the recruitment process and to using the program [36, p5]*<br>• A second important barrier for OPs was the limited time available for introducing employees to the program and working [illegible] *We lack the time to do this kind of projects [36, p5]*<br>• One OP stated that he did not use the program because he did not believe in [illegible] based [illegible] physical pain. He explained, *The ability to touch people is an essential element in the treatment of people with back or neck pain. [36, p5]* | • *DHI a good medium for counselling employees | • About half of the OPs indicated that a website is a good medium for counselling of employees with back or neck pain [36, p5] |

| | | | | | |
|---|---|---|---|---|---|
| | | | • One OP stated that he was quite capable of managing the RTW process himself and did not need a program for additional support. Many preferred the more familiar therapies (e.g. [illegible] They preferred having personal contact with employees [36, p5] | | |

*= HCP perspective; **IT**: information technology; **HCP**: healthcare professional; **DHI**: Digital health intervention

For peer review only

# PRISMA 2009 Checklist

| Section/topic | # | Checklist item | Reported on page # |
|---|---|---|---|
| **TITLE** | | | |
| Title | 1 | Identify the report as a systematic review, meta-analysis, or both. | 1 |
| **ABSTRACT** | | | |
| Structured summary | 2 | Provide a structured summary including, as applicable: background; objectives; data sources; study eligibility criteria, participants, and interventions; study appraisal and synthesis methods; results; limitations; conclusions and implications of key findings; systematic review registration number. | 2-3 |
| **INTRODUCTION** | | | |
| Rationale | 3 | Describe the rationale for the review in the context of what is already known. | 4-5 |
| Objectives | 4 | Provide an explicit statement of questions being addressed with reference to participants, interventions, comparisons, outcomes, and study design (PICOS). | 5 |
| **METHODS** | | | |
| Protocol and registration | 5 | Indicate if a review protocol exists, if and where it can be accessed (e.g., Web address), and, if available, provide registration information including registration number. | 6 |
| Eligibility criteria | 6 | Specify study characteristics (e.g., PICOS, length of follow-up) and report characteristics (e.g., years considered, language, publication status) used as criteria for eligibility, giving rationale. | 6-7 |
| Information sources | 7 | Describe all information sources (e.g., databases with dates of coverage, contact with study authors to identify additional studies) in the search and date last searched. | 7 |
| Search | 8 | Present full electronic search strategy for at least one database, including any limits used, such that it could be repeated. | 7 + suppl. file 2 |
| Study selection | 9 | State the process for selecting studies (i.e., screening, eligibility, included in systematic review, and, if applicable, included in the meta-analysis). | 7-8 |
| Data collection process | 10 | Describe method of data extraction from reports (e.g., piloted forms, independently, in duplicate) and any processes for obtaining and confirming data from investigators. | 8 |
| Data items | 11 | List and define all variables for which data were sought (e.g., PICOS, funding sources) and any assumptions and simplifications made. | 7 |
| Risk of bias in individual studies | 12 | Describe methods used for assessing risk of bias of individual studies (including specification of whether this was done at the study or outcome level), and how this information is to be used in any data synthesis. | 8 |
| Summary measures | 13 | State the principal summary measures (e.g., risk ratio, difference in means). | 8 |

For peer review only - http://bmjopen.bmj.com/site/about/guidelines.xhtml

# PRISMA 2009 Checklist

| | | | |
|---|---|---|---|
| Synthesis of results | 14 | Describe the methods of handling data and combining results of studies, if done, including measures of consistency (e.g., $I^2$) for each meta-analysis. | 8-10 |



| Section/topic | # | Checklist item | Reported on page # |
|---|---|---|---|
| Risk of bias across studies | 15 | Specify any assessment of risk of bias that may affect the cumulative evidence (e.g., publication bias, selective reporting within studies). | N/A |
| Additional analyses | 16 | Describe methods of additional analyses (e.g., sensitivity or subgroup analyses, meta-regression), if done, indicating which were pre-specified. | N/A |
| **RESULTS** | | | |
| Study selection | 17 | Give numbers of studies screened, assessed for eligibility, and included in the review, with reasons for exclusions at each stage, ideally with a flow diagram. | 10 |
| Study characteristics | 18 | For each study, present characteristics for which data were extracted (e.g., study size, PICOS, follow-up period) and provide the citations. | 11-15 |
| Risk of bias within studies | 19 | Present data on risk of bias of each study and, if available, any outcome level assessment (see item 12). | 16 + suppl. file 3 |
| Results of individual studies | 20 | For all outcomes considered (benefits or harms), present, for each study: (a) simple summary data for each intervention group (b) effect estimates and confidence intervals, ideally with a forest plot. | 11-16 |
| Synthesis of results | 21 | Present results of each meta-analysis done, including confidence intervals and measures of consistency. | 16-24 |
| Risk of bias across studies | 22 | Present results of any assessment of risk of bias across studies (see Item 15). | N/A |
| Additional analysis | 23 | Give results of additional analyses, if done (e.g., sensitivity or subgroup analyses, meta-regression [see Item 16]). | N/A |
| **DISCUSSION** | | | |
| Summary of evidence | 24 | Summarize the main findings including the strength of evidence for each main outcome; consider their relevance to key groups (e.g., healthcare providers, users, and policy makers). | 24-28 |
| Limitations | 25 | Discuss limitations at study and outcome level (e.g., risk of bias), and at review-level (e.g., incomplete retrieval of identified research, reporting bias). | 28-29 |
| Conclusions | 26 | Provide a general interpretation of the results in the context of other evidence, and implications for future research. | 29 |
| **FUNDING** | | | |
| Funding | 27 | Describe sources of funding for the systematic review and other support (e.g., supply of data); role of funders for the systematic review. | 30 |

For peer review only

# PRISMA 2009 Checklist

*From:* Moher D, Liberati A, Tetzlaff J, Altman DG, The PRISMA Group (2009). Preferred Reporting Items for Systematic Reviews and Meta-Analyses: The PRISMA Statement. PLoS Med 6(7): e1000097. doi:10.1371/journal.pmed1000097

For more information, visit: **www.prisma-statement.org**.



For peer review only

For peer review only - http://bmjopen.bmj.com/site/about/guidelines.xhtml

BMJ Open

# Barriers and facilitators to patient uptake and utilisation of digital interventions for the self-management of low back pain: a systematic review of qualitative studies

| | |
|---|---|
| Journal: | *BMJ Open* |
| Manuscript ID | bmjopen-2020-038800.R1 |
| Article Type: | Original research |
| Date Submitted by the Author: | 23-Sep-2020 |
| Complete List of Authors: | Svendsen, Malene; National Research Centre for the Working Environment, Musculoskeletal disorders and physical work demands; University of Southern Denmark Department of Sports Science and Clinical Biomechanics, Physical activity and health in work life<br>Wood, Karen; University of Glasgow<br>Kyle, John ; University of Glasgow, Institute of Health and Wellbeing<br>Cooper, K; Robert Gordon University, School of Health Sciences<br>Rasmussen, Charlotte Diana Nørregaard; The National Research Centre for The Working Environment,<br>Sandal, Louise; University of Southern Denmark, Department of Sport Science and Clinical Biomechanics<br>Stochkendahl, Mette; Nordic Institute of Chiropractic and Clinical Biomechanics, ; University of Southern Denmark, Institute of Sports Science and Clinical Biomechanics<br>Mair, Frances; University of Glasgow, General Practice and Primary Care<br>Nicholl, Barbara; University of Glasgow, General Practice and Primary Care |
| <b>Primary Subject Heading</b>: | Public health |
| Secondary Subject Heading: | Qualitative research |
| Keywords: | Back pain < ORTHOPAEDIC & TRAUMA SURGERY, Health informatics < BIOTECHNOLOGY & BIOINFORMATICS, PAIN MANAGEMENT, QUALITATIVE RESEARCH |
| | |

SCHOLARONE™
Manuscripts

*I, the Submitting Author has the right to grant and does grant on behalf of all authors of the Work (as defined in the below author licence), an exclusive licence and/or a non-exclusive licence for contributions from authors who are: i) UK Crown employees; ii) where BMJ has agreed a CC-BY licence shall apply, and/or iii) in accordance with the terms applicable for US Federal Government officers or employees acting as part of their official duties; on a worldwide, perpetual, irrevocable, royalty-free basis to BMJ Publishing Group Ltd ("BMJ") its licensees and where the relevant Journal is co-owned by BMJ to the co-owners of the Journal, to publish the Work in this journal and any other BMJ products and to exploit all rights, as set out in our licence.*

*The Submitting Author accepts and understands that any supply made under these terms is made by BMJ to the Submitting Author unless you are acting as an employee on behalf of your employer or a postgraduate student of an affiliated institution which is paying any applicable article publishing charge ("APC") for Open Access articles. Where the Submitting Author wishes to make the Work available on an Open Access basis (and intends to pay the relevant APC), the terms of reuse of such Open Access shall be governed by a Creative Commons licence – details of these licences and which Creative Commons licence will apply to this Work are set out in our licence referred to above.*

*Other than as permitted in any relevant BMJ Author's Self Archiving Policies, I confirm this Work has not been accepted for publication elsewhere, is not being considered for publication elsewhere and does not duplicate material already published. I confirm all authors consent to publication of this Work and authorise the granting of this licence.*

For peer review only

# Barriers and facilitators to patient uptake and utilisation of digital interventions for the self-management of low back pain: a systematic review of qualitative studies

Malene Jagd Svendsen[2,5], Karen Wood[1], John Kyle[1], Kay Cooper[3], Charlotte Diana Nørregaard Rasmussen[2], Louise Fleng Sandal[5], Mette Jensen Stochkendahl[5,6], Frances S Mair[1], Barbara I Nicholl[1]

**Affiliations**

1. Institute of Health & Wellbeing, University of Glasgow, Glasgow, UK

2. National Research Centre for the Working Environment, Copenhagen Ø, Denmark

3. School of Health Sciences, Robert Gordon University, Aberdeen, UK

4. Department of Public Health and Nursing, Faculty of Medicine, NTNU, Norwegian University of Science and Technology, Trondheim, Norway

5. Department of Sports Science and Clinical Biomechanics, University of Southern Denmark, Odense, Denmark

6. Nordic Institute of Chiropractic and Clinical Biomechanics, Odense, Denmark

**Email Addresses**

Malene Jagd Svendsen mas@nrcwe.dk

Karen Wood Karen.Wood@glasgow.ac.uk

John Kyle John.Kyle@nhs.net

Kay Cooper k.cooper@rgu.ac.uk

Charlotte Diana Nørregaard Rasmussen cnr@nrcwe.dk

Louise Fleng Sandal lsandal@health.sdu.dk

Mette Jensen Stochkendahl m.jensen@nikkb.dk

For peer review only

Frances S Mair Frances.Mair@glasgow.ac.uk

Barbara Nicholl Barbara.Nicholl@glasgow.ac.uk

**Corresponding Author:**

Malene Jagd Svendsen

National Research Centre for the Working Environment, Lersø Parkallé 105, 2100 Copenhagen, Denmark

Tel: +45 39165329 Email: mas@nrcwe.dk

Word count: 6531 words

**Abstract**

**Objectives:** Low back pain (LBP) is a leading contributor to disability globally. Self-management is a core component of LBP management. We aimed to synthesise published qualitative literature concerning digital health interventions (DHIs) to support LBP self-management to: 1) determine engagement strategies, 2) identify barriers and facilitators affecting patient uptake/utilisation, 3) develop a preliminary conceptual model of barriers and facilitators to uptake/utilisation.

Design: Systematic review following PRISMA guidelines.

Data sources: MEDLINE, Embase, CINAHL, PsycINFO, Cochrane Library, DoPHER, TROPHI, Web of Science and OT Seeker, from January 2000 – December 2018, using the concepts: LBP, DHI, self-management.

Eligibility criteria: Peer-reviewed qualitative study (or component) examining engagement with, or barriers and/or facilitators to the uptake/utilisation of, an interactive DHI for self-management of LBP in adults (community, primary or secondary care settings).

Data extraction and synthesis: Standardised data extraction form was completed for included studies. COREQ checklist was used to assess methodology. Data was synthesised narratively for engagement

For peer review only



For peer review only - http://bmjopen.bmj.com/site/about/guidelines.xhtml

strategies, thematically for barriers/facilitators to uptake/utilisation, and normalisation process theory was applied to produce a conceptual model.

**Results:** Our systematic search resulted in inclusion of five full text articles from four studies. These were from community and primary care contexts in Europe and the US, and involved, in total, 56 adults with LBP and 19 healthcare professionals. There was a lack of consideration on how to sustain engagement with DHIs. Examination of barriers and facilitators for uptake/utilisation identified four major themes: IT usability-accessibility; quality-quantity of content; tailoring-personalisation; motivation-support. These themes and sub-themes informed the development of a preliminary conceptual model for uptake/utilisation of a DHI for LBP self-management.

**Conclusions**: We highlight key barriers and facilitators that should be considered when designing DHIs for LBP self-management . Our findings are in keeping with reviews of DHIs for other long-term conditions, implying these findings may not be condition specific.

**PROSPERO Registration number** CRD42016051182

**Funding**: European Union Horizon 2020 Research and Innovation Programme (grant agreement No 689040)

**Systematic review registration**: A protocol for this systematic review was registered with https://www.crd.york.ac.uk/PROSPERO/ (CRD42016051182) on November 10th, 2016. https://www.crd.york.ac.uk/PROSPERO/display_record.php?ID=CRD42016051182

**Article summary**

Strengths and limitations of this study:

For peer review only



For peer review only - http://bmjopen.bmj.com/site/about/guidelines.xhtml

- This systematic review of qualitative studies explored barriers and facilitators for the uptake and utilisation of digital health interventions for low back pain (LBP) to inform the future design and implementation processes of such interventions.
- Searches in multiple databases and independent data extraction, quality appraisal and detailed data analysis are strengths of our review. However, our search strategy revealed that literature in the field of digital self-management for LBP is sparse as only a small number of eligible studies were identified.
- Given the limited literature, it is possible that not all important barriers and facilitators for uptake and utilisation have been identified and thus our conceptual model must be considered preliminary.

**Keywords:** Low back pain; eHealth; self-management; qualitative, engagement; utilisation; NPT

## Background

Low back pain (LBP) affects approximately 12% of the general population at any point in time (1); it is the leading contributor to disability worldwide (2) and is associated with significant personal (3) and societal costs (4, 5). Self-management approaches are consistently recommended in clinical guidelines as a core component of LBP management (6, 7); however, adherence to self-management strategies has proved challenging, especially without support and reinforcement (8, 9). Digital health interventions (DHIs), health interventions accessed through a computer, mobile phone, or other handheld device, involving a web-based programme, desktop programme or application; offer a potential method of supporting self-management (10-12), and particularly the possibility of tailoring self-management advice, may hold significant potential for people with LBP (13). DHIs or "digital therapeutics" are becoming increasingly popular and, as technological innovations increase, it is expected that this trend will continue (14, 15). Until now, two systematic reviews have examined the use of DHIs to support the self-management of LBP. The



For peer review only - http://bmjopen.bmj.com/site/about/guidelines.xhtml

first, by Garg et al., aimed to determine which web-based interventions are of benefit to patients (16). They identified nine randomised controlled trials (RCTs), including a total of 1796 participants. Four trials studied online cognitive behavioural therapy (CBT) with the remaining five trials studying web-based interventions with interactive features such as a virtual gym, testimonials, or moderated discussion groups. Garg et al. reported that online CBT approaches appeared to reduce catastrophizing and improve patient attitudes, whilst studies of web-based interventions with interactive features used a variety of diverse outcome measures yielding inconclusive results; thus, making it difficult to draw firm conclusions regarding long-term impact for people with LBP.

The second review, by Nicholl et al., aimed to appraise the evidence concerning the use of interactive DHIs to support patient self-management of LBP with a focus on the outcome measures used and reported effects (17). They identified six completed RCTs studying digital tools for the self-management of LBP including a total of 2706 participants. Nicholl et al. reported that only one of the six completed RCTs observed a between-group difference in favour of the digital intervention, with none of the studies demonstrating any evidence of harm. The authors noted that there was considerable variation in the nature and delivery of the interventions and inconsistency in the choice of outcomes and concluded that the current evidence base for DHIs to support the self-management of LBP remained weak.

Yet, hundreds of smartphone applications (apps) related to LBP are currently available on the app market, most developed with very little scientific rigour (18). In order to facilitate the development of appropriate and effective self-management DHIs for those with LBP, it is important to have an understanding of the factors that help or hinder user engagement and adherence. Across different conditions, multiple barriers and facilitators to engaging with DHIs have previously been identified, including issues such as motivation and support, digital literacy, privacy, usability, quality and tailoring (17, 19). However, given the diverse range of DHIs available, it can be difficult to apply these findings to a specific patient population or piece of technology. Understanding the experience of users of DHIs designed specifically to assist self-management of LBP would help determine how to optimise DHIs for this group of users.



For peer review only - http://bmjopen.bmj.com/site/about/guidelines.xhtml

The purpose of this systematic review was therefore to synthesise and critically appraise the published qualitative literature concerning the use of DHIs to promote self-management of LBP in order to address the following two research questions:

1. What engagement strategies at the time of enrolment have been utilised in DHIs aimed at supporting patient self-management of LBP?
2. What are the barriers and facilitators to patient uptake and utilisation of digital interventions to support self-management of LBP?

The final objective of the systematic review was to develop a preliminary conceptual model of barriers and facilitators to uptake and utilisation of digital interventions to support self-management of LBP.

**Methods**

Protocol and registration

This review was registered in the International Prospective Register of Systematic Reviews, PROSPERO, registration no. CRD42016051182 (20) and reporting is consistent with the Preferred Reporting Items for Systematic Reviews and Meta-Analyses (PRISMA) statement (21).

Eligibility criteria

Qualitative studies that examine engagement, barriers and/or facilitators to patient uptake and utilisation of digital interventions for the self-management of LBP were included; inclusion and exclusion criteria are outlined in Table 1.

**Table 1:** Inclusion and exclusion criteria.

| **Inclusion criteria** | |
|---|---|
| Study type | • Published in peer-reviewed journals between January 1st 2000 and December 18th 2018.<br>• Original qualitative studies, studies involving secondary qualitative analysis of qualitative data and qualitative studies that were part of a mixed methods study (provided the qualitative methodology was described).<br>• Qualitative data collected via questionnaires or other methods not involving direct contact or observation of participants were eligible for |

For peer review only

| | |
|---|---|
| | inclusion provided questions were answered using free text and analysed using a qualitative approach.<br>• Qualitative data describing barriers and/or facilitators to the uptake or utilisation of digital interventions or containing a description of an engagement strategy (i.e. any method used to get people to enrol into the study) from a patient or HCP's perspective. |
| Language | • Published in English, Danish or Norwegian. |
| Participants | • Adults >18 years with LBP or HCPs providing care for such patients. |
| Setting | • Community, primary or secondary care and other specialist contexts including those that recruit via media. |
| Digital intervention | • Any intervention accessed through a computer, mobile phone, or other handheld device, involving a web-based programme, desktop programme or application that provided self-management content (consistent with previous reviews (17, 22)).<br>• Interventions must involve an element of interaction between the user and the digital interface; this was defined as information being taken from users which then provided some form of automated feedback and/or advice in response.<br>• Interventions that included face-to-face contact were only included if this interaction was in addition to an automated, interactive digital component without direct HCP mediation. |
| **Exclusion criteria** | |
| Study type | • Descriptive case studies, lexical studies that analyse natural language data presented as qualitative results, literature or systematic reviews, meta-analyses, studies without a sampling procedure (i.e. no clear description of recruitment strategy) and commentary articles written to convey opinion or stimulate discussion with no research component. |

**HCP**: Healthcare professional

For peer review only

Information sources and search strategy

A systematic search of bibliographic databases (MEDLINE, Embase, CINAHL, PsycINFO, Cochrane Library, DoPHER, TROPHI, Web of Science and OT Seeker) was conducted after the search strategy had been developed in collaboration with a librarian at the Norwegian University of Science and Technology (NTNU) and experienced researchers in the field of LBP and digital health interventions. The search strategy has previously been described and published by Nicholl et al. (17). Reference and citation tracking was utilised to identify relevant references. All databases were searched for publications using three groups of concepts: (1) low back pain, (2) digital intervention, and (3) self-management. The search was conducted in three waves using the same search strategy: the first for publications added between January 2000 and



For peer review only - http://bmjopen.bmj.com/site/about/guidelines.xhtml

March 2016, then a subsequent updated search for articles added between March 2016 and October 2016, and lastly, articles added between October 2016 and December 2018. Limitation of year of publication from 2000 onwards was chosen as our review was aimed at understanding current experiences of digital health technologies, justified by emerging Internet access around the millennium and the developing field of DHIs that followed, and further supported by other systematic reviews of digital interventions (16, 23, 24). The complete search strategy, including specifications on the use of title, keywords or abstract screening is documented in Supplementary File 1.

Study selection

All identified citations were uploaded to Distiller SR software (Evidence Partners, Ottawa, Canada) and duplicates were removed. Title and abstract screening were performed by two of four independent reviewers (JK, MaS, KC, KW) using Distiller SR. Any disagreement between the two reviewers at title screening level resulted in inclusion of the citation to abstract level and subsequently any disagreement at abstract level resulted in inclusion of the citation to the full-text screening level. Full-text screening was also performed by two of four independent reviewers (JK, MaS, KC, KW) with any discrepancies at this level being resolved through discussion mediated by a third party (BN, CR, MeS, KC).

Data extraction

A comprehensive, standardised data extraction template designed specifically for this review in Distiller SR was utilised by two of four independent researchers (JK, MaS, BN, KW). Where available, information collected included the study title, authors, citation, year of study and publication, country, inclusion/exclusion criteria, aim, setting, characteristics of the digital intervention, recruitment methods, method of qualitative data collection and analysis, participant numbers and characteristics, any engagement strategies, barriers or facilitators identified either by the authors or in participant quotes, conclusions, limitations, funding sources and any potential conflicts of interest declared.



For peer review only - http://bmjopen.bmj.com/site/about/guidelines.xhtml

Quality appraisal

The complete 32-item Consolidated Criteria for Reporting Qualitative Research (COREQ) checklist (25, 26) was used to assess the methodological quality of the articles progressing to data extraction. Two of three reviewers (BN, KC, KW) independently identified whether each of the 32-items were reported or not, and descriptive information was provided where possible. Disagreements between reviewers were resolved through discussion. A-priori cut-off points were not determined as studies were not excluded on the basis of methodological quality due a lack of clear agreement on how best to apprise qualitative literature (27). Two of the included articles report on the qualitative evaluation of the same intervention but were treated as separate articles for quality appraisal (28, 29).

Data synthesis and analysis

Information on the engagement strategies, defined as methods used to recruit and initially motivate participants to enrol in the DHI study, in each study was described narratively as this was only provided descriptively in the included studies. Our data synthesis of barriers and facilitators to patient uptake and utilisation of the DHI for LBP involved a thematic approach (30). Data on barriers and facilitators were extracted from results and discussion sections of the included studies. Each item of extracted data was initially coded by one reviewer (MaS). When new codes appeared during the analysis of a particular article, the articles that had previously been examined were re-read and re-coded if appropriate. This continuous adjustment was carried out in cooperation with a second reviewer (KW). Emergence and mapping of codes were discussed at coding clinics to ensure construction of themes that were internally homogenous and externally heterogeneous (i.e. no data excluded due to lack of a suitable theme, and no data falling between two themes or fitting into more than one theme) (31, 32) (MaS, KW, FM, BN). This resulted in a coding taxonomy for mapping identified codes as barriers or facilitators for each theme.



For peer review only - http://bmjopen.bmj.com/site/about/guidelines.xhtml

202 A preliminary conceptual model of barriers and facilitators to uptake and utilisation of DHIs to support self-management of LBP was developed by mapping the identified themes to the four constructs of Normalization Process Theory (NPT). NPT is a sociological theory developed to explore the process of implementing a new complex intervention, in this case it can help explain how people individually and collectively embed DHIs into everyday practice (33, 34). The identified themes were mapped to NPT constructs by four reviewers (KW, FM, BN, JK) using the coding framework presented in Table 2. This approach has been successfully applied in other systematic reviews of DHIs for chronic disease self-management issues (19, 35, 36) and provides a solid conceptual basis from which to understand barriers and facilitators to patient and HCP uptake and utilisation of DHIs. Any themes that could not be coded to the NPT constructs were carefully noted to ensure that themes outside the scope of NPT would still be captured to assure appropriateness of the model.

**Table 2:** Core constructs of Normalization Process Theory (NPT) (33, 34) and related coding framework for development of preliminary conceptual model of barriers and facilitators to uptake and utilisations of digital interventions to support self-management of LBP.

| **Core constructs of NPT** | **Coding framework** |
|---|---|
| Coherence (Sense Making Work; enrolling with the DHI): development of an individual and collective understanding of the new intervention when faced with operationalizing it. | • How people understand and view the benefits versus disbenefits of DHIs and decide whether it is appropriate for them to use.<br>• Motivation and willingness to commit to self-management activities. |
| Cognitive Participation (Engagement Work; engaging with the DHI): relational work to build and sustain engagement with a new intervention. | • Willingness to "buy into" the DHI and whether it is a legitimate means to promote self-management of LBP.<br>• Issues relating to the support provided to use the DHI and level of engagement of HCPs involved with the DHI. |
| Collective Action (Operationalisation Work; utilising the DHI): investment of effort and resources to enact the new intervention. | • Ease of use, accessibility and appropriateness of the DHI.<br>• Resources, training, workload and technical support.<br>• Perceived quality and trustworthiness of DHI content and function. |
| Reflexive Monitoring (Appraisal Work; maintaining engagement with DHI): evaluation of the impact of the new intervention on individuals and groups along with any reconfigurations suggested. | • How people judge the new DHI and the self-monitoring work that accompanied uptake of the DHI.<br>• Ability to tailor to an individual's needs. |
| **Codes falling outside the NPT framework** | |
| | • Inherent personal attributes such as personal physical or cognitive abilities that could promote or inhibit DHI use. |



For peer review only - http://bmjopen.bmj.com/site/about/guidelines.xhtml

**DHI**: Digital health intervention; **HCP**: Healthcare professional

## Results

Study selection

Of 14191 citations identified, 5973 were excluded as duplicates; 8113 were excluded following title and abstract screening (7436 at title level and 677 at abstract level) and a further 100 citations were excluded after full text screening. Overall, five full text articles were included in the review (Figure 1). These articles described four separate studies and included a total of 75 participants. The two articles (28, 29) reporting on the same study (Oneself) consisted of a qualitative evaluation of a website (29) and a mixed-method reporting of the same qualitative data combined with quantitative (pre- and post-use surveys and log files) data (28). As these two studies included the same qualitative data and user quotes, they were combined for analysis purposes.

**Figure 1** PRISMA flow diagram illustrating the screening process (Adapted from Moher et al (21)).

Study characteristics

The Get Well Fast (37) and Oneself studies (28, 29) were undertaken between 2006 and 2008 in the Netherlands and Switzerland, respectively. The MyBehaviorCBP study was conducted in the US between 2012 and 2014 (38), whilst the study period for the Swedish Web-BCPA study was not reported (39). The characteristics of the study participants are summarised in Table 3. No information was reported on comorbidities or ethnicity and only limited information on participant socioeconomic status was included.



For peer review only - http://bmjopen.bmj.com/site/about/guidelines.xhtml

**Table 3:** Participant characteristics of included studies

| Study; Country | Year of study | Number of participants in qualitative study | Age range | Sex (%) | SES |
|---|---|---|---|---|---|
| Oneself (28, 29) Switzerland | 2006-2008 | N = 18 | 28-72 years<br><br><29 yrs: n = 1<br>30-39 yrs: n = 3<br>40-49 yrs: n = 5<br>50-59 yrs: n = 6<br>>60 yrs: n = 3 | 50% female | *Education*:<br>Secondary school: n = 2; High school or equivalent: n = 11; University degree: n = 5 |
| Get Well Fast (37) Netherlands | 2008 | N = 28<br><br>OP+ = 11<br>OP- = 8<br>Employee: 9 | 40-50 years | OP: N/R<br><br>Employee: 33% female | White and blue-collar workers. Various levels of education |
| MyBehaviorCBP (38) USA | 2012-2014 | N = 10 | 31-60 years | 70% female | N/R |
| Web-BCPA (39) Sweden | N/R | N = 19 | 27-60 years | 79 % female | *Education:*<br>Elementary school: n = 2; Secondary school: n = 12; University degree: n = 5)<br><br>*Employment:*<br>Permanent employment: n = 12; Temporary employment: n = 3; Unemployed: n = 3; Social benefits: n = 1 |

**N:** Number; **OP+**: occupational physicians who recruited patients into DHI; **OP-:** occupational physicians who did not recruit patients into DHI; **N/R:** not reported; **SES:** socioeconomic status

DHI delivery mode varied between studies. In the Oneself, Get Well Fast and Web-BCPA studies, the DHI consisted of information available on websites to which participants had either open access (28, 29) or had personal log-ins (37, 39). The content of the MyBehaviorCBP intervention was delivered to participants via a mobile phone app (38). Two of the studies tailored the content of their DHI to the individual participant by collecting information about the users and providing content that matched their needs (37, 38); in the



For peer review only - http://bmjopen.bmj.com/site/about/guidelines.xhtml

Get Well Fast study, content was tailored based on patient reports on pain, limitations, treatment, counselling, reintegration to work, work situation and work characteristics, relations at work, personality and daily activities (37), while the MyBehaviourCBP intervention collected sensory data from the users' smartphone (accelerometer signals and geolocation) and patient self-reported physical activity logs (38). Three interventions offered time limited programs of either five (37, 38) or eight weeks (39), while the fourth intervention was an open-to-access website with no time restrictions (28, 29) (Table 4).

**Table 4:** Participant inclusion criteria, sampling procedure for qualitative component and characteristics of digital intervention in included studies

| Study | Inclusion criteria for digital health intervention | Inclusion criteria and sampling procedures for qualitative study | Characteristics of digital health intervention |
|---|---|---|---|
| Oneself (28, 29) | • Anyone could register and use the Oneself website. | • Registered users of Oneself for at least 6 months.<br>• Visited the website at least 3 times.<br>• Suffering from chronic LBP (duration not defined).<br>• Living in the Italian part of Switzerland.<br>• Invitation to participate in interview sent via email to eligible users (N=238).<br>• Reminder email sent after 2 weeks to anyone who had not responded. | Open access website containing:<br>• Library – textual educational information on back pain.<br>• Radio – 10x2-minute recorded audio messages on relevant topics.<br>• Gym - videos demonstrating stretching, stabilization and mobilization exercises accompanied by photographs and written descriptions.<br>• Forum – users could interact with other users and HCPs, monitored by a content manager.<br>• Chat room – users could interact with other users and HCPs. Once a week, a HCP would be available to discuss specific topics selected from conversations published on the Forum.<br>• Specialist answers – information on topics suggested by users.<br>• Testimonials - users could share stories and comment on other users' stories. |

For peer review only



For peer review only - http://bmjopen.bmj.com/site/about/guidelines.xhtml

| | | | |
|---|---|---|---|
| | | | • Ability for users to request information they felt lacked on the website. |
| Get Well Fast (37) | • Employees of KLM Royal Dutch Airlines or National Railways and their OPs.<br>Employee criteria:<br>• Contracted for at least 12 hours per week.<br>• Absent from work for a minimum of 2 weeks due to non-specific back or neck pain.<br>• No serious health problems defined as "warning flags: e.g. fever, pain in arms or legs, serious disease".<br>• Ability to speak and write in Dutch.<br>• Internet access. | • Users of the Get Well Fast website.<br>• The employees' OPs.<br>• All employees using the website and OPs were invited to participate in an interview. | • Web-based, 5-weeks programme during which the employee completed 4 questionnaires and received tailored information via a personal digital diary.<br>• Based on weekly questionnaires, information about advice on improving physical fitness, setting a daily timetable, pain-coping strategies, and exercise instructions is provided.<br>• Employees spent around 15 minutes/day reading information, completing questionnaires, and following exercises.<br>• Employee's OP had access to the employee's diary and received reports when the employee completed a questionnaire, detailing the employee's condition, current treatments, and absence details. |
| MyBehaviorCBP (38) | • Aged 18-65 years<br>• History of chronic back pain (≥6 months).<br>• Willingness to use MyBehaviorCBP app on an Android mobile phone (own or provided by study).<br>• Reasonable level of outdoor movement (e.g. travelling to and from work).<br>• Not being significantly housebound.<br>• Fluent in English<br>• Basic level of mobile proficiency. | • All participants in received web-based exit survey; one question was open ended and results from this component of the study are included in this review. | • 5-week app based programme during which participants received recommendations for PA.<br>• App tracks participant's mobility state and geolocation using in-phone sensors or manual input. Recurring patterns of PA form base for new PA recommendations.<br>• Week 1 - baseline period: no recommendations were given.<br>• Week 2 & 3 - control phase: PA recommendations were random, generic and unrelated to participants' past behaviour. |



For peer review only - http://bmjopen.bmj.com/site/about/guidelines.xhtml

| | | | |
|---|---|---|---|
| | | | • Week 4 & 5 – experimental phase: PA recommendations generated by MyBehaviorCBP based on PA behaviour during control phase.<br>• Participants were blinded to when the different PA recommendation forms were activated. Participants completed a daily in-phone survey regarding ease of following recommendations, how many recommendations they followed, and their emotional state. |
| Web-BCPA (39) | • Aged 18-63 years.<br>• Persistent musculoskeletal pain with duration of at least 3 months in the back, neck, shoulder, and/or generalised pain.<br>• OMPSQ score ≥90, screening for psychosocial factors that indicates an estimated risk for long-lasting pain and future disability (40).<br>• Work ability of at least 25% (assessment method N/R).<br>• Familiar with written and spoken Swedish.<br>• Internet and computer access. | • Participants must have spent at least 15 minutes per module in 5 of 8 modules.<br>• Participants had to have reached their 4-month follow-up assessment<br>• Participants contacted consecutively with information about interview study in conjunction with 4-month follow-up.<br>• Formal invitation subsequently via telephone. | • Website-based Web Behavior Change Program for Activity (Web-BCPA) in combination with MMR.<br>• Web-BCPA consisted of eight modules: 1) pain, 2) activity, 3) behavior, 4) stress and thoughts, 5) sleep and negative thoughts, 6) communication and self-esteem, 7) solutions, and 8) maintenance and progress.<br>• Modules contained information, assignments and exercises delivered as educational texts, videos and writing tasks.<br>• Participants could access 1 new module/week during the first 8 weeks of rehabilitation, and had access to the website 24/7 for 4 months. |

**HCP**: healthcare professional; **OP**: occupational physician; **OMPSQ**: Örebro musculoskeletal pain screening questionnaire; **MMR**: multimodal rehabilitation; **PA**: physical activity; **N/R**: not reported



For peer review only - http://bmjopen.bmj.com/site/about/guidelines.xhtml

Qualitative components of included studies

Sampling procedures used for the qualitative component of the included studies (Table 4) were described for three of the studies as an invitation to participants to take part in an interview (28, 29, 37) and for the fourth study, where the qualitative component was part of a self-administered survey, all participants took part (38). Qualitative interviews were conducted via telephone (37), in the participant's home (28, 29, 39), or at a local university (28, 29), health care centre (39) or council building (39). All of the interviews were semi-structured, recorded and either transcribed verbatim (28, 29, 39) or as written descriptions of answers including quotes (37). For the MyBehaviorCBP study (38), free-text answers from the electronic exit survey were extracted. Data was then analysed to identify common themes (28, 29, 37-39).

Quality appraisal

The comprehensiveness of reporting varied across the included studies (Supplementary File 2) and ranged from 12 (38%) to 21 (67%) of the 32-item COREQ checklist (28, 39). Items within domain 1 (Research team and reflexivity) generally had very poor reporting with several items not reported by any studies, for example researcher occupation and experience and training were not reported by any of the included studies. All studies reported sampling procedure, sample size, setting of data collection, description of sample, recording, derivation of themes, quotations presented, consistency of data and findings and clarity of major themes

Engagement strategies

We defined engagement strategies as any method used to recruit and initially motivate participants to enrol in the DHI study. The identified engagement strategies included: use of mailing lists of retired personnel (38); mailing list for a university wellness centre (38); or invitation from OP or HCP (28, 29, 37). In addition, the Oneself study advertised for participation through media: radio (project leader and managers interviewed about project at local radio station), television (rheumatologists involved in project spoke

For peer review only



For peer review only - http://bmjopen.bmj.com/site/about/guidelines.xhtml

about project on local television station), and through a press conference for which the major daily journals from the area were invited (28, 29).

Barriers and facilitators for uptake and utilisation of digital health interventions

We identified four major themes: 1) IT usability and accessibility, 2) Quality and amount of content, 3) Tailoring and personalisation, and 4) Motivation and support (Table 5). Under each theme, both barriers and facilitators were identified. Distinction between uptake (initial engagement) and utilisation (use) in the included studies was not possible, and they are therefore treated as one. Participant quotes are provided in the text to substantiate the data for each theme. More exemplar quotations are provided in Supplementary File 3.

**Table 5:** Factors affecting uptake and utilisation of DHIs for self-management of LBP

| Theme | Subtheme | Barriers | Facilitators |
|---|---|---|---|
| **IT usability and accessibility** | Functionality and usability | • Too much choice between functions<br>• Fixed advancement pace<br>• Issues logging into DHI<br>• *Low user-friendliness<br>• *Issues logging into DHI<br>• *Low level of functionality (e.g. registration, navigation, helpdesk) | • Flexible structure and navigation<br>• Conveniently arranged<br>• Variation of media types (text, audio and video)<br>• Reminders and notifications<br>• High user-friendliness<br>• *High user-friendliness |
| | IT affinity | • Lack of affinity with computers<br>• *Lack of affinity with web-based programmes | • Enjoying working with a computer |
| | Access and convenience | • Not able to choose starting time of DHI<br>• *No access to computer during consultation | • Easily accessible with low effort<br>• Accessible at all hours and locations<br>• Accessible even during periods with severe pain symptoms<br>• Ability to take all the time needed |
| **Quality and amount of content** | Quality of content | • Contradictory content between DHI and HCP | • Trustworthy content and source<br>• Easily understandable content |



For peer review only - http://bmjopen.bmj.com/site/about/guidelines.xhtml

| | | | |
|---|---|---|---|
| | | | • High quality of content<br>• Steady content<br>• *Appropriate content |
| | Amount of content | • Too much content to choose from<br>• Too much information to fully comprehend | • A lot of content to choose from |
| **Tailoring and personalisation** | Tailoring, specificity and personalisation | • Content not tailored to individual needs and/or pain severity<br>• Content perceived not new or relevant | • Content accounting for individual needs and/or pain severity<br>• Self-identification in content<br>• Opportunity to influence treatment |
| **Motivation and support** | Personal attributes and resources | • Adhering to biomedical model of LBP<br>• Seeing LBP as a marginal problem<br>• Preferring other treatment regimens, e.g. with human contact<br>• Lack of knowledge about LBP and treatments<br>• Physical health (e.g. pain, fatigue)<br>• Psychological symptoms | • High level of awareness and self-management of LBP<br>• Aware that LBP would not be fixed with a medical solution and ready to accept active role<br>• Emotional and cognitive resources, e.g. motivation, interest, commitment and self-confidence in self-management of LBP<br>• Enjoy solution focused work |
| | Support to use DHI | • HCP unsupportive of use of DHI<br>• No support from authorities | • HCP supportive of use of DHI<br>• Support from family<br>• Support from authorities<br>• Support from other suffers (e.g. successful testimonials) |
| | Features of DHI | • DHI not guiding or supporting participants enough (e.g. to plan for execution of physical activity recommendation from DHI) | • Interaction/interactivity<br>• Information about self-management of LBP<br>• Goal-setting<br>• Action-planning<br>• Follow-up and evaluation<br>• Adjusting treatment related to setbacks and progress<br>• Monitoring own progress in graphs<br>• Variation of content<br>• Update of content |
| | HCP factors for support of patients | • *Time restrictions of consultations<br>• *Difficulty keeping DHI in mind during consultations | • *DHI a good medium for counselling employees |



For peer review only - http://bmjopen.bmj.com/site/about/guidelines.xhtml

| | | | |
|---|---|---|---|
| | | • *Difficulty providing patients with accurate information about DHI<br>• *Perceiving no benefit of DHI compared to usual treatment<br>• *Preferring other treatment regimens, e.g. with human contact | |

*: Occupational physician perspective; **IT**: information technology; **HCP**: healthcare professional; **DHI**: Digital health intervention

*1) IT usability and accessibility*

The first theme that emerged concerned functionality and usability, IT affinity or access and convenience of the DHI. A flexible and convenient structure with high user-friendliness aided use of DHIs (37, 39). Inclusion of a variety of media types such as video was also appreciated (28, 29) as well as getting reminders or notifications from the DHI (28, 29).

*"Usually I went on the website when I read the newsletter. I read the letter and then I'm there, it's like a conditioned reflex (Woman, 49, nurse)"* (28, 29).

On the other hand, low user-friendliness and problems with logging in were barriers for use of DHIs for both study participants and HCPs (37). A fixed starting point or set advancement pace were also demotivating for some users (39). Affinity with computers and web-based programmes highly affected uptake of DHIs. Participants with a high level of computer affinity and who enjoyed working on a computer expressed positive feelings towards using DHIs (39), whereas lack of computer affinity was an important barrier for uptake of the intervention (37). Accessibility to a computer was surprisingly not a requirement for uptake to the study. When computers were readily available, DHIs were considered easy to access with unlimited 24h access (28, 29, 39).

*"… thanks to the program (the Web-BCPA) I was able to perform the basic body awareness exercises of my own choice… and to repeat those that I felt most effective as many times that I preferred… the flexibility made it mine (the rehabilitation) (Woman, participant)"* (39).



For peer review only - http://bmjopen.bmj.com/site/about/guidelines.xhtml

Even during periods with severe pain symptoms, a DHI was considered an attainable and effortless option as participants did not have to go anywhere (e.g. a healthcare centre) (28, 29, 39).

2) *Quality and amount of content*

Quality and amount of content provided in DHIs affected use for both participants and HCPs. Trustworthiness of the source and information provided facilitated use, and participants seemed to be reassured when knowing the content had been reviewed and validated by HCPs (28, 29, 39). For participants, richness and consistency of content facilitated use (28, 29), especially when the content was easily understandable (37).

*"Knowing that there is a serious website where there are contributions, it strengthens you a bit (Woman, 37, teacher"* (29).

Likewise, content that suited the patients was appreciated by HCPs (37). On the other hand, when participants experienced contradictory advice from their HCP and the DHI, this was a barrier for using the DHI (37). Large volumes of information or too much content to choose from also limited uptake and utilisation, particularly in relation to the amount of time required to go through it (28, 29, 37).

*"There is a lot of information, probably almost too much, don't you think? (Man, 47, bank director)"* (28, 29).

3) *Tailoring and personalisation*

The participants' perception of the degree of tailoring and personalisation of the content to their needs was the third major theme affecting use of DHIs for self-management of LBP. Self-identification increased utilisation of DHIs when participants were able to recognise themselves in the content, e.g. in the information and explanations about pain and symptoms, or thoughts related to dealing with LBP (28, 29, 39).



For peer review only - http://bmjopen.bmj.com/site/about/guidelines.xhtml

For peer review only

*"It gives you descriptions and you say: this stuff here... I see it, I see it! I recognise myself in it, I recognise myself here (Man, 58, teacher)"* (28, 29).

When the content of the DHI accounted for the individual participant's activities, needs or pain severity it further encouraged use of the DHI (37-39).

*"I really liked the personalization. I thought it was a nice touch. Suggestions were more specific and tailored, which for me made them more relevant and likely for me to use them (Participant)"* (38).

Participants appreciated the opportunity to influence their own rehabilitation by being able to select exactly what they wanted from a variety of options that fitted their situation (38, 39).

*"Previously I had read about CBT (Cognitive Behavioral Therapy), but I had never thought of it as a help for my condition... I want to compare this rehabilitation with a smorgasbord from which is it easy to taste (Participant)"* (39).

When content was not tailored to the individual participant or the participant's pain severity, it was experienced as a barrier for use of the DHI as it was not perceived to apply to their situation. This in turn would negatively impact the participant's motivation and sustained engagement (29, 37). Content that was not perceived relevant or new to the participant could also lead to a feeling of hopelessness as participants' got the impression that there was no solution to their problem (29).

4) *Motivation and support*

The fourth major theme related to the participants motivation and support, and included subthemes related to the personal attributes and resources of participants, support to use DHIs, features of DHIs, and lastly HCPs' perceptions and how they affect HCPs' support of DHIs. Specific participant attributes impacted the utilisation of DHIs; already being involved  or being ready to accept an active role in rehabilitation (28), and having motivation, interest, commitment and confidence in self-managing LBP facilitated use (28, 29, 39). Enjoying solution focused work, e.g. as experience from day job, was also a facilitator (39). Contrary, not wanting to take an active role (28), or preferring other treatment regimens (28) hindered use, as well as



For peer review only - http://bmjopen.bmj.com/site/about/guidelines.xhtml

lacking information about treatments (39) or preferring other available treatment regimens, e.g. with human contact (37). Relying on a HCP to find a solution (28, 29) or seeing LBP as only a marginal problem, led to lower motivation for use of the DHI (28). Furthermore, use of DHIs was constrained by physical (37, 39) or psychological (39) restrictions. Getting support from a variety of sources facilitated use; both support from outside and within the DHI. Support from family, authorities and HCPs was perceived as encouraging (39), and so were successful testimonials from other users whose LBP symptoms had improved (28, 29).

*"When you are going through a moment when you have backache and you read a testimony which says 'yes, there is someone who was able to do it', it gives you hope (Woman, 28, academic researcher)* (28, 29).

Not having HCPs or local agencies (e.g. authorities) support in their use of the DHI held participants back from utilising DHIs to manage their LBP (37, 39).

*"I expected more commitment from my OP [occupational physician] (Employee)"* (37).

Features of DHIs could both facilitate and restrain use. DHIs that were interactive, used goal-setting and action-planning, and had a great variation of content encouraged use (38, 39). Participants also appreciated information that guided them on how to self-manage their LBP (e.g. exercises and advice) (28, 29, 37-39), and some participants felt updates of content facilitated their use further (28, 29). Furthermore, DHIs that allowed participants to monitor and reflect on their own progress, improvement or goal attainment, e.g. through interactive graphs, were considered to enable self-management actions and to motivate further use (39). Follow-up and evaluation on goal achievement was also appreciated and reinforced the importance of tailoring DHIs towards individual participant's experience.

*" ... days when I had a lot of pain I used to remain sedentary, and as soon as I had a better day I was eager to do all kinds of activities that day.. before I started with the assignment activity planning (in the Web-BCPA) I was not aware of how my behaviour related to the days with pain, but by monitoring this over time I started to plan my daily activities in a more balanced way (Woman, participant)"* (39).

On the contrary, DHIs that did not support or guide participants enough, e.g. to execute recommendations given by the DHI, were perceived as constraining (38).



For peer review only - http://bmjopen.bmj.com/site/about/guidelines.xhtml

HCPs had reasons to support or not support participants' use of DHIs for self-management of LBP. HCPs either did not perceive additional benefits of DHIs compared to usual care or preferred other treatment regimens, e.g. ones that involved physical contact (37).

*"The ability to touch people is an essential element in the treatment of people with back or neck pain (Occupational physician)"* (37).

HCPs also reported having too little time during consultations to support use of DHI or difficulty in keeping the DHI in mind during their consultation – and even if they remembered it, they struggled with providing patients with accurate information about the DHI (37). However, HCPs who perceived DHIs as a good medium for counselling were positive about using and recommending DHIs (37).

Suggestions for improved utilisation

Participants of all included studies provided the authors with suggestions for how DHIs could be improved to facilitate continued or improved utilisation. As these items were only perceived as potential facilitators if implemented they are reported separately from the themes above. Some suggestions were improvement of usability of existing DHIs, e.g. increased user-friendliness (37), incorporation of illustrations and cartoons (37), or easier registration (37). Optimisation of tailoring to adjust for changes over time (37), or better adaption of physical activity recommendations that accommodated differences between weekdays and accounted for weather forecasts was also suggested (38). System improvements that enabled the DHI to learn from participants' activity level related to their pain days was also proposed (38). Lastly, application of a participatory approach for the process of designing DHIs was suggested (39). Other suggestions were new features to add to DHIs, e.g. direct contact to HCPs via DHI (37), a helpdesk  (37), content about how to deal with LBP mentally (37), and a sophisticated reminder system with just-in-time notifications for both planning and execution of physical activities (38).

Developing a conceptual understanding



For peer review only - http://bmjopen.bmj.com/site/about/guidelines.xhtml

We applied the NPT framework (Table 2) to the taxonomy of barriers and facilitators as summarised in Table 5. Most of the identified codes fell within the four NPT constructs, with the exception of codes related to participants' own physical, mental and emotional health, which although affecting an individual's capacity, they are not specific actionable tasks involved in the uptake and utilisation of a DHI for LBP. Applying the NPT framework allowed us to conceptualise how the codes identified may affect the uptake and utilisation of DHIs for the self-management of LBP (Figure 2), at both an individual and collective level, through the four stages of deciding whether to enrol, engage, utilise and maintain engagement with such a tool.

**Figure 2** Preliminary conceptual model of barriers and facilitators to uptake and utilisation of LBP DHIs

## Discussion

We have conducted a systematic search of the literature to explore the methods used to encourage participation with DHIs for the self-management of LBP and the barriers and facilitators to patient uptake and utilisation of these tools. Our review identified four studies published in five articles, demonstrating that the literature remains sparse.

Our review has enabled us to develop a preliminary conceptual model for engagement and utilisation of a DHI for LBP self-management by applying the NPT framework to the barriers and facilitators identified in the included studies. The model suggests that users value DHIs that are easily understandable, which they can navigate at their own pace and which help enhance subsequent communication with HCPs, family and colleagues. Providing regular updates and prompts appears to help users engage with DHIs whilst the ability to interact with other users is viewed positively in terms of providing support, motivation and validation. Users expect information to be easily accessible, structured, up-to-date and accurate, with tailoring to individual user experience being particularly valued.



For peer review only - http://bmjopen.bmj.com/site/about/guidelines.xhtml

Conversely, large volumes of information and lack of time appear to have a negative impact on user understanding, motivation and engagement. Lack of support or encouragement by HCPs also appears to be off putting for some whilst others face challenges accessing the DHIs. Participant's own attributes including the symptoms they experienced and their attitudes and preferences for treatment for LBP can further restrict capacity to self-manage and influence motivation and engagement with DHIs. Other significant barriers to user engagement and utilisation include missing or conflicting information, content that was not tailored to the individual, and lack of feedback or evaluation.

In this review we explored how studies engaged participants to enrol into the study and begin using a DHI, this was mainly through identification of potential participants and subsequent invitation. Sustaining engagement beyond initial participation was not discussed in-depth in any of the included studies, some used email prompts and regular updates or newsletters. However, all studies did report participants' suggestions to improve DHIs, which mainly focussed on improving usability, (dynamic) tailoring of content, additional features to support users and the inclusion of participants in the design of DHIs. While not considered as facilitators to uptake and utilisation, some positive consequences of using the DHIs were identified by some users, e.g. acquiring a vocabulary and an individual understanding of their situation, and increased confidence in self-managing their LBP, which may have reinforced users in their self-management and in turn may have increased use of DHIs. Further, some general points to increase utilisation of DHIs for LBP were highlighted by participants, including the importance of participatory involvement of patients in the development of a DHI.

Comparison with previous literature

Although there was a significant variation in intervention recruitment and content in studies included in our review, there was a large degree of overlap in terms of the barriers and facilitators identified. Many of these are generally in keeping with the findings of other qualitative reviews for DHIs in general (19, 41) as well as those looking specifically at hypertension (42) and pain management in older adults (43). A review



For peer review only - http://bmjopen.bmj.com/site/about/guidelines.xhtml

by O'Connor et al (19) identified four main themes relating to barriers and facilitators to engagement and recruitment to DHIs in general: personal agency and motivation; personal life and values; engagement and recruitment approach, and quality of the DHI. Another review by Hardiker & Grant (41) identified five overarching themes concerning barriers and facilitators influencing engagement with eHealth services: characteristics of users; technological issues; characteristics of eHealth services; social aspects of use; and eHealth services in use. Despite the differing terminology of the major theme headings used in these studies and those found in this review, comparison of the codes or subthemes reveals the barriers and facilitators to be broadly similar, suggesting that these may be generally transferable across DHIs. The main exception is the specific mention of security and privacy of personal information in these earlier reviews (19, 41), which was not found as a barrier in this review, although this may be due to the small number of studies in our review compared to O'Connor et al (19) and Hardiker & Grant (41), reviews which included 19 and 50 studies, respectively.

*Functionality and general IT issues*

Factors including age, ethnicity, economic status, level of educational attainment and familiarity with the internet are recognised as being significant factors influencing access to and engagement with DHIs (41). O'Connor et al. (19) reported that a lack of digital literacy, issues accessing IT equipment or the internet and the cost of such equipment or access are barriers to the use of DHIs. The user friendliness, design and ease of registration/logging in to a DHI were found to be significant issues for users in this review and should be carefully considered when planning a DHI.

*Quality and amount of content*

Trust is a significant issue when accessing information online (41). Clinical endorsement seems to be important to users in terms of the perceived quality of content and is in keeping with the findings of other studies in this area (19, 44). Additionally, consideration should be given to the potential for users to receive

For peer review only



For peer review only - http://bmjopen.bmj.com/site/about/guidelines.xhtml

contradictory advice from the DHI and their HCP. Our findings suggest that whilst some users considered large volumes of information as a barrier, others valued the ability to read widely on the subject. This is thought to reflect individual preference and personal factors such as time pressures. Taking such preferences into account during the development and delivery of DHIs may increase user engagement.

*Tailoring and personalisation*

It is clear from our findings that user's symptomology, prior knowledge and experience play a role in engagement. Tailoring DHIs to the user's individual symptoms and functional limitations is thought to enhance engagement (19) and may thus improve the effectiveness of the intervention. A recent review of DHIs for the self-management of LBP (17) found that no DHI for LBP used tailoring to enhance effectiveness, but commented that this could be an important means of enhancing engagement. In addition, O'Connor et al. (19) recommended that any DHI should be designed and tailored to individual needs in order to reduce the self-care burden. Our findings suggest that users improved understanding of LBP and enhanced communication with their HCP during subsequent consultations. Some users commented that they would have appreciated some direct support from a HCP or that this might have enhanced engagement. This finding is consistent with those of Steele et al (45), who during an evaluation of an internet-based physical activity behaviour change program, found that many participants in the internet group would have preferred traditional face-to-face sessions. Some of the occupational physician's interviewed felt that they did not have the time and capacity within their consultation to discuss DHI use in detail (37). If the intended purpose of a DHI is to facilitate HCP – patient communication then how the DHI or a supporting HCP dashboard could be designed to allow for efficient and useful interactions during a consultation should be considered at the design and development stage.

*Motivation and support*



For peer review only - http://bmjopen.bmj.com/site/about/guidelines.xhtml

Personal recommendations and social support were recognised as being important in encouraging DHI user registration and in fostering engagement (19). We found that some users valued the emotional support of being able to interact with other users. Whilst this was a positive finding in our study and is consistent with those reported elsewhere (41), there exists the possibility of potentially abusive or threatening behaviours developing online which could act as a barrier to some (46). Other reports of discussion threads deviating from the original topic or containing misleading information (41) raise questions on the need for monitoring such interactive features. Our findings further suggest that an individual's personal attributes and resources (e.g. emotional and cognitive) and attitudes towards self-management can influence their use of DHIs. Additional support may therefore be required for some potential users to participate and benefit from DHIs.

O'Connor et al (19) reported that some individuals do not view technology as a way of addressing healthcare needs and prefer alternative approaches to managing their health issues such as seeking support from family, friends or healthcare professionals. They also highlight the potential for DHIs to be impersonal and commented on the lack of a therapeutic relationship, particularly in situations where sensitive health or social issues are involved. Such views were also reflected among individuals, including some HCPs, in our findings. In contrast, other users appreciate the freedom to access health information at a time and place that suits the user along with the anonymity DHIs can offer (44), issues that can be challenging for traditional healthcare services to match.

Strengths and limitations

This systematic review was conducted by an experienced team and follows the PRISMA guidelines for the reporting of systematic reviews. Our iterative search strategy utilised multiple databases and involved independent data extraction, quality appraisal and data analysis by two reviewers, with a third reviewer adjudicating in the case of any disagreements.

For peer review only

Our review does however have some limitations. Many DHIs are developed commercially and do not undergo formal academic evaluation (15) resulting in relatively sparse literature in this area. Our search strategy involved several eligibility criteria, including that studies must be published in peer-reviewed academic journals, and as such we did not identify any grey literature. However, it is unlikely that such findings, if available, would have held scientific rigour and added to the findings of this review. Further, as our analysis and synthesis of data was based on reviewing published literature, not the original data, this could have impacted on the background context to some of the quotes used in this manuscript.

The studies included in this review (28, 29, 37-39) were conducted in real-life settings and as a result sampling procedures were acknowledged as being convenient, had the potential to be biased towards individuals who found the interventions beneficial and may not have been representative of all users. Furthermore, the literature contained very limited information on user's sociodemographic characteristics. However, as a consequence of the small number of studies identified by our search strategy, we did not exclude studies on the basis of quality, potentially reducing the reliability of the findings of this review.

Finally, due to the lack of literature in this field, our conceptual model for the update and utilisation of DHIs to support the self-management of LBP is limited to four studies to date. It is possible that not all the important barriers and facilitators may have been identified, and thus our conceptual model must be considered preliminary. As more rigorous studies are conducted and reported this model should be further developed and amended.. This information will be of particular use to those involved in designing and implementing DHIs focused on self-management of LBP and more widely.

**Conclusions**

For peer review only



For peer review only - http://bmjopen.bmj.com/site/about/guidelines.xhtml

Our systematic review highlights barriers and facilitators affecting the utilisation of DHIs for the self-management of LBP and identified key areas involved in embedding such interventions into everyday practice. The limited and varied quality of literature found by this review suggests that further primary research investigating the implementation of DHIs and user's experiences is required. Future research should aim to describe DHIs and their users in more detail and include descriptions of engagement strategies and barriers or facilitators encountered in order to enhance our knowledge of which approaches are likely to have the greatest impact on user engagement and outcomes, and for whom.

**List of abbreviations**

COREQ - Consolidated Criteria for Reporting Qualitative Research

DHI - Digital health intervention

HCP – Healthcare professional

IT - Information technology

LBP - Low back pain

NPT - Normalization process theory

OP - Occupational physician

PA – Physical activity

PRISMA - Preferred reporting items for systematic reviews and meta-analyses

**Supplementary File 1:** Search details, as previously described and published by Nicholl et al. (17)

**Supplementary File 2:** Consensus summary of quality appraisal as per 32-item COREQ checklist and comprehensiveness of reporting

**Supplementary File 3:** Taxonomy of barriers and facilitators with exemplar quotations



For peer review only - http://bmjopen.bmj.com/site/about/guidelines.xhtml

**Declarations**

Ethics approval and consent to participate: Not applicable

Consent for publication: Not applicable

Patient and public involvement: This research was done without patient involvement. Patients were not invited to comment on the study design and were not consulted to develop patient relevant outcomes or interpret the results. Patients were not invited to contribute to the writing or editing of this document for readability or accuracy.

Acknowledgements: We would like to thank our librarian adviser Ingrid Ingeborg Riphagen, Department of Public Health and Nursing, Faculty of Medicine and Health Sciences, Norwegian University of Science and Technology (NTNU).

**Contributorship statement**: Study design was developed by all authors. Title, abstract and full text screening was performed JK, MaS, KC and KW with any discrepancies being resolved by BN, CR and MeS. JK, MaS, BN and KW carried out data extraction. JK, KW and MaS conducted data synthesis and analysis aided by BN and FM. Quality appraisal was assessed by BN, KC and KW. LS, CR and MeS critically scrutinized first drafts and provided comments. All authors read, commented and approved the final manuscript.

**Competing interests:** The authors declare that they have no competing interests

**Funding**: This project has received funding from the European Union Horizon 2020 Research and innovation programme under grant agreement No 689040.

**Data sharing statement**: The datasets used and analysed during the current study are available from the corresponding author on reasonable request.

**References**

1. Hoy D, Bain C, Williams G, March L, Brooks P, Blyth F, et al. A systematic review of the global prevalence of low back pain. Arthritis and rheumatism. 2012;64(6):2028-37.

2. Vos T, Flaxman AD, Naghavi M, Lozano R, Michaud C, Ezzati M, et al. Years lived with disability (YLDs) for 1160 sequelae of 289 diseases and injuries 1990-2010: a systematic analysis for the Global Burden of Disease Study 2010. Lancet (London, England). 2012;380(9859):2163-96.

For peer review only



For peer review only - http://bmjopen.bmj.com/site/about/guidelines.xhtml

3. Nolet PS, Kristman VL, Cote P, Carroll LJ, Cassidy JD. Is low back pain associated with worse health-related quality of life 6 months later? European spine journal : official publication of the European Spine Society, the European Spinal Deformity Society, and the European Section of the Cervical Spine Research Society. 2015;24(3):458-66.
4. Dagenais S, Caro J, Haldeman S. A systematic review of low back pain cost of illness studies in the United States and internationally. The spine journal : official journal of the North American Spine Society. 2008;8(1):8-20.
5. Maniadakis N, Gray A. The economic burden of back pain in the UK. Pain. 2000;84(1):95-103.
6. (NICE) NIfHaCE. Low back pain and sciatica in over 16s: assessment and management: National Institute for Health and Care Excellence (NICE). 2016 [Available from: https://www.nice.org.uk/guidance/ng59.
7. Authority DH. National clinical guideline: Interventions for recent onset low back pain [in Danish]: Danish Health Authority; 2016 [Available from: https://www.sst.dk/da/udgivelser/2016/nkr-laenderygsmerter.
8. Sluijs EM, Kok GJ, van der Zee J. Correlates of exercise compliance in physical therapy. Physical therapy. 1993;73(11):771-82; discussion 83-6.
9. Newson JT, Huguet N, Ramage-Morin PL, McCarthy MJ, Bernier J, Kaplan MS, et al. Health behaviour changes after diagnosis of chronic illness among Canadians aged 50 or older. Health reports. 2012;23(4):49-53.
10. Murray E, Burns J, See TS, Lai R, Nazareth I. Interactive Health Communication Applications for people with chronic disease. The Cochrane database of systematic reviews. 2005(4):Cd004274.
11. Beatty L, Lambert S. A systematic review of internet-based self-help therapeutic interventions to improve distress and disease-control among adults with chronic health conditions. Clinical psychology review. 2013;33(4):609-22.
12. Zhao J, Freeman B, Li M. Can Mobile Phone Apps Influence People's Health Behavior Change? An Evidence Review. Journal of medical Internet research. 2016;18(11):e287.
13. Hayden JA, van Tulder MW, Tomlinson G. Systematic review: strategies for using exercise therapy to improve outcomes in chronic low back pain. Annals of internal medicine. 2005;142(9):776-85.
14. Strecher V. Internet methods for delivering behavioral and health-related interventions (eHealth). Annual review of clinical psychology. 2007;3:53-76.
15. Lalloo C, Jibb LA, Rivera J, Agarwal A, Stinson JN. "There's a Pain App for That": Review of Patient-targeted Smartphone Applications for Pain Management. The Clinical journal of pain. 2015;31(6):557-63.
16. Garg S, Garg D, Turin TC, Chowdhury MF. Web-Based Interventions for Chronic Back Pain: A Systematic Review. Journal of medical Internet research. 2016;18(7):e139.
17. Nicholl BI, Sandal LF, Stochkendahl MJ, McCallum M, Suresh N, Vasseljen O, et al. Digital Support Interventions for the Self-Management of Low Back Pain: A Systematic Review. Journal of medical Internet research. 2017;19(5):e179.
18. Machado GC, Pinheiro MB, Lee H, Ahmed OH, Hendrick P, Williams C, et al. Smartphone apps for the self-management of low back pain: A systematic review. Best practice & research Clinical rheumatology. 2016;30(6):1098-109.
19. O'Connor S, Hanlon P, O'Donnell CA, Garcia S, Glanville J, Mair FS. Understanding factors affecting patient and public engagement and recruitment to digital health interventions: a systematic review of qualitative studies. BMC medical informatics and decision making. 2016;16(1):120.
20. International Register of Systematic Reviews P. [Available from: https://www.crd.york.ac.uk/PROSPERO/display_record.php?ID=CRD42016051182.
21. Moher D, Liberati A, Tetzlaff J, Altman DG. Preferred reporting items for systematic reviews and meta-analyses: the PRISMA statement. International journal of surgery (London, England). 2010;8(5):336-41.
22. McLean G, Band R, Saunderson K, Hanlon P, Murray E, Little P, et al. Digital interventions to promote self-management in adults with hypertension systematic review and meta-analysis. Journal of hypertension. 2016;34(4):600-12.



For peer review only - http://bmjopen.bmj.com/site/about/guidelines.xhtml

23. Fleming T, Bavin L, Lucassen M, Stasiak K, Hopkins S, Merry S. Beyond the Trial: Systematic Review of Real-World Uptake and Engagement With Digital Self-Help Interventions for Depression, Low Mood, or Anxiety. Journal of medical Internet research. 2018;20(6):e199.

24. Carolan S, Harris PR, Cavanagh K. Improving Employee Well-Being and Effectiveness: Systematic Review and Meta-Analysis of Web-Based Psychological Interventions Delivered in the Workplace. Journal of medical Internet research. 2017;19(7):e271.

25. Booth A, Hannes K, Harden A, Noyes J, Harris J, Tong A. COREQ (Consolidated Criteria for Reporting Qualitative Studies). In: Moher D, Altman DG, Schulz KF, Simera I, Wager E, editors. Guidelines for reporting health research: A user's manual. Oxford: Wiley; 2014. p. 214-26.

26. Tong A, Sainsbury P, Craig J. Consolidated criteria for reporting qualitative research (COREQ): a 32-item checklist for interviews and focus groups. International journal for quality in health care : journal of the International Society for Quality in Health Care. 2007;19(6):349-57.

27. Dixon-Woods M, Sutton A, Shaw R, Miller T, Smith J, Young B, et al. Appraising qualitative research for inclusion in systematic reviews: a quantitative and qualitative comparison of three methods. J Health Serv Res Policy. 2007;12(1):42-7.

28. Schulz PJ, Rubinelli S, Zufferey MC, Hartung U. Coping with Chronic Lower Back Pain: Designing and Testing the Online Tool ONESELF. Journal of Computer-Mediated Communication. 2010;15(4):625-45.

29. Caiata Zufferey M, Schulz PJ. Self-management of chronic low back pain: an exploration of the impact of a patient-centered website. Patient education and counseling. 2009;77(1):27-32.

30. Thomas J, Harden A. Methods for the thematic synthesis of qualitative research in systematic reviews. BMC medical research methodology. 2008;8:45.

31. Graneheim UH, Lundman B. Qualitative content analysis in nursing research: concepts, procedures and measures to achieve trustworthiness. Nurse Education Today. 2004;24(2):105-12.

32. Krippendorff K. Content Analysis: An Introduction to Its Methodology. 2nd ed. Thousand Oaks, CA: SAGE Publications; 2004. 422 p.

33. May C, Finch T. Implementing, Embedding, and Integrating Practices: An Outline of Normalization Process Theory. Sociology. 2009;43(3):535-54.

34. May CR, Mair F, Finch T, MacFarlane A, Dowrick C, Treweek S, et al. Development of a theory of implementation and integration: Normalization Process Theory. Implementation Science. 2009;4(1):29.

35. Mair FS, May C, O'Donnell C, Finch T, Sullivan F, Murray E. Factors that promote or inhibit the implementation of e-health systems: an explanatory systematic review. Bulletin of the World Health Organization. 2012;90(5):357-64.

36. Gallacher K, Morrison D, Jani B, Macdonald S, May CR, Montori VM, et al. Uncovering treatment burden as a key concept for stroke care: a systematic review of qualitative research. PLoS medicine. 2013;10(6):e1001473.

37. de Jong T, Heinrich J, Blatter BM, Anema JR, van der Beek AJ. The feasibility of a web-based counselling program for occupational physicians and employees on sick leave due to back or neck pain. BMC medical informatics and decision making. 2009;9:46.

38. Rabbi M, Aung MS, Gay G, Reid MC, Choudhury T. Feasibility and Acceptability of Mobile Phone-Based Auto-Personalized Physical Activity Recommendations for Chronic Pain Self-Management: Pilot Study on Adults. Journal of medical Internet research. 2018;20(10):e10147-e.

39. Nordin C, Michaelson P, Eriksson MK, Gard G. It's About Me: Patients' Experiences of Patient Participation in the Web Behavior Change Program for Activity in Combination With Multimodal Pain Rehabilitation. Journal of medical Internet research. 2017;19(1):e22-e.

40. Linton S. Early assessment of psychological factors: the Örebro Screening Questionnaire for Pain. In: Linton S, editor. New avenues for the prevention of chronic musculoskeletal pain. Amsterdam: Elsevier; 2002.

41. Hardiker NR, Grant MJ. Factors that influence public engagement with eHealth: A literature review. International journal of medical informatics. 2011;80(1):1-12.



For peer review only

For peer review only - http://bmjopen.bmj.com/site/about/guidelines.xhtml

42. Band R, Bradbury K, Morton K, May C, Michie S, Mair FS, et al. Intervention planning for a digital intervention for self-management of hypertension: a theory-, evidence- and person-based approach. Implementation science : IS. 2017;12(1):25.
43. Parker SJ, Jessel S, Richardson JE, Reid MC. Older adults are mobile too!Identifying the barriers and facilitators to older adults' use of mHealth for pain management. BMC geriatrics. 2013;13:43.
44. Skinner H, Biscope S, Poland B. Quality of internet access: barrier behind internet use statistics. Social science & medicine (1982). 2003;57(5):875-80.
45. Steele R, Mummery KW, Dwyer T. Development and process evaluation of an internet-based physical activity behaviour change program. Patient education and counseling. 2007;67(1-2):127-36.
46. Horvath KJ, Danilenko GP, Williams ML, Simoni J, Amico KR, Oakes JM, et al. Technology use and reasons to participate in social networking health websites among people living with HIV in the US. AIDS and behavior. 2012;16(4):900-10.

For peer review only



For peer review only - http://bmjopen.bmj.com/site/about/guidelines.xhtml

# PRISMA 2009 Flow Diagram

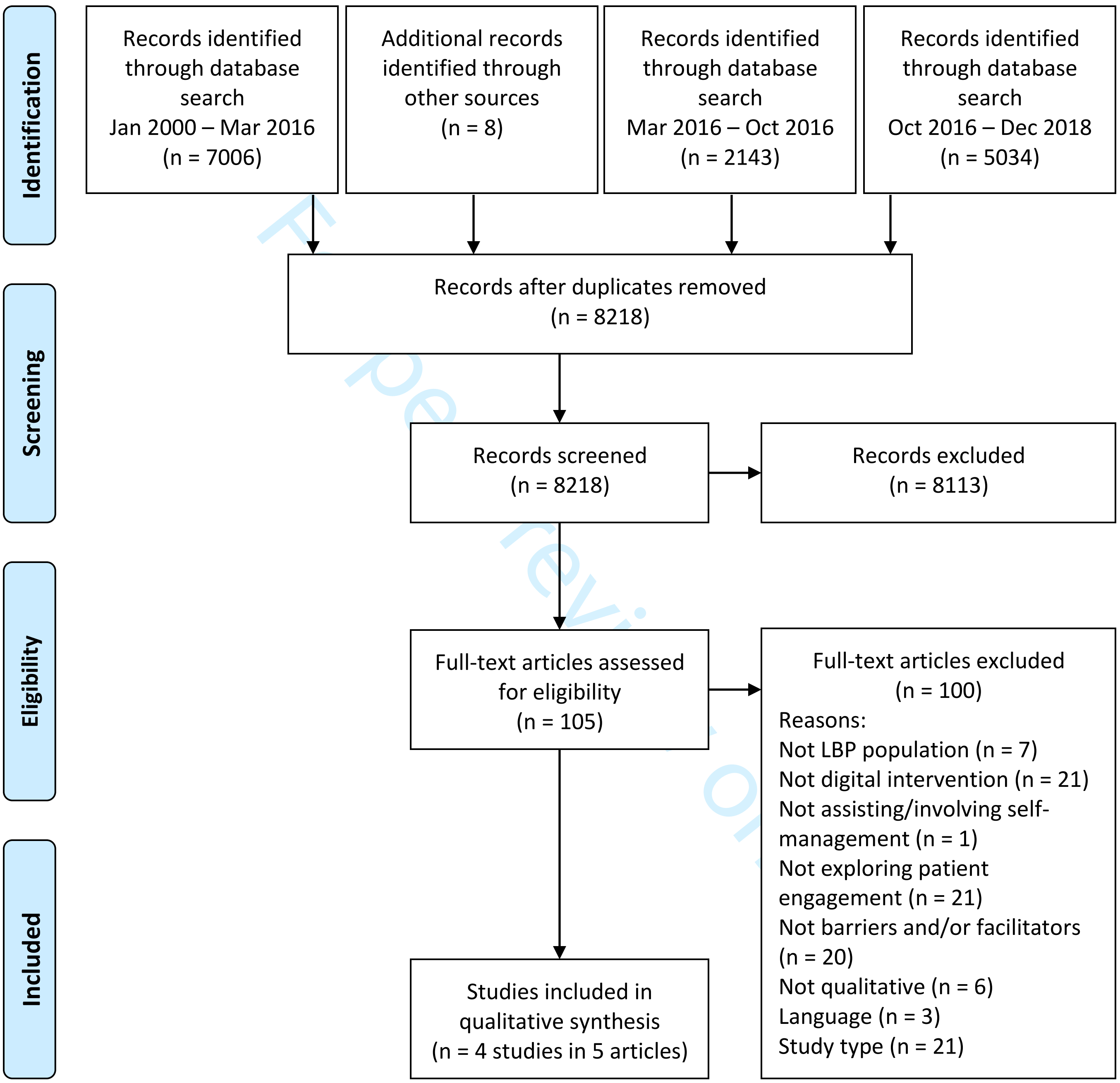


*From:* Moher D, Liberati A, Tetzlaff J, Altman DG, The PRISMA Group (2009). *P*referred *R*eporting *I*tems for *S*ystematic Reviews and *M*eta-*A*nalyses: The PRISMA Statement. PLoS Med 6(7): e1000097. doi:10.1371/journal.pmed1000097

**For more information, visit www.prisma-statement.org.**

For peer review only - http://bmjopen.bmj.com/site/about/guidelines.xhtml

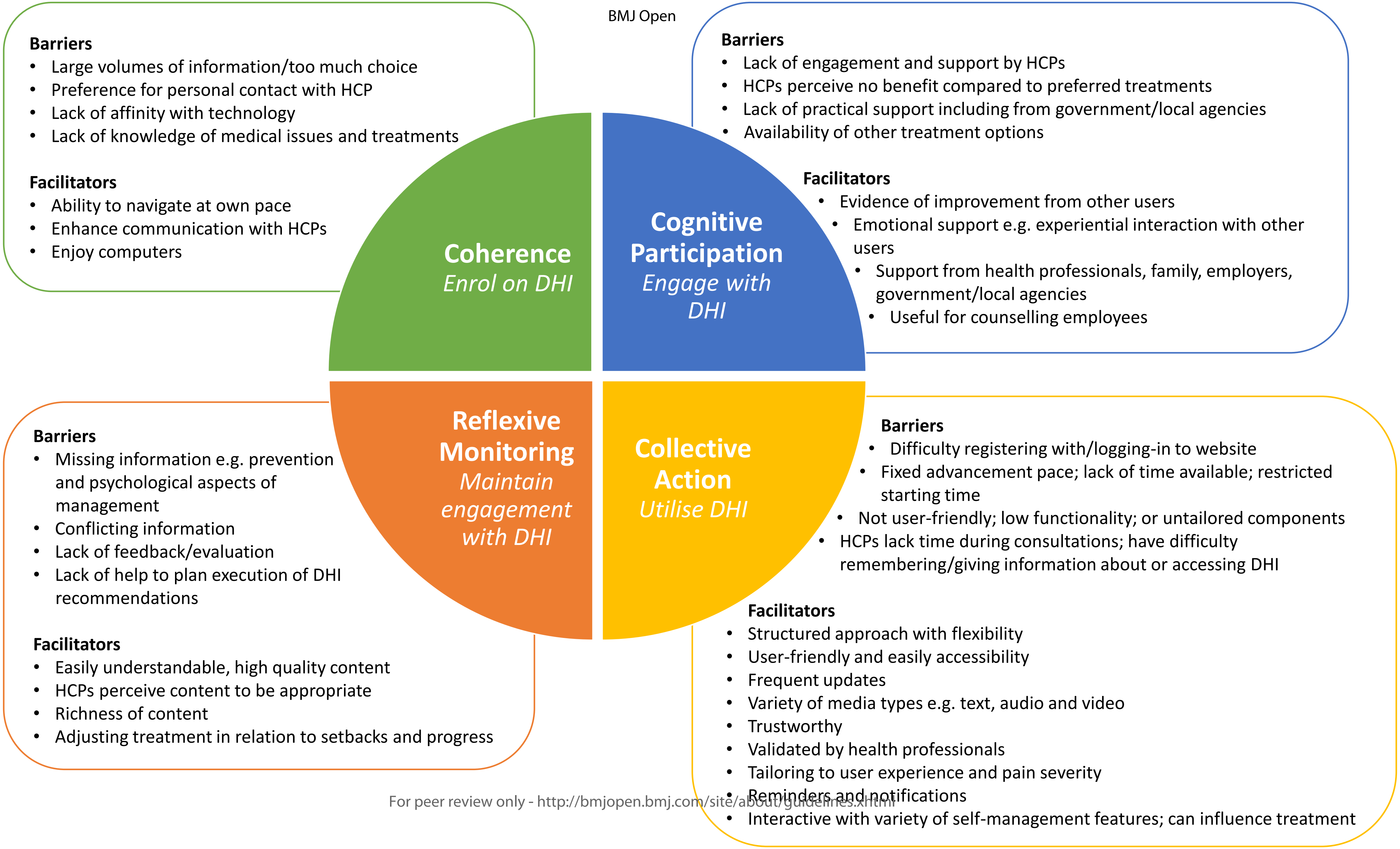
Barriers
Large volumes of information/too much choice
Preference for personal contact with HCP
Lack of affinity with technology
Lack of knowledge of medical issues and treatments
Facilitators
Ability to navigate at own pace
Enhance communication with HCPs
Enjoy computers
Coherence
Enrol on DHI
Cognitive Participation
Engage with DHI
Barriers
Lack of engagement and support by HCPs
HCPs perceive no benefit compared to preferred treatments
Lack of practical support including from government/local agencies
Availability of other treatment options
Facilitators
Evidence of improvement from other users
Emotional support e.g. experiential interaction with other users
Support from health professionals, family, employers, government/local agencies
Useful for counselling employees
Reflexive Monitoring
Maintain engagement with DHI
Barriers
Missing information e.g. prevention and psychological aspects of management
Conflicting information
Lack of feedback/evaluation
Lack of help to plan execution of DHI recommendations
Facilitators
Easily understandable, high quality content
HCPs perceive content to be appropriate
Richness of content
Adjusting treatment in relation to setbacks and progress
Collective Action
Utilise DHI
Barriers
Difficulty registering with/logging-in to website
Fixed advancement pace; lack of time available; restricted starting time
Not user-friendly; low functionality; or untailored components
HCPs lack time during consultations; have difficulty remembering/giving information about or accessing DHI
Facilitators
Structured approach with flexibility
User-friendly and easily accessibility
Frequent updates
Variety of media types e.g. text, audio and video
Trustworthy
Validated by health professionals
Tailoring to user experience and pain severity
Reminders and notifications
Interactive with variety of self-management features; can influence treatment

**Supplementary File 2:** Search details

## MEDLINE - search details

Ovid MEDLINE(R) 1946 to March Week 1 2016

| | |
|---|---|
| 1 | exp back pain/(back pain$ or lumbago or back ache$ or backache$ or (lumbar adj2 pain$) or (spin$ adj2 pain$)).ti,ab,kw,kf. |
| 2 | computer peripherals/ or computer storage devices/ or computer terminals/ or modems/ or microcomputers/ or computers, handheld/ or minicomputers/ or attitude to computers/ or computers/ or computer systems/ or medical informatics/ or medical informatics applications/ or educational technology/ or audiovisual aids/ or telecommunications/ or multimedia/ or computer-assisted instruction/ or user-computer interface/ or hypermedia/ or video games/ or electronic health records/ or social networking/ or (computer$ or microcomputer$ or pc or pcs or mac or macs or internet or www or web or website$ or webpage$ or local area network$).ti,ab,kf. or software.ti,ab,kf. or (cellular phone$ or cellular telephone$ or mobile$ or cell phone$ or cell telephone$ or smartphone$ or smart-phone$ or smart-telephone$).ti,ab,kf. or (handset$ or hand-set$ or wireless or wire-less or wifi or wi-fi or gps or global positioning system$ or bluetooth or text messag$ or texting or sms or short messag$ or multimedia messag$ or multi-media messag$ or mms or instant messag$ or social media$ or facebook or twitter or webcast$ or webinar$ or podcast$ or wiki or wikis or app or apps or android$ or blackberr$ or apple$ or ios or iphone$ or ipad$ or s40 or symbian$ or windows).ti,ab,kf. or ((electronic$ or digital$ or device$) adj2 tablet$).ti,ab,kf. or (video$ or dvd or dvds).ti,ab,kf. or (youtube or you tube or vimeo).ti,ab,kf. or (online or on line or interactive).ti,ab,kf. or (chat room$ or chatroom$).ti,ab,kf. or (blog$1 or web-log$1 or weblog$1).ti,ab,kf. or (bulletin board$ or bulletinboard$ or messageboard$ or message board$).ti,ab,kf. or (ehealth or e-health or mhealth or m-health).ti,ab,kf. or exp telemedicine/ or mobile applications/ or (pda or pdas or personal digital).ti,ab,kf. or device-based.ti,ab,kf. or (email$ or e-mail$ or electronic mail$).ti,ab,kw,kf. |
| 3 | 1 and 2 |
| 4 | limit 3 to yr="2000 -Current" |

Updated searches: 1) October 21 2016 (not shown), 2) December 18 2018 (below):

| | |
|---|---|
| 1 | exp back pain/(back pain$ or lumbago or back ache$ or backache$ or (lumbar adj2 pain$) or (spin$ adj2 pain$)).ti,ab,kw,kf. |
| 2 | computer peripherals/ or computer storage devices/ or computer terminals/ or modems/ or microcomputers/ or computers, handheld/ or minicomputers/ or attitude to computers/ or computers/ or computer systems/ or medical informatics/ or medical informatics applications/ or educational technology/ or audiovisual aids/ or telecommunications/ or multimedia/ or computer-assisted instruction/ or user-computer interface/ or hypermedia/ or video games/ or electronic health records/ or social networking/ |
| 3 | (computer$ or microcomputer$ or pc or pcs or mac or macs or internet or www or web or website$ or webpage$ or local area network$).ti,ab,kf. or software.ti,ab,kf. or (cellular phone$ or cellular telephone$ or mobile$ or cell phone$ or cell telephone$ or smartphone$ or smart-phone$ or smart-telephone$).ti,ab,kf. or (handset$ or hand-set$ or wireless or wire-less or wifi or wi-fi or gps or global positioning system$ or bluetooth or text messag$ or texting or sms or short messag$ or multimedia messag$ or multi-media messag$ or mms or instant messag$ or social media$ or facebook or twitter or webcast$ or webinar$ or podcast$ or wiki or wikis or app or apps or android$ or blackberr$ or apple$ or ios or iphone$ or ipad$ or s40 or symbian$ or windows).ti,ab,kf. or ((electronic$ or digital$ or device$) adj2 tablet$).ti,ab,kf. or (video$ or dvd or dvds).ti,ab,kf. or (youtube or you tube or vimeo).ti,ab,kf. or (online or on line or interactive).ti,ab,kf. or (chat room$ or chatroom$).ti,ab,kf. or (blog$1 or web-log$1 or weblog$1).ti,ab,kf. or (bulletin board$ or bulletinboard$ or messageboard$ or message board$).ti,ab,kf. or (ehealth or e-health or mhealth or m-health).ti,ab,kf. or exp telemedicine/ or mobile applications/ or (pda or pdas or personal digital).ti,ab,kf. or device-based.ti,ab,kf. or (email$ or e-mail$ or electronic mail$).ti,ab,kw,kf. |
| 4 | 1 and (2 or 3) |
| 5 | limit 4 to yr="2000 -Current" |
| 6 | 5 and (201610* or 201611* or 2017* or 2018*).ed. |

## Embase - search details

For peer review only

Ovid Embase (R) 1974 to 2016 March 18

| | |
|---|---|
| 1 | exp backache/ or (spinal pain$ or back pain$ or lumbago or back ache$ or backache$ or (lumbar adj2 pain$) or (spin$ adj2 pain$)).ti,ab,kw. |
| 2 | (exp backache/th or exp backache/pc or exp backache/rh or exp *backache/) not exp backache/su |
| 3 | exp communication protocol/ or computer assisted therapy/ or e-mail/ or human computer interaction/ or information technology/ or interactive voice response system/ or internet/ or mass communication/ or medical informatics/ or medical technology/ or mobile application/ or mobile phone/ or social media/ or exp telecommunication/ or exp telehealth/ or telephone/ or text messaging/ or webcast/ or wireless communication/ |
| 4 | computer storage device/ or computer terminal/ or microcomputer/ or minicomputer/ or attitude to computers/ or computer/ or computer system/ or medical information system/ or educational technology/ or audiovisual aid/ or exp multimedia/ or computer interface/ or hypermedia/ or electronic medical record/ or social networking/ |
| 5 | (computer$ or microcomputer$ or pc or pcs or mac or macs or internet or www or web or website$ or webpage$ or local area network$ or software or (cellular phone$ or cellular telephone$ or mobile$ or cell phone$ or cell telephone$ or smartphone$ or smart-phone$ or smart-telephone$) or (handset$ or hand-set$ or wireless or wire-less or wifi or wi-fi or gps or global positioning system$ or bluetooth or text messag$ or texting or sms or short messag$ or multimedia messag$ or multi-media messag$ or mms or instant messag$ or social media$ or facebook or twitter or webcast$ or webinar$ or podcast$ or wiki or wikis or app or apps or android$ or blackberr$ or apple$ or ios or iphone$ or ipad$ or s40 or symbian$ or windows) or ((electronic$ or digital$ or device$) adj2 tablet$) or (video$ or dvd or dvds) or (youtube or you tube or vimeo) or (online or on line or interactive) or (chat room$ or chatroom$) or (blog$1 or web-log$1 or weblog$1) or (bulletin board$ or bulletinboard$ or messageboard$ or message board$) or (ehealth or e-health or mhealth or m-health) or (app or apps) or (pda or pdas or personal digital) or device-based or (email$ or e-mail$ or electronic mail$)).ti,ab,kw. |
| 6 | 2 and 3 |
| 7 | limit 6 to yr="2000 -Current" |
| 8 | 1 and (3 or 4 or 5) |
| 9 | limit 8 to yr="2000 -Current" |
| 10 | 9 not 7 |

Updated searches: 1) October 21 2016 (not shown), 2) December 18 2018 (below):

| | |
|---|---|
| 1 | exp backache/ or (spinal pain$ or back pain$ or lumbago or back ache$ or backache$ or (lumbar adj2 pain$) or (spin$ adj2 pain$)).ti,ab,kw. |
| 2 | exp communication protocol/ or computer assisted therapy/ or e-mail/ or human computer interaction/ or information technology/ or interactive voice response system/ or internet/ or mass communication/ or medical informatics/ or medical technology/ or mobile application/ or mobile phone/ or social media/ or exp telecommunication/ or exp telehealth/ or telephone/ or text messaging/ or webcast/ or wireless communication/ |
| 3 | computer storage device/ or computer terminal/ or microcomputer/ or minicomputer/ or attitude to computers/ or computer/ or computer system/ or medical information system/ or educational technology/ or audiovisual aid/ or exp multimedia/ or computer interface/ or hypermedia/ or electronic medical record/ or social networking/ |
| 4 | (computer$ or microcomputer$ or pc or pcs or mac or macs or internet or www or web or website$ or webpage$ or local area network$ or software or (cellular phone$ or cellular telephone$ or mobile$ or cell phone$ or cell telephone$ or smartphone$ or smart-phone$ or smart-telephone$) or (handset$ or hand-set$ or wireless or wire-less or wifi or wi-fi or gps or global positioning system$ or bluetooth or text messag$ or texting or sms or short messag$ or multimedia messag$ or multi-media messag$ or mms or instant messag$ or social media$ or facebook or twitter or webcast$ or webinar$ or podcast$ or wiki or wikis or app or apps or android$ or blackberr$ or apple$ or ios or iphone$ or ipad$ or s40 or symbian$ or windows) or ((electronic$ or digital$ or device$) adj2 tablet$) or (video$ or dvd or dvds) or (youtube or you tube or vimeo) or (online or on line or interactive) or (chat room$ or chatroom$) or (blog$1 or web-log$1 or weblog$1) or (bulletin board$ or bulletinboard$ or messageboard$ or message board$) or (ehealth or e-health or mhealth or m-health) or (app or apps) or (pda or pdas or personal digital) or device-based or (email$ or e-mail$ or electronic mail$)).ti,ab,kw. |
| 5 | 1 and (2 or 3 or 4) |
| 6 | limit 5 to yr="2000 -Current" |

For peer review only

| | |
|---|---|
| 7 | limit 5 to yr="2016 -Current" |

**CINAHL - search details**

CINAHL (R) March 2016 through EBSCOhost

| | | |
|---|---|---|
| S6 | S1 AND S4 | |
| S5 | S1 AND S4 | |
| S4 | S2 OR S3 | |
| S3 | TI (computer* OR microcomputer* OR pc OR pcs OR mac OR macs OR internet OR www OR web OR website* OR webpage* OR "local area network*" OR software OR "cellular phone*" OR "cellular telephone*" OR mobile* OR "cell phone"* OR "cell telephone*" OR smartphone* OR smart-phone* OR smart-telephone* OR handset* OR hand-set* OR wireless OR wire-less OR wifi OR wi-fi OR gps OR "global positioning system*" OR bluetooth OR "text messag*" OR texting OR sms OR "short messag*" OR "multimedia messag*" OR "multi-media messag*" OR mms OR "instant messag*" OR "social media*" OR facebook OR twitter OR webcast* OR webinar* OR podcast* OR wiki OR wikis OR app OR apps OR android* OR blackberr* OR apple* OR ios OR iphone* OR ipad* OR s40 OR symbian* OR windows OR ((electronic* OR digital* OR device*) W2 tablet*) OR video* OR dvd OR dvds OR youtube OR "you tube" OR vimeo OR online OR "on line" or interactive OR "chat room*" OR chatroom* OR blog OR blogs OR web-log OR web-logs OR weblog OR weblogs OR "bulletin board*" OR bulletinboard$ OR messageboard$ OR "message board*" OR ehealth OR e-health OR mhealth OR m-health OR app OR apps OR pda OR pdas OR "personal digital" OR "device-based" OR email* OR e-mail* OR "electronic mail*") OR AB (computer* OR microcomputer* OR pc OR pcs OR mac OR macs OR internet OR www OR web OR website* OR webpage* OR "local area network*" OR software OR "cellular phone*" OR "cellular telephone*" OR mobile* OR "cell phone"* OR "cell telephone*" OR smartphone* OR smart-phone* OR smart-telephone* OR handset* OR hand-set* OR wireless OR wire-less OR wifi OR wi-fi OR gps OR "global positioning system*" OR bluetooth OR "text messag*" OR texting OR sms OR "short messag*" OR "multimedia messag*" OR "multi-media messag*" OR mms OR "instant messag*" OR "social media*" OR facebook OR twitter OR webcast* OR webinar* OR podcast* OR wiki OR wikis OR app OR apps OR android* OR blackberr* OR apple* OR ios OR iphone* OR ipad* OR s40 OR symbian* OR windows OR ((electronic* OR digital* OR device*) W2 tablet*) OR video* OR dvd OR dvds OR youtube OR "you tube" OR vimeo OR online OR "on line" or interactive OR "chat room*" OR chatroom* OR blog OR blogs OR web-log OR web-logs OR weblog OR weblogs OR "bulletin board*" OR bulletinboard$ OR messageboard$ OR "message board*" OR ehealth OR e-health OR mhealth OR m-health OR app OR apps OR pda OR pdas OR "personal digital" OR "device-based" OR email* OR e-mail* OR "electronic mail*")) | |

For peer review only

| | | |
|---|---|---|
| S2 | (MH "Computer peripherals") OR (MH "Computer storage devices") OR (MH "Computer terminals") OR (MH "Microcomputers") OR (MH "Computers, hand-held") OR (MH "Attitude to computers") OR (MH "Computer systems") OR (MH "Medical informatics") OR (MH "Educational technology") OR (MH "Audiovisuals") OR (MH "Audiorecording") OR (MH "Videorecording") OR (MH "Multimedia") OR (MH "Computer Environment") OR (MH "Computer Assisted Instruction") OR (MH "Hypermedia") OR (MH "Video games") OR (MH "Mobile applications") OR (MH "Patient record systems") OR (MH "Computerized patient record") OR (MH "") OR (MH "Computer communication networks+") OR (MH "Telecommunications") OR (MH "Electronic Bulletin Boards") OR (MH "Electronic Mail") OR (MH "Instant Messaging") OR (MH "Interactive Voice Response Systems") OR (MH "Text Messaging") OR (MH "Cellular Phone") OR (MH "Telephone") OR (MH "Internet+") OR (MH "Remote Consultation") OR (MH "Telemedicine") OR (MH "Telehealth") OR (MH "Telenursing") OR (MH "Smartphone") OR (MH "User-Computer Interface+") | |
| S1 | (MH "Back Pain+") OR TI ("spinal pain* " OR "back pain*" OR lumbago OR "back acke*" OR backache OR (lumbar W2 pain*) OR (spin* W2 pain*)) OR AB ("spinal pain* " OR "back pain*" OR lumbago OR "back acke*" OR backache OR (lumbar W2 pain*) OR (spin* W2 pain*)) | |

Updated searches: 1) October 21 2016, 2) December 18 2018

**Cochrane Library - search details** (Through Wiley Online Library)

- Cochrane Database of Systematic Review (CDSR)

For peer review only

- Database of Reviews of Systematic Reviews (DARE, discontinued)
- Central Cochrane Register of Controlled Trials (CENTRAL)
- ‘Method studies’
- ‘Technology assessments’
- ‘Economic evaluations’

| | |
|---|---|
| #1 | (spinal next pain* or back next pain* or lumbago or back next acke* or backache or (lumbar near/2 pain*) or (spin* near/2 pain*)):ti,ab,kw |
| #2 | (computer* or microcomputer* or "pc" or "pcs" or "mac" or "macs" or "internet" or "www" or "web" or website* or webpage* or local next area next network* or "software" or cellular next phone* or cellular next telephone* or mobile* or cell next phone* or cell next telephone* or smartphone* or smart-phone* or smart-telephone* or handset* or hand-set* or "wireless" or "wire-less" or "wifi" or "wi-fi" or "gps" or global next positioning next system* or "bluetooth" or text next messag* or "texting" or "sms" or short next messag* or multimedia next messag* or multi-media next messag* or "mms" or instant next messag* or social next media* or "facebook" or "twitter" or webcast* or webinar* or podcast* or "wiki" or "wikis" or "app" or "apps" or android* or blackberr* or apple* or "ios" or iphone* or ipad* or "s40" or symbian* or "windows" or ((electronic* or digital* or device*) near/2 tablet*) or video* or "dvd" or "dvds" or "youtube" or "you tube" or "vimeo" or "online" or "on line" or "interactive" or chat next room* or chatroom* or "blog" or "blogs" or "web-log" or "web-logs" or "weblog" or "weblogs" or bulletin next board* or bulletinboard* or messageboard* or message next board* or "ehealth" or "e-health" or "mhealth" or "m-health" or "app" or "apps" or "pda" or "pdas" or "personal digital" or "device-based" or email* or e-mail* or electronic next mail*):ti,ab,kw |
| #3 | #1 and #2 |

Updated searches: 1) October 21 2016 (not shown), 2) December 18 2018 (below):

| | |
|---|---|
| #1 | (spinal next pain* or back next pain* or lumbago or back next acke* or backache or (lumbar near/2 pain*) or (spin* near/2 pain*)):ti,ab,kw |
| #2 | (computer* or microcomputer* or "pc" or "pcs" or "mac" or "macs" or "internet" or "www" or "web" or website* or webpage* or local next area next network* or "software" or cellular next phone* or cellular next telephone* or mobile* or cell next phone* or cell next telephone* or smartphone* or smart-phone* or smart-telephone* or handset* or hand-set* or "wireless" or "wire-less" or "wifi" or "wi-fi" or "gps" or global next positioning next system* or "bluetooth" or text next messag* or "texting" or "sms" or short next messag* or multimedia next messag* or multi-media next messag* or "mms" or instant next messag* or social next media* or "facebook" or "twitter" or webcast* or webinar* or podcast* or "wiki" or "wikis" or "app" or "apps" or android* or blackberr* or apple* or "ios" or iphone* or ipad* or "s40" or symbian* or "windows" or ((electronic* or digital* or device*) near/2 tablet*) or video* or "dvd" or "dvds" or "youtube" or "you tube" or "vimeo" or "online" or "on line" or "interactive" or chat next room* or chatroom* or "blog" or "blogs" or "web-log" or "web-logs" or "weblog" or "weblogs" or bulletin next board* or bulletinboard* or messageboard* or message next board* or "ehealth" or "e-health" or "mhealth" or "m-health" or "app" or "apps" or "pda" or "pdas" or "personal digital" or "device-based" or email* or e-mail* or electronic next mail*):ti,ab,kw |
| #3 | #1 and #2 |

For peer review only

| | |
|---|---|
| | With Publication Year from 2016 to 2018, with Cochrane Library publication date from Jan 2016 to Dec 2018, in Trials |

| | |
|---|---|
| #1 | (spinal next pain* or back next pain* or lumbago or back next acke* or backache or (lumbar near/2 pain*) or (spin* near/2 pain*)):ti,ab,kw |
| #2 | (computer* or microcomputer* or "pc" or "pcs" or "mac" or "macs" or "internet" or "www" or "web" or website* or webpage* or local next area next network* or "software" or cellular next phone* or cellular next telephone* or mobile* or cell next phone* or cell next telephone* or smartphone* or smart-phone* or smart-telephone* or handset* or hand-set* or "wireless" or "wire-less" or "wifi" or "wi-fi" or "gps" or global next positioning next system* or "bluetooth" or text next messag* or "texting" or "sms" or short next messag* or multimedia next messag* or multi-media next messag* or "mms" or instant next messag* or social next media* or "facebook" or "twitter" or webcast* or webinar* or podcast* or "wiki" or "wikis" or "app" or "apps" or android* or blackberr* or apple* or "ios" or iphone* or ipad* or "s40" or symbian* or "windows" or ((electronic* or digital* or device*) near/2 tablet*) or video* or "dvd" or "dvds" or "youtube" or "you tube" or "vimeo" or "online" or "on line" or "interactive" or chat next room* or chatroom* or "blog" or "blogs" or "web-log" or "web-logs" or "weblog" or "weblogs" or bulletin next board* or bulletinboard* or messageboard* or message next board* or "ehealth" or "e-health" or "mhealth" or "m-health" or "app" or "apps" or "pda" or "pdas" or "personal digital" or "device-based" or email* or e-mail* or electronic next mail*):ti,ab,kw |
| #3 | #1 and #2 |
| | With Cochrane Library publication date from Jan 2016 to Dec 2018, in Cochrane Reviews and Cochrane Protocols |

For peer review only

**PsycINFO - search details**

Ovid PsycINFO (R) 1987 to March Week 4 2016

| | |
|---|---|
| 1 | exp back pain/ or (spinal pain$ or back pain$ or lumbago or back ache$ or backache$ or (lumbar adj2 pain$) or (spin$ adj2 pain$)).ti,ab,id. |
| 2 | exp Human Computer Interaction/ or Computer Peripheral Devices/ or Computer Software/ or Human Machine Systems/ or exp Electronic Communication/ or exp Computers/ or exp Mobile Devices/ or exp Internet/ or exp Computer Applications/ or Computer Attitudes/ or Information Technology/ or exp AUDIOVISUAL INSTRUCTION/ or exp AUDIOVISUAL COMMUNICATIONS MEDIA/ or exp EDUCATIONAL AUDIOVISUAL AIDS/ or Telecommunications Media/ or Multimedia/ or exp Social media/ or exp Telephone systems/ or Telemedicine/ or exp Websites/ or (computer$ or microcomputer$ or pc or pcs or mac or macs or internet or www or web or website$ or webpage$ or local area network$ or software or cellular phone$ or cellular telephone$ or mobile$ or cell phone$ or cell telephone$ or smartphone$ or smart-phone$ or smart-telephone$ or handset$ or hand-set$ or wireless or wire-less or wifi or wi-fi or gps or global positioning system$ or bluetooth or text messag$ or texting or sms or short messag$ or multimedia messag$ or multi-media messag$ or mms or instant messag$ or social media$ or facebook or twitter or webcast$ or webinar$ or podcast$ or wiki or wikis or app or apps or android$ or blackberr$ or apple$ or ios or iphone$ or ipad$ or s40 or symbian$ or windows or ((electronic$ or digital$ or device$) adj2 tablet$) or video$ or dvd or dvds or youtube or you tube or vimeo or online or on line or interactive or chat room$ or chatroom$ or blog$1 or web-log$1 or weblog$1 or bulletin board$ or bulletinboard$ or messageboard$ or message board$ or ehealth or e-health or mhealth or m-health or app or apps or pda or pdas or personal digital or device-based or email$ or e-mail$ or electronic mail$).ti,ab,id. |
| 3 | 1 and 2 |
| 4 | limit 3 to yr="2000 -Current" |

Updated searches: 1) October 21 2016 (not shown), 2) December 18 2018 (below):

| | |
|---|---|
| 1 | exp back pain/ or (spinal pain$ or back pain$ or lumbago or back ache$ or backache$ or (lumbar adj2 pain$) or (spin$ adj2 pain$)).ti,ab,id. |
| 2 | exp Human Computer Interaction/ or Computer Peripheral Devices/ or Computer Software/ or Human Machine Systems/ or exp Electronic Communication/ or exp Computers/ or exp Mobile Devices/ or exp Internet/ or exp Computer Applications/ or Computer Attitudes/ or Information Technology/ or exp AUDIOVISUAL INSTRUCTION/ or exp AUDIOVISUAL COMMUNICATIONS MEDIA/ or exp EDUCATIONAL AUDIOVISUAL AIDS/ or Telecommunications Media/ or Multimedia/ or exp Social media/ or exp Telephone systems/ or Telemedicine/ or exp Websites/ |
| 3 | (computer$ or microcomputer$ or pc or pcs or mac or macs or internet or www or web or website$ or webpage$ or local area network$ or software or cellular phone$ or cellular telephone$ or mobile$ or cell phone$ or cell telephone$ or smartphone$ or smart-phone$ or smart-telephone$ or handset$ or hand-set$ or wireless or wire-less or wifi or wi-fi or gps or global positioning system$ or bluetooth or text messag$ or texting or sms or short messag$ or multimedia messag$ or multi-media messag$ or mms or instant messag$ or social media$ or facebook or twitter or webcast$ or webinar$ or podcast$ or wiki or wikis or app or apps or android$ or blackberr$ or apple$ or ios or iphone$ or ipad$ or s40 or symbian$ or windows or ((electronic$ or digital$ or device$) adj2 tablet$) or video$ or dvd or dvds or youtube or you tube or vimeo or online or on line or interactive or chat room$ or chatroom$ or blog$1 or web-log$1 or weblog$1 or bulletin board$ or bulletinboard$ or messageboard$ or message board$ or ehealth or e-health or mhealth or m-health or app or apps or pda or pdas or personal digital or device-based or email$ or e-mail$ or electronic mail$).ti,ab,id. |
| 4 | 1 and (2 or 3) |
| 5 | limit 4 to yr="2000 -Current" |
| 6 | 5 and (20161* or 2017* or 2018* or 2019*).up. |

**DoPHER - search details**

Database of Promoting Health Effectiveness Reviews
Focussed coverage of systematic and non-systematic reviews of effectiveness in health promotion and public health worldwide (3700).

Search date 11.04.2016

| | |
|---|---|
| 1 | Freetext (Year): >1999 |
| 2 | Freetext (All but Authors): “spinal pain” OR “back pain” OR “spinal pains” OR “back pains” OR lumbago OR “back ache” OR “back aches” OR “backache*” |
| 3 | 1 AND 2 |

Updated searches: 1) October 21 2016, 2) December 18 2018

**TROPHI - search details**

Trials Register of Promoting Health Interventions
Focussed coverage of trials of interventions in health promotion and public health worldwide. It covers both randomised and non-randomised controlled trials and currently contains details of over 7,750 trials.

Search date 11.04.2016

For peer review only - http://bmjopen.bmj.com/site/about/guidelines.xhtml

| | |
|---|---|
| 5 | Freetext (All but Authors): “spinal pain” OR “back pain” OR “spinal pains” OR “back pains” OR lumbago OR “back ache” OR “back aches” OR “backache*” |
| 6 | Freetext (Year): >1999 |
| 7 | 5 AND 6 |

Updated searches: 1) October 21 2016, 2) December 18 2018

**Web of Science - search details**

(Thomson Reuters)

Databases selected:

- Science Citation Index (SCI Expanded)
- Social Science Citation Index (SSCI)
- Conference Proceedings Citation Index – Science (CPCI-S)
- Conference Proceedings Citation Index – Social Science (SPCI-SSH)

Search date 6.4.2016

| | |
|---|---|
| #3 | #2 AND #1<br>*Indexes=SCI-EXPANDED, SSCI, CPCI-S, CPCI-SSH Timespan=2000-2016* |
| #2 | **TOPIC:** (computer$ OR microcomputer* OR pc OR pcs OR mac OR macs OR internet OR www OR web OR website* OR webpage* OR "local area network*" OR software OR "cellular phone*" OR "cellular telephone*" OR mobile* OR "cell phone*" OR "cell telephone*" OR smartphone* OR smart-phone* OR smart-telephone* OR handset* OR hand-set* OR wireless OR wire-less OR wifi OR wi-fi OR gps OR "global positioning system*" OR bluetooth OR "text messag*" OR texting OR sms OR "short messag*" OR "multimedia messag*" OR "multi-media messag*" OR mms OR "instant messag*" OR "social media*" OR facebook OR twitter OR webcast* OR webinar* OR podcast* OR wiki OR wikis OR app OR apps OR android* OR blackberr* OR apple* OR ios OR iphone* OR ipad* OR s40 OR symbian* OR windows OR ((electronic* OR digital* OR device*) NEAR/2 tablet*) OR video* OR dvd OR dvds OR youtube OR "you tube" OR vimeo OR online OR "on line" OR interactive OR "chat room*" OR chatroom* OR blog OR blogs OR web-log OR web-logs OR weblog OR weblogs OR "bulletin board*" OR bulletinboard* OR messageboard* OR "message board*" OR ehealth OR e-health OR mhealth OR m-health OR pda OR pdas OR "personal digital" OR "device-based" OR email* OR e-mail* OR "electronic mail*")<br>*Indexes=SCI-EXPANDED, SSCI, CPCI-S, CPCI-SSH Timespan=2000-2016* |
| #1 | **TOPIC:** ("spinal pain*" OR "back pain*" OR lumbago OR "back ache*" OR backache* OR lumbar NEAR/2 pain* OR spin* NEAR/2 pain*)<br>*Indexes=SCI-EXPANDED, SSCI, CPCI-S, CPCI-SSH Timespan=2000-2016* |

Updated searches: 1) October 21 2016, 2) December 18 2018

**OT Seeker - search details**

Occupational therapy systematic evaluation of evidence.
http://www.otseeker.com/Search/BasicSearch.aspx

| | |
|---|---|
| 1 | back pain AND (internet OR web) |

Updated searches: 1) October 21 2016, 2) December 18 2018

For peer review only

**Supplementary File 2:** Consensus summary of quality appraisal as per the 32-item Consolidated Criteria for Reporting Qualitative Research (COREQ) checklist (Booth et al., 2014; Tong et al., 2007) and comprehensiveness of reporting.

| No | Item | Guide questions | de Jong et al., 2009 | Caiata Zufferey & Schulz, 2009 | Schulz et al., 2010 | Nordin et al., 2017 | Rabbi et al., 2018 | Number of articles reporting each item (%) |
|---|---|---|---|---|---|---|---|---|
| **Domain 1: Research team and reflexivity** | | | | | | | | |
| **Personal characteristics** | | | | | | | | |
| **1** | Interviewer/facilitator | Which author/s conducted the interview or focus group? | N/R | N/R | N/R | Principal author | N/R | 1 (20%) |
| **2** | Credentials | What were the researcher's credentials? E.g. PhD, MD | N/R | N/R | N/R | PhD | PhD, PhD and MD | 2 (40%) |
| **3** | Occupation | What was their occupation at the time of the study? | N/R | N/R | N/R | N/R | N/R | 0 (0%) |
| **4** | Gender | Was the researcher male or female? | N/R | N/R | N/R | Female | N/R | 1 (20%) |
| **5** | Experience and training | What experience or training did the researcher have? | N/R | N/R | N/R | N/R | N/R | 0 (0%) |
| **Relationship with participants** | | | | | | | | |
| **6** | Relationship established | Was a relationship established prior to study commencement? | N/R | N/R | N/R | Participants had participated in the RCT, of which the qualitative study was a later part | N/R | 1 (20%) |
| **7** | Participant knowledge of the interviewer | What did the participants know about the researcher? e.g. | N/R | N/R | N/R | N/R | N/R | 0 (0%) |

For peer review only

| | | | | | | | | |
|---|---|---|---|---|---|---|---|---|
| | | personal goals, reasons for doing the research | | | | | | |
| **8** | Interviewer characteristics | What characteristics were reported about the interviewer/facilitator? e.g. Bias, assumptions, reasons and interests in the research topic | N/R | N/R | N/R | N/R | N/R | 0 (0%) |
| **Domain 2: Study design** | | | | | | | | |
| **Theoretical framework** | | | | | | | | |
| **9** | Methodological orientation and theory | What methodological orientation was stated to underpin the study? e.g. grounded theory, discourse analysis, ethnography, phenomenology, content analysis | N/R | Grounded theory | N/R | Content Analysis | N/R | 2 (40%) |
| **Participant selection** | | | | | | | | |
| **10** | Sampling | How were participants selected? e.g. purposive, convenience, consecutive, snowball | Consecutively | Purposive and convenience | Purposive and convenience | Convenience | No selection, all participants of the DHI took part. | 5 (100%) |
| **11** | Method of approach | How were participants approached? e.g. face-to-face, telephone, mail, email | N/R | Email | Email | First approach not clear, but once given oral consent contacted by telephone | Method of sending invitations not clear. If eligible face-to-face meeting | 4 (80%) |
| **12** | Sample size | How many participants were in the study? | 11 OPs who recruited; 8 OPs who did not recruit & 9 employees | 18 | 18 | 19 | 10 | 5 (100%) |

For peer review only

| | | | | | | | | |
|---|---|---|---|---|---|---|---|---|
| **13** | Non-participation | How many people refused to participate or dropped out? Reasons? | 7 OPs who did not recruit; 15 employees. Reasons - no time, insufficient use of program, problems with recalling experiences | 238 approached to participate; 32 responded; 14 of these did not participate – reasons not stated | N/R | 3 – reasons not stated | None | 4 (80%) |
| **Setting** | | | | | | | | |
| **14** | Setting of data collection | Where was the data collected? e.g. home, clinic, workplace | Telephone interviews | Home or University | Home or University | Health Care Centres, County City Buildings, Participant's home | Web-based exit survey | 5 (100%) |
| **15** | Presence of non-participants | Was anyone else present besides the participants and researchers? | N/R | N/R | N/R | N/R | N/R | 0 (0%) |
| **16** | Description of sample | What are the important characteristics of the sample? e.g. demographic data, date | Not stated for OPs; Employees 67% male; 40-50 years; 75% LBP; white & blue-collar workers; varying educational levels; varying sickness absence levels due to LBP | 9 females, 9 males; 28-72 years; chronic LBP for 1-30 years; mix of diagnoses including 8 with no clear diagnosis; all had at least secondary school education (5 had degree); | 9 females, 9 males; 28-72 years; chronic LBP 1-30 years ; mixed diagnoses, varied level of education and frequency of website use | 15 females, 4 males; mean age 45; MSK pain for average 7.5 years; most at least secondary education; majority working. | 7 females, 3 males; 31-60 years; chronic LBP 5-33 years duration; mixed diagnoses. | 5 (100%) |

For peer review only

| | | | | | | | | |
|---|---|---|---|---|---|---|---|---|
| | | | 7weeks-6 months | range of website use amongst participants | | | | |
| **Data collection** | | | | | | | | |
| **17** | Interview guide | Were questions, prompts, guides provided by the authors? Was it pilot tested? | Topic guides used. Pilot tested | No questions, prompts or guides provided; Piloting not reported | No questions, prompts or guides provided; Piloting not reported | No questions, prompts or guides provided; Piloting not reported | Open-ended question in web survey provided. Piloting not reported | 2 (40%) |
| **18** | Repeat interviews | Were repeat interviews carried out? If yes, how many? | N/R | N/R | N/R | N/R | N/R | 0 (0%) |
| **19** | Audio/visual recording | Did the research use audio or visual recording to collect the data? | Audio recorded | Not specifically stated "Recorded" and transcribed verbatim | Audio recorded | Audio recorded | No – used free text web survey | 5 (100%) |
| **20** | Field notes | Were field notes made during and/or after the interview or focus group? | N/R | N/R | N/R | N/R | N/R | 0 (0%) |
| **21** | Duration | What was the duration of the interviews or focus group? | Approx. 30 minutes | Approx. 45 minutes | Approx. 45 minutes | 31 – 56 minutes. Mean 48 minutes | N/R | 4 (80%) |
| **22** | Data saturation | Was data saturation discussed? | Yes | Yes | N/R | N/R | N/R | 2 (40%) |
| **23** | Transcripts returned | Were transcripts returned to participants for comment and/or correction? | N/R | N/R | N/R | N/R | N/R | 0 (0%) |
| **Domain 3: Analysis and findings** | | | | | | | | |

For peer review only

| | | | | | | | | |
|---|---|---|---|---|---|---|---|---|
| **Data analysis** | | | | | | | | |
| **24** | Number of data coders | How many data coders coded the data? | N/R | N/R | N/R | 4 | N/R | 1 (20%) |
| **25** | Description of coding tree | Did authors provide a description of the coding tree? | N/R | N/R | N/R | Yes | N/R | 1 (20%) |
| **26** | Derivation of themes | Were themes identified in advance or derived from the data? | Derived from data | Derived from data | Essentially inductive | Derived from data | Derived from data | 5 (100%) |
| **27** | Software | What software, if applicable, was used to manage the data? | Excel | ATLAS.ti | ATLAS.ti | Open Code | N/R | 4 (80%) |
| **28** | Participant checking | Did participants provide feedback on the findings? | N/R | N/R | N/R | N/R | N/R | 0 (0%) |
| **Reporting** | | | | | | | | |
| **29** | Quotations presented | Were participant quotations presented to illustrate the themes / findings? Was each quotation identified? e.g. participant number | Few direct quotes; only identified as either OP or employee | Yes - identified by gender, age & occupation | Yes - identified by gender, age & occupation | Yes – identified by participant number and gender | Yes – identified by participant number | 5 (100%) |
| **30** | Data and findings consistent | Was there consistency between the data presented and the findings? | A little unclear – little qualitative data presented | Yes | Yes | Yes | Yes | 5 (100%) |
| **31** | Clarity of major themes | Were major themes clearly presented in the findings? | Yes | Yes | Yes | Yes | Yes | 5 (100%) |
| **32** | Clarity of minor themes | Is there a description of diverse cases or discussion of minor themes? | Not clear | Range of themes presented but not clear what is major/minor | Range of themes presented but not clear what is major/minor | Yes | Yes | 2 (40%) |

For peer review only

| | | | | | | |
|---|---|---|---|---|---|---|
| **TOTAL, number (%)** | 14 (44%) | 15 (47%) | 12 (38%) | 21 (67%) | 14 (44%) | |
| **DHI: digital health intervention; LBP: low back pain; N/R: not reported; OPs: occupational physicians;** | | | | | | |

## References

Booth A, Hannes K, Harden A, Noyes J, Harris J, Tong A. COREQ (Consolidated Criteria for Reporting Qualitative Studies). In: Moher D, Altman DG, Schulz KF, Simera I, Wager E, editors. Guidelines for reporting health research: A user's manual. Oxford: Wiley; 2014. p. 214-26.

Caiata Zufferey M, Schulz PJ. Self-management of chronic low back pain: an exploration of the impact of a patient-centered website. Patient education and counseling. 2009;77(1):27-32.

de Jong T, Heinrich J, Blatter BM, Anema JR, van der Beek AJ. The feasibility of a web-based counselling program for occupational physicians and employees on sick leave due to back or neck pain. BMC medical informatics and decision making. 2009;9:46.

Nordin C, Michaelson P, Eriksson MK, Gard G. It's About Me: Patients' Experiences of Patient Participation in the Web Behavior Change Program for Activity in Combination With Multimodal Pain Rehabilitation. Journal of medical Internet research. 2017;19(1):e22-e.

Rabbi M, Aung MS, Gay G, Reid MC, Choudhury T. Feasibility and Acceptability of Mobile Phone-Based Auto-Personalized Physical Activity Recommendations for Chronic Pain Self-Management: Pilot Study on Adults. Journal of medical Internet research. 2018;20(10):e10147-e.

Schulz PJ, Rubinelli S, Zufferey MC, Hartung U. Coping with Chronic Lower Back Pain: Designing and Testing the Online Tool ONESELF. Journal of Computer-Mediated Communication. 2010;15(4):625-45.

Tong A, Sainsbury P, Craig J. Consolidated criteria for reporting qualitative research (COREQ): a 32-item checklist for interviews and focus groups. International journal for quality in health care : journal of the International Society for Quality in Health Care. 2007;19(6):349-57.

For peer review only

**Supplementary File 3:** Taxonomy of barriers and facilitators with exemplar quotations

**References**:

[27] Schulz PJ, Rubinelli S, Zufferey MC, Hartung U. Coping with Chronic Lower Back Pain: Designing and Testing the Online Tool ONESELF. Journal of Computer-Mediated Communication. 2010;15(4):625-45.
[28] Caiata Zufferey M, Schulz PJ. Self-management of chronic low back pain: an exploration of the impact of a patient-centered website. Patient education and counseling. 2009;77(1):27-32.
[36] de Jong T, Heinrich J, Blatter BM, Anema JR, van der Beek AJ. The feasibility of a web-based counselling program for occupational physicians and employees on sick leave due to back or neck pain. BMC medical informatics and decision making. 2009;9:46.
[37] Rabbi M, Aung MS, Gay G, Reid MC, Choudhury T. Feasibility and Acceptability of Mobile Phone-Based Auto-Personalized Physical Activity Recommendations for Chronic Pain Self-Management: Pilot Study on Adults. Journal of medical Internet research. 2018;20(10):e10147-e.
[38] Nordin C, Michaelson P, Eriksson MK, Gard G. It's About Me: Patients' Experiences of Patient Participation in the Web Behavior Change Program for Activity in Combination With Multimodal Pain Rehabilitation. Journal of medical Internet research. 2017;19(1):e22-e.

| **Barriers and facilitators for patient uptake and utilisation of digital self-management interventions for LBP** | | | | | |
|---|---|---|---|---|---|
| **Theme** | **Taxonomy** | **Barriers** | **Exemplar quotations** | **Facilitators** | **Exemplar quotations** |
| IT usability and accessibility | Functionality and usability | • Too much choice between functions<br>• Fixed advancement pace<br>• Issues logging into DHI<br>• *Low user-friendliness<br>• *Issues logging into DHI<br>• *Low level of functionality (e.g. registration, navigation, helpdesk) | • Though, the freedom of choice in the Web-BCPA entailed perceptions of restrained patient participation for some informants [38, p4]<br>• Finally, some OPs faced practical obstacles such as log-in problems […] [36, p5]<br>• Although OPs were generally positive about the user-friendliness and | • Flexible structure and navigation<br>• Conveniently arranged<br>• Variation of media types (text, audio and video)<br>• Reminders and notifications<br>• High user-friendliness<br>• *High user-friendliness | • *I liked this thing about the exercise video a lot because seeing it with the video gives you a lot more. They seem simple, but a lot of times when there are drawings I can't understand them easily, then I don't have the will anymore [28, p29]*<br>• It was enough to open the mailbox for reasons that could be independent of cLBP |

| | | | | | |
|---|---|---|---|---|---|
| | | | design of the program, some felt that further improving user-friendliness (functionality) might enhance its use. […] It should also be easier to register employees in the program [36, p6]<br>• Although, some informants perceived restrained patient participation by the fact that […] not being able to select a faster advancement in the program by themselves [36, p5]<br>• A small number of employees either had problems with 'logging in into the program' […] [36, p6] | | to get a reminder of the website and the necessity of self-management […] *I usually went on the website when I read the newsletter. I read the letter and then I'm there, it's like a conditioned reflex [27, p641]*<br>• *It would be helpful to have reminders and suggestions pop up in the morning or at other chosen times. This could be optional and set by the user [37, p10]*<br>• These effects could be reached thanks to the specificities of the website, that is its […] multimediality (material was provided in written, audio and video form), usability (the website was easy to use […]) [28, p31]<br>• They [the users] were positive about the content, user-friendliness and web-based design. They |

| | | | | | |
|---|---|---|---|---|---|
| | | | | | said the information was easy to understand, to the point and conveniently arranged [36, p5]<br>• Finally, almost all OPs were positive about the user-friendliness and design of the program [36, p5] |
| | IT affinity | • Lack of affinity with computers<br>• *Lack of affinity with web-based programmes | • Some OPs had no affinity with the use of a web-based program in general and therefore preferred not to use this method [36, p5]<br>• A small number of employees […] had 'no affinity with computers' [36, p6] | • Enjoying working with a computer | • In addition, some informants stated that […] to enjoy working at the computer, facilitated patient participation in the rehabilitation [38, p6] |
| | Access and convenience | • Not able to choose starting time of DHI<br>• *No access to computer during consultation | • Although, some informants perceived restrained patient participation by the fact that they were not able to choose the starting time of the Web-BCPA course themselves (due to study protocol) […] [38, p5]<br>• Finally, some OPs faced practical | • Easily accessible with low effort<br>• Accessible at all hours and locations<br>• Accessible even during periods with severe pain symptoms<br>• Ability to take all the time needed | • Patient participation was emphasized by having access to the Web-BCPA on computer or tablet at all hours and locations [38, p5]<br>• The opportunities to work in the Web-BCPA at home were experienced to provide continuity in |

For peer review only

| | | | | | |
|---|---|---|---|---|---|
| | | | obstacles such as […] no access to a computer or the internet in their consulting rooms [36, p5] | | the rehabilitation [38, p5]<br>• These effects could be reached thanks to the specificities of the website, that is its usability ([…] accessible from home without the necessity of intermediaries) [28, p31]<br>• […] informants described that the Web-BCPA provided opportunities to rehabilitation during periods with severe symptoms without having to be present at the health care center [38, p6] |
| Quality and quantity of content | Quality of content | • Contradicting content between DHI and HCP | • For some employees the exercises suggested by the program conflicted with the exercises given by the physiotherapist [36, p5] | • Trustworthy content and source<br>• Easily understandable content<br>• High quality of content<br>• Steady content<br>• *Appropriate content | • *Knowing there is a serious website where there are contributions, it strengthens you a bit [28, p29]*<br>• Some users felt reassured because they had a trustworthy place where they could address concerns [27, p641] |

For peer review only

| | | | | | |
|---|---|---|---|---|---|
| | | | | | • These effects could be reached thanks to the specificities of the website, that is its […] trustworthiness (material was controlled by health professionals according to the criteria of Evidence Based Medicine) [28, p31]<br>• They [the users] were positive about the content, user-friendliness and web-based design. They said the information was easy to understand, to the point and conveniently arranged [36, p5]<br>• More than half of the OPs were positive about the content (e.g. information, exercises, instructions) [36, p5]<br>• […] the stability of the material helped them to construct their personal frame of reference about the nature and the |

For peer review only

| | | | | | |
|---|---|---|---|---|---|
| | | | | | course of their cLBP [27, p640] |
| | Amount of content | • Too much content to choose from<br>• Too much information to fully comprehend | • According to some people, Oneself provided too much information, risking creating confusion about the comprehension of the health problem and the identification of the best way to treat it: *There is a lot of information, probably almost too much, don't you think? [28, p29]*<br>• […] having difficulties to choose from its content, were experienced to restrain patient participation [38, p8] | • A lot of content to choose from | • The richness and trustworthiness of the information […] helped them to construct their personal frame of reference about the nature and the course of their cLBP [28, p28]<br>• First, the quality and continual update of the website encouraged people to visit Oneself again and to continue thinking about self-management [28, p29] |
| Tailoring and personalisation | Tailoring, specificity and personalisation | • Content not tailored to individual needs and/or pain severity<br>• Content perceived not new or relevant | • […] because some of the advice and exercises were not specific enough, they did not apply to the employee's situation [36, p5] | • Content accounting for individual needs and/or pain severity<br>• Self-identification in content<br>• Opportunity to influence treatment | • *..it was obvious that it (the rehabilitation) was about me, it wasn't about just anyone.. it was about my problems, my strengths and how I felt.. they (the HCPs* |

For peer review only

| | | | | | |
|---|---|---|---|---|---|
| | | | • Some persons perceived information not new nor relevant. In this case, the use of Oneself lead to feelings of hopelessness: two participants had the impression that again there was no solution for their problem [28, p29]<br>• *The exercises that you have on the website are good, but I can't do any of them, no. I tried to do them a bit on the bed, but with my arm that doesn't work, my knees that don't work... There are lots, indeed I had written down those that I could do, but then many times your will is missing (...) Then you get sick of it. I know, that it's for my own good that I should exercise, but after a while I... Then you don't have grand results, and so even* | | *started from a blank page, I was not fitted into an average template of how it ought to be.. it (the rehabilitation) started with my point of view [38, p4-5]*<br>• *I really liked the personalization. I thought it was a nice touch. Suggestions were more specific and tailored, which for me made them more relevant and likely for me to use them [37, p9]*<br>• *Previously I had read about CBT (Cognitive Behavioral Therapy), but I had never thought of it as a help for my condition.. I want to compare this rehabilitation with a smorgasbord from which is it easy to taste [38, p5]*<br>• *It gives you descriptions and you say: this stuff here.. I see it, I see it! I recognise myself in it,* |

For peer review only

| | | | | | |
|---|---|---|---|---|---|
| | | | *for my back sometimes I go through periods, moments where I'm, let's say, very diligent, and then sometimes… (…) Yes, it's interesting. But there are always the same things that you then don't do [28, p29]* | | *I recognise myself here [27, p640]*<br>• Informants experienced that being able to identify themselves with the content in the rehabilitation and finding it trustworthy were important to patient participation and being confirmed [38, p5]<br>• They [informants] described that they were confirmed when they could identify their illness experience and life situation, as well as their own thoughts and cognitions about their pain condition, in the texts and the assignments of the Web-BCPA [38, p7] |
| Motivation and support | Personal attributes and resources | • Adhering to biomedical model of LBP<br>• Seeing LBP as a marginal problem<br>• Preferring other treatment regimens, e.g. with human contact | • *I went to a doctor who told me 'there is nothing to do, just resign yourself to it'. So this unleashed really the research to find something. But after eight years I didn't find the magic* | • High level of awareness and self-management of LBP<br>• Aware that LBP would not be fixed with a medical solution and ready to accept active role | • In addition, some informants stated that their work experience, such as having a solution-focused work […] facilitated patient participation in the rehabilitation [38, p6] |

For peer review only

| | | | | | |
|---|---|---|---|---|---|
| | | • Lack of knowledge about LBP and treatments<br>• Physical health (e.g. pain, fatigue)<br>• Psychological symptoms | *cure, unfortunately. And one continuously hears 'they are doing new research!' But hopefully they will arrive in time in order to do something. (…) I'm always in search of the super novelty, the one that heals [28, p30]*<br>• One employee mentioned that the back or neck pain they were suffering from may have prevented them from sitting at a computer [36, p6]<br>• Pain, fatigue and other psychological symptoms were perceived to limit patient participation [38, p6]<br>• Three users could be defined as passive self-managers: They adhered to a traditional biomedical model of cLBP and were convinced that the solution of their problem had to be | • Emotional and cognitive resources, e.g. motivation, interest, commitment and self-confidence in self-management of LBP<br>• Enjoy solution focused work | • *I already know which road I have to follow in detail. I need details or confirmation on these details [28, p29]*<br>• They described emotions and cognitions that affected patient participation. Having motivation, interest, commitment, and self-confidence were perceived to favor patient participation [38, p6]<br>• Most of the users could be defined as experienced self-managers, in the sense that they had a rather high level of awareness and self-management of cLBP even before knowing Oneself. These people […] had a rather clear idea about their diagnosis, and knew that they had to play an active role in dealing with their health problem [27, p635] |

For peer review only

| | | | | | |
|---|---|---|---|---|---|
| | | | found by health professionals. These people went to Oneself to find a definitive medical solution for their cLBP [27, p635]<br>• Three users could be defined as latent self-managers. […] For all of them, cLBP was at the moment a marginal problem, in the sense that it was intermittent and light. These users did not really need to engage in a long-term process of self-management: When pain appeared, they usually dealt with it through some easy coping strategies, such as taking painkillers, going to the chiropractic, etc [27, p636] | | • Two users could be defined as novices in terms of self-management. These participants were aware that a medical solution to cLBP did not exist and were ready to accept that they had to become actively involved in their cLBP care. However, they did not know how to do it [27, p635] |
| | Support to use DHI | • HCP unsupportive of use of DHI<br>• No support from authorities | • *I planned to complete the program (the Web-BCPA).. I am not sure how much I had left.. probably the last module.. but I was denied sick-leave* | • HCP supportive of use of DHI<br>• Support from family<br>• Support from authorities<br>• Support from other suffers (e.g. successful testimonials) | • *It's nice knowing that there is someone else [28, p29]*<br>• *When you are going through a moment when you have backache and you* |

| | | | | | |
|---|---|---|---|---|---|
| | | | *compensation by the Social Insurance Agency and had to put in a lot of energy to explain my situation and meet with the psychosocial counsellor.. I did not have the strength to do anything else.. I have used so much energy to fight for my cause [12052, 6]*<br>• One employee said, *I expected more commitment from my OP.* This did not encourage employees to use the program [2120, 5] | | *read a testimony which says 'yes, there is someone who was able to do it', it gives you hope [28, p29]*<br>• Support, trust and respect from a family member, employer, the Swedish Social Insurance Agency (SSIA) or the Employment Service were experienced to facilitate patient participation in the rehabilitation [38, p6] |
| | Features of DHI | • DHI not guiding or supporting participants enough (e.g. to plan for execution of physical activity recommendation from DHI) | • *I received the suggestion to ride a bike, but that's currently simply not possible, logistically [37, p10]*<br>• *If it could ask me to rank the things I enjoy doing and then download weather data for the following days. This could suggest times when I have performed these tasks in the* | • Interaction/interactivity<br>• Information about self-management of LBP<br>• Goal-setting<br>• Action-planning<br>• Follow-up and evaluation<br>• Adjusting treatment related to setbacks and progress<br>• Monitoring own progress in graphs<br>• Variation of content<br>• Update of content | • To acquire knowledge and insights were thought of as patient participation, and included self-reflection, self-identification, and feedback [38, p5]<br>• […] with opportunities to influence and a variety of treatments to choose according to one's own needs and priorities [38, p5] |

For peer review only

| | | | | | |
|---|---|---|---|---|---|
| | | | *past and also match it with weather predictions. "You played tennis for last Tuesday in the afternoon for 90 minutes. How about from 2 to 4 today when the weather will be clear and 85".* *[37, p10]* | | • To adjust a goal or treatment planning in relation to progress or setback was described as patient participation: *I feel it is important to set goals and to follow-up those goals.. and to why a goal is reached and why another is not.. this made me aware of that I needed other tools (in the rehabilitation)* [38, p6]<br>• Patient participation was reported when informants monitored results shown by the interactive graphs in the Web-BCPA: *.. days when I had a lot of pain I used to remain sedentary, and as soon as I had a better day I was eager to do all kinds of activities that day.. before I started with the assignment activity planning (in the Web-BCPA) I was* |

For peer review only

| | | | | | |
|---|---|---|---|---|---|
| | | | | | *not aware of how my behaviour related to the days with pain, but by monitoring this over time I started to plan my daily activities in a more balanced way [38, p6]*<br>• These effects could be reached thanks to the specificities of the website, that is its interactivity (people could ask specific questions to health professionals who were available daily for responding), […] dynamism (the website was updated weekly) […] [28, p31]<br>• The informants' experienced patient participation when they analyzed their situation taken into account their resources and restrictions, set goals for behavior change, and planned treatments and activities. Also, patient participation was stated when |

For peer review only

| | | | | | |
|---|---|---|---|---|---|
| | | | | | treatments, self-care, and planning were followed-up and evaluated [38, p6] |
| | HCP factors for support of patients | • *Time restrictions of consultations<br>• *Difficulty keeping DHI in mind during consultations<br>• *Difficulty providing patients with accurate information about DHI<br>• *Perceiving no benefit of DHI compared to usual treatment<br>• *Preferring other treatment regimens, e.g. with human contact | • *It takes time to get used to the recruitment process and to using the program [36, p5]*<br>• A second important barrier for OPs was the limited time available for introducing employees to the program and working with it as well. […] *We lack the time to do this kind of projects [36, p5]*<br>• One OP stated that he did not use the program because he did not believe in 'computer-based treatment' of physical pain. He explained, *The ability to touch people is an essential element in the treatment of people with back or neck pain. [36, p5]* | • *DHI a good medium for counselling employees | • About half of the OPs indicated that a website is a good medium for counselling of employees with back or neck pain [36, p5] |

For peer review only

| | | | | | |
|---|---|---|---|---|---|
| | | | • One OP stated that he was quite capable of managing the RTW process himself and did not need a program for additional support. Many preferred the more familiar therapies (e.g. physiotherapy) […]. They preferred having personal contact with employees [36, p5] | | |

*= HCP perspective; **IT**: information technology; **HCP**: healthcare professional; **DHI**: Digital health intervention

For peer review only

# PRISMA 2009 Checklist

| Section/topic | # | Checklist item | Reported on page # |
|---|---|---|---|
| **TITLE** | | | |
| Title | 1 | Identify the report as a systematic review, meta-analysis, or both. | #1 lines 1-3 |
| **ABSTRACT** | | | |
| Structured summary | 2 | Provide a structured summary including, as applicable: background; objectives; data sources; study eligibility criteria, participants, and interventions; study appraisal and synthesis methods; results; limitations; conclusions and implications of key findings; systematic review registration number. | #2 lines 35-74 |
| **INTRODUCTION** | | | |
| Rationale | 3 | Describe the rationale for the review in the context of what is already known. | 4-6 |
| Objectives | 4 | Provide an explicit statement of questions being addressed with reference to participants, interventions, comparisons, outcomes, and study design (PICOS). | #5 lines 136-139 |
| **METHODS** | | | |
| Protocol and registration | 5 | Indicate if a review protocol exists, if and where it can be accessed (e.g., Web address), and, if available, provide registration information including registration number. | #6 line 146 |
| Eligibility criteria | 6 | Specify study characteristics (e.g., PICOS, length of follow-up) and report characteristics (e.g., years considered, language, publication status) used as criteria for eligibility, giving rationale. | #7 (Table 1) |
| Information sources | 7 | Describe all information sources (e.g., databases with dates of coverage, contact with study authors to identify additional studies) in the search and date last searched. | #8 |
| Search | 8 | Present full electronic search strategy for at least one database, including any limits used, such that it could be repeated. | #8 & Suppl. file 1 |
| Study selection | 9 | State the process for selecting studies (i.e., screening, eligibility, included in systematic review, and, if applicable, included in the meta-analysis). | #8 |
| Data collection process | 10 | Describe method of data extraction from reports (e.g., piloted forms, independently, in duplicate) and any processes for obtaining and confirming data from investigators. | #9 |
| Data items | 11 | List and define all variables for which data were sought (e.g., PICOS, funding sources) and any assumptions and simplifications made. | #8 |
| Risk of bias in individual studies | 12 | Describe methods used for assessing risk of bias of individual studies (including specification of whether this was done at the study or outcome level), and how this information is to be used in any data synthesis. | #9 |
| Summary measures | 13 | State the principal summary measures (e.g., risk ratio, difference in means). | #9-10 |

For peer review only

# PRISMA 2009 Checklist

| | | | |
|---|---|---|---|
| Synthesis of results | 14 | Describe the methods of handling data and combining results of studies, if done, including measures of consistency (e.g., $I^2$) for each meta-analysis. | #9-10 |



| Section/topic | # | Checklist item | Reported on page # |
|---|---|---|---|
| Risk of bias across studies | 15 | Specify any assessment of risk of bias that may affect the cumulative evidence (e.g., publication bias, selective reporting within studies). | #31 |
| Additional analyses | 16 | Describe methods of additional analyses (e.g., sensitivity or subgroup analyses, meta-regression), if done, indicating which were pre-specified. | N/A |
| **RESULTS** | | | |
| Study selection | 17 | Give numbers of studies screened, assessed for eligibility, and included in the review, with reasons for exclusions at each stage, ideally with a flow diagram. | #11 |
| Study characteristics | 18 | For each study, present characteristics for which data were extracted (e.g., study size, PICOS, follow-up period) and provide the citations. | #12-15 |
| Risk of bias within studies | 19 | Present data on risk of bias of each study and, if available, any outcome level assessment (see item 12). | #18 & Suppl. File 2 |
| Results of individual studies | 20 | For all outcomes considered (benefits or harms), present, for each study: (a) simple summary data for each intervention group (b) effect estimates and confidence intervals, ideally with a forest plot. | #18-26 (No quantitative assessment) |
| Synthesis of results | 21 | Present results of each meta-analysis done, including confidence intervals and measures of consistency. | #18-26 (No quantitative assessment) |
| Risk of bias across studies | 22 | Present results of any assessment of risk of bias across studies (see Item 15). | N/A (No quantitative assessment) |
| Additional analysis | 23 | Give results of additional analyses, if done (e.g., sensitivity or subgroup analyses, meta-regression [see Item 16]). | N/A |
| **DISCUSSION** | | | |
| Summary of evidence | 24 | Summarize the main findings including the strength of evidence for each main outcome; consider their relevance to key groups (e.g., healthcare providers, users, and policy makers). | #26-30 |
| Limitations | 25 | Discuss limitations at study and outcome level (e.g., risk of bias), and at review-level (e.g., incomplete retrieval of identified research, reporting bias). | #30-31 |
| Conclusions | 26 | Provide a general interpretation of the results in the context of other evidence, and implications for future | #32 |

For peer review only

For peer review only - http://bmjopen.bmj.com/site/about/guidelines.xhtml

# PRISMA 2009 Checklist

| | | | |
|---|---|---|---|
| | | research. | |
| **FUNDING** | | | |
| Funding | 27 | Describe sources of funding for the systematic review and other support (e.g., supply of data); role of funders for the systematic review. | #33 line 610-611 |

*From:* Moher D, Liberati A, Tetzlaff J, Altman DG, The PRISMA Group (2009). Preferred Reporting Items for Systematic Reviews and Meta-Analyses: The PRISMA Statement. PLoS Med 6(7): e1000097. doi:10.1371/journal.pmed1000097

For more information, visit: www.prisma-statement.org.



For peer review only

For peer review only - http://bmjopen.bmj.com/site/about/guidelines.xhtml

BMJ Open

# Barriers and facilitators to patient uptake and utilisation of digital interventions for the self-management of low back pain: a systematic review of qualitative studies

| | |
|---|---|
| Journal: | *BMJ Open* |
| Manuscript ID | bmjopen-2020-038800.R2 |
| Article Type: | Original research |
| Date Submitted by the Author: | 16-Nov-2020 |
| Complete List of Authors: | Svendsen, Malene; National Research Centre for the Working Environment, Musculoskeletal disorders and physical work demands; University of Southern Denmark Department of Sports Science and Clinical Biomechanics, Physical activity and health in work life<br>Wood, Karen; University of Glasgow<br>Kyle, John ; University of Glasgow, Institute of Health and Wellbeing<br>Cooper, K; Robert Gordon University, School of Health Sciences<br>Rasmussen, Charlotte Diana Nørregaard; The National Research Centre for The Working Environment,<br>Sandal, Louise; University of Southern Denmark, Department of Sport Science and Clinical Biomechanics<br>Stochkendahl, Mette; Nordic Institute of Chiropractic and Clinical Biomechanics, ; University of Southern Denmark, Institute of Sports Science and Clinical Biomechanics<br>Mair, Frances; University of Glasgow, General Practice and Primary Care<br>Nicholl, Barbara; University of Glasgow, General Practice and Primary Care |
| <b>Primary Subject Heading</b>: | Public health |
| Secondary Subject Heading: | Qualitative research |
| Keywords: | Back pain < ORTHOPAEDIC & TRAUMA SURGERY, Health informatics < BIOTECHNOLOGY & BIOINFORMATICS, PAIN MANAGEMENT, QUALITATIVE RESEARCH |
| | |

SCHOLARONE™
Manuscripts

*I, the Submitting Author has the right to grant and does grant on behalf of all authors of the Work (as defined in the below author licence), an exclusive licence and/or a non-exclusive licence for contributions from authors who are: i) UK Crown employees; ii) where BMJ has agreed a CC-BY licence shall apply, and/or iii) in accordance with the terms applicable for US Federal Government officers or employees acting as part of their official duties; on a worldwide, perpetual, irrevocable, royalty-free basis to BMJ Publishing Group Ltd ("BMJ") its licensees and where the relevant Journal is co-owned by BMJ to the co-owners of the Journal, to publish the Work in this journal and any other BMJ products and to exploit all rights, as set out in our licence.*

*The Submitting Author accepts and understands that any supply made under these terms is made by BMJ to the Submitting Author unless you are acting as an employee on behalf of your employer or a postgraduate student of an affiliated institution which is paying any applicable article publishing charge ("APC") for Open Access articles. Where the Submitting Author wishes to make the Work available on an Open Access basis (and intends to pay the relevant APC), the terms of reuse of such Open Access shall be governed by a Creative Commons licence – details of these licences and which Creative Commons licence will apply to this Work are set out in our licence referred to above.*

*Other than as permitted in any relevant BMJ Author's Self Archiving Policies, I confirm this Work has not been accepted for publication elsewhere, is not being considered for publication elsewhere and does not duplicate material already published. I confirm all authors consent to publication of this Work and authorise the granting of this licence.*

For peer review only

# Barriers and facilitators to patient uptake and utilisation of digital interventions for the self-management of low back pain: a systematic review of qualitative studies

Malene Jagd Svendsen[2,4], Karen Wood[1], John Kyle[1], Kay Cooper[3], Charlotte Diana Nørregaard Rasmussen[2], Louise Fleng Sandal[4], Mette Jensen Stochkendahl[4,5], Frances S Mair[1], Barbara I Nicholl[1]

**Affiliations**

1. Institute of Health & Wellbeing, University of Glasgow, Glasgow, UK

2. National Research Centre for the Working Environment, Copenhagen Ø, Denmark

3. School of Health Sciences, Robert Gordon University, Aberdeen, UK

4. Department of Sports Science and Clinical Biomechanics, University of Southern Denmark, Odense, Denmark

5. Nordic Institute of Chiropractic and Clinical Biomechanics, Odense, Denmark

**Email Addresses**

Malene Jagd Svendsen mas@nrcwe.dk

Karen Wood Karen.Wood@glasgow.ac.uk

John Kyle John.Kyle@nhs.net

Kay Cooper k.cooper@rgu.ac.uk

Charlotte Diana Nørregaard Rasmussen cnr@nrcwe.dk

Louise Fleng Sandal lsandal@health.sdu.dk

Mette Jensen Stochkendahl m.jensen@nikkb.dk

Frances S Mair Frances.Mair@glasgow.ac.uk

Barbara Nicholl Barbara.Nicholl@glasgow.ac.uk

For peer review only

**Corresponding Author:**

Malene Jagd Svendsen

National Research Centre for the Working Environment, Lersø Parkallé 105, 2100 Copenhagen, Denmark

Tel: +45 39165329 Email: mas@nrcwe.dk

Word count: 6232 words

## Abstract

**Objectives:** Low back pain (LBP) is a leading contributor to disability globally. Self-management is a core component of LBP management. We aimed to synthesise published qualitative literature concerning digital health interventions (DHIs) to support LBP self-management to: 1) determine engagement strategies, 2) identify barriers and facilitators affecting patient uptake/utilisation, 3) develop a preliminary conceptual model of barriers and facilitators to uptake/utilisation.

Design: Systematic review following PRISMA guidelines.

Data sources: MEDLINE, Embase, CINAHL, PsycINFO, Cochrane Library, DoPHER, TROPHI, Web of Science and OT Seeker, from January 2000 – December 2018, using the concepts: LBP, DHI, self-management.

Eligibility criteria: Peer-reviewed qualitative study (or component) examining engagement with, or barriers and/or facilitators to the uptake/utilisation of an interactive DHI for self-management of LBP in adults (community, primary or secondary care settings).

Data extraction and synthesis: Standardised data extraction form was completed. COREQ checklist was used to assess methodology. Data was synthesised narratively for engagement strategies, thematically for barriers/facilitators to uptake/utilisation, and normalisation process theory was applied to produce a conceptual model.



For peer review only - http://bmjopen.bmj.com/site/about/guidelines.xhtml

**Results:** We identified 14191 citations, of which 105 full-text articles were screened, and five full text articles from four studies included. These were from community and primary care contexts in Europe and the US, and involved 56 adults with LBP and 19 healthcare professionals. There was a lack of consideration on how to sustain engagement with DHIs. Examination of barriers and facilitators for uptake/utilisation identified four major themes: IT usability-accessibility; quality-quantity of content; tailoring-personalisation; motivation-support. These themes informed the development of a preliminary conceptual model for uptake/utilisation of a DHI for LBP self-management.

**Conclusions**: We highlight key barriers and facilitators that should be considered when designing DHIs for LBP self-management. Our findings are in keeping with reviews of DHIs for other long-term conditions, implying these findings may not be condition specific.

**PROSPERO Registration number** CRD42016051182

**Systematic review registration**: A protocol for this systematic review was registered with https://www.crd.york.ac.uk/PROSPERO/ (CRD42016051182) on November 10th, 2016. https://www.crd.york.ac.uk/PROSPERO/display_record.php?ID=CRD42016051182

**Article summary**

Strengths and limitations of this study:

- This systematic review of qualitative studies explored barriers and facilitators for the uptake and utilisation of digital health interventions for low back pain (LBP) to inform the future design and implementation processes of such interventions.
- Searches in multiple databases and independent data extraction, quality appraisal and detailed data analysis are strengths of our review. However, our search strategy revealed that literature in

For peer review only



For peer review only - http://bmjopen.bmj.com/site/about/guidelines.xhtml

the field of digital self-management for LBP is sparse as only a small number of eligible studies were identified.

- Given the limited literature, it is possible that not all important barriers and facilitators for uptake and utilisation have been identified and thus our conceptual model must be considered preliminary.

**Keywords:** Low back pain; eHealth; self-management; qualitative, engagement; utilisation; NPT

## Background

Low back pain (LBP) affects approximately 12% of the general population at any point in time (1); it is the leading contributor to disability worldwide (2) and is associated with significant personal (3) and societal costs (4, 5). Self-management approaches are consistently recommended in clinical guidelines as a core component of LBP management (6, 7); however, adherence to self-management strategies has proved challenging, especially without support and reinforcement (8, 9). Digital health interventions (DHIs), health interventions accessed through a computer, mobile phone, or other handheld device, involving a web-based programme, desktop programme or application; offer a potential method of supporting self-management (10-12), and particularly the possibility of tailoring self-management advice, may hold significant potential for people with LBP (13). DHIs or “digital therapeutics” are becoming increasingly popular and, as technological innovations increase, it is expected that this trend will continue (14, 15). Until now, two systematic reviews have examined the use of DHIs to support the self-management of LBP. The first, by Garg et al., aimed to determine which web-based interventions are of benefit to patients (16). They identified nine randomised controlled trials (RCTs), including a total of 1796 participants. Four trials studied online cognitive behavioural therapy (CBT) with the remaining five trials studying web-based interventions with interactive features such as a virtual gym, testimonials, or moderated discussion groups. Garg et al. reported that online CBT approaches appeared to reduce catastrophizing and improve patient attitudes,



For peer review only - http://bmjopen.bmj.com/site/about/guidelines.xhtml

For peer review only

whilst studies of web-based interventions with interactive features used a variety of diverse outcome measures yielding inconclusive results; thus, making it difficult to draw firm conclusions regarding long-term impact for people with LBP.

The second review, by Nicholl et al., aimed to appraise the evidence concerning the use of interactive DHIs to support patient self-management of LBP with a focus on the outcome measures used and reported effects (17). They identified six completed RCTs studying digital tools for the self-management of LBP including a total of 2706 participants. Nicholl et al. reported that only one of the six completed RCTs observed a between-group difference in favour of the digital intervention, with none of the studies demonstrating any evidence of harm. The authors noted that there was considerable variation in the nature and delivery of the interventions and inconsistency in the choice of outcomes and concluded that the current evidence base for DHIs to support the self-management of LBP remained weak.

Yet, hundreds of smartphone applications (apps) related to LBP are currently available on the app market, most developed with very little scientific rigour (18). In order to facilitate the development of appropriate and effective self-management DHIs for those with LBP, it is important to have an understanding of the factors that help or hinder user engagement and adherence. Across different conditions, multiple barriers and facilitators to engaging with DHIs have previously been identified, including issues such as motivation and support, digital literacy, privacy, usability, quality and tailoring (17, 19). However, given the diverse range of DHIs available, it can be difficult to apply these findings to a specific patient population or piece of technology. Understanding the experience of users of DHIs designed specifically to assist self-management of LBP would help determine how to optimise DHIs for this group of users.

The purpose of this systematic review was therefore to synthesise and critically appraise the published qualitative literature concerning the use of DHIs to promote self-management of LBP in order to address the following two research questions:

1. What engagement strategies at the time of enrolment have been utilised in DHIs aimed at supporting patient self-management of LBP?



For peer review only - http://bmjopen.bmj.com/site/about/guidelines.xhtml

2. What are the barriers and facilitators to patient uptake and utilisation of digital interventions to support self-management of LBP?

The final objective of the systematic review was to develop a preliminary conceptual model of barriers and facilitators to uptake and utilisation of digital interventions to support self-management of LBP.

**Methods**

Protocol and registration

This review was registered in the International Prospective Register of Systematic Reviews, PROSPERO, registration no. CRD42016051182 (20) and reporting is consistent with the Preferred Reporting Items for Systematic Reviews and Meta-Analyses (PRISMA) statement (21).

Patient and public involvement

This research was done without patient involvement. Patients were not invited to comment on the study design and were not consulted to develop patient relevant outcomes or interpret the results. Patients were not invited to contribute to the writing or editing of this document for readability or accuracy.

Eligibility criteria

Qualitative studies that examine engagement, barriers and/or facilitators to patient uptake and utilisation of digital interventions for the self-management of LBP were included; inclusion and exclusion criteria are outlined in Table 1.

**Table 1:** Inclusion and exclusion criteria.

| **Inclusion criteria** | |
|---|---|
| Study type | • Published in peer-reviewed journals between January 1st 2000 and December 18th 2018.<br>• Original qualitative studies, studies involving secondary qualitative analysis of qualitative data and qualitative studies that were part of a mixed methods study (provided the qualitative methodology was described). |



For peer review only - http://bmjopen.bmj.com/site/about/guidelines.xhtml

| | |
|---|---|
| | • Qualitative data collected via questionnaires or other methods not involving direct contact or observation of participants were eligible for inclusion provided questions were answered using free text and analysed using a qualitative approach.<br>• Qualitative data describing barriers and/or facilitators to the uptake or utilisation of digital interventions or containing a description of an engagement strategy (i.e. any method used to get people to enrol into the study) from a patient or HCP's perspective. |
| Language | • Published in English, Danish or Norwegian. |
| Participants | • Adults >18 years with LBP or HCPs providing care for such patients. |
| Setting | • Community, primary or secondary care and other specialist contexts including those that recruit via media. |
| Digital intervention | • Any intervention accessed through a computer, mobile phone, or other handheld device, involving a web-based programme, desktop programme or application that provided self-management content (consistent with previous reviews (17, 22)).<br>• Interventions must involve an element of interaction between the user and the digital interface; this was defined as information being taken from users which then provided some form of automated feedback and/or advice in response.<br>• Interventions that included face-to-face contact were only included if this interaction was in addition to an automated, interactive digital component without direct HCP mediation. |
| **Exclusion criteria** | |
| Study type | • Descriptive case studies, lexical studies that analyse natural language data presented as qualitative results, literature or systematic reviews, meta-analyses, studies without a sampling procedure (i.e. no clear description of recruitment strategy) and commentary articles written to convey opinion or stimulate discussion with no research component. |

**HCP**: Healthcare professional

Information sources and search strategy

A systematic search of bibliographic databases (MEDLINE, Embase, CINAHL, PsycINFO, Cochrane Library, DoPHER, TROPHI, Web of Science and OT Seeker) was conducted after the search strategy had been developed in collaboration with a librarian at the Norwegian University of Science and Technology (NTNU) and experienced researchers in the field of LBP and digital health interventions. The search strategy has previously been described and published by Nicholl et al. (17). Reference and citation tracking was utilised to identify relevant references. All databases were searched for publications using three groups of concepts: (1) low back pain, (2) digital intervention, and (3) self-management. The search was conducted in

For peer review only

three waves using the same search strategy: the first for publications added between January 2000 and March 2016, then a subsequent updated search for articles added between March 2016 and October 2016, and lastly, articles added between October 2016 and December 2018. Limitation of year of publication from 2000 onwards was chosen as our review was aimed at understanding current experiences of digital health technologies, justified by emerging Internet access around the millennium and the developing field of DHIs that followed, and further supported by other systematic reviews of digital interventions (16, 23, 24). The complete search strategy, including specifications on the use of title, keywords or abstract screening is documented in Supplementary File 1.

Study selection

All identified citations were uploaded to Distiller SR software (Evidence Partners, Ottawa, Canada) and duplicates were removed. Title and abstract screening were performed by two of four independent reviewers (JK, MaS, KC, KW) using Distiller SR. Any disagreement between the two reviewers at title screening level resulted in inclusion of the citation to abstract level and subsequently any disagreement at abstract level resulted in inclusion of the citation to the full-text screening level. Full-text screening was also performed by two of four independent reviewers (JK, MaS, KC, KW) with any discrepancies at this level being resolved through discussion mediated by a third party (BN, CR, MeS, KC).

Data extraction

A comprehensive, standardised data extraction template designed specifically for this review in Distiller SR was utilised by two of four independent researchers (JK, MaS, BN, KW). Where available, information collected included the study title, authors, citation, year of study and publication, country, inclusion/exclusion criteria, aim, setting, characteristics of the digital intervention, recruitment methods, method of qualitative data collection and analysis, participant numbers and characteristics, any



For peer review only - http://bmjopen.bmj.com/site/about/guidelines.xhtml

engagement strategies, barriers or facilitators identified either by the authors or in participant quotes, conclusions, limitations, funding sources and any potential conflicts of interest declared.

Quality appraisal

The complete 32-item Consolidated Criteria for Reporting Qualitative Research (COREQ) checklist (25, 26) was used to assess the methodological quality of the articles progressing to data extraction. Two of three reviewers (BN, KC, KW) independently identified whether each of the 32-items were reported or not, and descriptive information was provided where possible. Disagreements between reviewers were resolved through discussion. A-priori cut-off points were not determined as studies were not excluded on the basis of methodological quality due a lack of clear agreement on how best to apprise qualitative literature (27). Two of the included articles report on the qualitative evaluation of the same intervention but were treated as separate articles for quality appraisal (28, 29).

Data synthesis and analysis

Information on the engagement strategies, defined as methods used to recruit and initially motivate participants to enrol in the DHI study, in each study was described narratively as this was only provided descriptively in the included studies. Our data synthesis of barriers and facilitators to patient uptake and utilisation of the DHI for LBP involved a thematic approach (30). Data on barriers and facilitators were extracted from results and discussion sections of the included studies. Each item of extracted data was initially coded by one reviewer (MaS). When new codes appeared during the analysis of a particular article, the articles that had previously been examined were re-read and re-coded if appropriate. This continuous adjustment was carried out in cooperation with a second reviewer (KW). Emergence and mapping of codes were discussed at coding clinics to ensure construction of themes that were internally homogenous and externally heterogeneous (i.e. no data excluded due to lack of a suitable theme, and no data falling

For peer review only

202 between two themes or fitting into more than one theme) (31, 32) (MaS, KW, FM, BN). This resulted in a coding taxonomy for mapping identified codes as barriers or facilitators for each theme.

204 A preliminary conceptual model of barriers and facilitators to uptake and utilisation of DHIs to support self-management of LBP was developed by mapping the identified themes to the four constructs of Normalization Process Theory (NPT). NPT is a sociological theory developed to explore the process of implementing a new complex intervention, in this case it can help explain how people individually and collectively embed DHIs into everyday practice (33, 34). The identified themes were mapped to NPT constructs by four reviewers (KW, FM, BN, JK) using the coding framework presented in Table 2. This approach has been successfully applied in other systematic reviews of DHIs for chronic disease self-management issues (19, 35, 36) and provides a solid conceptual basis from which to understand barriers and facilitators to patient and HCP uptake and utilisation of DHIs. Any themes that could not be coded to the NPT constructs were carefully noted to ensure that themes outside the scope of NPT would still be captured to assure appropriateness of the model.

**Table 2:** Core constructs of Normalization Process Theory (NPT) (33, 34) and related coding framework for development of preliminary conceptual model of barriers and facilitators to uptake and utilisations of digital interventions to support self-management of LBP.

| Core constructs of NPT | Coding framework |
|---|---|
| Coherence (Sense Making Work; enrolling with the DHI): development of an individual and collective understanding of the new intervention when faced with operationalizing it. | • How people understand and view the benefits versus disbenefits of DHIs and decide whether it is appropriate for them to use.<br>• Motivation and willingness to commit to self-management activities. |
| Cognitive Participation (Engagement Work; engaging with the DHI): relational work to build and sustain engagement with a new intervention. | • Willingness to "buy into" the DHI and whether it is a legitimate means to promote self-management of LBP.<br>• Issues relating to the support provided to use the DHI and level of engagement of HCPs involved with the DHI. |
| Collective Action (Operationalisation Work; utilising the DHI): investment of effort and resources to enact the new intervention. | • Ease of use, accessibility and appropriateness of the DHI.<br>• Resources, training, workload and technical support.<br>• Perceived quality and trustworthiness of DHI content and function. |
| Reflexive Monitoring (Appraisal Work; maintaining engagement with DHI): evaluation of the impact of the new intervention on individuals and groups | • How people judge the new DHI and the self-monitoring work that accompanied uptake of the DHI.<br>• Ability to tailor to an individual's needs. |



For peer review only - http://bmjopen.bmj.com/site/about/guidelines.xhtml

| | |
|---|---|
| along with any reconfigurations suggested. | |
| **Codes falling outside the NPT framework** | |
| | • Inherent personal attributes such as personal physical or cognitive abilities that could promote or inhibit DHI use. |

**DHI**: Digital health intervention; **HCP**: Healthcare professional

**Results**

Study selection

Of 14191 citations identified, 5973 were excluded as duplicates; 8113 were excluded following title and abstract screening (7436 at title level and 677 at abstract level) and a further 100 citations were excluded after full text screening. Overall, five full text articles were included in the review (Figure 1). These articles described four separate studies and included a total of 75 participants. The two articles (28, 29) reporting on the same study (Oneself) consisted of a qualitative evaluation of a website (29) and a mixed-method reporting of the same qualitative data combined with quantitative (pre- and post-use surveys and log files) data (28). As these two studies included the same qualitative data and user quotes, they were combined for analysis purposes.

**Figure 1** PRISMA flow diagram illustrating the screening process (Adapted from Moher et al (21)).

Study characteristics

The Get Well Fast (37) and Oneself studies (28, 29) were undertaken between 2006 and 2008 in the Netherlands and Switzerland, respectively. The MyBehaviorCBP study was conducted in the US between 2012 and 2014 (38), whilst the study period for the Swedish Web-BCPA study was not reported (39). The characteristics of the study participants are summarised in Table 3. No information was reported on comorbidities or ethnicity and only limited information on participant socioeconomic status was included.



For peer review only - http://bmjopen.bmj.com/site/about/guidelines.xhtml

238 **Table 3:** Participant characteristics of included studies

| Study; Country | Year of study | Number of participants in qualitative study | Age range | Sex (%) | SES |
|---|---|---|---|---|---|
| Oneself (28, 29)<br>Switzerland | 2006-2008 | N = 18 | 28-72 years<br><br><29 yrs: n = 1<br>30-39 yrs: n = 3<br>40-49 yrs: n = 5<br>50-59 yrs: n = 6<br>>60 yrs: n = 3 | 50% female | *Education*:<br>Secondary school: n = 2; High school or equivalent: n = 11; University degree: n = 5 |
| Get Well Fast (37)<br>Netherlands | 2008 | N = 28<br><br>OP+ = 11<br>OP- = 8<br>Employee: 9 | 40-50 years | OP: N/R<br><br>Employee: 33% female | White and blue-collar workers. Various levels of education |
| MyBehaviorCBP (38)<br>USA | 2012-2014 | N = 10 | 31-60 years | 70% female | N/R |
| Web-BCPA (39)<br>Sweden | N/R | N = 19 | 27-60 years | 79 % female | *Education:*<br>Elementary school: n = 2;<br>Secondary school: n = 12;<br>University degree: n = 5)<br><br>*Employment:*<br>Permanent employment: n = 12;<br>Temporary employment: n = 3;<br>Unemployed: n = 3; Social benefits: n = 1 |

**N:** Number; **OP+**: occupational physicians who recruited patients into DHI; **OP-:** occupational physicians who did not recruit patients into DHI; **N/R:** not reported; **SES:** socioeconomic status

239

240 DHI delivery mode varied between studies. In the Oneself, Get Well Fast and Web-BCPA studies, the DHI
241 consisted of information available on websites to which participants had either open access (28, 29) or had
242 personal log-ins (37, 39). The content of the MyBehaviorCBP intervention was delivered to participants via
243 a mobile phone app (38). Two of the studies tailored the content of their DHI to the individual participant
244 by collecting information about the users and providing content that matched their needs (37, 38); in the



For peer review only - http://bmjopen.bmj.com/site/about/guidelines.xhtml

Get Well Fast study, content was tailored based on patient reports on pain, limitations, treatment, counselling, reintegration to work, work situation and work characteristics, relations at work, personality and daily activities (37), while the MyBehaviourCBP intervention collected sensory data from the users' smartphone (accelerometer signals and geolocation) and patient self-reported physical activity logs (38). Three interventions offered time limited programs of either five (37, 38) or eight weeks (39), while the fourth intervention was an open-to-access website with no time restrictions (28, 29) (Table 4).

**Table 4:** Participant inclusion criteria, sampling procedure for qualitative component and characteristics of digital intervention in included studies

| Study | Inclusion criteria for digital health intervention | Inclusion criteria and sampling procedures for qualitative study | Characteristics of digital health intervention |
|---|---|---|---|
| Oneself (28, 29) | • Anyone could register and use the Oneself website. | • Registered users of Oneself for at least 6 months.<br>• Visited the website at least 3 times.<br>• Suffering from chronic LBP (duration not defined).<br>• Living in the Italian part of Switzerland.<br>• Purposive and convenience sampling<br>• Invitation to participate in interview sent via email to eligible users.<br>• Reminder email sent after 2 weeks to anyone who had not responded.<br>• 238 users invited to participate, 18 agreed. | Open access website containing:<br>• Library – textual educational information on back pain.<br>• Radio – 10x2-minute recorded audio messages on relevant topics.<br>• Gym - videos demonstrating stretching, stabilization and mobilization exercises accompanied by photographs and written descriptions.<br>• Forum – users could interact with other users and HCPs, monitored by a content manager.<br>• Chat room – users could interact with other users and HCPs. Once a week, a HCP would be available to discuss specific topics selected from conversations published on the Forum.<br>• Specialist answers – information on topics suggested by users.<br>• Testimonials - users could share stories and comment on other users' stories. |



For peer review only - http://bmjopen.bmj.com/site/about/guidelines.xhtml

| | | | |
|---|---|---|---|
| | | | • Ability for users to request information they felt lacked on the website. |
| Get Well Fast (37) | • Employees of KLM Royal Dutch Airlines or National Railways and their OPs.<br>Employee criteria:<br>• Contracted for at least 12 hours per week.<br>• Absent from work for a minimum of 2 weeks due to non-specific back or neck pain.<br>• No serious health problems defined as “warning flags: e.g. fever, pain in arms or legs, serious disease”.<br>• Ability to speak and write in Dutch.<br>• Internet access. | • Users of the Get Well Fast website.<br>• The employees’ OPs.<br>• All employees using the website and OPs were invited to participate in an interview.<br>• Convenience sample | • Web-based, 5-weeks programme during which the employee completed 4 questionnaires and received tailored information via a personal digital diary.<br>• Based on weekly questionnaires, information about advice on improving physical fitness, setting a daily timetable, pain-coping strategies, and exercise instructions is provided.<br>• Employees spent around 15 minutes/day reading information, completing questionnaires, and following exercises.<br>• Employee’s OP had access to the employee's diary and received reports when the employee completed a questionnaire, detailing the employee’s condition, current treatments, and absence details. |
| MyBehaviorCBP (38) | • Aged 18-65 years<br>• History of chronic back pain (≥6 months).<br>• Willingness to use MyBehaviorCBP app on an Android mobile phone (own or provided by study).<br>• Reasonable level of outdoor movement (e.g. travelling to and from work).<br>• Not being significantly housebound.<br>• Fluent in English<br>• Basic level of mobile proficiency. | • All participants received web-based exit survey; one question was open ended and results from this component of the study are included in this review. | • 5-week app based programme during which participants received recommendations for PA.<br>• App tracks participant’s mobility state and geolocation using in-phone sensors or manual input. Recurring patterns of PA form base for new PA recommendations.<br>• Week 1 - baseline period: no recommendations were given.<br>• Week 2 & 3 - control phase: PA recommendations were random, generic and unrelated to participants’ past behaviour. |



For peer review only - http://bmjopen.bmj.com/site/about/guidelines.xhtml

| | | | |
|---|---|---|---|
| | | | • Week 4 & 5 – experimental phase: PA recommendations generated by MyBehaviorCBP based on PA behaviour during control phase.<br>• Participants were blinded to when the different PA recommendation forms were activated. Participants completed a daily in-phone survey regarding ease of following recommendations, how many recommendations they followed, and their emotional state. |
| Web-BCPA (39) | • Aged 18-63 years.<br>• Persistent musculoskeletal pain with duration of at least 3 months in the back, neck, shoulder, and/or generalised pain.<br>• OMPSQ score ≥90, screening for psychosocial factors that indicates an estimated risk for long-lasting pain and future disability (40).<br>• Work ability of at least 25% (assessment method N/R).<br>• Familiar with written and spoken Swedish.<br>• Internet and computer access. | • Participants must have spent at least 15 minutes per module in 5 of 8 modules.<br>• Participants had to have reached their 4-month follow-up assessment<br>• Participants contacted consecutively with information about interview study in conjunction with 4-month follow-up.<br>• Formal invitation subsequently via telephone. | • Website-based Web Behavior Change Program for Activity (Web-BCPA) in combination with MMR.<br>• Web-BCPA consisted of eight modules: 1) pain, 2) activity, 3) behavior, 4) stress and thoughts, 5) sleep and negative thoughts, 6) communication and self-esteem, 7) solutions, and 8) maintenance and progress.<br>• Modules contained information, assignments and exercises delivered as educational texts, videos and writing tasks.<br>• Participants could access 1 new module/week during the first 8 weeks of rehabilitation, and had access to the website 24/7 for 4 months. |

**HCP**: healthcare professional; **OP**: occupational physician; **OMPSQ**: Örebro musculoskeletal pain screening questionnaire; **MMR**: multimodal rehabilitation; **PA**: physical activity; **N/R**: not reported



For peer review only - http://bmjopen.bmj.com/site/about/guidelines.xhtml

## Qualitative components of included studies

Sampling procedures used for the qualitative component of the included studies (Table 4) were described for three of the studies as an invitation to participants to take part in an interview (28, 29, 37). Several sampling strategies were utilised, including purposive (28, 29) and convenience sampling (28, 29, 37), while in another study participants were sampled consecutively (39). In the further study, where the qualitative component was part of a self-administered survey, all participants took part (38). Qualitative interviews were conducted via telephone (37), in the participant's home (28, 29, 39), or at a local university (28, 29), health care centre (39) or council building (39). All of the interviews were semi-structured, recorded and either transcribed verbatim (28, 29, 39) or as written descriptions of answers including quotes (37). For the MyBehaviorCBP study (38), free-text answers from the electronic exit survey were extracted. Data was then analysed inductively (28), using grounded theory (29), thematically (37, 38) and using content analysis (39) to identify common themes. Just one article (29) referred to data collection and analysis continuing until data saturation was achieved.

## Quality appraisal

The comprehensiveness of reporting varied across the included studies (Supplementary File 2) and ranged from 12 (38%) to 21 (67%) of the 32-item COREQ checklist (28, 39). Items within domain 1 (Research team and reflexivity) generally had very poor reporting with several items not reported by any studies, for example researcher occupation and experience and training were not reported by any of the included studies. All studies reported sampling procedure, sample size, setting of data collection, description of sample, recording, derivation of themes, quotations presented, consistency of data and findings and clarity of major themes

## Engagement strategies



For peer review only - http://bmjopen.bmj.com/site/about/guidelines.xhtml

We defined engagement strategies as any method used to recruit and initially motivate participants to enrol in the DHI study. The identified engagement strategies included: use of mailing lists of retired personnel (38); mailing list for a university wellness centre (38); or invitation from OP or HCP (28, 29, 37). In addition, the Oneself study advertised for participation through media: radio (project leader and managers interviewed about project at local radio station), television (rheumatologists involved in project spoke about project on local television station), and through a press conference for which the major daily journals from the area were invited (28, 29).

Barriers and facilitators for uptake and utilisation of digital health interventions

We identified four major themes: 1) IT usability and accessibility, 2) Quality and amount of content, 3) Tailoring and personalisation, and 4) Motivation and support (Table 5). Under each theme, both barriers and facilitators were identified. Distinction between uptake (initial engagement) and utilisation (use) in the included studies was not possible, and they are therefore treated as one. Participant quotes are provided in the text to substantiate the data for each theme. More exemplar quotations are provided in Supplementary File 3.

**Table 5:** Factors affecting uptake and utilisation of DHIs for self-management of LBP

| Theme | Subtheme | Barriers | Facilitators |
|---|---|---|---|
| **IT usability and accessibility** | Functionality and usability | • Too much choice between functions<br>• Fixed advancement pace<br>• Issues logging into DHI<br>• *Low user-friendliness<br>• *Issues logging into DHI<br>• *Low level of functionality (e.g. registration, navigation, helpdesk) | • Flexible structure and navigation<br>• Conveniently arranged<br>• Variation of media types (text, audio and video)<br>• Reminders and notifications<br>• High user-friendliness<br>• *High user-friendliness |
| | IT affinity | • Lack of affinity with computers<br>• *Lack of affinity with web-based programmes | • Enjoying working with a computer |
| | Access and convenience | • Not able to choose starting time of DHI | • Easily accessible with low effort |



For peer review only - http://bmjopen.bmj.com/site/about/guidelines.xhtml

| | | | |
|---|---|---|---|
| | | • *No access to computer during consultation | • Accessible at all hours and locations<br>• Accessible even during periods with severe pain symptoms<br>• Ability to take all the time needed |
| **Quality and amount of content** | Quality of content | • Contradictory content between DHI and HCP | • Trustworthy content and source<br>• Easily understandable content<br>• High quality of content<br>• Steady content<br>• *Appropriate content |
| | Amount of content | • Too much content to choose from<br>• Too much information to fully comprehend | • A lot of content to choose from |
| **Tailoring and personalisation** | Tailoring, specificity and personalisation | • Content not tailored to individual needs and/or pain severity<br>• Content perceived not new or relevant | • Content accounting for individual needs and/or pain severity<br>• Self-identification in content<br>• Opportunity to influence treatment |
| **Motivation and support** | Personal attributes and resources | • Adhering to biomedical model of LBP<br>• Seeing LBP as a marginal problem<br>• Preferring other treatment regimens, e.g. with human contact<br>• Lack of knowledge about LBP and treatments<br>• Physical health (e.g. pain, fatigue)<br>• Psychological symptoms | • High level of awareness and self-management of LBP<br>• Aware that LBP would not be fixed with a medical solution and ready to accept active role<br>• Emotional and cognitive resources, e.g. motivation, interest, commitment and self-confidence in self-management of LBP<br>• Enjoy solution focused work |
| | Support to use DHI | • HCP unsupportive of use of DHI<br>• No support from authorities | • HCP supportive of use of DHI<br>• Support from family<br>• Support from authorities<br>• Support from other suffers (e.g. successful testimonials) |
| | Features of DHI | • DHI not guiding or supporting participants enough (e.g. to plan for execution of physical activity recommendation from DHI) | • Interaction/interactivity<br>• Information about self-management of LBP<br>• Goal-setting<br>• Action-planning<br>• Follow-up and evaluation |



For peer review only - http://bmjopen.bmj.com/site/about/guidelines.xhtml

| | | | |
|---|---|---|---|
| | | | • Adjusting treatment related to setbacks and progress<br>• Monitoring own progress in graphs<br>• Variation of content<br>• Update of content |
| | HCP factors for support of patients | • *Time restrictions of consultations<br>• *Difficulty keeping DHI in mind during consultations<br>• *Difficulty providing patients with accurate information about DHI<br>• *Perceiving no benefit of DHI compared to usual treatment<br>• *Preferring other treatment regimens, e.g. with human contact | • *DHI a good medium for counselling employees |

*: Occupational physician perspective; **IT**: information technology; **HCP**: healthcare professional; **DHI**: Digital health intervention

*1) IT usability and accessibility*

The first theme that emerged concerned functionality and usability, IT affinity or access and convenience of the DHI. A flexible and convenient structure with high user-friendliness aided use of DHIs (37, 39). Inclusion of a variety of media types such as video was also appreciated (28, 29) as well as getting reminders or notifications from the DHI (28, 29).

*"Usually I went on the website when I read the newsletter. I read the letter and then I'm there, it's like a conditioned reflex (Woman, 49, nurse)"* (28, 29).

On the other hand, low user-friendliness and problems with logging in were barriers for use of DHIs for both study participants and HCPs (37). A fixed starting point or set advancement pace were also demotivating for some users (39). Affinity with computers and web-based programmes highly affected uptake of DHIs. Participants with a high level of computer affinity and who enjoyed working on a computer expressed positive feelings towards using DHIs (39), whereas lack of computer affinity was an important barrier for uptake of the intervention (37). Accessibility to a computer was surprisingly not a requirement

For peer review only

for uptake to the study. When computers were readily available, DHIs were considered easy to access with unlimited 24h access (28, 29, 39).

*"… thanks to the program (the Web-BCPA) I was able to perform the basic body awareness exercises of my own choice… and to repeat those that I felt most effective as many times that I preferred… the flexibility made it mine (the rehabilitation) (Woman, participant)"* (39).

Even during periods with severe pain symptoms, a DHI was considered an attainable and effortless option as participants did not have to go anywhere (e.g. a healthcare centre) (28, 29, 39).

*2) Quality and amount of content*

Quality and amount of content provided in DHIs affected use for both participants and HCPs. Trustworthiness of the source and information provided facilitated use, and participants seemed to be reassured when knowing the content had been reviewed and validated by HCPs (28, 29, 39). For participants, richness and consistency of content facilitated use (28, 29), especially when the content was easily understandable (37).

*"Knowing that there is a serious website where there are contributions, it strengthens you a bit (Woman, 37, teacher"* (29).

Likewise, content that suited the patients was appreciated by HCPs (37). On the other hand, when participants experienced contradictory advice from their HCP and the DHI, this was a barrier for using the DHI (37). Large volumes of information or too much content to choose from also limited uptake and utilisation, particularly in relation to the amount of time required to go through it (28, 29, 37).

*"There is a lot of information, probably almost too much, don't you think? (Man, 47, bank director)"* (28, 29).

*3) Tailoring and personalisation*



For peer review only - http://bmjopen.bmj.com/site/about/guidelines.xhtml

The participants' perception of the degree of tailoring and personalisation of the content to their needs was the third major theme affecting use of DHIs for self-management of LBP. Self-identification increased utilisation of DHIs when participants were able to recognise themselves in the content, e.g. in the information and explanations about pain and symptoms, or thoughts related to dealing with LBP (28, 29, 39).

*"It gives you descriptions and you say: this stuff here... I see it, I see it! I recognise myself in it, I recognise myself here (Man, 58, teacher)"* (28, 29).

When the content of the DHI accounted for the individual participant's activities, needs or pain severity it further encouraged use of the DHI (37-39).

*"I really liked the personalization. I thought it was a nice touch. Suggestions were more specific and tailored, which for me made them more relevant and likely for me to use them (Participant)"* (38).

Participants appreciated the opportunity to influence their own rehabilitation by being able to select exactly what they wanted from a variety of options that fitted their situation (38, 39).

*"Previously I had read about CBT (Cognitive Behavioral Therapy), but I had never thought of it as a help for my condition... I want to compare this rehabilitation with a smorgasbord from which is it easy to taste (Participant)"* (39).

When content was not tailored to the individual participant or the participant's pain severity, it was experienced as a barrier for use of the DHI as it was not perceived to apply to their situation. This in turn would negatively impact the participant's motivation and sustained engagement (29, 37). Content that was not perceived relevant or new to the participant could also lead to a feeling of hopelessness as participants' got the impression that there was no solution to their problem (29).

### 4) *Motivation and support*

The fourth major theme related to the participants motivation and support, and included subthemes related to the personal attributes and resources of participants, support to use DHIs, features of DHIs, and

For peer review only

lastly HCPs' perceptions and how they affect HCPs' support of DHIs. Specific participant attributes impacted the utilisation of DHIs; already being involved or being ready to accept an active role in rehabilitation (28), and having motivation, interest, commitment and confidence in self-managing LBP facilitated use (28, 29, 39). Enjoying solution focused work, e.g. as experience from day job, was also a facilitator (39). Contrary, not wanting to take an active role (28), or preferring other treatment regimens (28) hindered use, as well as lacking information about treatments (39) or preferring other available treatment regimens, e.g. with human contact (37). Relying on a HCP to find a solution (28, 29) or seeing LBP as only a marginal problem, led to lower motivation for use of the DHI (28). Furthermore, use of DHIs was constrained by physical (37, 39) or psychological (39) restrictions. Getting support from a variety of sources facilitated use; both support from outside and within the DHI. Support from family, authorities and HCPs was perceived as encouraging (39), and so were successful testimonials from other users whose LBP symptoms had improved (28, 29).

*"When you are going through a moment when you have backache and you read a testimony which says 'yes, there is someone who was able to do it', it gives you hope (Woman, 28, academic researcher)* (28, 29).

Not having HCPs or local agencies (e.g. authorities) support in their use of the DHI held participants back from utilising DHIs to manage their LBP (37, 39).

*"I expected more commitment from my OP [occupational physician] (Employee)"* (37).

Features of DHIs could both facilitate and restrain use. DHIs that were interactive, used goal-setting and action-planning, and had a great variation of content encouraged use (38, 39). Participants also appreciated information that guided them on how to self-manage their LBP (e.g. exercises and advice) (28, 29, 37-39), and some participants felt updates of content facilitated their use further (28, 29). Furthermore, DHIs that allowed participants to monitor and reflect on their own progress, improvement or goal attainment, e.g. through interactive graphs, were considered to enable self-management actions and to motivate further use (39). Follow-up and evaluation on goal achievement was also appreciated and reinforced the importance of tailoring DHIs towards individual participant's experience.



For peer review only - http://bmjopen.bmj.com/site/about/guidelines.xhtml

*" ... days when I had a lot of pain I used to remain sedentary, and as soon as I had a better day I was eager to do all kinds of activities that day.. before I started with the assignment activity planning (in the Web-BCPA) I was not aware of how my behaviour related to the days with pain, but by monitoring this over time I started to plan my daily activities in a more balanced way (Woman, participant)"* (39).

On the contrary, DHIs that did not support or guide participants enough, e.g. to execute recommendations given by the DHI, were perceived as constraining (38).

HCPs had reasons to support or not support participants' use of DHIs for self-management of LBP. HCPs either did not perceive additional benefits of DHIs compared to usual care or preferred other treatment regimens, e.g. ones that involved physical contact (37).

*"The ability to touch people is an essential element in the treatment of people with back or neck pain (Occupational physician)"* (37).

HCPs also reported having too little time during consultations to support use of DHI or difficulty in keeping the DHI in mind during their consultation – and even if they remembered it, they struggled with providing patients with accurate information about the DHI (37). However, HCPs who perceived DHIs as a good medium for counselling were positive about using and recommending DHIs (37).

Suggestions for improved utilisation

Participants of all included studies provided the authors with suggestions for how DHIs could be improved to facilitate continued or improved utilisation. As these items were only perceived as potential facilitators if implemented they are reported separately from the themes above. Some suggestions were improvement of usability of existing DHIs, e.g. increased user-friendliness (37), incorporation of illustrations and cartoons (37), or easier registration (37). Optimisation of tailoring to adjust for changes over time (37), or better adaption of physical activity recommendations that accommodated differences between weekdays and accounted for weather forecasts was also suggested (38). System improvements that enabled the DHI to learn from participants' activity level related to their pain days was also proposed (38). Lastly, application of



For peer review only - http://bmjopen.bmj.com/site/about/guidelines.xhtml

a participatory approach for the process of designing DHIs was suggested (39). Other suggestions were new features to add to DHIs, e.g. direct contact to HCPs via DHI (37), a helpdesk (37), content about how to deal with LBP mentally (37), and a sophisticated reminder system with just-in-time notifications for both planning and execution of physical activities (38).

Developing a conceptual understanding

We applied the NPT framework (Table 2) to the taxonomy of barriers and facilitators as summarised in Table 5. Most of the identified codes fell within the four NPT constructs, with the exception of codes related to participants' own physical, mental and emotional health, which although affecting an individual's capacity, they are not specific actionable tasks involved in the uptake and utilisation of a DHI for LBP. Applying the NPT framework allowed us to conceptualise how the codes identified may affect the uptake and utilisation of DHIs for the self-management of LBP (Figure 2), at both an individual and collective level, through the four stages of deciding whether to enrol, engage, utilise and maintain engagement with such a tool.

**Figure 2** Preliminary conceptual model of barriers and facilitators to uptake and utilisation of LBP DHIs

## Discussion

We have conducted a systematic search of the literature to explore the methods used to encourage participation with DHIs for the self-management of LBP and the barriers and facilitators to patient uptake and utilisation of these tools. Our review identified four studies published in five articles, demonstrating that the literature remains sparse.

Our review has enabled us to develop a preliminary conceptual model for engagement and utilisation of a DHI for LBP self-management by applying the NPT framework to the barriers and facilitators identified in the included studies. The model suggests that users value DHIs that are easily understandable, which they can navigate at their own pace and which help enhance subsequent communication with HCPs, family and



For peer review only - http://bmjopen.bmj.com/site/about/guidelines.xhtml

colleagues. Providing regular updates and prompts appears to help users engage with DHIs whilst the ability to interact with other users is viewed positively in terms of providing support, motivation and validation. Users expect information to be easily accessible, structured, up-to-date and accurate, with tailoring to individual user experience being particularly valued.

Conversely, large volumes of information and lack of time appear to have a negative impact on user understanding, motivation and engagement. Lack of support or encouragement by HCPs also appears to be off putting for some whilst others face challenges accessing the DHIs. Participant's own attributes including the symptoms they experienced and their attitudes and preferences for treatment for LBP can further restrict capacity to self-manage and influence motivation and engagement with DHIs. Other significant barriers to user engagement and utilisation include missing or conflicting information, content that was not tailored to the individual, and lack of feedback or evaluation.

In this review we explored how studies engaged participants to enrol into the study and begin using a DHI, this was mainly through identification of potential participants and subsequent invitation. Sustaining engagement beyond initial participation was not discussed in-depth in any of the included studies, some used email prompts and regular updates or newsletters. However, all studies did report participants' suggestions to improve DHIs, which mainly focussed on improving usability, (dynamic) tailoring of content, additional features to support users and the inclusion of participants in the design of DHIs. While not considered as facilitators to uptake and utilisation, some positive consequences of using the DHIs were identified by some users, e.g. acquiring a vocabulary and an individual understanding of their situation, and increased confidence in self-managing their LBP, which may have reinforced users in their self-management and in turn may have increased use of DHIs. Further, some general points to increase utilisation of DHIs for LBP were highlighted by participants, including the importance of participatory involvement of patients in the development of a DHI.



For peer review only - http://bmjopen.bmj.com/site/about/guidelines.xhtml

Comparison with previous literature

Although there was a significant variation in intervention recruitment and content in studies included in our review, there was a large degree of overlap in terms of the barriers and facilitators identified. Many of these are generally in keeping with the findings of other qualitative reviews for DHIs in general (19, 41) as well as those looking specifically at hypertension (42) and pain management in older adults (43). A review by O'Connor et al (19) identified four main themes relating to barriers and facilitators to engagement and recruitment to DHIs in general: personal agency and motivation; personal life and values; engagement and recruitment approach, and quality of the DHI. Another review by Hardiker & Grant (41) identified five overarching themes concerning barriers and facilitators influencing engagement with eHealth services: characteristics of users; technological issues; characteristics of eHealth services; social aspects of use; and eHealth services in use. Despite the differing terminology of the major theme headings used in these studies and those found in this review, comparison of the codes or subthemes reveals the barriers and facilitators to be broadly similar, suggesting that these may be generally transferable across DHIs. The main exception is the specific mention of security and privacy of personal information in these earlier reviews (19, 41), which was not found as a barrier in this review, although this may be due to the small number of studies in our review compared to O'Connor et al (19) and Hardiker & Grant (41), reviews which included 19 and 50 studies, respectively.

*Functionality and general IT issues*

Factors including age, ethnicity, economic status, level of educational attainment and familiarity with the internet are recognised as being significant factors influencing access to and engagement with DHIs (41). O'Connor et al. (19) reported that a lack of digital literacy, issues accessing IT equipment or the internet and the cost of such equipment or access are barriers to the use of DHIs. The user friendliness, design and ease of registration/logging in to a DHI were found to be significant issues for users in this review and should be carefully considered when planning a DHI.



For peer review only - http://bmjopen.bmj.com/site/about/guidelines.xhtml

*Quality and amount of content*

Trust is a significant issue when accessing information online (41). Clinical endorsement seems to be important to users in terms of the perceived quality of content and is in keeping with the findings of other studies in this area (19, 44). Additionally, consideration should be given to the potential for users to receive contradictory advice from the DHI and their HCP. Our findings suggest that whilst some users considered large volumes of information as a barrier, others valued the ability to read widely on the subject. This is thought to reflect individual preference and personal factors such as time pressures. Taking such preferences into account during the development and delivery of DHIs may increase user engagement.

*Tailoring and personalisation*

It is clear from our findings that user's symptomology, prior knowledge and experience play a role in engagement. Tailoring DHIs to the user's individual symptoms and functional limitations is thought to enhance engagement (19) and may thus improve the effectiveness of the intervention. A recent review of DHIs for the self-management of LBP (17) found that no DHI for LBP used tailoring to enhance effectiveness, but commented that this could be an important means of enhancing engagement. In addition, O'Connor et al. (19) recommended that any DHI should be designed and tailored to individual needs in order to reduce the self-care burden. Our findings suggest that users improved understanding of LBP and enhanced communication with their HCP during subsequent consultations. Some users commented that they would have appreciated some direct support from a HCP or that this might have enhanced engagement. This finding is consistent with those of Steele et al (45), who during an evaluation of an internet-based physical activity behaviour change program, found that many participants in the internet group would have preferred traditional face-to-face sessions. Some of the occupational physician's interviewed felt that they did not have the time and capacity within their consultation to discuss DHI use in detail (37). If the intended purpose of a DHI is to facilitate HCP – patient communication then how the DHI



For peer review only - http://bmjopen.bmj.com/site/about/guidelines.xhtml

or a supporting HCP dashboard could be designed to allow for efficient and useful interactions during a consultation should be considered at the design and development stage.

*Motivation and support*

Personal recommendations and social support were recognised as being important in encouraging DHI user registration and in fostering engagement (19). We found that some users valued the emotional support of being able to interact with other users. Whilst this was a positive finding in our study and is consistent with those reported elsewhere (41), there exists the possibility of potentially abusive or threatening behaviours developing online which could act as a barrier to some (46). Other reports of discussion threads deviating from the original topic or containing misleading information (41) raise questions on the need for monitoring such interactive features. Our findings further suggest that an individual's personal attributes and resources (e.g. emotional and cognitive) and attitudes towards self-management can influence their use of DHIs. Additional support may therefore be required for some potential users to participate and benefit from DHIs.

O'Connor et al (19) reported that some individuals do not view technology as a way of addressing healthcare needs and prefer alternative approaches to managing their health issues such as seeking support from family, friends or healthcare professionals. They also highlight the potential for DHIs to be impersonal and commented on the lack of a therapeutic relationship, particularly in situations where sensitive health or social issues are involved. Such views were also reflected among individuals, including some HCPs, in our findings. In contrast, other users appreciate the freedom to access health information at a time and place that suits the user along with the anonymity DHIs can offer (44), issues that can be challenging for traditional healthcare services to match.

Strengths and limitations



For peer review only - http://bmjopen.bmj.com/site/about/guidelines.xhtml

This systematic review was conducted by an experienced team and follows the PRISMA guidelines for the reporting of systematic reviews. Our iterative search strategy utilised multiple databases and involved independent data extraction, quality appraisal and data analysis by two reviewers, with a third reviewer adjudicating in the case of any disagreements.

Our review does however have some limitations. Many DHIs are developed commercially and do not undergo formal academic evaluation (15) resulting in relatively sparse literature in this area. Our search strategy involved several eligibility criteria, including that studies must be published in peer-reviewed academic journals, and as such we did not identify any grey literature. However, it is unlikely that such findings, if available, would have held scientific rigour and added to the findings of this review. Further, as our analysis and synthesis of data was based on reviewing published literature, not the original data, this could have impacted on the background context to some of the quotes used in this manuscript.

The studies included in this review (28, 29, 37-39) were conducted in real-life settings and as a result sampling procedures were acknowledged as being convenient, had the potential to be biased towards individuals who found the interventions beneficial and may not have been representative of all users. Furthermore, the literature contained very limited information on user's sociodemographic characteristics. However, as a consequence of the small number of studies identified by our search strategy, we did not exclude studies on the basis of quality, potentially reducing the reliability of the findings of this review.

Finally, due to the lack of literature in this field, our conceptual model for the update and utilisation of DHIs to support the self-management of LBP is limited to four studies to date. It is possible that not all the important barriers and facilitators may have been identified, and thus our conceptual model must be considered preliminary. As more rigorous studies are conducted and reported this model should be further



For peer review only - http://bmjopen.bmj.com/site/about/guidelines.xhtml

developed and amended.. This information will be of particular use to those involved in designing and implementing DHIs focused on self-management of LBP and more widely.

**Conclusions**

Our systematic review highlights barriers and facilitators affecting the utilisation of DHIs for the self-management of LBP and identified key areas involved in embedding such interventions into everyday practice. The limited and varied quality of literature found by this review suggests that further primary research investigating the implementation of DHIs and user's experiences is required. Future research should aim to describe DHIs and their users in more detail and include descriptions of engagement strategies and barriers or facilitators encountered in order to enhance our knowledge of which approaches are likely to have the greatest impact on user engagement and outcomes, and for whom.

**List of abbreviations**

COREQ - Consolidated Criteria for Reporting Qualitative Research

DHI - Digital health intervention

HCP – Healthcare professional

IT - Information technology

LBP - Low back pain

NPT - Normalization process theory

OP - Occupational physician

PA – Physical activity

PRISMA - Preferred reporting items for systematic reviews and meta-analyses

**Supplementary File 1:** Search details, as previously described and published by Nicholl et al. (17)



For peer review only - http://bmjopen.bmj.com/site/about/guidelines.xhtml

**Supplementary File 2:** Consensus summary of quality appraisal as per 32-item COREQ checklist and comprehensiveness of reporting

**Supplementary File 3:** Taxonomy of barriers and facilitators with exemplar quotations

## Declarations

Ethics approval and consent to participate: Not applicable

Consent for publication: Not applicable

Acknowledgements: We would like to thank our librarian adviser Ingrid Ingeborg Riphagen, Department of Public Health and Nursing, Faculty of Medicine and Health Sciences, Norwegian University of Science and Technology (NTNU).

**Contributorship statement**: Study design was developed by all authors. Title, abstract and full text screening was performed JK, MaS, KC and KW with any discrepancies being resolved by BN, CR and MeS. JK, MaS, BN and KW carried out data extraction. JK, KW and MaS conducted data synthesis and analysis aided by BN and FM. Quality appraisal was assessed by BN, KC and KW. LS, CR and MeS critically scrutinized first drafts and provided comments. All authors read, commented and approved the final manuscript.

**Competing interests:** The authors declare that they have no competing interests

**Funding**: This project has received funding from the European Union Horizon 2020 Research and innovation programme under grant agreement No 689040.

**Data sharing statement**: The datasets used and analysed during the current study are available from the corresponding author on reasonable request.

## References

1. Hoy D, Bain C, Williams G, March L, Brooks P, Blyth F, et al. A systematic review of the global prevalence of low back pain. Arthritis and rheumatism. 2012;64(6):2028-37.
2. Vos T, Flaxman AD, Naghavi M, Lozano R, Michaud C, Ezzati M, et al. Years lived with disability (YLDs) for 1160 sequelae of 289 diseases and injuries 1990-2010: a systematic analysis for the Global Burden of Disease Study 2010. Lancet (London, England). 2012;380(9859):2163-96.

For peer review only

3. Nolet PS, Kristman VL, Cote P, Carroll LJ, Cassidy JD. Is low back pain associated with worse health-related quality of life 6 months later? European spine journal : official publication of the European Spine Society, the European Spinal Deformity Society, and the European Section of the Cervical Spine Research Society. 2015;24(3):458-66.
4. Dagenais S, Caro J, Haldeman S. A systematic review of low back pain cost of illness studies in the United States and internationally. The spine journal : official journal of the North American Spine Society. 2008;8(1):8-20.
5. Maniadakis N, Gray A. The economic burden of back pain in the UK. Pain. 2000;84(1):95-103.
6. (NICE) NIfHaCE. Low back pain and sciatica in over 16s: assessment and management: National Institute for Health and Care Excellence (NICE). 2016 [Available from: https://www.nice.org.uk/guidance/ng59.
7. Authority DH. National clinical guideline: Interventions for recent onset low back pain [in Danish]: Danish Health Authority; 2016 [Available from: https://www.sst.dk/da/udgivelser/2016/nkr-laenderygsmerter.
8. Sluijs EM, Kok GJ, van der Zee J. Correlates of exercise compliance in physical therapy. Physical therapy. 1993;73(11):771-82; discussion 83-6.
9. Newson JT, Huguet N, Ramage-Morin PL, McCarthy MJ, Bernier J, Kaplan MS, et al. Health behaviour changes after diagnosis of chronic illness among Canadians aged 50 or older. Health reports. 2012;23(4):49-53.
10. Murray E, Burns J, See TS, Lai R, Nazareth I. Interactive Health Communication Applications for people with chronic disease. The Cochrane database of systematic reviews. 2005(4):Cd004274.
11. Beatty L, Lambert S. A systematic review of internet-based self-help therapeutic interventions to improve distress and disease-control among adults with chronic health conditions. Clinical psychology review. 2013;33(4):609-22.
12. Zhao J, Freeman B, Li M. Can Mobile Phone Apps Influence People's Health Behavior Change? An Evidence Review. Journal of medical Internet research. 2016;18(11):e287.
13. Hayden JA, van Tulder MW, Tomlinson G. Systematic review: strategies for using exercise therapy to improve outcomes in chronic low back pain. Annals of internal medicine. 2005;142(9):776-85.
14. Strecher V. Internet methods for delivering behavioral and health-related interventions (eHealth). Annual review of clinical psychology. 2007;3:53-76.
15. Lalloo C, Jibb LA, Rivera J, Agarwal A, Stinson JN. "There's a Pain App for That": Review of Patient-targeted Smartphone Applications for Pain Management. The Clinical journal of pain. 2015;31(6):557-63.
16. Garg S, Garg D, Turin TC, Chowdhury MF. Web-Based Interventions for Chronic Back Pain: A Systematic Review. Journal of medical Internet research. 2016;18(7):e139.
17. Nicholl BI, Sandal LF, Stochkendahl MJ, McCallum M, Suresh N, Vasseljen O, et al. Digital Support Interventions for the Self-Management of Low Back Pain: A Systematic Review. Journal of medical Internet research. 2017;19(5):e179.
18. Machado GC, Pinheiro MB, Lee H, Ahmed OH, Hendrick P, Williams C, et al. Smartphone apps for the self-management of low back pain: A systematic review. Best practice & research Clinical rheumatology. 2016;30(6):1098-109.
19. O'Connor S, Hanlon P, O'Donnell CA, Garcia S, Glanville J, Mair FS. Understanding factors affecting patient and public engagement and recruitment to digital health interventions: a systematic review of qualitative studies. BMC medical informatics and decision making. 2016;16(1):120.
20. International Register of Systematic Reviews P. [Available from: https://www.crd.york.ac.uk/PROSPERO/display_record.php?ID=CRD42016051182.
21. Moher D, Liberati A, Tetzlaff J, Altman DG. Preferred reporting items for systematic reviews and meta-analyses: the PRISMA statement. International journal of surgery (London, England). 2010;8(5):336-41.
22. McLean G, Band R, Saunderson K, Hanlon P, Murray E, Little P, et al. Digital interventions to promote self-management in adults with hypertension systematic review and meta-analysis. Journal of hypertension. 2016;34(4):600-12.

For peer review only



For peer review only - http://bmjopen.bmj.com/site/about/guidelines.xhtml

23. Fleming T, Bavin L, Lucassen M, Stasiak K, Hopkins S, Merry S. Beyond the Trial: Systematic Review of Real-World Uptake and Engagement With Digital Self-Help Interventions for Depression, Low Mood, or Anxiety. Journal of medical Internet research. 2018;20(6):e199.
24. Carolan S, Harris PR, Cavanagh K. Improving Employee Well-Being and Effectiveness: Systematic Review and Meta-Analysis of Web-Based Psychological Interventions Delivered in the Workplace. Journal of medical Internet research. 2017;19(7):e271.
25. Booth A, Hannes K, Harden A, Noyes J, Harris J, Tong A. COREQ (Consolidated Criteria for Reporting Qualitative Studies). In: Moher D, Altman DG, Schulz KF, Simera I, Wager E, editors. Guidelines for reporting health research: A user's manual. Oxford: Wiley; 2014. p. 214-26.
26. Tong A, Sainsbury P, Craig J. Consolidated criteria for reporting qualitative research (COREQ): a 32-item checklist for interviews and focus groups. International journal for quality in health care : journal of the International Society for Quality in Health Care. 2007;19(6):349-57.
27. Dixon-Woods M, Sutton A, Shaw R, Miller T, Smith J, Young B, et al. Appraising qualitative research for inclusion in systematic reviews: a quantitative and qualitative comparison of three methods. J Health Serv Res Policy. 2007;12(1):42-7.
28. Schulz PJ, Rubinelli S, Zufferey MC, Hartung U. Coping with Chronic Lower Back Pain: Designing and Testing the Online Tool ONESELF. Journal of Computer-Mediated Communication. 2010;15(4):625-45.
29. Caiata Zufferey M, Schulz PJ. Self-management of chronic low back pain: an exploration of the impact of a patient-centered website. Patient education and counseling. 2009;77(1):27-32.
30. Thomas J, Harden A. Methods for the thematic synthesis of qualitative research in systematic reviews. BMC medical research methodology. 2008;8:45.
31. Graneheim UH, Lundman B. Qualitative content analysis in nursing research: concepts, procedures and measures to achieve trustworthiness. Nurse Education Today. 2004;24(2):105-12.
32. Krippendorff K. Content Analysis: An Introduction to Its Methodology. 2nd ed. Thousand Oaks, CA: SAGE Publications; 2004. 422 p.
33. May C, Finch T. Implementing, Embedding, and Integrating Practices: An Outline of Normalization Process Theory. Sociology. 2009;43(3):535-54.
34. May CR, Mair F, Finch T, MacFarlane A, Dowrick C, Treweek S, et al. Development of a theory of implementation and integration: Normalization Process Theory. Implementation Science. 2009;4(1):29.
35. Mair FS, May C, O'Donnell C, Finch T, Sullivan F, Murray E. Factors that promote or inhibit the implementation of e-health systems: an explanatory systematic review. Bulletin of the World Health Organization. 2012;90(5):357-64.
36. Gallacher K, Morrison D, Jani B, Macdonald S, May CR, Montori VM, et al. Uncovering treatment burden as a key concept for stroke care: a systematic review of qualitative research. PLoS medicine. 2013;10(6):e1001473.
37. de Jong T, Heinrich J, Blatter BM, Anema JR, van der Beek AJ. The feasibility of a web-based counselling program for occupational physicians and employees on sick leave due to back or neck pain. BMC medical informatics and decision making. 2009;9:46.
38. Rabbi M, Aung MS, Gay G, Reid MC, Choudhury T. Feasibility and Acceptability of Mobile Phone-Based Auto-Personalized Physical Activity Recommendations for Chronic Pain Self-Management: Pilot Study on Adults. Journal of medical Internet research. 2018;20(10):e10147-e.
39. Nordin C, Michaelson P, Eriksson MK, Gard G. It's About Me: Patients' Experiences of Patient Participation in the Web Behavior Change Program for Activity in Combination With Multimodal Pain Rehabilitation. Journal of medical Internet research. 2017;19(1):e22-e.
40. Linton S. Early assessment of psychological factors: the Örebro Screening Questionnaire for Pain. In: Linton S, editor. New avenues for the prevention of chronic musculoskeletal pain. Amsterdam: Elsevier; 2002.
41. Hardiker NR, Grant MJ. Factors that influence public engagement with eHealth: A literature review. International journal of medical informatics. 2011;80(1):1-12.



For peer review only - http://bmjopen.bmj.com/site/about/guidelines.xhtml

42. Band R, Bradbury K, Morton K, May C, Michie S, Mair FS, et al. Intervention planning for a digital intervention for self-management of hypertension: a theory-, evidence- and person-based approach. Implementation science : IS. 2017;12(1):25.

43. Parker SJ, Jessel S, Richardson JE, Reid MC. Older adults are mobile too!Identifying the barriers and facilitators to older adults' use of mHealth for pain management. BMC geriatrics. 2013;13:43.

44. Skinner H, Biscope S, Poland B. Quality of internet access: barrier behind internet use statistics. Social science & medicine (1982). 2003;57(5):875-80.

45. Steele R, Mummery KW, Dwyer T. Development and process evaluation of an internet-based physical activity behaviour change program. Patient education and counseling. 2007;67(1-2):127-36.

46. Horvath KJ, Danilenko GP, Williams ML, Simoni J, Amico KR, Oakes JM, et al. Technology use and reasons to participate in social networking health websites among people living with HIV in the US. AIDS and behavior. 2012;16(4):900-10.

For peer review only



For peer review only - http://bmjopen.bmj.com/site/about/guidelines.xhtml

# PRISMA 2009 Flow Diagram

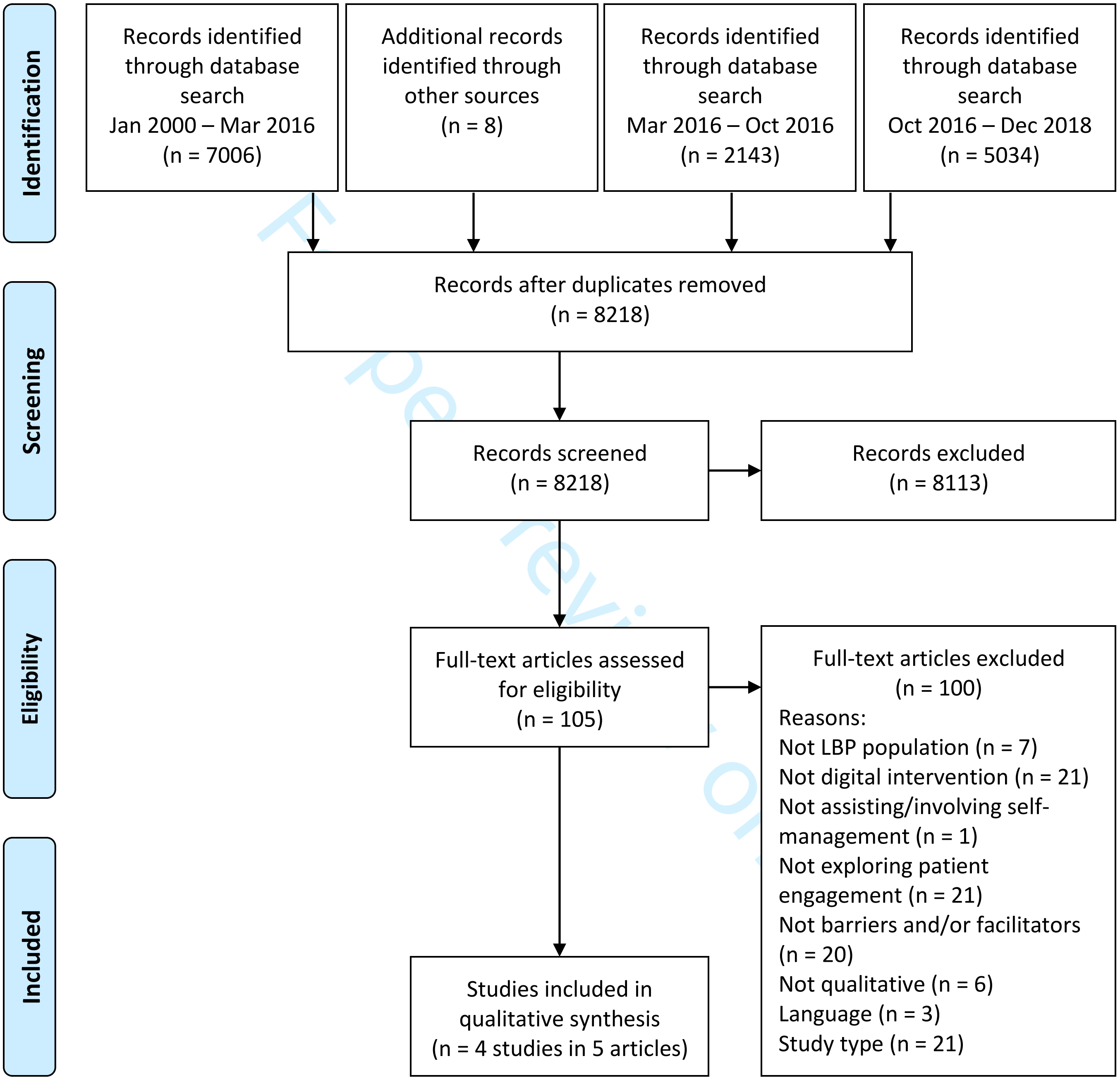


*From:* Moher D, Liberati A, Tetzlaff J, Altman DG, The PRISMA Group (2009). *P*referred *R*eporting *I*tems for *S*ystematic Reviews and *M*eta-*A*nalyses: The PRISMA Statement. PLoS Med 6(7): e1000097. doi:10.1371/journal.pmed1000097

**For more information, visit www.prisma-statement.org.**

For peer review only - http://bmjopen.bmj.com/site/about/guidelines.xhtml

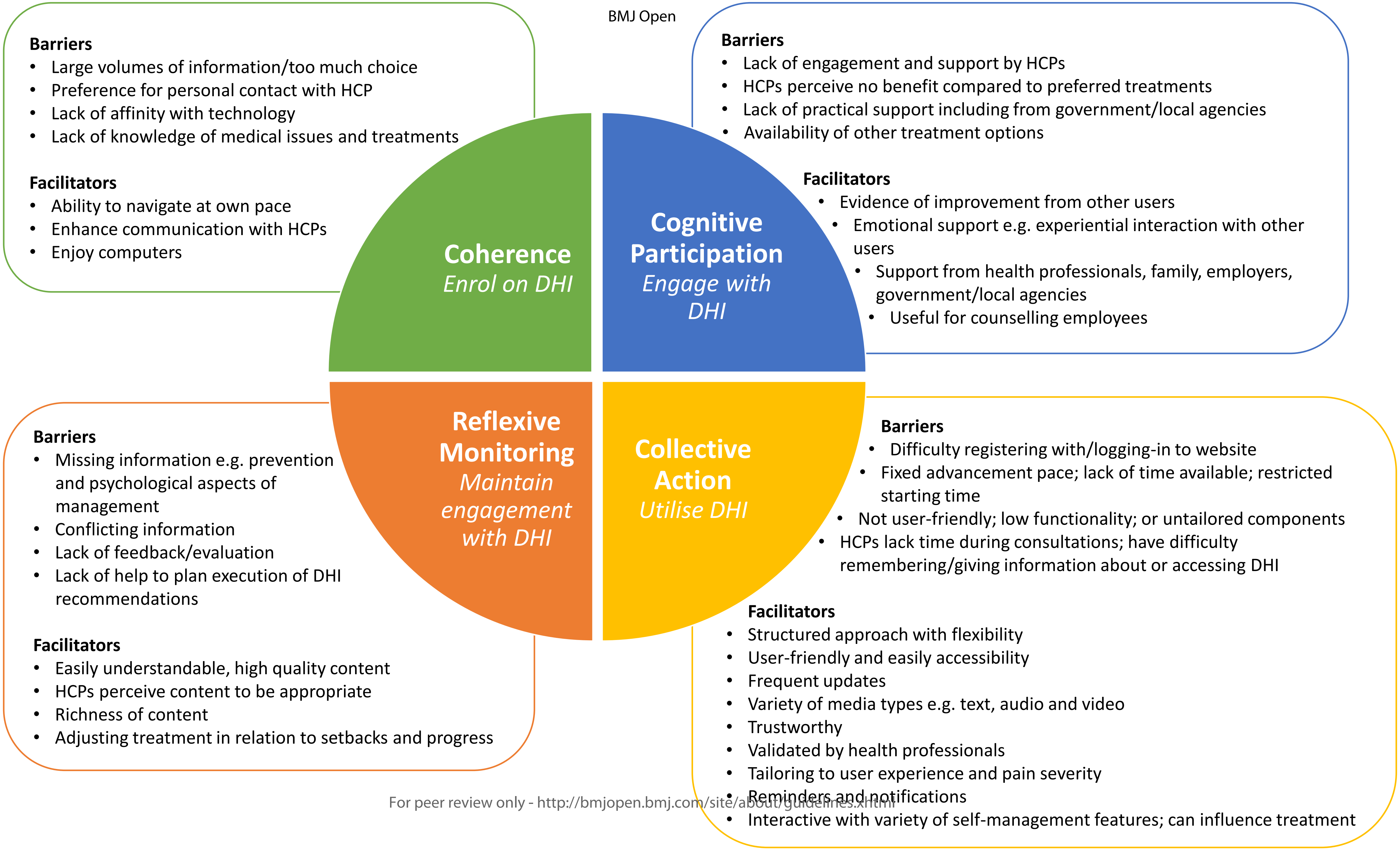
Barriers
• Large volumes of information/too much choice
• Preference for personal contact with HCP
• Lack of affinity with technology
• Lack of knowledge of medical issues and treatments
Facilitators
• Ability to navigate at own pace
• Enhance communication with HCPs
• Enjoy computers
Coherence
Enrol on DHI
Cognitive Participation
Engage with DHI
Barriers
• Lack of engagement and support by HCPs
• HCPs perceive no benefit compared to preferred treatments
• Lack of practical support including from government/local agencies
• Availability of other treatment options
Facilitators
• Evidence of improvement from other users
• Emotional support e.g. experiential interaction with other users
• Support from health professionals, family, employers, government/local agencies
• Useful for counselling employees
Reflexive Monitoring
Maintain engagement with DHI
Barriers
• Missing information e.g. prevention and psychological aspects of management
• Conflicting information
• Lack of feedback/evaluation
• Lack of help to plan execution of DHI recommendations
Facilitators
• Easily understandable, high quality content
• HCPs perceive content to be appropriate
• Richness of content
• Adjusting treatment in relation to setbacks and progress
Collective Action
Utilise DHI
Barriers
• Difficulty registering with/logging-in to website
• Fixed advancement pace; lack of time available; restricted starting time
• Not user-friendly; low functionality; or untailored components
• HCPs lack time during consultations; have difficulty remembering/giving information about or accessing DHI
Facilitators
• Structured approach with flexibility
• User-friendly and easily accessibility
• Frequent updates
• Variety of media types e.g. text, audio and video
• Trustworthy
• Validated by health professionals
• Tailoring to user experience and pain severity
• Reminders and notifications
• Interactive with variety of self-management features; can influence treatment

**Supplementary File 1:** Search details

**MEDLINE - search details**

Ovid MEDLINE(R) 1946 to March Week 1 2016

| | |
|---|---|
| 1 | exp back pain/(back pain$ or lumbago or back ache$ or backache$ or (lumbar adj2 pain$) or (spin$ adj2 pain$)).ti,ab,kw,kf. |
| 2 | computer peripherals/ or computer storage devices/ or computer terminals/ or modems/ or microcomputers/ or computers, handheld/ or minicomputers/ or attitude to computers/ or computers/ or computer systems/ or medical informatics/ or medical informatics applications/ or educational technology/ or audiovisual aids/ or telecommunications/ or multimedia/ or computer-assisted instruction/ or user-computer interface/ or hypermedia/ or video games/ or electronic health records/ or social networking/ or (computer$ or microcomputer$ or pc or pcs or mac or macs or internet or www or web or website$ or webpage$ or local area network$).ti,ab,kf. or software.ti,ab,kf. or (cellular phone$ or cellular telephone$ or mobile$ or cell phone$ or cell telephone$ or smartphone$ or smart-phone$ or smart-telephone$).ti,ab,kf. or (handset$ or hand-set$ or wireless or wire-less or wifi or wi-fi or gps or global positioning system$ or bluetooth or text messag$ or texting or sms or short messag$ or multimedia messag$ or multi-media messag$ or mms or instant messag$ or social media$ or facebook or twitter or webcast$ or webinar$ or podcast$ or wiki or wikis or app or apps or android$ or blackberr$ or apple$ or ios or iphone$ or ipad$ or s40 or symbian$ or windows).ti,ab,kf. or ((electronic$ or digital$ or device$) adj2 tablet$).ti,ab,kf. or (video$ or dvd or dvds).ti,ab,kf. or (youtube or you tube or vimeo).ti,ab,kf. or (online or on line or interactive).ti,ab,kf. or (chat room$ or chatroom$).ti,ab,kf. or (blog$1 or web-log$1 or weblog$1).ti,ab,kf. or (bulletin board$ or bulletinboard$ or messageboard$ or message board$).ti,ab,kf. or (ehealth or e-health or mhealth or m-health).ti,ab,kf. or exp telemedicine/ or mobile applications/ or (pda or pdas or personal digital).ti,ab,kf. or device-based.ti,ab,kf. or (email$ or e-mail$ or electronic mail$).ti,ab,kw,kf. |
| 3 | 1 and 2 |
| 4 | limit 3 to yr="2000 -Current" |

Updated searches: 1) October 21 2016 (not shown), 2) December 18 2018 (below):

| | |
|---|---|
| 1 | exp back pain/(back pain$ or lumbago or back ache$ or backache$ or (lumbar adj2 pain$) or (spin$ adj2 pain$)).ti,ab,kw,kf. |
| 2 | computer peripherals/ or computer storage devices/ or computer terminals/ or modems/ or microcomputers/ or computers, handheld/ or minicomputers/ or attitude to computers/ or computers/ or computer systems/ or medical informatics/ or medical informatics applications/ or educational technology/ or audiovisual aids/ or telecommunications/ or multimedia/ or computer-assisted instruction/ or user-computer interface/ or hypermedia/ or video games/ or electronic health records/ or social networking/ |
| 3 | (computer$ or microcomputer$ or pc or pcs or mac or macs or internet or www or web or website$ or webpage$ or local area network$).ti,ab,kf. or software.ti,ab,kf. or (cellular phone$ or cellular telephone$ or mobile$ or cell phone$ or cell telephone$ or smartphone$ or smart-phone$ or smart-telephone$).ti,ab,kf. or (handset$ or hand-set$ or wireless or wire-less or wifi or wi-fi or gps or global positioning system$ or bluetooth or text messag$ or texting or sms or short messag$ or multimedia messag$ or multi-media messag$ or mms or instant messag$ or social media$ or facebook or twitter or webcast$ or webinar$ or podcast$ or wiki or wikis or app or apps or android$ or blackberr$ or apple$ or ios or iphone$ or ipad$ or s40 or symbian$ or windows).ti,ab,kf. or ((electronic$ or digital$ or device$) adj2 tablet$).ti,ab,kf. or (video$ or dvd or dvds).ti,ab,kf. or (youtube or you tube or vimeo).ti,ab,kf. or (online or on line or interactive).ti,ab,kf. or (chat room$ or chatroom$).ti,ab,kf. or (blog$1 or web-log$1 or weblog$1).ti,ab,kf. or (bulletin board$ or bulletinboard$ or messageboard$ or message board$).ti,ab,kf. or (ehealth or e-health or mhealth or m-health).ti,ab,kf. or exp telemedicine/ or mobile applications/ or (pda or pdas or personal digital).ti,ab,kf. or device-based.ti,ab,kf. or (email$ or e-mail$ or electronic mail$).ti,ab,kw,kf. |
| 4 | 1 and (2 or 3) |
| 5 | limit 4 to yr="2000 -Current" |
| 6 | 5 and (201610* or 201611* or 2017* or 2018*).ed. |

**Embase - search details**

Ovid Embase (R) 1974 to 2016 March 18

| | |
|---|---|
| 1 | exp backache/ or (spinal pain$ or back pain$ or lumbago or back ache$ or backache$ or (lumbar adj2 pain$) or (spin$ adj2 pain$)).ti,ab,kw. |
| 2 | (exp backache/th or exp backache/pc or exp backache/rh or exp *backache/) not exp backache/su |
| 3 | exp communication protocol/ or computer assisted therapy/ or e-mail/ or human computer interaction/ or information technology/ or interactive voice response system/ or internet/ or mass communication/ or medical informatics/ or medical technology/ or mobile application/ or mobile phone/ or social media/ or exp telecommunication/ or exp telehealth/ or telephone/ or text messaging/ or webcast/ or wireless communication/ |
| 4 | computer storage device/ or computer terminal/ or microcomputer/ or minicomputer/ or attitude to computers/ or computer/ or computer system/ or medical information system/ or educational technology/ or audiovisual aid/ or exp multimedia/ or computer interface/ or hypermedia/ or electronic medical record/ or social networking/ |
| 5 | (computer$ or microcomputer$ or pc or pcs or mac or macs or internet or www or web or website$ or webpage$ or local area network$ or software or (cellular phone$ or cellular telephone$ or mobile$ or cell phone$ or cell telephone$ or smartphone$ or smart-phone$ or smart-telephone$) or (handset$ or hand-set$ or wireless or wire-less or wifi or wi-fi or gps or global positioning system$ or bluetooth or text messag$ or texting or sms or short messag$ or multimedia messag$ or multi-media messag$ or mms or instant messag$ or social media$ or facebook or twitter or webcast$ or webinar$ or podcast$ or wiki or wikis or app or apps or android$ or blackberr$ or apple$ or ios or iphone$ or ipad$ or s40 or symbian$ or windows) or ((electronic$ or digital$ or device$) adj2 tablet$) or (video$ or dvd or dvds) or (youtube or you tube or vimeo) or (online or on line or interactive) or (chat room$ or chatroom$) or (blog$1 or web-log$1 or weblog$1) or (bulletin board$ or bulletinboard$ or messageboard$ or message board$) or (ehealth or e-health or mhealth or m-health) or (app or apps) or (pda or pdas or personal digital) or device-based or (email$ or e-mail$ or electronic mail$)).ti,ab,kw. |
| 6 | 2 and 3 |
| 7 | limit 6 to yr="2000 -Current" |
| 8 | 1 and (3 or 4 or 5) |
| 9 | limit 8 to yr="2000 -Current" |
| 10 | 9 not 7 |

Updated searches: 1) October 21 2016 (not shown), 2) December 18 2018 (below):

| | |
|---|---|
| 1 | exp backache/ or (spinal pain$ or back pain$ or lumbago or back ache$ or backache$ or (lumbar adj2 pain$) or (spin$ adj2 pain$)).ti,ab,kw. |
| 2 | exp communication protocol/ or computer assisted therapy/ or e-mail/ or human computer interaction/ or information technology/ or interactive voice response system/ or internet/ or mass communication/ or medical informatics/ or medical technology/ or mobile application/ or mobile phone/ or social media/ or exp telecommunication/ or exp telehealth/ or telephone/ or text messaging/ or webcast/ or wireless communication/ |
| 3 | computer storage device/ or computer terminal/ or microcomputer/ or minicomputer/ or attitude to computers/ or computer/ or computer system/ or medical information system/ or educational technology/ or audiovisual aid/ or exp multimedia/ or computer interface/ or hypermedia/ or electronic medical record/ or social networking/ |
| 4 | (computer$ or microcomputer$ or pc or pcs or mac or macs or internet or www or web or website$ or webpage$ or local area network$ or software or (cellular phone$ or cellular telephone$ or mobile$ or cell phone$ or cell telephone$ or smartphone$ or smart-phone$ or smart-telephone$) or (handset$ or hand-set$ or wireless or wire-less or wifi or wi-fi or gps or global positioning system$ or bluetooth or text messag$ or texting or sms or short messag$ or multimedia messag$ or multi-media messag$ or mms or instant messag$ or social media$ or facebook or twitter or webcast$ or webinar$ or podcast$ or wiki or wikis or app or apps or android$ or blackberr$ or apple$ or ios or iphone$ or ipad$ or s40 or symbian$ or windows) or ((electronic$ or digital$ or device$) adj2 tablet$) or (video$ or dvd or dvds) or (youtube or you tube or vimeo) or (online or on line or interactive) or (chat room$ or chatroom$) or (blog$1 or web-log$1 or weblog$1) or (bulletin board$ or bulletinboard$ or messageboard$ or message board$) or (ehealth or e-health or mhealth or m-health) or (app or apps) or (pda or pdas or personal digital) or device-based or (email$ or e-mail$ or electronic mail$)).ti,ab,kw. |
| 5 | 1 and (2 or 3 or 4) |
| 6 | limit 5 to yr="2000 -Current" |

For peer review only

| | |
|---|---|
| 7 | limit 5 to yr="2016 -Current" |

**CINAHL - search details**

CINAHL (R) March 2016 through EBSCOhost

| | | |
|---|---|---|
| S6 | S1 AND S4 | |
| S5 | S1 AND S4 | |
| S4 | S2 OR S3 | |
| S3 | TI (computer* OR microcomputer* OR pc OR pcs OR mac OR macs OR internet OR www OR web OR website* OR webpage* OR "local area network*" OR software OR "cellular phone*" OR "cellular telephone*" OR mobile* OR "cell phone"* OR "cell telephone*" OR smartphone* OR smart-phone* OR smart-telephone* OR handset* OR hand-set* OR wireless OR wire-less OR wifi OR wi-fi OR gps OR "global positioning system*" OR bluetooth OR "text messag*" OR texting OR sms OR "short messag*" OR "multimedia messag*" OR "multi-media messag*" OR mms OR "instant messag*" OR "social media*" OR facebook OR twitter OR webcast* OR webinar* OR podcast* OR wiki OR wikis OR app OR apps OR android* OR blackberr* OR apple* OR ios OR iphone* OR ipad* OR s40 OR symbian* OR windows OR ((electronic* OR digital* OR device*) W2 tablet*) OR video* OR dvd OR dvds OR youtube OR "you tube" OR vimeo OR online OR "on line" or interactive OR "chat room*" OR chatroom* OR blog OR blogs OR web-log OR web-logs OR weblog OR weblogs OR "bulletin board*" OR bulletinboard$ OR messageboard$ OR "message board*" OR ehealth OR e-health OR mhealth OR m-health OR app OR apps OR pda OR pdas OR "personal digital" OR "device-based" OR email* OR e-mail* OR "electronic mail*") OR AB (computer* OR microcomputer* OR pc OR pcs OR mac OR macs OR internet OR www OR web OR website* OR webpage* OR "local area network*" OR software OR "cellular phone*" OR "cellular telephone*" OR mobile* OR "cell phone"* OR "cell telephone*" OR smartphone* OR smart-phone* OR smart-telephone* OR handset* OR hand-set* OR wireless OR wire-less OR wifi OR wi-fi OR gps OR "global positioning system*" OR bluetooth OR "text messag*" OR texting OR sms OR "short messag*" OR "multimedia messag*" OR "multi-media messag*" OR mms OR "instant messag*" OR "social media*" OR facebook OR twitter OR webcast* OR webinar* OR podcast* OR wiki OR wikis OR app OR apps OR android* OR blackberr* OR apple* OR ios OR iphone* OR ipad* OR s40 OR symbian* OR windows OR ((electronic* OR digital* OR device*) W2 tablet*) OR video* OR dvd OR dvds OR youtube OR "you tube" OR vimeo OR online OR "on line" or interactive OR "chat room*" OR chatroom* OR blog OR blogs OR web-log OR web-logs OR weblog OR weblogs OR "bulletin board*" OR bulletinboard$ OR messageboard$ OR "message board*" OR ehealth OR e-health OR mhealth OR m-health OR app OR apps OR pda OR pdas OR "personal digital" OR "device-based" OR email* OR e-mail* OR "electronic mail*")) | |
| S2 | (MH "Computer peripherals") OR (MH "Computer storage devices") OR (MH "Computer terminals") OR (MH | |

For peer review only

| | | |
|---|---|---|
| | "Microcomputers") OR (MH "Computers, hand-held") OR (MH "Attitude to computers") OR (MH "Computer systems") OR (MH "Medical informatics") OR (MH "Educational technology") OR (MH "Audiovisuals") OR (MH "Audiorecording") OR (MH "Videorecording") OR (MH "Multimedia") OR (MH "Computer Environment") OR (MH "Computer Assisted Instruction") OR (MH "Hypermedia") OR (MH "Video games") OR (MH "Mobile applications") OR (MH "Patient record systems") OR (MH "Computerized patient record") OR (MH "") OR (MH "Computer communication networks+") OR (MH "Telecommunications") OR (MH "Electronic Bulletin Boards") OR (MH "Electronic Mail") OR (MH "Instant Messaging") OR (MH "Interactive Voice Response Systems") OR (MH "Text Messaging") OR (MH "Cellular Phone") OR (MH "Telephone") OR (MH "Internet+") OR (MH "Remote Consultation") OR (MH "Telemedicine") OR (MH "Telehealth") OR (MH "Telenursing") OR (MH "Smartphone") OR (MH "User-Computer Interface+") | |
| S1 | (MH "Back Pain+") OR TI ("spinal pain* " OR "back pain*" OR lumbago OR "back acke*" OR backache OR (lumbar W2 pain*) OR (spin* W2 pain*)) OR AB ("spinal pain* " OR "back pain*" OR lumbago OR "back acke*" OR backache OR (lumbar W2 pain*) OR (spin* W2 pain*)) | |

Updated searches: 1) October 21 2016, 2) December 18 2018

**Cochrane Library - search details** (Through Wiley Online Library)

- Cochrane Database of Systematic Review (CDSR)
- Database of Reviews of Systematic Reviews (DARE, discontinued)
- Central Cochrane Register of Controlled Trials (CENTRAL)
- ‘Method studies’
- ‘Technology assessments’
- ‘Economic evaluations’

| | |
|---|---|
| #1 | (spinal next pain* or back next pain* or lumbago or back next acke* or backache or (lumbar near/2 pain*) or (spin* near/2 pain*)):ti,ab,kw |
| #2 | (computer* or microcomputer* or "pc" or "pcs" or "mac" or "macs" or "internet" or "www" or "web" or website* or webpage* or local next area next network* or "software" or cellular next phone* or cellular next telephone* or mobile* or cell next phone* or cell next telephone* or smartphone* or smart-phone* or smart-telephone* or handset* or hand-set* or "wireless" or "wire-less" or "wifi" or "wi-fi" or "gps" or global next positioning next system* or "bluetooth" or text next messag* or "texting" or "sms" or short next messag* or multimedia next messag* or multi-media next messag* or "mms" or instant next messag* or social next media* or "facebook" or "twitter" or webcast* or webinar* or podcast* or "wiki" or "wikis" or "app" or "apps" or android* or blackberr* or apple* or "ios" or iphone* or ipad* or "s40" or symbian* or "windows" or ((electronic* or digital* or device*) near/2 tablet*) or video* or "dvd" or "dvds" or "youtube" or "you tube" or "vimeo" or "online" or "on line" or "interactive" or chat next room* or chatroom* or "blog" or "blogs" or "web-log" or "web-logs" or "weblog" or "weblogs" or bulletin next board* or bulletinboard* or messageboard* or message next board* or "ehealth" or "e-health" or "mhealth" or "m-health" or "app" or "apps" |

For peer review only

| | |
|---|---|
| | or "pda" or "pdas" or "personal digital" or "device-based" or email* or e-mail* or electronic next mail*):ti,ab,kw |
| #3 | #1 and #2 |

Updated searches: 1) October 21 2016 (not shown), 2) December 18 2018 (below):

| | |
|---|---|
| #1 | (spinal next pain* or back next pain* or lumbago or back next acke* or backache or (lumbar near/2 pain*) or (spin* near/2 pain*)):ti,ab,kw |
| #2 | (computer* or microcomputer* or "pc" or "pcs" or "mac" or "macs" or "internet" or "www" or "web" or website* or webpage* or local next area next network* or "software" or cellular next phone* or cellular next telephone* or mobile* or cell next phone* or cell next telephone* or smartphone* or smart-phone* or smart-telephone* or handset* or hand-set* or "wireless" or "wire-less" or "wifi" or "wi-fi" or "gps" or global next positioning next system* or "bluetooth" or text next messag* or "texting" or "sms" or short next messag* or multimedia next messag* or multi-media next messag* or "mms" or instant next messag* or social next media* or "facebook" or "twitter" or webcast* or webinar* or podcast* or "wiki" or "wikis" or "app" or "apps" or android* or blackberr* or apple* or "ios" or iphone* or ipad* or "s40" or symbian* or "windows" or ((electronic* or digital* or device*) near/2 tablet*) or video* or "dvd" or "dvds" or "youtube" or "you tube" or "vimeo" or "online" or "on line" or "interactive" or chat next room* or chatroom* or "blog" or "blogs" or "web-log" or "web-logs" or "weblog" or "weblogs" or bulletin next board* or bulletinboard* or messageboard* or message next board* or "ehealth" or "e-health" or "mhealth" or "m-health" or "app" or "apps" or "pda" or "pdas" or "personal digital" or "device-based" or email* or e-mail* or electronic next mail*):ti,ab,kw |
| #3 | #1 and #2 |
| | With Publication Year from 2016 to 2018, with Cochrane Library publication date from Jan 2016 to Dec 2018, in Trials |

| | |
|---|---|
| #1 | (spinal next pain* or back next pain* or lumbago or back next acke* or backache or (lumbar near/2 pain*) or (spin* near/2 pain*)):ti,ab,kw |
| #2 | (computer* or microcomputer* or "pc" or "pcs" or "mac" or "macs" or "internet" or "www" or "web" or website* or webpage* or local next area next network* or "software" or cellular next phone* or cellular next telephone* or mobile* or cell next phone* or cell next telephone* or smartphone* or smart-phone* or smart-telephone* or handset* or hand-set* or "wireless" or "wire-less" or "wifi" or "wi-fi" or "gps" or global next positioning next system* or "bluetooth" or text next messag* or "texting" or "sms" or short next messag* or multimedia next messag* or multi-media next messag* or "mms" or instant next messag* or social next media* or "facebook" or "twitter" or webcast* or webinar* or podcast* or "wiki" or "wikis" or "app" or "apps" or android* or blackberr* or apple* or "ios" or iphone* or ipad* or "s40" or symbian* or "windows" or ((electronic* or digital* or device*) near/2 tablet*) or video* or "dvd" or "dvds" or "youtube" or "you tube" or "vimeo" or "online" or "on line" or "interactive" or chat next room* or chatroom* or "blog" or "blogs" or "web-log" or "web-logs" or "weblog" or "weblogs" or bulletin next board* or bulletinboard* or messageboard* or message next board* or "ehealth" or "e-health" or "mhealth" or "m-health" or "app" or "apps" or "pda" or "pdas" or "personal digital" or "device-based" or email* or e-mail* or electronic next mail*):ti,ab,kw |
| #3 | #1 and #2 |

For peer review only

| | |
|---|---|
| | With Cochrane Library publication date from Jan 2016 to Dec 2018, in Cochrane Reviews and Cochrane Protocols |

**PsycINFO - search details**

Ovid PsycINFO (R) 1987 to March Week 4 2016

| | |
|---|---|
| 1 | exp back pain/ or (spinal pain$ or back pain$ or lumbago or back ache$ or backache$ or (lumbar adj2 pain$) or (spin$ adj2 pain$)).ti,ab,id. |
| 2 | exp Human Computer Interaction/ or Computer Peripheral Devices/ or Computer Software/ or Human Machine Systems/ or exp Electronic Communication/ or exp Computers/ or exp Mobile Devices/ or exp Internet/ or exp Computer Applications/ or Computer Attitudes/ or Information Technology/ or exp AUDIOVISUAL INSTRUCTION/ or exp AUDIOVISUAL COMMUNICATIONS MEDIA/ or exp EDUCATIONAL AUDIOVISUAL AIDS/ or Telecommunications Media/ or Multimedia/ or exp Social media/ or exp Telephone systems/ or Telemedicine/ or exp Websites/ or (computer$ or microcomputer$ or pc or pcs or mac or macs or internet or www or web or website$ or webpage$ or local area network$ or software or cellular phone$ or cellular telephone$ or mobile$ or cell phone$ or cell telephone$ or smartphone$ or smart-phone$ or smart-telephone$ or handset$ or hand-set$ or wireless or wire-less or wifi or wi-fi or gps or global positioning system$ or bluetooth or text messag$ or texting or sms or short messag$ or multimedia messag$ or multi-media messag$ or mms or instant messag$ or social media$ or facebook or twitter or webcast$ or webinar$ or podcast$ or wiki or wikis or app or apps or android$ or blackberr$ or apple$ or ios or iphone$ or ipad$ or s40 or symbian$ or windows or ((electronic$ or digital$ or device$) adj2 tablet$) or video$ or dvd or dvds or youtube or you tube or vimeo or online or on line or interactive or chat room$ or chatroom$ or blog$1 or web-log$1 or weblog$1 or bulletin board$ or bulletinboard$ or messageboard$ or message board$ or ehealth or e-health or mhealth or m-health or app or apps or pda or pdas or personal digital or device-based or email$ or e-mail$ or electronic mail$).ti,ab,id. |
| 3 | 1 and 2 |
| 4 | limit 3 to yr="2000 -Current" |

Updated searches: 1) October 21 2016 (not shown), 2) December 18 2018 (below):

| | |
|---|---|
| 1 | exp back pain/ or (spinal pain$ or back pain$ or lumbago or back ache$ or backache$ or (lumbar adj2 pain$) or (spin$ adj2 pain$)).ti,ab,id. |
| 2 | exp Human Computer Interaction/ or Computer Peripheral Devices/ or Computer Software/ or Human Machine Systems/ or exp Electronic Communication/ or exp Computers/ or exp Mobile Devices/ or exp Internet/ or exp Computer Applications/ or Computer Attitudes/ or Information Technology/ or exp AUDIOVISUAL INSTRUCTION/ or exp AUDIOVISUAL COMMUNICATIONS MEDIA/ or exp EDUCATIONAL AUDIOVISUAL AIDS/ or Telecommunications Media/ or Multimedia/ or exp Social media/ or exp Telephone systems/ or Telemedicine/ or exp Websites/ |
| 3 | (computer$ or microcomputer$ or pc or pcs or mac or macs or internet or www or web or website$ or webpage$ or local area network$ or software or cellular phone$ or cellular telephone$ or mobile$ or cell phone$ or cell telephone$ or smartphone$ or smart-phone$ or smart-telephone$ or handset$ or hand-set$ or wireless or wire-less or wifi or wi-fi or gps or global positioning system$ or bluetooth or text messag$ or texting or sms or short messag$ or multimedia messag$ or multi-media messag$ or mms or instant messag$ or social media$ or facebook or twitter or webcast$ or webinar$ or podcast$ or wiki or wikis or app or apps or android$ or blackberr$ or apple$ or ios or iphone$ or ipad$ or s40 or symbian$ or windows or ((electronic$ or digital$ or device$) adj2 tablet$) or video$ or dvd or dvds or youtube or you tube or vimeo or online or on line or interactive or chat room$ or chatroom$ or blog$1 or web-log$1 or weblog$1 or bulletin board$ or bulletinboard$ or messageboard$ or message board$ or ehealth or e-health or mhealth or m-health or app or apps or pda or pdas or personal digital or device-based or email$ or e-mail$ or electronic mail$).ti,ab,id. |
| 4 | 1 and (2 or 3) |
| 5 | limit 4 to yr="2000 -Current" |
| 6 | 5 and (20161* or 2017* or 2018* or 2019*).up. |

For peer review only

**DoPHER - search details**

Database of Promoting Health Effectiveness Reviews
Focussed coverage of systematic and non-systematic reviews of effectiveness in health promotion and public health worldwide (3700).

Search date 11.04.2016

| | |
|---|---|
| 1 | Freetext (Year): >1999 |
| 2 | Freetext (All but Authors): "spinal pain" OR "back pain" OR "spinal pains" OR "back pains" OR lumbago OR "back ache" OR "back aches" OR "backache*" |
| 3 | 1 AND 2 |

Updated searches: 1) October 21 2016, 2) December 18 2018

**TROPHI - search details**

Trials Register of Promoting Health Interventions
Focussed coverage of trials of interventions in health promotion and public health worldwide. It covers both randomised and non-randomised controlled trials and currently contains details of over 7,750 trials.

Search date 11.04.2016

| | |
|---|---|
| 5 | Freetext (All but Authors): "spinal pain" OR "back pain" OR "spinal pains" OR "back pains" OR lumbago OR "back ache" OR "back aches" OR "backache*" |
| 6 | Freetext (Year): >1999 |
| 7 | 5 AND 6 |

Updated searches: 1) October 21 2016, 2) December 18 2018

**Web of Science - search details**
(Thomson Reuters)

Databases selected:
- Science Citation Index (SCI Expanded)
- Social Science Citation Index (SSCI)
- Conference Proceedings Citation Index – Science (CPCI-S)
- Conference Proceedings Citation Index – Social Science (SPCI-SSH)

Search date 6.4.2016

| | |
|---|---|
| #3 | #2 AND #1<br>*Indexes=SCI-EXPANDED, SSCI, CPCI-S, CPCI-SSH Timespan=2000-2016* |

For peer review only

| | |
|---|---|
| #2 | **TOPIC:** (computer$ OR microcomputer* OR pc OR pcs OR mac OR macs OR internet OR www OR web OR website* OR webpage* OR "local area network*" OR software OR "cellular phone*" OR "cellular telephone*" OR mobile* OR "cell phone*" OR "cell telephone*" OR smartphone* OR smart-phone* OR smart-telephone* OR handset* OR hand-set* OR wireless OR wire-less OR wifi OR wi-fi OR gps OR "global positioning system*" OR bluetooth OR "text messag*" OR texting OR sms OR "short messag*" OR "multimedia messag*" OR "multi-media messag*" OR mms OR "instant messag*" OR "social media*" OR facebook OR twitter OR webcast* OR webinar* OR podcast* OR wiki OR wikis OR app OR apps OR android* OR blackberr* OR apple* OR ios OR iphone* OR ipad* OR s40 OR symbian* OR windows OR ((electronic* OR digital* OR device*) NEAR/2 tablet*) OR video* OR dvd OR dvds OR youtube OR "you tube" OR vimeo OR online OR "on line" OR interactive OR "chat room*" OR chatroom* OR blog OR blogs OR web-log OR web-logs OR weblog OR weblogs OR "bulletin board*" OR bulletinboard* OR messageboard* OR "message board*" OR ehealth OR e-health OR mhealth OR m-health OR pda OR pdas OR "personal digital" OR "device-based" OR email* OR e-mail* OR "electronic mail*")<br>*Indexes=SCI-EXPANDED, SSCI, CPCI-S, CPCI-SSH Timespan=2000-2016* |
| #1 | **TOPIC:** ("spinal pain*" OR "back pain*" OR lumbago OR "back ache*" OR backache* OR lumbar NEAR/2 pain* OR spin* NEAR/2 pain*)<br>*Indexes=SCI-EXPANDED, SSCI, CPCI-S, CPCI-SSH Timespan=2000-2016* |

Updated searches: 1) October 21 2016, 2) December 18 2018

**OT Seeker - search details**

Occupational therapy systematic evaluation of evidence.
http://www.otseeker.com/Search/BasicSearch.aspx

| | |
|---|---|
| 1 | back pain AND (internet OR web) |

Updated searches: 1) October 21 2016, 2) December 18 2018

For peer review only

**Supplementary File 2:** Consensus summary of quality appraisal as per the 32-item Consolidated Criteria for Reporting Qualitative Research (COREQ) checklist (Booth et al., 2014; Tong et al., 2007) and comprehensiveness of reporting.

| No | Item | Guide questions | de Jong et al., 2009 | Caiata Zufferey & Schulz, 2009 | Schulz et al., 2010 | Nordin et al., 2017 | Rabbi et al., 2018 | Number of articles reporting each item (%) |
|---|---|---|---|---|---|---|---|---|
| **Domain 1: Research team and reflexivity** | | | | | | | | |
| **Personal characteristics** | | | | | | | | |
| **1** | Interviewer/facilitator | Which author/s conducted the interview or focus group? | N/R | N/R | N/R | Principal author | N/R | 1 (20%) |
| **2** | Credentials | What were the researcher's credentials? E.g. PhD, MD | N/R | N/R | N/R | PhD | PhD, PhD and MD | 2 (40%) |
| **3** | Occupation | What was their occupation at the time of the study? | N/R | N/R | N/R | N/R | N/R | 0 (0%) |
| **4** | Gender | Was the researcher male or female? | N/R | N/R | N/R | Female | N/R | 1 (20%) |
| **5** | Experience and training | What experience or training did the researcher have? | N/R | N/R | N/R | N/R | N/R | 0 (0%) |
| **Relationship with participants** | | | | | | | | |
| **6** | Relationship established | Was a relationship established prior to study commencement? | N/R | N/R | N/R | Participants had participated in the RCT, of which the qualitative study was a later part | N/R | 1 (20%) |
| **7** | Participant knowledge of the interviewer | What did the participants know about the researcher? e.g. | N/R | N/R | N/R | N/R | N/R | 0 (0%) |

For peer review only

| | | | | | | | | |
|---|---|---|---|---|---|---|---|---|
| | | personal goals, reasons for doing the research | | | | | | |
| **8** | Interviewer characteristics | What characteristics were reported about the interviewer/facilitator? e.g. Bias, assumptions, reasons and interests in the research topic | N/R | N/R | N/R | N/R | N/R | 0 (0%) |
| **Domain 2: Study design** | | | | | | | | |
| **Theoretical framework** | | | | | | | | |
| **9** | Methodological orientation and theory | What methodological orientation was stated to underpin the study? e.g. grounded theory, discourse analysis, ethnography, phenomenology, content analysis | Thematic analysis | Grounded theory | Inductive approach | Content Analysis | Thematic analysis | 2 (40%) |
| **Participant selection** | | | | | | | | |
| **10** | Sampling | How were participants selected? e.g. purposive, convenience, consecutive, snowball | Convenience | Purposive and convenience | Purposive and convenience | Consecutively | No selection, all participants of the DHI took part. | 5 (100%) |
| **11** | Method of approach | How were participants approached? e.g. face-to-face, telephone, mail, email | N/R | Email | Email | First approach not clear, but once given oral consent contacted by telephone | Method of sending invitations not clear. If eligible face-to-face meeting | 4 (80%) |
| **12** | Sample size | How many participants were in the study? | 11 OPs who recruited; 8 OPs who did not recruit & 9 employees | 18 | 18 | 19 | 10 | 5 (100%) |

For peer review only

| | | | | | | | | |
|---|---|---|---|---|---|---|---|---|
| **13** | Non-participation | How many people refused to participate or dropped out? Reasons? | 7 OPs who did not recruit; 15 employees. Reasons - no time, insufficient use of program, problems with recalling experiences | 238 approached to participate; 32 responded; 14 of these did not participate – reasons not stated | N/R | 3 – reasons not stated | None | 4 (80%) |
| **Setting** | | | | | | | | |
| **14** | Setting of data collection | Where was the data collected? e.g. home, clinic, workplace | Telephone interviews | Home or University | Home or University | Health Care Centres, County City Buildings, Participant's home | Web-based exit survey | 5 (100%) |
| **15** | Presence of non-participants | Was anyone else present besides the participants and researchers? | N/R | N/R | N/R | N/R | N/R | 0 (0%) |
| **16** | Description of sample | What are the important characteristics of the sample? e.g. demographic data, date | Not stated for OPs; Employees 67% male; 40-50 years; 75% LBP; white & blue-collar workers; varying educational levels; varying sickness absence levels due to LBP | 9 females, 9 males; 28-72 years; chronic LBP for 1-30 years; mix of diagnoses including 8 with no clear diagnosis; all had at least secondary school education (5 had degree); | 9 females, 9 males; 28-72 years; chronic LBP 1-30 years ; mixed diagnoses, varied level of education and frequency of website use | 15 females, 4 males; mean age 45; MSK pain for average 7.5 years; most at least secondary education; majority working. | 7 females, 3 males; 31-60 years; chronic LBP 5-33 years duration; mixed diagnoses. | 5 (100%) |

For peer review only

| | | | | | | | | |
|---|---|---|---|---|---|---|---|---|
| | | | 7weeks-6 months | range of website use amongst participants | | | | |
| **Data collection** | | | | | | | | |
| **17** | Interview guide | Were questions, prompts, guides provided by the authors? Was it pilot tested? | Topic guides used. Pilot tested | No questions, prompts or guides provided; Piloting not reported | No questions, prompts or guides provided; Piloting not reported | No questions, prompts or guides provided; Piloting not reported | Open-ended question in web survey provided. Piloting not reported | 2 (40%) |
| **18** | Repeat interviews | Were repeat interviews carried out? If yes, how many? | N/R | N/R | N/R | N/R | N/R | 0 (0%) |
| **19** | Audio/visual recording | Did the research use audio or visual recording to collect the data? | Audio recorded | Not specifically stated "Recorded" and transcribed verbatim | Audio recorded | Audio recorded | No – used free text web survey | 5 (100%) |
| **20** | Field notes | Were field notes made during and/or after the interview or focus group? | N/R | N/R | N/R | N/R | N/R | 0 (0%) |
| **21** | Duration | What was the duration of the interviews or focus group? | Approx. 30 minutes | Approx. 45 minutes | Approx. 45 minutes | 31 – 56 minutes. Mean 48 minutes | N/R | 4 (80%) |
| **22** | Data saturation | Was data saturation discussed? | Yes | Yes | N/R | N/R | N/R | 2 (40%) |
| **23** | Transcripts returned | Were transcripts returned to participants for comment and/or correction? | N/R | N/R | N/R | N/R | N/R | 0 (0%) |
| **Domain 3: Analysis and findings** | | | | | | | | |

For peer review only

| | | | | | | | | |
|---|---|---|---|---|---|---|---|---|
| **Data analysis** | | | | | | | | |
| **24** | Number of data coders | How many data coders coded the data? | N/R | N/R | N/R | 4 | N/R | 1 (20%) |
| **25** | Description of coding tree | Did authors provide a description of the coding tree? | N/R | N/R | N/R | Yes | N/R | 1 (20%) |
| **26** | Derivation of themes | Were themes identified in advance or derived from the data? | Derived from data | Derived from data | Essentially inductive | Derived from data | Derived from data | 5 (100%) |
| **27** | Software | What software, if applicable, was used to manage the data? | Excel | ATLAS.ti | ATLAS.ti | Open Code | N/R | 4 (80%) |
| **28** | Participant checking | Did participants provide feedback on the findings? | N/R | N/R | N/R | N/R | N/R | 0 (0%) |
| **Reporting** | | | | | | | | |
| **29** | Quotations presented | Were participant quotations presented to illustrate the themes / findings? Was each quotation identified? e.g. participant number | Few direct quotes; only identified as either OP or employee | Yes - identified by gender, age & occupation | Yes - identified by gender, age & occupation | Yes – identified by participant number and gender | Yes – identified by participant number | 5 (100%) |
| **30** | Data and findings consistent | Was there consistency between the data presented and the findings? | A little unclear – little qualitative data presented | Yes | Yes | Yes | Yes | 5 (100%) |
| **31** | Clarity of major themes | Were major themes clearly presented in the findings? | Yes | Yes | Yes | Yes | Yes | 5 (100%) |
| **32** | Clarity of minor themes | Is there a description of diverse cases or discussion of minor themes? | Not clear | Range of themes presented but not clear what is major/minor | Range of themes presented but not clear what is major/minor | Yes | Yes | 2 (40%) |

For peer review only

For peer review only - http://bmjopen.bmj.com/site/about/guidelines.xhtml

| | | | | | | |
|---|---|---|---|---|---|---|
| **TOTAL, number (%)** | 14 (44%) | 15 (47%) | 12 (38%) | 21 (67%) | 14 (44%) | |
| **DHI: digital health intervention; LBP: low back pain; N/R: not reported; OPs: occupational physicians;** | | | | | | |

## References

Booth A, Hannes K, Harden A, Noyes J, Harris J, Tong A. COREQ (Consolidated Criteria for Reporting Qualitative Studies). In: Moher D, Altman DG, Schulz KF, Simera I, Wager E, editors. Guidelines for reporting health research: A user's manual. Oxford: Wiley; 2014. p. 214-26.

Caiata Zufferey M, Schulz PJ. Self-management of chronic low back pain: an exploration of the impact of a patient-centered website. Patient education and counseling. 2009;77(1):27-32.

de Jong T, Heinrich J, Blatter BM, Anema JR, van der Beek AJ. The feasibility of a web-based counselling program for occupational physicians and employees on sick leave due to back or neck pain. BMC medical informatics and decision making. 2009;9:46.

Nordin C, Michaelson P, Eriksson MK, Gard G. It's About Me: Patients' Experiences of Patient Participation in the Web Behavior Change Program for Activity in Combination With Multimodal Pain Rehabilitation. Journal of medical Internet research. 2017;19(1):e22-e.

Rabbi M, Aung MS, Gay G, Reid MC, Choudhury T. Feasibility and Acceptability of Mobile Phone-Based Auto-Personalized Physical Activity Recommendations for Chronic Pain Self-Management: Pilot Study on Adults. Journal of medical Internet research. 2018;20(10):e10147-e.

Schulz PJ, Rubinelli S, Zufferey MC, Hartung U. Coping with Chronic Lower Back Pain: Designing and Testing the Online Tool ONESELF. Journal of Computer-Mediated Communication. 2010;15(4):625-45.

Tong A, Sainsbury P, Craig J. Consolidated criteria for reporting qualitative research (COREQ): a 32-item checklist for interviews and focus groups. International journal for quality in health care : journal of the International Society for Quality in Health Care. 2007;19(6):349-57.

For peer review only

**Supplementary File 3:** Taxonomy of barriers and facilitators with exemplar quotations

**References**:

[27] Schulz PJ, Rubinelli S, Zufferey MC, Hartung U. Coping with Chronic Lower Back Pain: Designing and Testing the Online Tool ONESELF. Journal of Computer-Mediated Communication. 2010;15(4):625-45.
[28] Caiata Zufferey M, Schulz PJ. Self-management of chronic low back pain: an exploration of the impact of a patient-centered website. Patient education and counseling. 2009;77(1):27-32.
[36] de Jong T, Heinrich J, Blatter BM, Anema JR, van der Beek AJ. The feasibility of a web-based counselling program for occupational physicians and employees on sick leave due to back or neck pain. BMC medical informatics and decision making. 2009;9:46.
[37] Rabbi M, Aung MS, Gay G, Reid MC, Choudhury T. Feasibility and Acceptability of Mobile Phone-Based Auto-Personalized Physical Activity Recommendations for Chronic Pain Self-Management: Pilot Study on Adults. Journal of medical Internet research. 2018;20(10):e10147-e.
[38] Nordin C, Michaelson P, Eriksson MK, Gard G. It's About Me: Patients' Experiences of Patient Participation in the Web Behavior Change Program for Activity in Combination With Multimodal Pain Rehabilitation. Journal of medical Internet research. 2017;19(1):e22-e.

| **Barriers and facilitators for patient uptake and utilisation of digital self-management interventions for LBP** | | | | | |
|---|---|---|---|---|---|
| **Theme** | **Taxonomy** | **Barriers** | **Exemplar quotations** | **Facilitators** | **Exemplar quotations** |
| IT usability and accessibility | Functionality and usability | • Too much choice between functions<br>• Fixed advancement pace<br>• Issues logging into DHI<br>• *Low user-friendliness<br>• *Issues logging into DHI<br>• *Low level of functionality (e.g. registration, navigation, helpdesk) | • Though, the freedom of choice in the Web-BCPA entailed perceptions of restrained patient participation for some informants [38, p4]<br>• Finally, some OPs faced practical obstacles such as log-in problems […] [36, p5]<br>• Although OPs were generally positive about the user-friendliness and | • Flexible structure and navigation<br>• Conveniently arranged<br>• Variation of media types (text, audio and video)<br>• Reminders and notifications<br>• High user-friendliness<br>• *High user-friendliness | • *I liked this thing about the exercise video a lot because seeing it with the video gives you a lot more. They seem simple, but a lot of times when there are drawings I can't understand them easily, then I don't have the will anymore [28, p29]*<br>• It was enough to open the mailbox for reasons that could be independent of cLBP |

For peer review only

| | | | | | |
|---|---|---|---|---|---|
| | | | design of the program, some felt that further improving user-friendliness (functionality) might enhance its use. […] It should also be easier to register employees in the program [36, p6]<br>• Although, some informants perceived restrained patient participation by the fact that […] not being able to select a faster advancement in the program by themselves [36, p5]<br>• A small number of employees either had problems with 'logging in into the program' […] [36, p6] | | to get a reminder of the website and the necessity of self-management […] *I usually went on the website when I read the newsletter. I read the letter and then I'm there, it's like a conditioned reflex [27, p641]*<br>• *It would be helpful to have reminders and suggestions pop up in the morning or at other chosen times. This could be optional and set by the user [37, p10]*<br>• These effects could be reached thanks to the specificities of the website, that is its […] multimediality (material was provided in written, audio and video form), usability (the website was easy to use […]) [28, p31]<br>• They [the users] were positive about the content, user-friendliness and web-based design. They |

For peer review only

| | | | | | |
|---|---|---|---|---|---|
| | | | | | said the information was easy to understand, to the point and conveniently arranged [36, p5]<br>• Finally, almost all OPs were positive about the user-friendliness and design of the program [36, p5] |
| | IT affinity | • Lack of affinity with computers<br>• *Lack of affinity with web-based programmes | • Some OPs had no affinity with the use of a web-based program in general and therefore preferred not to use this method [36, p5]<br>• A small number of employees […] had 'no affinity with computers' [36, p6] | • Enjoying working with a computer | • In addition, some informants stated that […] to enjoy working at the computer, facilitated patient participation in the rehabilitation [38, p6] |
| | Access and convenience | • Not able to choose starting time of DHI<br>• *No access to computer during consultation | • Although, some informants perceived restrained patient participation by the fact that they were not able to choose the starting time of the Web-BCPA course themselves (due to study protocol) […] [38, p5]<br>• Finally, some OPs faced practical | • Easily accessible with low effort<br>• Accessible at all hours and locations<br>• Accessible even during periods with severe pain symptoms<br>• Ability to take all the time needed | • Patient participation was emphasized by having access to the Web-BCPA on computer or tablet at all hours and locations [38, p5]<br>• The opportunities to work in the Web-BCPA at home were experienced to provide continuity in |

For peer review only

| | | | | | |
|---|---|---|---|---|---|
| | | | obstacles such as […] no access to a computer or the internet in their consulting rooms [36, p5] | | the rehabilitation [38, p5]<br>• These effects could be reached thanks to the specificities of the website, that is its usability ([…] accessible from home without the necessity of intermediaries) [28, p31]<br>• […] informants described that the Web-BCPA provided opportunities to rehabilitation during periods with severe symptoms without having to be present at the health care center [38, p6] |
| Quality and quantity of content | Quality of content | • Contradicting content between DHI and HCP | • For some employees the exercises suggested by the program conflicted with the exercises given by the physiotherapist [36, p5] | • Trustworthy content and source<br>• Easily understandable content<br>• High quality of content<br>• Steady content<br>• *Appropriate content | • *Knowing there is a serious website where there are contributions, it strengthens you a bit [28, p29]*<br>• Some users felt reassured because they had a trustworthy place where they could address concerns [27, p641] |

For peer review only

| | | | | | |
|---|---|---|---|---|---|
| | | | | | • These effects could be reached thanks to the specificities of the website, that is its […] trustworthiness (material was controlled by health professionals according to the criteria of Evidence Based Medicine) [28, p31]<br>• They [the users] were positive about the content, user-friendliness and web-based design. They said the information was easy to understand, to the point and conveniently arranged [36, p5]<br>• More than half of the OPs were positive about the content (e.g. information, exercises, instructions) [36, p5]<br>• […] the stability of the material helped them to construct their personal frame of reference about the nature and the |

For peer review only

| | | | | | |
|---|---|---|---|---|---|
| | | | | | course of their cLBP [27, p640] |
| | Amount of content | • Too much content to choose from<br>• Too much information to fully comprehend | • According to some people, Oneself provided too much information, risking creating confusion about the comprehension of the health problem and the identification of the best way to treat it: *There is a lot of information, probably almost too much, don't you think? [28, p29]*<br>• [...] having difficulties to choose from its content, were experienced to restrain patient participation [38, p8] | • A lot of content to choose from | • The richness and trustworthiness of the information [...] helped them to construct their personal frame of reference about the nature and the course of their cLBP [28, p28]<br>• First, the quality and continual update of the website encouraged people to visit Oneself again and to continue thinking about self-management [28, p29] |
| Tailoring and personalisation | Tailoring, specificity and personalisation | • Content not tailored to individual needs and/or pain severity<br>• Content perceived not new or relevant | • [...] because some of the advice and exercises were not specific enough, they did not apply to the employee's situation [36, p5] | • Content accounting for individual needs and/or pain severity<br>• Self-identification in content<br>• Opportunity to influence treatment | • *..it was obvious that it (the rehabilitation) was about me, it wasn't about just anyone.. it was about my problems, my strengths and how I felt.. they (the HCPs* |

| | | | | | |
|---|---|---|---|---|---|
| | | | • Some persons perceived information not new nor relevant. In this case, the use of Oneself lead to feelings of hopelessness: two participants had the impression that again there was no solution for their problem [28, p29]<br>• *The exercises that you have on the website are good, but I can't do any of them, no. I tried to do them a bit on the bed, but with my arm that doesn't work, my knees that don't work... There are lots, indeed I had written down those that I could do, but then many times your will is missing (...) Then you get sick of it. I know, that it's for my own good that I should exercise, but after a while I... Then you don't have grand results, and so even* | | *started from a blank page, I was not fitted into an average template of how it ought to be.. it (the rehabilitation) started with my point of view [38, p4-5]*<br>• *I really liked the personalization. I thought it was a nice touch. Suggestions were more specific and tailored, which for me made them more relevant and likely for me to use them [37, p9]*<br>• *Previously I had read about CBT (Cognitive Behavioral Therapy), but I had never thought of it as a help for my condition.. I want to compare this rehabilitation with a smorgasbord from which is it easy to taste [38, p5]*<br>• *It gives you descriptions and you say: this stuff here.. I see it, I see it! I recognise myself in it,* |

For peer review only

| | | | | | |
|---|---|---|---|---|---|
| | | | *for my back sometimes I go through periods, moments where I'm, let's say, very diligent, and then sometimes… (…) Yes, it's interesting. But there are always the same things that you then don't do [28, p29]* | | *I recognise myself here [27, p640]*<br>• Informants experienced that being able to identify themselves with the content in the rehabilitation and finding it trustworthy were important to patient participation and being confirmed [38, p5]<br>• They [informants] described that they were confirmed when they could identify their illness experience and life situation, as well as their own thoughts and cognitions about their pain condition, in the texts and the assignments of the Web-BCPA [38, p7] |
| Motivation and support | Personal attributes and resources | • Adhering to biomedical model of LBP<br>• Seeing LBP as a marginal problem<br>• Preferring other treatment regimens, e.g. with human contact | • *I went to a doctor who told me 'there is nothing to do, just resign yourself to it'. So this unleashed really the research to find something. But after eight years I didn't find the magic* | • High level of awareness and self-management of LBP<br>• Aware that LBP would not be fixed with a medical solution and ready to accept active role | • In addition, some informants stated that their work experience, such as having a solution-focused work […] facilitated patient participation in the rehabilitation [38, p6] |

For peer review only

| | | | | | |
|---|---|---|---|---|---|
| | | • Lack of knowledge about LBP and treatments<br>• Physical health (e.g. pain, fatigue)<br>• Psychological symptoms | *cure, unfortunately. And one continuously hears 'they are doing new research!' But hopefully they will arrive in time in order to do something. (…) I'm always in search of the super novelty, the one that heals [28, p30]*<br>• One employee mentioned that the back or neck pain they were suffering from may have prevented them from sitting at a computer [36, p6]<br>• Pain, fatigue and other psychological symptoms were perceived to limit patient participation [38, p6]<br>• Three users could be defined as passive self-managers: They adhered to a traditional biomedical model of cLBP and were convinced that the solution of their problem had to be | • Emotional and cognitive resources, e.g. motivation, interest, commitment and self-confidence in self-management of LBP<br>• Enjoy solution focused work | • *I already know which road I have to follow in detail. I need details or confirmation on these details [28, p29]*<br>• They described emotions and cognitions that affected patient participation. Having motivation, interest, commitment, and self-confidence were perceived to favor patient participation [38, p6]<br>• Most of the users could be defined as experienced self-managers, in the sense that they had a rather high level of awareness and self-management of cLBP even before knowing Oneself. These people […] had a rather clear idea about their diagnosis, and knew that they had to play an active role in dealing with their health problem [27, p635] |

For peer review only

| | | | | | |
|---|---|---|---|---|---|
| | | | found by health professionals. These people went to Oneself to find a definitive medical solution for their cLBP [27, p635]<br>• Three users could be defined as latent self-managers. […] For all of them, cLBP was at the moment a marginal problem, in the sense that it was intermittent and light. These users did not really need to engage in a long-term process of self-management: When pain appeared, they usually dealt with it through some easy coping strategies, such as taking painkillers, going to the chiropractic, etc [27, p636] | | • Two users could be defined as novices in terms of self-management. These participants were aware that a medical solution to cLBP did not exist and were ready to accept that they had to become actively involved in their cLBP care. However, they did not know how to do it [27, p635] |
| | Support to use DHI | • HCP unsupportive of use of DHI<br>• No support from authorities | • *I planned to complete the program (the Web-BCPA).. I am not sure how much I had left.. probably the last module.. but I was denied sick-leave* | • HCP supportive of use of DHI<br>• Support from family<br>• Support from authorities<br>• Support from other suffers (e.g. successful testimonials) | • *It's nice knowing that there is someone else [28, p29]*<br>• *When you are going through a moment when you have backache and you* |

| | | | | | |
|---|---|---|---|---|---|
| | | | *compensation by the Social Insurance Agency and had to put in a lot of energy to explain my situation and meet with the psychosocial counsellor.. I did not have the strength to do anything else.. I have used so much energy to fight for my cause [12052, 6]*<br>• One employee said, *I expected more commitment from my OP.* This did not encourage employees to use the program [2120, 5] | | *read a testimony which says 'yes, there is someone who was able to do it', it gives you hope [28, p29]*<br>• Support, trust and respect from a family member, employer, the Swedish Social Insurance Agency (SSIA) or the Employment Service were experienced to facilitate patient participation in the rehabilitation [38, p6] |
| | Features of DHI | • DHI not guiding or supporting participants enough (e.g. to plan for execution of physical activity recommendation from DHI) | • *I received the suggestion to ride a bike, but that's currently simply not possible, logistically [37, p10]*<br>• *If it could ask me to rank the things I enjoy doing and then download weather data for the following days. This could suggest times when I have performed these tasks in the* | • Interaction/interactivity<br>• Information about self-management of LBP<br>• Goal-setting<br>• Action-planning<br>• Follow-up and evaluation<br>• Adjusting treatment related to setbacks and progress<br>• Monitoring own progress in graphs<br>• Variation of content<br>• Update of content | • To acquire knowledge and insights were thought of as patient participation, and included self-reflection, self-identification, and feedback [38, p5]<br>• [...] with opportunities to influence and a variety of treatments to choose according to one's own needs and priorities [38, p5] |

For peer review only

| | | | | | |
|---|---|---|---|---|---|
| | | | *past and also match it with weather predictions. "You played tennis for last Tuesday in the afternoon for 90 minutes. How about from 2 to 4 today when the weather will be clear and 85". [37, p10]* | | • To adjust a goal or treatment planning in relation to progress or setback was described as patient participation: *I feel it is important to set goals and to follow-up those goals.. and to why a goal is reached and why another is not.. this made me aware of that I needed other tools (in the rehabilitation)* [38, p6]<br>• Patient participation was reported when informants monitored results shown by the interactive graphs in the Web-BCPA: .. *days when I had a lot of pain I used to remain sedentary, and as soon as I had a better day I was eager to do all kinds of activities that day.. before I started with the assignment activity planning (in the Web-BCPA) I was* |

For peer review only

| | | | | | |
|---|---|---|---|---|---|
| | | | | | *not aware of how my behaviour related to the days with pain, but by monitoring this over time I started to plan my daily activities in a more balanced way [38, p6]*<br>• These effects could be reached thanks to the specificities of the website, that is its interactivity (people could ask specific questions to health professionals who were available daily for responding), […] dynamism (the website was updated weekly) […] [28, p31]<br>• The informants' experienced patient participation when they analyzed their situation taken into account their resources and restrictions, set goals for behavior change, and planned treatments and activities. Also, patient participation was stated when |

For peer review only

| | | | | | |
|---|---|---|---|---|---|
| | | | | | treatments, self-care, and planning were followed-up and evaluated [38, p6] |
| | HCP factors for support of patients | • *Time restrictions of consultations<br>• *Difficulty keeping DHI in mind during consultations<br>• *Difficulty providing patients with accurate information about DHI<br>• *Perceiving no benefit of DHI compared to usual treatment<br>• *Preferring other treatment regimens, e.g. with human contact | • *It takes time to get used to the recruitment process and to using the program [36, p5]*<br>• A second important barrier for OPs was the limited time available for introducing employees to the program and working with it as well. […] *We lack the time to do this kind of projects [36, p5]*<br>• One OP stated that he did not use the program because he did not believe in 'computer-based treatment' of physical pain. He explained, *The ability to touch people is an essential element in the treatment of people with back or neck pain. [36, p5]* | • *DHI a good medium for counselling employees | • About half of the OPs indicated that a website is a good medium for counselling of employees with back or neck pain [36, p5] |

For peer review only - http://bmjopen.bmj.com/site/about/guidelines.xhtml

| | | | | | |
|---|---|---|---|---|---|
| | | | • One OP stated that he was quite capable of managing the RTW process himself and did not need a program for additional support. Many preferred the more familiar therapies (e.g. physiotherapy) […]. They preferred having personal contact with employees [36, p5] | | |

*= HCP perspective; **IT**: information technology; **HCP**: healthcare professional; **DHI**: Digital health intervention

For peer review only

# PRISMA 2009 Checklist

| Section/topic | # | Checklist item | Reported on page # |
|---|---|---|---|
| **TITLE** | | | |
| Title | 1 | Identify the report as a systematic review, meta-analysis, or both. | #1 lines 1-3 |
| **ABSTRACT** | | | |
| Structured summary | 2 | Provide a structured summary including, as applicable: background; objectives; data sources; study eligibility criteria, participants, and interventions; study appraisal and synthesis methods; results; limitations; conclusions and implications of key findings; systematic review registration number. | #2 lines 35-74 |
| **INTRODUCTION** | | | |
| Rationale | 3 | Describe the rationale for the review in the context of what is already known. | 4-6 |
| Objectives | 4 | Provide an explicit statement of questions being addressed with reference to participants, interventions, comparisons, outcomes, and study design (PICOS). | #5 lines 136-139 |
| **METHODS** | | | |
| Protocol and registration | 5 | Indicate if a review protocol exists, if and where it can be accessed (e.g., Web address), and, if available, provide registration information including registration number. | #6 line 146 |
| Eligibility criteria | 6 | Specify study characteristics (e.g., PICOS, length of follow-up) and report characteristics (e.g., years considered, language, publication status) used as criteria for eligibility, giving rationale. | #7 (Table 1) |
| Information sources | 7 | Describe all information sources (e.g., databases with dates of coverage, contact with study authors to identify additional studies) in the search and date last searched. | #8 |
| Search | 8 | Present full electronic search strategy for at least one database, including any limits used, such that it could be repeated. | #8 & Suppl. file 1 |
| Study selection | 9 | State the process for selecting studies (i.e., screening, eligibility, included in systematic review, and, if applicable, included in the meta-analysis). | #8 |
| Data collection process | 10 | Describe method of data extraction from reports (e.g., piloted forms, independently, in duplicate) and any processes for obtaining and confirming data from investigators. | #9 |
| Data items | 11 | List and define all variables for which data were sought (e.g., PICOS, funding sources) and any assumptions and simplifications made. | #8 |
| Risk of bias in individual studies | 12 | Describe methods used for assessing risk of bias of individual studies (including specification of whether this was done at the study or outcome level), and how this information is to be used in any data synthesis. | #9 |
| Summary measures | 13 | State the principal summary measures (e.g., risk ratio, difference in means). | #9-10 |

For peer review only - http://bmjopen.bmj.com/site/about/guidelines.xhtml

# PRISMA 2009 Checklist

| | | | |
|---|---|---|---|
| Synthesis of results | 14 | Describe the methods of handling data and combining results of studies, if done, including measures of consistency (e.g., $I^2$) for each meta-analysis. | #9-10 |



| Section/topic | # | Checklist item | Reported on page # |
|---|---|---|---|
| Risk of bias across studies | 15 | Specify any assessment of risk of bias that may affect the cumulative evidence (e.g., publication bias, selective reporting within studies). | #31 |
| Additional analyses | 16 | Describe methods of additional analyses (e.g., sensitivity or subgroup analyses, meta-regression), if done, indicating which were pre-specified. | N/A |
| **RESULTS** | | | |
| Study selection | 17 | Give numbers of studies screened, assessed for eligibility, and included in the review, with reasons for exclusions at each stage, ideally with a flow diagram. | #11 |
| Study characteristics | 18 | For each study, present characteristics for which data were extracted (e.g., study size, PICOS, follow-up period) and provide the citations. | #12-15 |
| Risk of bias within studies | 19 | Present data on risk of bias of each study and, if available, any outcome level assessment (see item 12). | #18 & Suppl. File 2 |
| Results of individual studies | 20 | For all outcomes considered (benefits or harms), present, for each study: (a) simple summary data for each intervention group (b) effect estimates and confidence intervals, ideally with a forest plot. | #18-26 (No quantitative assessment) |
| Synthesis of results | 21 | Present results of each meta-analysis done, including confidence intervals and measures of consistency. | #18-26 (No quantitative assessment) |
| Risk of bias across studies | 22 | Present results of any assessment of risk of bias across studies (see Item 15). | N/A (No quantitative assessment) |
| Additional analysis | 23 | Give results of additional analyses, if done (e.g., sensitivity or subgroup analyses, meta-regression [see Item 16]). | N/A |
| **DISCUSSION** | | | |
| Summary of evidence | 24 | Summarize the main findings including the strength of evidence for each main outcome; consider their relevance to key groups (e.g., healthcare providers, users, and policy makers). | #26-30 |
| Limitations | 25 | Discuss limitations at study and outcome level (e.g., risk of bias), and at review-level (e.g., incomplete retrieval of identified research, reporting bias). | #30-31 |
| Conclusions | 26 | Provide a general interpretation of the results in the context of other evidence, and implications for future | #32 |

For peer review only - http://bmjopen.bmj.com/site/about/guidelines.xhtml

# PRISMA 2009 Checklist

| | | | |
|---|---|---|---|
| | | research. | |
| **FUNDING** | | | |
| Funding | 27 | Describe sources of funding for the systematic review and other support (e.g., supply of data); role of funders for the systematic review. | #33 line 610-611 |

*From:* Moher D, Liberati A, Tetzlaff J, Altman DG, The PRISMA Group (2009). Preferred Reporting Items for Systematic Reviews and Meta-Analyses: The PRISMA Statement. PLoS Med 6(7): e1000097. doi:10.1371/journal.pmed1000097

For more information, visit: www.prisma-statement.org.



For peer review only
